# Transcriptional analysis of immune-related gene expression in p53-deficient mice with increased susceptibility to influenza A virus infection

**DOI:** 10.1186/s12920-015-0127-8

**Published:** 2015-08-18

**Authors:** Wenjun Yan, Jianchao Wei, Xufang Deng, Zixue Shi, Zixiang Zhu, Donghua Shao, Beibei Li, Shaohui Wang, Guangzhi Tong, Zhiyong Ma

**Affiliations:** Shanghai Veterinary Research Institute, Chinese Academy of Agricultural Science, No. 518, Ziyue Road, Shanghai,, 200241 PR China; Lanzhou Veterinary Research Institute, Chinese Academy of Agricultural Sciences, Lanzhou, 730046 China

## Abstract

**Background:**

p53 is a tumor suppressor that contributes to the host immune response against viral infections in addition to its well-established protective role against cancer development. In response to influenza A virus (IAV) infection, p53 is activated and plays an essential role in inhibiting IAV replication. As a transcription factor, p53 regulates the expression of a range of downstream responsive genes either directly or indirectly in response to viral infection. We compared the expression profiles of immune-related genes between IAV-infected wild-type p53 (p53WT) and p53-deficient (p53KO) mice to gain an insight into the basis of p53-mediated antiviral response.

**Methods:**

p53KO and p53WT mice were infected with influenza A/Puerto Rico/8/1934 (PR8) strain. Clinical symptoms and body weight changes were monitored daily. Lung specimens of IAV-infected mice were collected for analysis of virus titers and gene expression profiles. The difference in immune-related gene expression levels between IAV-infected p53KO and p53WT mice was comparatively determined using microarray analysis and confirmed by quantitative real-time reverse transcription polymerase chain reaction.

**Results:**

p53KO mice showed an increased susceptibility to IAV infection compared to p53WT mice. Microarray analysis of gene expression profiles in the lungs of IAV-infected mice indicated that the increased susceptibility was associated with significantly changed expression levels in a range of immune-related genes in IAV-infected p53KO mice. A significantly attenuated expression of *Ifng* (encoding interferon (IFN)-gamma), *Irf7* (encoding IFN regulator factor 7), and antiviral genes, such as *Mx2* and *Eif2ak2* (encoding PKR), were observed in IAV-infected p53KO mice, suggesting an impaired IFN-mediated immune response against IAV infection in the absence of p53. In addition, dysregulated expression levels of proinflammatory cytokines and chemokines, such as *Ccl2* (encoding MCP-1), *Cxcl9*, *Cxcl10* (encoding IP-10), and *Tnf*, were detected in IAV-infected p53KO mice during early IAV infection, reflecting an aberrant inflammatory response.

**Conclusion:**

Lack of p53 resulted in the impaired expression of genes involved in IFN signaling and the dysregulated expression of cytokine and chemokine genes in IAV-infected mice, suggesting an essential role of p53 in the regulation of antiviral and inflammatory responses during IAV infection.

**Electronic supplementary material:**

The online version of this article (doi:10.1186/s12920-015-0127-8) contains supplementary material, which is available to authorized users.

## Background

Influenza A virus (IAV) is a member of the *Orthomyxoviridae* family of RNA viruses and a primary cause of respiratory tract infections that result in approximately 500,000 deaths per year worldwide [1]. IAV evokes the host immune response to inhibit viral replication and clear viral infections. Meanwhile, an aberrant host immune response during IAV infection has been hypothesized to be the main cause of IAV-related pneumonia. The host immune response to IAV infection has been extensively studied for more than 70 years; however, many uncertainties still exist [2]. For example, host gene involvement in both the host immune response and IAV pathogenesis remain unclear [3, 4].

The p53 protein is a major tumor suppressor that plays important roles in regulating various cellular activities, including cell cycle arrest, DNA repair, senescence, and apoptosis [5]. The p53 protein primarily functions as a transcription factor that positively and negatively regulates the expression of a large and disparate group of responsive genes [6]. In addition to its well-established role in protecting against cancer development, p53 has been recently shown to contribute to the host immune response against viral infections due to vesicular stomatitis virus, Newcastle disease virus, and hepatitis C virus [7–9]. The expression of p53 can be induced at the transcriptional level by type I interferon (IFN). The IFN-stimulated response elements have been identified in p53 gene [7]. On the other hand, p53 upregulates the expression of several IFN-inducible proteins, including IFN regulatory factor (IRF) 9, IRF5, IFN-stimulated gene 15, and toll-like receptor 3, suggesting a crosstalk between the p53 and IFN pathways [10].

In response to IAV infection, p53 is significantly upregulated and activated in cultured cells [11–13] as well as in the lungs of IAV-infected mice [14]. Previous studies indicated that p53 activation plays an essential role in inhibiting IAV replication and regulating apoptosis of IAV-infected cells [11, 15]. In this study, we observed increased mortality, severe weight loss, and increased viral loads in the lungs of p53-deficient mice after IAV infection, indicating an increased susceptibility to IAV. It is known that p53, as a transcription factor, upregulates or downregulates a series of immune-related genes either directly or indirectly in response to viral infection [10, 16]. To gain an insight into the basis of different susceptibilities to IAV infection, we compared the expression profiles of immune-related genes in the lung tissues of IAV-infected p53WT and p53 knockout (p53KO) mice and found that a range of immune-related genes involved in the regulation of host immune and inflammatory responses showed significantly altered expression levels in the absence of p53.

## Methods

### Virus and mice infection

Influenza A/Puerto Rico/8/1934 (PR8) (H1N1 subtype) virus was propagated in the allantoic cavities of 9-day-old embryonated specific-pathogen-free (SPF) chicken eggs. The lethal dose to 50 % (LD_50_) of the test animals due to the PR8 virus was measured by intranasally infecting p53WT C57BL/6 mice and calculated using the method of Reed and Muench [17]. Homozygous p53^+/+^ (p53WT) and p53^−/−^ (p53KO) mice on C57BL/6 backgrounds were obtained from a breeding colony at the SPF facility of the Shanghai Veterinary Research Institute (Shanghai, China) by mating heterozygous p53^+/−^ mice originally obtained from the Jackson Laboratory (Bar Harbor, ME, USA). For viral infection, 8­ to 10-week-old p53WT and p53KO mice (*n* = 10/group) were intranasally inoculated with a sublethal dose (0.75 LD_50_/mouse) of PR8 virus. Mock-infection of mice (*n* = 10/group) was performed in an identical fashion to the viral infection using inoculums of phosphate-buffered saline (PBS). Clinical symptoms and body weight changes in PR8- and mock-infected mice were monitored daily for 16 days. Mice were euthanized upon a decrease in body weight >25 % from the initial weight. The 50 % egg infectious dose (EID_50_) in lung homogenates from PR8-infected mice was determined in 10-day-old embryonated chicken eggs and calculated using the method of Reed and Muench [17]. All animal experiments were performed in compliance with the Guidelines on the Humane Treatment of Laboratory Animals (Ministry of Science and Technology of the People’s Republic of China, Policy No. 2006 398) and were approved by the Institutional Animal Care and Use Committee at the Shanghai Veterinary Research Institute.

### Microarray analysis

Eight­ to 10-week-old p53WT and p53KO mice were randomly assigned to eight groups, three mice per group (Table 1). The mice were intranasally infected with a sublethal dose of PR8 virus or mock-infected with PBS. The PR8- and mock-infected mice were euthanized 3 and 6 days post-infection (dpi) to collect lung specimens. Half of the collected lung samples was used to determine viral titers and the other half was processed for microarray analyses. Gene expression profiling was performed using the Affymetrix GeneChip Mouse Genome 430 2.0 Array (Affymetrix, Santa Clara, CA, USA), which completely covers the Mouse Expression Set 430 and analyzes over 39,000 transcripts on a single array. Sample preparation and microarray experiments were performed according to the manufacturer’s protocols. The data generated from the microarray experiments were analyzed using the SBC Analysis System (http://www.ebioservice.com). Bonferroni correction was used for multiple comparisons between groups. The upregulation or downregulation of more than 2-fold when PR8- and mock-infected mice were compared, with Bonferroni-corrected *p* < 0.05, was considered significant.

**Table 1:** Mice groups assigned for microarray analysis

		Sampling 3 dpi	Sampling 6 dpi
PR8-infection	p53WT mice	3^a^	3
	p53KO mice	3	3
Mock-infection	p53WT mice	3	3
	p53KO mice	3	3

*dpi* days post-infection

^a^number of mice per group

### Quantitative real-time reverse transcription polymerase chain reaction (qRT-PCR)

Total RNA was extracted from lung tissue using the RNeasy Plus Mini Kit (Qiagen, Valencia, CA, USA) according to the manufacturer’s protocol. The complementary DNA (cDNA) was synthesized using avian myeloblastosis virus reverse transcriptase (TaKaRa, Otsu, Japan). qRT-PCR analysis was performed using SYBR Premix Ex *Taq*™ (TaKaRa) according to the manufacturer’s protocol. Briefly, total reaction volumes of 20 μl were prepared containing 1 μl of cDNA, 10 μl of SYBR Premix Ex Taq™ (2×), and 0.2 μM of specific primers. The amplification parameters were an initial denaturation step at 95 °C for 2 min followed by 40 cycles of 15 s at 95 °C and 60 s at 60 °C. The primer sequences are shown in Additional file 1: Table S1. Relative quantification of gene expression was calculated using the 2^-∆∆Ct^ method [18]. Data are presented as the fold change (FC) in gene expression normalized to endogenous glyceraldehyde-3-phosphate dehydrogenase (GAPDH) and relative to the mock-infected mice.

### Statistical analysis

All measured values are expressed as the mean ± standard error (SE). The significance of the results was analyzed using the Student’s two-tailed *t*-test or the Gehan-Breslow-Wilcoxon test. A *p* value < 0.05 was considered statistically significant.

## Results

### p53KO mice shows an increased susceptibility to IAV infection

**Fig. 1:**
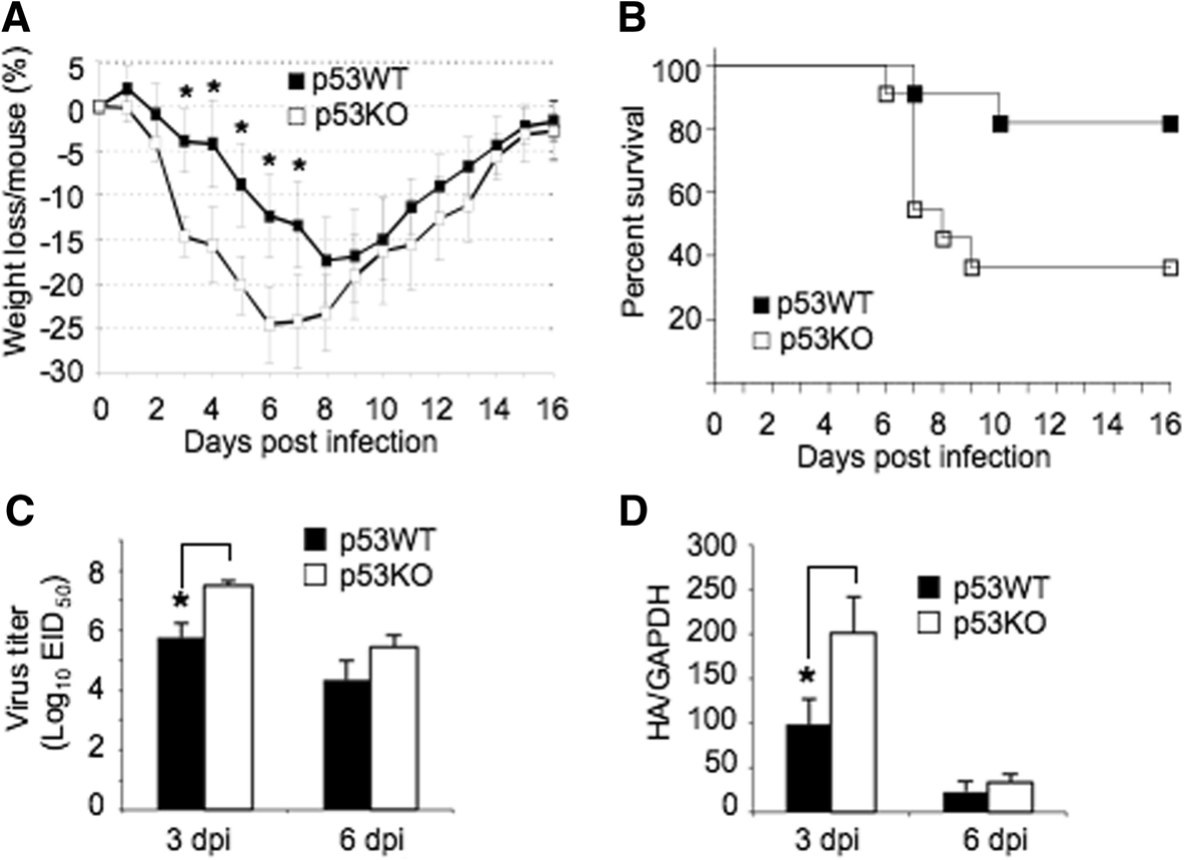
Increased susceptibility of p53KO mice to PR8 infection. p53WT and p53KO mice (*n* = 10 per group) were intranasally inoculated with a sublethal dose of PR8 virus. Clinical signs and weight loss were assessed daily for 16 days. Lungs of infected mice were collected 3 and 6 dpi for analysis of viral loads. **a** Weight loss analysis in PR8-infected mice. Results are percentages of mean weight loss relative to initial weight. **b** The survival rates of PR8-infected mice. **c** Viral loads were determined by serial titration of lung homogenates in 10-day-old embryonated SPF chicken eggs. The EID_50_ was calculated. **d** The expression of viral hemagglutinin (HA) in the lungs of infected mice was determined by qRT-PCR. Values are means ± SE of at least 4 mice. *, *p* < 0.05 as assessed by the Student’s *t*-test; dpi, days post-infection

The p53WT and p53KO mice were intranasally inoculated with a sublethal dose of PR8 virus and monitored for 16 days. Mock infection of mice was performed in an identical fashion to serve as controls. PR8-infected p53KO mice displayed clinical signs of influenza (reduced activity, tachypnea, labored respiration, ruffled fur, and increased weight loss) beginning 2 dpi, whereas PR8-infected p53WT mice showed clinical signs beginning 3 dpi. A significant difference in weight loss was observed between PR8-infected p53WT and p53KO mice 3–7 dpi (Fig. 1a). By 16 dpi, 63.6 % of PR8-infected p53KO mice died of viral infections (survival rate 36.4 %), whereas 18.2 % of PR8-infected p53WT mice died (survival rate 81.8 %) (*p* = 0.0222, as assessed by the Gehan-Breslow-Wilcoxon test) (Fig. 1b). Viral loads in the lungs of PR8-infected mice were analyzed 3 and 6 dpi by viral titration and qRT-PCR analysis. The virus titer in lung homogenates from PR8-infected p53KO mice was 10^7.5^ EID_50_/ml, which was significantly higher than the 10^5.8^ EID_50_/ml in the lungs of PR8-infected p53WT mice 3 dpi; however, no significant difference was found between PR8-infected p53WT and p53KO mice 6 dpi (Fig. 1c). These results were confirmed by qRT-PCR analyses that examined the abundance of viral hemagglutinin (HA) mRNA in lung homogenates of PR8-infected mice. As shown in Fig. 1d, HA expression was significantly higher in the lungs of PR8-infected p53KO mice compared to PR8-infected p53WT mice 3 dpi, but not 6 dpi. These observations indicated that p53-deficient mice had an increased susceptibility to IAV infection and that p53 played an antiviral role against IAV infection *in vivo*.

### Global analysis of immune-related gene expression between PR8-infected p53WT and p53KO mice

The p53 protein functions as a transcription factor that regulates expression of a series of immune-related genes in response to viral infection [10, 15, 16]. To explore the basis of the susceptibility differences to IAV infection between p53WT and p53KO mice, we compared expression profiles of immune-related genes in the lungs of PR8-infected p53WT and p53KO mice. To this end, we isolated RNA from lung homogenates of PR8- and mock-infected mice 3 and 6 dpi and performed microarray analysis. The genes that showed significantly changed expression levels between PR8-infected p53WT and p53KO mice are shown in Additional files 2: Table S2 and Additional file 3: Table S3. The possible functions of these significantly changed genes were analyzed by Gene Ontology (GO) analysis (Additional file 4: Figure S1).

A list of immune-related genes was obtained from the Immunology Database and Analysis Portal (ImmPort) System (https://immport.niaid.nih.gov), which contains 6005 gene entries (Additional file 5: Table S4). The number of immune-related genes that showed significantly changed expression levels in PR8-infected p53WT mice compared with their counterparts in mock-infected mice is shown in Table 2. The FC of significantly altered expressions of immune-related genes in PR8-infected p53WT mice was compared to that in PR8-infected p53KO mice. Among 275 (3 dpi) and 320 (6 dpi) upregulated immune-related genes in PR8-infected p53WT mice, 94 (3 dpi) and 193 (6 dpi) genes showed significantly attenuated expression in PR8-infected p53KO mice (Table 2, Additional file 6: Table S5). The possible functions of these significantly attenuated immune-related genes were analyzed by GO analysis. A considerable number (20 genes 3 dpi and 57 genes 6 dpi) of these genes belonged to the GO category “immune system process”, which were further classified into 11 sub-categories (Table 3).

**Table 2:** Number of significantly changed genes

	Regulation		p53WT^a^	p53WT vs p53KO^b^
3 dpi	Up	Total genes	964	508
		Immune-related genes	275	94
	Down	Total genes	1016	579
		Immune-related genes	236	135
6 dpi	Up	Total genes	987	639
		Immune-related genes	320	193
	Down	Total genes	1258	861
		Immune-related gens	279	184

^a^number of significantly changed genes in PR8-infected p53WT mice compared to mock-infected p53WT mice

^b^number of genes showing a significantly different expression between PR8-infected p53WT and p53KO mice

**Table 3:** GO analysis of genes with attenuated expression in PR8-infected p53KO mice

GO ID	Category	3 dpi		6 dpi	
		Number of genes	Representative genes	Number of genes	Representative genes
GO:0002684	Positive regulation of immune system process	5	*Ifng, Il2, Lag3, Stat6, Trat1*	18	*Blm, Lag3, Nfkbia, Il6, Fcer1g, Il15, Ifng,* etc.
GO:0002682	Regulation of immune system process	8	*Il2, Lag3, Orm1, Ifng, Trat1, Hmgb1, Jag1, Stat6*	24	*Blm, Orm1, Tnf, Nfkbia, Ifng, Tnfsf11,* etc.
GO:0050900	Leukocyte migration	2	*Ifng, Il16*	6	*Tnf, Ifng, Fcer1g, Il1b, Selp, Tlr2*
GO:0045321	Leukocyte activation	7	*Fyn, Ifng, Il2, Lag3, Stat6, Exo1, Mink1*	20	*Lag3, Blm, Fcgr2b, Was, Nfkbid,* etc.
GO:0019882	Antigen processing and presentation	2	*Ctse, Ifng*	5	*Ifng, Fcgr2b, Fcer1g, Slc11a1, Ctse*
GO:0001776	Leukocyte homeostasis	2	*Ifng, Il2*	6	*Ifng, Fcer1g, Gpam, Ikbkb, Il6, Pik3cd*
GO:0002252	Immune effecter process	6	*Ifng, Il2, Lag3, Stat6, Exo1, Irf7*	17	*Irf7, Tnf, Lag3, Fcgr2b, Fcer1g, Msh2,* etc.
GO:0002253	Activation of immune response	1	*Trat1*	8	*Nfkbia, Fcer1g, C1qb, Lat2, Plcg2,* etc.
GO:0002200	Somatic diversification of immune receptors	4	*Ifng, Il2, Stat6, Exo1*	5	*Ifng, Msh2, Cd40, Pms2, Exo1*
GO:0002520	Immune system development	10	*Hmgb1, Ifng, Il2, Jag1, Exo1, Tlx1, Six4*	23	*Blm, Timp1, Tnf, Tnfsf11, Cxcl13, Msh2,* etc.
GO:0006955	Immune response	13	*Ctla4, Cxcl1, Ifng, Mx2, Irf7, Polr3c,* etc.	47	*Irf7, Cxcl9, Cxcl10, Ccl2, Ccl7,* etc.

Out of 236 (3 dpi) and 279 (6 dpi) downregulated immune-related genes in PR8-infected p53WT mice, 135 (3 dpi) and 184 (6 dpi) genes showed significantly higher expression in PR8-infected p53KO mice than that in PR8-infected p53WT mice (Table 2, Additional file 7: Table S6). The GO analysis of the possible functions of these differentially regulated genes indicated that only 12 genes 3 dpi and 10 genes 6 dpi belonged to the GO category “immune system process” (Table 4), of which, most were broadly classified into GO categories “biological adhesion,” “growth,” “death,” “locomotion,” etc.

**Table 4:** GO analysis of genes significantly expressed in PR8-p53KO mice

GO ID	Category	3 dpi		6 dpi	
		Number of genes	Representative genes	Number of genes	Representative genes
GO:0002683	Negative regulation of immune system process	1	*Il20rb*	0	
GO:0002682	Regulation of immune system process	1	*Il20rb*	2	*Cd80, Il7*
GO:0031294	Lymphocyte costimulation	0		1	*Tgfb2*
GO:0050900	Leukocyte migration	1	*Ccl25*	0	
GO:0045321	Leukocyte activation	3	*Msh2, Ndrg1, Il20rb*	3	*Ms4a1, Cd80, Il7*
GO:0002684	Positive regulation of immune system process	0		2	*Cd80, Il7*
GO:0001776	Leukocyte homeostasis	0		1	*Tgfb2*
GO:0002252	Immune effector process	3	*Il20rb, Msh2, Ung*	0	
GO:0002200	Somatic diversification of immune receptors	2	*Msh2, Ung*	0	
GO:0002520	Immune system development	6	*Chuk, Nfkb2, Pbx1, Six1, Msh2, Ung*	7	*Cebpa, Il7, Mb, Mlf1, Med1, Six1, Tgfb2*
GO:0006955	Immune response	8	*Spon2, Bmi1, Il1rap, Msh2, Nfkb2, Ccl25, Il20rb, Ung*	2	*Il7, Polr3g*

### Impaired expression of immune-related genes involved in IFN signaling pathways in the absence of p53

**Fig. 2:**
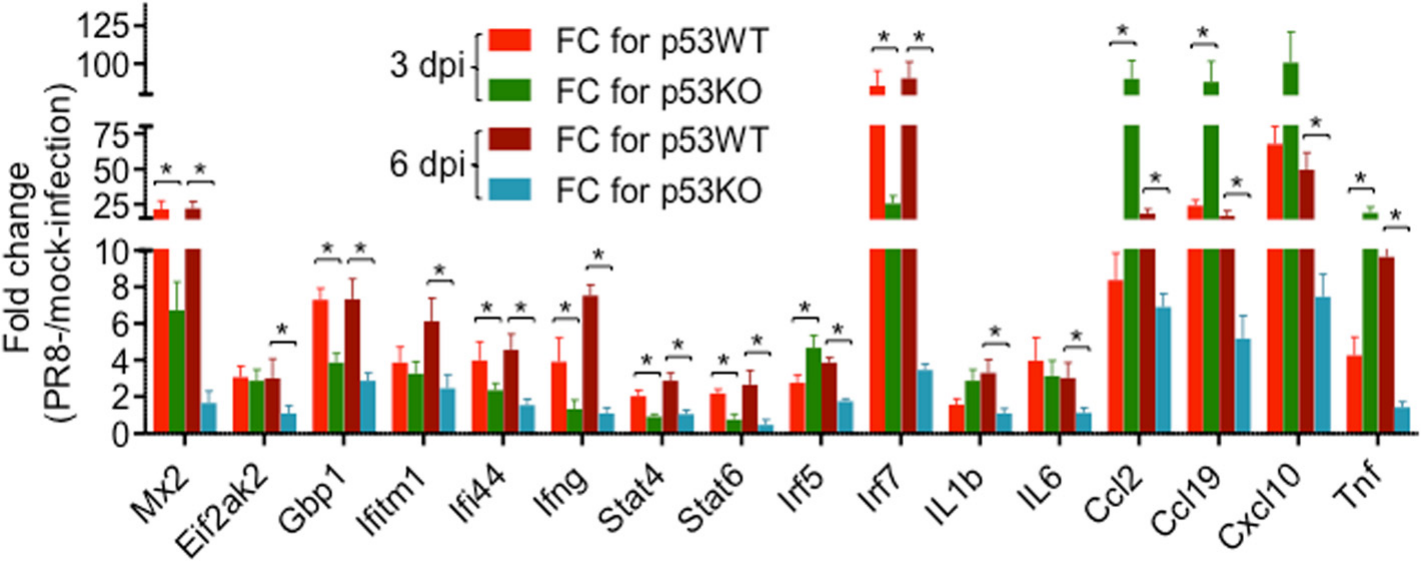
Detection of gene expression in PR8-infected mice by qRT-PCR. Lung samples were collected from PR8-infected mice 3 and 6 dpi and subjected to qRT-PCR for expression analysis of the indicated genes. FC, fold change. Results are means ± SE from 3 mice. *, *p* < 0.05 between PR8-infected p53WT and p53KO mice

The IFN signaling pathway plays a key role in regulation of immune response against IAV infection [19], therefore, we compared the expression of immune-related genes involved in IFN signaling between PR8-infected p53WT and 53KO mice. The products of IFN-stimulated genes (ISGs), such as Mx, PKR, OAS, and tetherin, play a major role in IFN-mediated antiviral responses against IAV infection [19]. First, we compared the differences in the expression of ISGs between PR8-infected p53WT and p53KO mice. Among 924 ISGs analyzed (list of ISGs are available upon request), 58 (3 dpi) and 66 (6 dpi) genes were significantly upregulated in PR8-infected p53WT mice compared to mock-infected mice, respectively (data are available upon request). Among the upregulated ISGs, 13 were expressed at a significantly attenuated level in PR8-infected p53KO mice compared to PR8-infected p53WT mice (Table 5). Remarkably, IFN-induced antiviral genes, including *Mx2*, *Oas2*, *Oas3*, and *Eif2ak2* (encoding protein kinase R (PKR), also known as eukaryotic translation initiation factor 2-alpha kinase 2 (EIF2AK2)), *Gbp1*, *Ifitm1*, and *Bst2* (encoding tetherin, also known as bone marrow stromal antigen 2), have been shown to possess anti-IAV effects [19–23] and are expressed at significantly attenuated levels in PR8-infected p53KO mice. Other IFN-induced antiviral genes, such as *Ifi44*, *Nampt*, *Rtp4*, *Trex1*, *and Daxx* [24, 25], have not yet been found to restrict IAV replication, but have significantly attenuated expression levels in PR8-infected p53KO mice. The difference in the expression of *Mx2*, *Eif2ak2*, *Gbp1*, *Ifitm1*, and *Ifi44* between PR8-infected p53WT and p53KO mice was confirmed by qRT-PCR (Fig. 2). Antiviral genes play key roles in the host immune response against IAV infection [19]. The impaired expression of antiviral genes in the absence of p53 during IAV infection was probably responsible for the high level of viral replication in the lungs of PR8-infected p53KO mice.

**Table 5:** The expression of selected genes involved in interferon signaling pathway

Gene symbol	Gene description	FC^a^ (p53WT/p53KO)	
		3 dpi	6 dpi
*Mx2*	myxovirus (influenza virus) resistance 2	42.94/14.54^b^	37.85/2.92^b^
*Oas2*	2'-5' oligoadenylate synthetase 2	14.75/12.43	13.20/3.06^b^
*Oas3*	2'-5' oligoadenylate synthetase 3	9.39/5.46	6.37/2.41^b^
*Eif2ak2*	eukaryotic translation initiation factor 2-alpha kinase 2 (PKR)	4.98/4.71	4.53/1.60^b^
*Gbp1*	guanylate binding protein 1	2.95/1.59	2.51/1.12^b^
*Ifitm1*	interferon induced transmembrane protein 1	2.50/2.32	2.58/1.24^b^
*Bst2*	bone marrow stromal cell antigen 2 (Tetherin)	3.41/2.22	2.96/1.02^b^
*Ifi44*	interferon-induced protein 44	4.59/2.82	2.98/1.09^b^
*Nampt*	nicotinamide phosphoribosyltransferase	2.59/2.52	3.62/1.78^b^
*Rtp4*	receptor transporter protein 4	7.16/7.68	9.56/3.91^b^
*Trex1*	three prime repair exonuclease 1	6.72/11.63	8.32/2.98^b^
*Daxx*	Fas death domain-associated protein	6.27/6.39	7.79/1.59^b^
*Ifng*	interferon gamma	2.87/1.05^b^	6.07/0.90^b^
*Ifnab*	interferon alpha B	1.80/0.80^b^	2.38/0.60^b^
*Stat4*	signal transducer and activator of transcription 4	1.78/0.79	2.08/0.80^b^
*Stat6*	signal transducer and activator of transcription 6	2.28/0.81^b^	1.87/0.34
*Irf5*	interferon regulatory factor 5	2.10/3.99	2.89/1.27^b^
*Irf7*	interferon regulatory factor 7	109.99/37.81^b^	93.18/7.18^b^

^a^ *FC* fold change

^b^significant difference in gene expression between PR8-infected p53WT and p53KO mice

The expression of ISGs is mainly regulated by IFN at the transcriptional level. The attenuated expression of antiviral ISGs in the absence of p53 may result from altered IFN expression. Next, we compared the expression of IFN and IFN receptor genes between PR8-infected p53WT and p53KO mice. Among IFN and IFN receptor genes analyzed, only *Ifng* and *Ifnab* were found to show a significantly attenuated expression in PR8-infected p53KO mice (Table 5). In addition, the Jak-Stat signaling pathway, which is activated by IFN, plays an essential role in the expression and activation of ISGs [26]. In the absence of p53, the expression levels of *Stat4* and *Stat6* were attenuated significantly (Table 5). The difference in the expression levels of *Ifng*, *Stat4*, and *Stat6* between PR8-infected p53WT and p53KO mice were confirmed by qRT-PCR (Fig. 2).

The IFN regulatory factors (Irf) are essential for expression and regulation of IFN and ISGs [27]. Among 9 Irf genes analyzed, *Irf1*, *Irf5*, *Irf7*, and *Irf9* were significantly upregulated in PR8-infected p53WT mice compared to mock-infected mice (data are available upon request), especially, *Irf7*, which is the master regulator of type I IFN-dependent immune responses [28] and significantly upregulated in IAV-infected mice [29] with a FC > 93 (Table 5). However, in PR8-infected p53KO mice, although *Irf7* was expressed at a significant level compared to mock-infected p53KO mice, the FC was remarkably less than that in PR8-infected p53WT mice, showing significantly attenuated expression. Significantly attenuated expression of *Irf5* was also found 6 dpi in the absence of p53 (Table 5). The difference in the expression levels of *Irf5* and *Irf7* between PR8-infected p53WT and p53KO mice was confirmed by qRT-PCR (Fig. 2).

Taken together, a number of genes essential for regulating IFN-mediated immune responses against viral infection were expressed at significantly attenuated levels in the absence of p53 during IAV infection, suggesting that the IFN-mediated immune response against IAV infection was impaired in the absence of p53.

### Dysregulated expression of cytokine and chemokine genes in the absence of p53

Viral infection results in the release of cytokines and chemokines designed to recruit and shape innate and adaptive immune responses. Unbalanced cytokine and chemokine responses lead to uncontrolled inflammation and unfavorable disease outcomes. In response to IAV infection, a number of cytokines and chemokines are significantly changed at the expression levels and play important roles in immune responses against IAV infection as well as in IAV pathogenesis [15, 29–31]. Therefore, we analyzed difference in expression levels of cytokines and chemokines between PR8-infected p53WT and 53KO mice. The cytokines, chemokines, and their respective receptors that were expressed with significant differences between PR8-infected p53WT and 53KO mice are shown in Table 6.

**Table 6:** The expression of selected cytokine and chemokine genes

Gene symbol	Gene description	FC^a^ (p53WT/p53KO)	
		3 dpi	6 dpi
*Il1b*	interleukin 1 beta	2.30/3.71	2.85/0.93^b^
*Il6*	interleukin 6	3.27/2.79	2.52/0.95^b^
*Il7*	interleukin 7	0.65/0.47	0.34/0.83^b^
*Il15*	interleukin 15	2.64/1.72	2.05/0.67^b^
*Il16*	interleukin 16	4.93/0.15^b^	1.20/0.99
*Il3ra*	interleukin 3 receptor, alpha chain	1.40/0.96	2.09/0.73^b^
*Il10ra*	Interleukin 10 receptor, alpha (Il10ra), mRNA	3.04/3.16	4.14/1.21^b^
*Il17rd*	interleukin 17 receptor D	0.48/0.41	0.07/0.45^b^
*Il17re*	interleukin 17 receptor E	0.60/0.81	0.45/1.55^b^
*Il20rb*	interleukin 20 receptor beta	0.27/0.98^b^	0.73/1.33
*Ccl2*	chemokine (C-C motif) ligand 2 (MCP-1)	11.83/130.82^b^	22.32/9.89^b^
*Ccl3*	chemokine (C-C motif) ligand 3 (MIP-1α)	7.90/11.23	9.62/1.90^b^
*Ccl4*	chemokine (C-C motif) ligand 4 (MIP-1β)	18.00/15.62	16.91/2.03^b^
*Ccl7*	chemokine (C-C motif) ligand 7	42.24/79.44	52.60/8.94^b^
*Ccl11*	chemokine (C-C motif) ligand 11	1.15/3.31	8.15/3.12^b^
*Ccl19*	chemokine (C-C motif) ligand 19	2.55/4.33	3.68/1.83^b^
*Ccl25*	chemokine (C-C motif) ligand 25	0.27/1.09^b^	0.58/1.53
*Cxcl1*	chemokine (C-X-C motif) ligand 1	21.07/6.71^b^	8.63/0.30^b^
*Cxcl9*	chemokine (C-X-C motif) ligand 9	39.58/120.08^b^	40.63/17.95^b^
*Cxcl10*	chemokine (C-X-C motif) ligand 10 (IP-10)	161.21/220.07	114.77/19.91^b^
*Cxcl13*	chemokine (C-X-C motif) ligand 13	2.48/10.71	7.60/1.71^b^
*Cxcl14*	chemokine (C-X-C motif) ligand 14	1.38/1.93	2.10/0.96^b^
*Ccr6*	chemokine (C-C motif) receptor 6	0.56/0.47	0.28/1.04^b^
*Ccrl2*	chemokine (C-C motif) receptor-like 2	2.89/2.26	2.35/0.82^b^
*Tnf*	tumor necrosis factor	9.43/34.25^b^	8.90/1.06^b^
*Tnfrsf10b*	tumor necrosis factor receptor superfamily, member 10b	0.46/2.18^b^	1.26/1.80
*Tnfsf11*	tumor necrosis factor (ligand) superfamily, member 11	0.27/0.08	5.88/1.81^b^
*Tnfrsf8*	tumor necrosis factor receptor superfamily, member 8	5.86/1.08^b^	0.88/0.01^b^
*Tnfrsf18*	tumor necrosis factor receptor superfamily, member 18	2.55/2.13	3.86/1.43^b^

^a^ *FC* fold change

^b^significant difference in gene expression between PR8-infected p53WT and p53KO mice

Of the 35 interleukin and respective receptor genes analyzed, interleukin genes, such as *IL1b*, *IL6*, *IL15*, and *IL16*, showed significantly attenuated expression levels 6 dpi, whereas most were expressed at relatively attenuated levels 3 dpi in PR8-infected p53KO mice compared with PR8-infected p53WT mice (Table 6). Analysis of the expression levels of chemokines and their respective receptor genes indicated that many were significantly changed between PR8-infected p53WT and p53KO mice. Notably, *Ccl2* (encoding MCP-1), *Cxcl9*, and *Tnf* showed remarkably higher expression levels 3 dpi and significantly attenuated expression levels 6 dpi in PR8-infected p53KO mice compared to PR8-infected p53WT mice (Table 6). For instance, *Ccl2*, which is significantly expressed in H5N1-infected primary human cells and in IAV-infected highly susceptible mice [29, 32], showed an upregulated expression 3 dpi in PR8-infected p53KO mice with a FC of 130.8, which was 11-fold higher than that (FC = 11.8) in PR8-infected p53WT mice, whereas it displayed attenuated expression 6 dpi in PR8-infected p53KO mice with a FC of 9.89, which was 2.3-fold lower than that (FC = 22.32) in PR8-infected p53WT mice. Similar expression patterns were also observed for *Ccl3*, *Ccl7*, *Ccl11*, *Ccl19*, *Cxcl10, Cxcl13* and *Cxcl14* (Table 6). The differences in expression levels of *IL1b*, *IL6*, *Ccl2*, *Ccl19*, *Cxcl10*, and *Tnf* between PR8-infected p53WT and p53KO mice were confirmed by qRT-PCR (Fig. 2). These observations suggested dysregulated expression of cytokines and chemokines in the absence of p53 during IAV infection.

## Discussion

Tumor suppressor p53 is ubiquitously expressed in cells and plays an important role in host defense against tumor development. A growing body of evidence has indicated that p53 is involved in regulation of immune responses against viral infections [7–10]. In this study, we observed that p53-deficient mice infected with PR8 virus showed increased mortality, severe weight loss, and higher viral loads in the infected lungs compared to PR8-infected p53WT mice (Fig. 1), suggesting that p53 was involved in host defense mechanisms against IAV infection. These observations were in good agreement with a previous description that p53 serves as a host antiviral factor against IAV infection [15].

The major mechanism by which p53 functions is as a transcription factor that regulates, both positively and negatively, the expression of a large and disparate group of responsive genes [6]. We comparatively analyzed the global expression profiles of immune-related genes between IAV-infected p53WT and p53KO mice, which could gain an insight into the basis of susceptibility differences to IAV infection between p53WT and p53KO mice. We observed that a number of immune-related genes showed a significant change in expression levels between PR8-infected p53WT and p53KO mice (Table 2). Notably, a considerable number of genes that showed significantly attenuated expression in PR8-infected p53KO mice compared with PR8-infected p53WT mice belonged to the GO category “immune system process” (Tables 2 and 3). These data indicated that the expression of a range of immune-related genes was impaired in the absence of p53 during IAV infection.

The IFN signaling pathway and especially, IFN-induced antiviral genes, plays a key role in regulating the immune response against IAV infection [19]. In this study, we found that several anti-IAV genes, including *Mx2*, *Oas2*, *Oas3*, *Eif2ak2* (encoding PKR), *Gbp1*, *Ifitm1*, and *Bst2* (encoding tetherin) [19–23] and other antiviral genes, including *Ifi44, Nampt*, *Rtp4*, *Trex1*, and *Daxx* [24, 25] were expressed at significantly attenuated levels in PR8-infected p53KO mice compared to PR8-infected p53WT mice (Table 5). We thought that this impaired expression of antiviral genes in the absence of p53 during IAV infection was responsible for the high level of viral replication in the lungs of PR8-infected p53KO mice. In addition, the expression of several genes, such as *Irf7*, *Ifng*, *Stat4*, and *Stat6*, which play important roles in IFN-mediated immune response, was detected at significantly attenuated levels in PR8-infected p53KO mice (Table 5), suggesting that the IFN-mediated immune response against IAV infection was impaired in the absence of p53.

During IAV infection, unbalanced cytokine and chemokine responses lead to uncontrolled inflammation and unfavorable disease outcomes [31]. A comparison in cytokine and chemokine expression levels between PR8-infected p53WT and 53KO mice showed that several were significantly different (Table 6), suggesting a dysregulated cytokine and chemokine response in the absence of p53 during IAV infection. It is known that the upregulated expression of proinflammatory cytokines and chemokines, including *Ccl2* (encoding MCP-1), *Ccl3* (encoding MIP-1α), *Ccl4* (encoding MIP-1β), *Cxcl10* (encoding IP-10), and *Tnf,* was observed during IAV infection and thought to be associated with unfavorable disease outcomes [31], such as the significant expression of *Ccl2* in H5N1-infected cells and in IAV-infected highly susceptible mice [29, 32]. We observed that *Ccl2* was upregulated 3 dpi in PR8-infected p53KO mice with a FC of 130.8, which was 11-fold higher than that in PR8-infected p53WT mice (FC = 11.8) (Table 6). The p53 protein is a suppressor of inflammation [33]. The upregulated expression of proinflammatory cytokine and chemokine genes, such as *Ccl2*, *Ccl3*, *Cxcl9, Cxcl10*, and *Tnf*, suggested aberrant inflammation conditions in PR8-infected p53KO mice during early IAV infection and that the absence of p53 was responsible for the upregulated expression.

In addition to inducing inflammatory responses, cytokines and chemokines are immunological messengers that play important roles in the development of innate and adaptive immunity against IAV infection [31, 34]. For example, IFN-γ, the most important cytokine in cell-mediated immunity, mediates expression of major histocompatibility complex classes I and II and stimulates antigen presentation and cytokine production [34]. IFN-γ treatment at early stages of IAV infection protects mice from death [35]. IL1b and IL6 play crucial roles in the regulation of immune responses against IAV infection. *IL-1b*-deficient mice infected with IAV exhibited greater mortalities than wild-type mice [36]. IL-6 is involved in the development of influenza-specific memory CD4 T cells [37]. Cxcl10 is a potent chemoattractant for activated Th1 lymphocytes and natural killer cells and is thought to play a role in the temporal development of innate and adaptive immunity in concert with type I and II IFNs [38]. We observed that a range of cytokines and chemokines, such as *Ifng*, *IL-1b*, *Il6*, *Ccl2*, and *Cxcl10*, showed significantly attenuated expression 6 dpi in PR8-infected p53KO mice compared to PR8-infected p53WT mice (Tables 5 and 6). The rapid decline in the expression of cytokine and chemokine genes in the absence of p53 might be associated with an impairment of innate and adaptive immunity against IAV infection.

## Conclusions

Lack of p53 resulted in an increased susceptibility of mice to IAV infection, which was associated with significantly altered expression of a range of immune-related genes in IAV-infected p53-deficient mice. The significantly attenuated expression of *Ifng*, *Irf7*, and antiviral genes, such as *Mx2* and *Eif2ak2*, suggested an impaired IFN-mediated immune response against IAV infection in the absence of p53. On the other hand, dysregulated expression of cytokines and chemokines, such as *Ccl2*, *Cxcl9*, *Cxcl10*, and *Tnf*, has been observed, reflecting aberrant inflammation conditions in p53-deficient mice during early IAV infection. The impaired IFN-mediated antiviral response and the aberrant inflammatory response in the absence of p53 suggested an essential role of p53 in the regulation of antiviral and inflammatory responses during IAV infection.

## Additional files

Additional file 1: Table S1. Sequence of primer used. (DOC 50 kb) Additional file 2: Table S2. List of genes showing a significantly attenuated expression 3 and 6 dpi in p53KO mice as compared to p53WT mice. (XLS 254 kb) Additional file 3: Table S3. List of genes expressed 3 and 6 dpi in p53KO mice with an expression significantly higher than in p53 WT mice. (XLS 324 kb) Additional file 4:
**GO analysis for biological process.** (PPT 403 kb) Additional file 5: Table S4. List of immune-related genes. (PDF 661 kb) Additional file 6: Table S5. List of immune-related genes showing a significantly attenuated expression 3 and 6 dpi in p53KO mice as compared to p53WT mice. (XLS 104 kb) Additional file 7: Table S6. List of immune-related genes expressed 3 and 6 dpi in p53KO mice with an expression significantly higher than in p53 WT mice. (XLS 108 kb)
