# Supplementary material for: Transcriptional analysis of immune-related gene expression in p53-deficient mice with increased susceptibility to influenza A virus infection
Source: BMC Med Genomics. 2015 Aug 18;8:52. doi: 10.1186/s12920-015-0127-8 (PMC4539693; doi:10.1186/s12920-015-0127-8)
Supplement: Additional file 1: Table S1. — Sequence of primer used. (DOC 50 kb) [file 12920_2015_127_MOESM1_ESM.doc]

**Table S1 Sequence of primer used**

| **Gene name** |  | **Primer sequence (5’ to 3’)** |
| --- | --- | --- |
| *IL1b* | Forward primer | TCGTGCTGTCGGACCCATAT |
|  | Reverse primer | GTCGTTGCTTGGTTCTCCTTGT |
| *IL6* | Forward primer | CTTCTTGGGACTGATG |
|  | Reverse primer | CTCATTTCCACGATTT |
| *Ccl3* | Forward primer | CCAAGTCTTCTCAGCGCCAT |
|  | Reverse primer | TCCGGCTGTAGGAGAAGCAG |
| *Ccl19* | Forward primer | ATGTGAATCACTCTGGCCCAGGAA |
|  | Reverse primer | AAGCGGCTTTATTGGAAGCTCTGC |
| *Cxcl10* | Forward primer | GGATGGCTGTCCTAGCTCTG |
|  | Reverse primer | TGAGCTAGGGAGGACAAGGA |
| *Tnf* | Forward primer | GACCCTCACACTCAGATCATCTTCT |
|  | Reverse primer | CCTCCACTTGGTGGTTTGCT |
| *Irf5* | Forward primer | CAACGCACCCTATTCC |
|  | Reverse primer | CAGCAGGTCAGGCAAG |
| *Irf7* | Forward primer | CACCCCCATCTTCGACTTCA |
|  | Reverse primer | CCAAAACCCAGGTAGATGGTGTA |
| *Ifng* | Forward primer | CGGCACAGTCATTGAAAGCCTA |
|  | Reverse primer | GTTGCTGATGGCCTGATTGTC |
| *Stat4* | Forward primer | ACGCAGATAGTGAACG |
|  | Reverse primer | GCCAGTAGGGTAAAGC |
| *Stat6* | Forward primer | AAGAAACCCAAAGATGAGG |
|  | Reverse primer | GGATGGACTGTGGAGGATA |
| *Mx2* | Forward primer | CCTGCCTGCCATCGCTGTC |
|  | Reverse primer | GCCTCTCCACTCCTCTCCCTCATT |
| *Eif2ak2* | Forward primer | GGAAAATCCCGAACAAGGAG |
|  | Reverse primer | CCCAAAGCAAAGATGTCCAC |
| *Gbp1* | Forward primer | CCACTGTGCAGTCTCACACAAA |
|  | Reverse primer | GTTCTGGCTTCTCGGGATGA |
| *Ifitm1* | Forward primer | GCCTAAGGAGCAGCAAGA |
|  | Reverse primer | CCAGTCGTATCACCCACC |
| *Ifi44* | Forward primer | AGACTTGATAAAACATGGCATTCTGC |
|  | Reverse primer | CATGGAATGCCTCCAGCTTGG |
| *HA* | Forward primer | TGAACTATTACTGGACCTTGCT |
|  | Reverse primer | CTCCTATTGTGACTGGGTGTAT |
| *GAPDH* | Forward primer | CGGGAAGCTTGTGATCAATGG |
|  | Reverse primer | GGCAGTGATGGCATGGACTG |
