# Supplementary material for: Transcriptional analysis of immune-related gene expression in p53-deficient mice with increased susceptibility to influenza A virus infection
Source: BMC Med Genomics. 2015 Aug 18;8:52. doi: 10.1186/s12920-015-0127-8 (PMC4539693; doi:10.1186/s12920-015-0127-8)
Supplement: Additional file 4: — GO analysis for biological process. (PPT 403 kb) [file 12920_2015_127_MOESM4_ESM.ppt]

## Slide 1
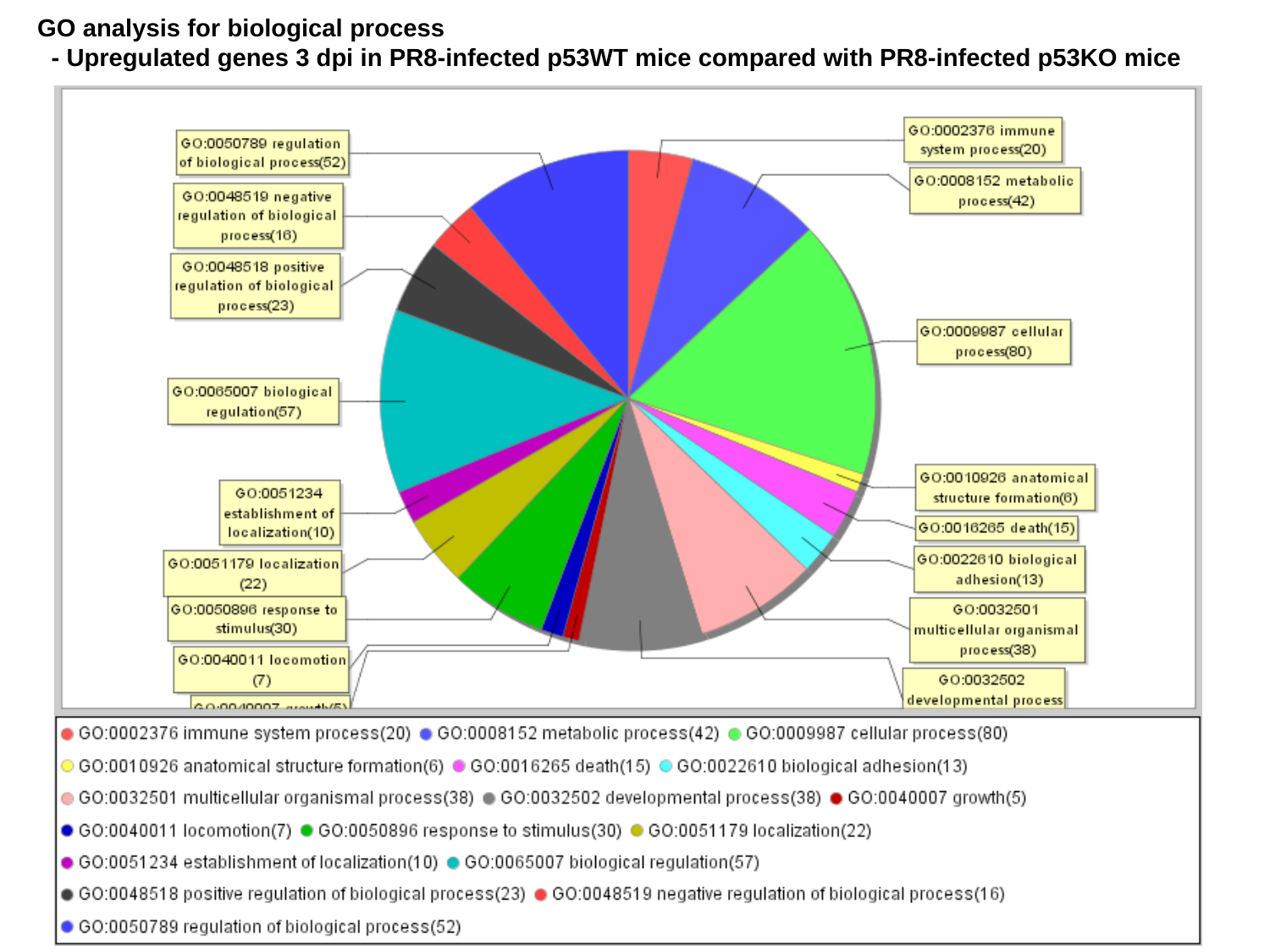

GO analysis for biological process
 - Upregulated genes 3 dpi in PR8-infected p53WT mice compared with PR8-infected p53KO mice

## Slide 2
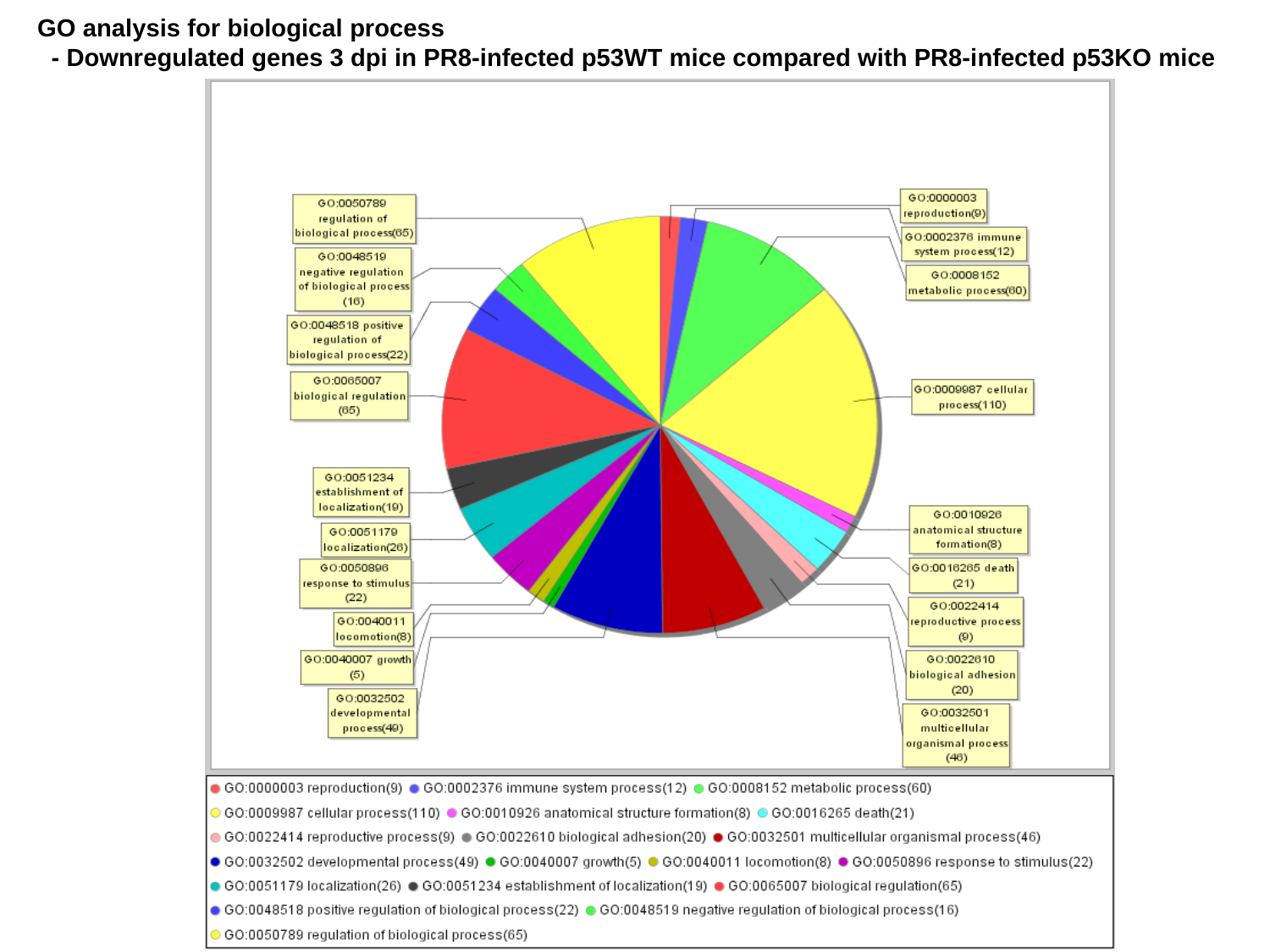

GO analysis for biological process
 - Downregulated genes 3 dpi in PR8-infected p53WT mice compared with PR8-infected p53KO mice

## Slide 3
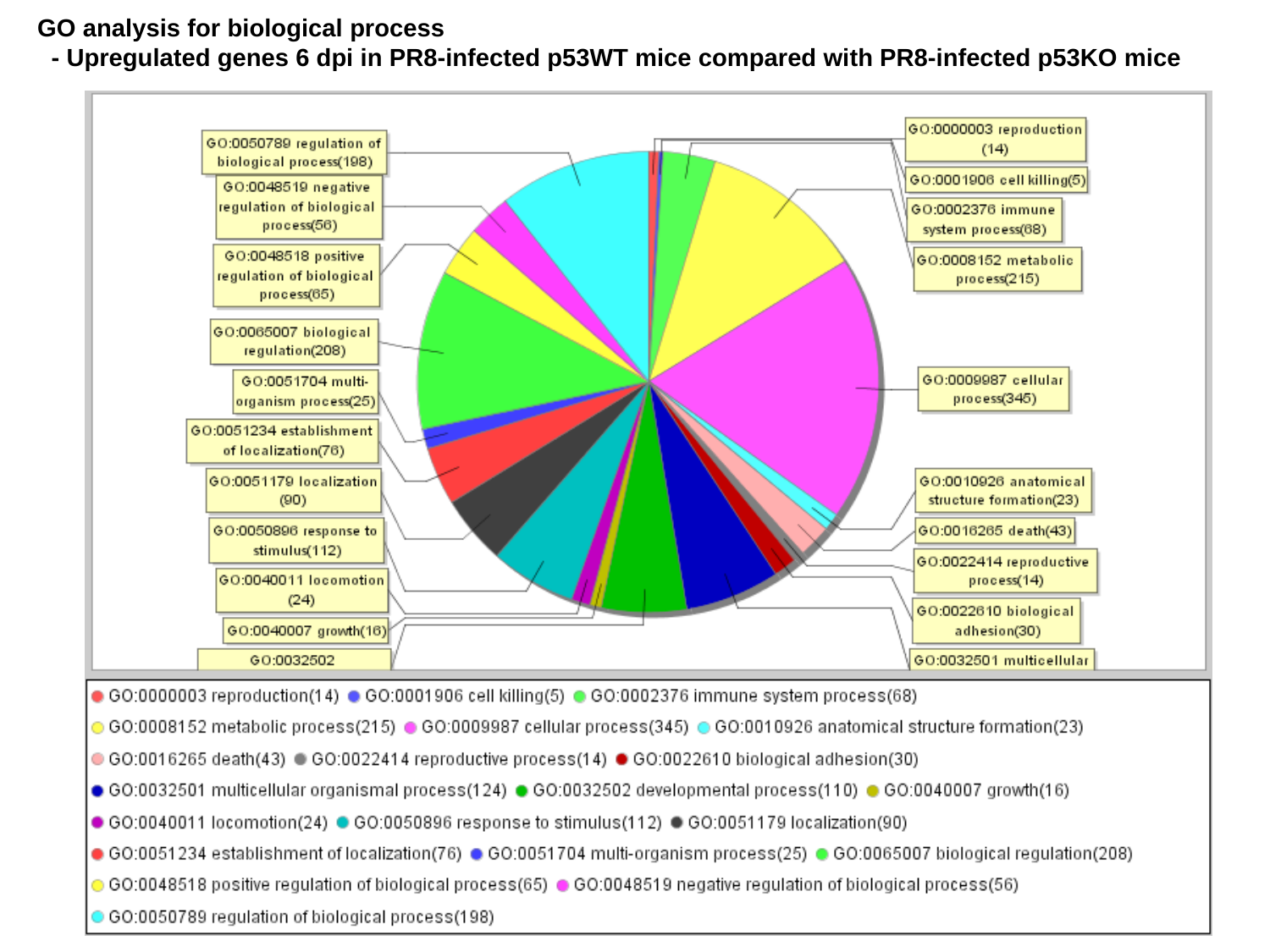

GO analysis for biological process
 - Upregulated genes 6 dpi in PR8-infected p53WT mice compared with PR8-infected p53KO mice

## Slide 4
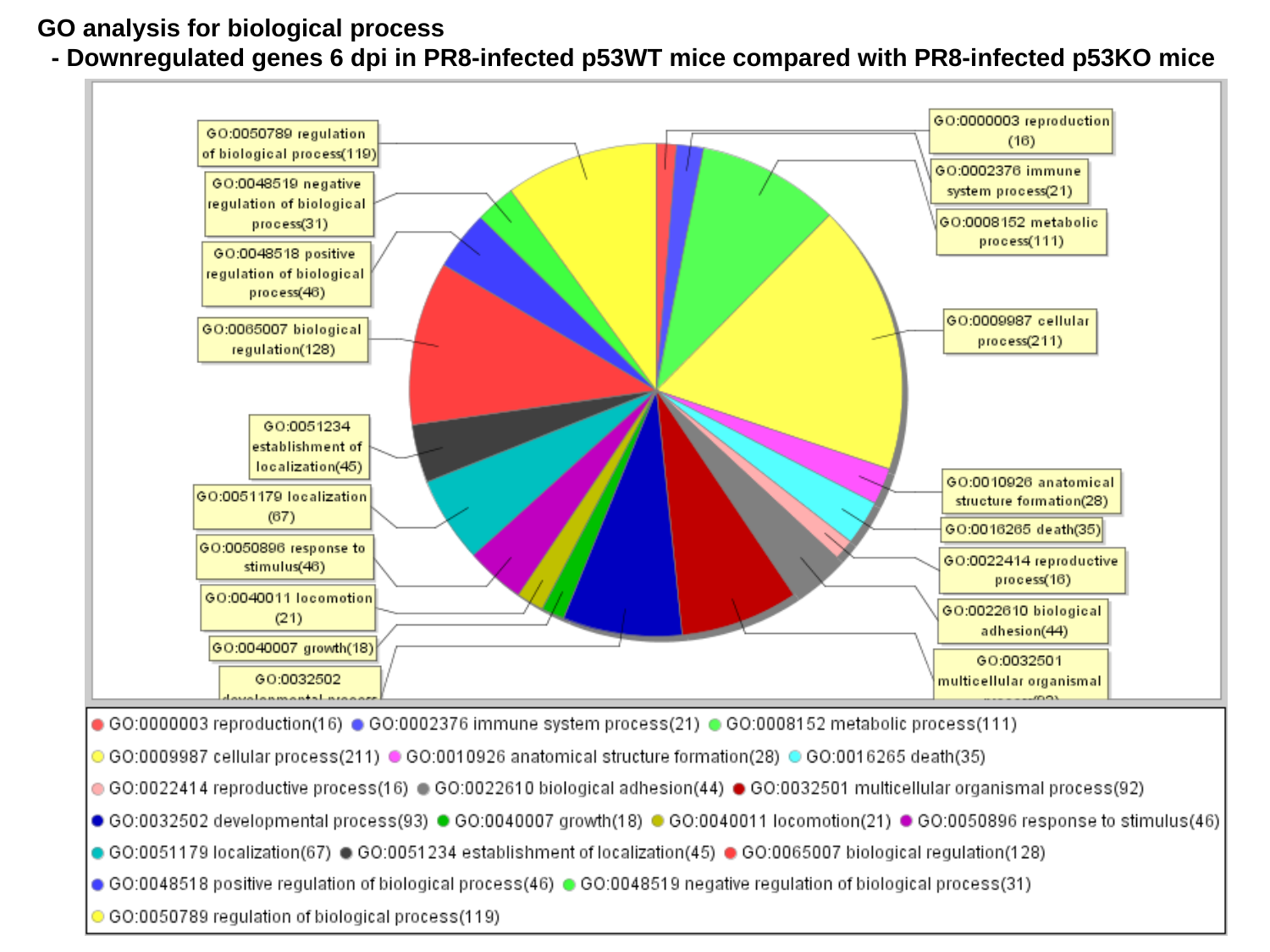

GO analysis for biological process
 - Downregulated genes 6 dpi in PR8-infected p53WT mice compared with PR8-infected p53KO mice
