# Supplementary material for: Transcriptional analysis of immune-related gene expression in p53-deficient mice with increased susceptibility to influenza A virus infection
Source: BMC Med Genomics. 2015 Aug 18;8:52. doi: 10.1186/s12920-015-0127-8 (PMC4539693; doi:10.1186/s12920-015-0127-8)
Supplement: Additional file 5: Table S4. — List of immune-related genes. (PDF 661 kb) [file 12920_2015_127_MOESM5_ESM.pdf]

Table S4. List of immune-related genes

ENTREZ_GENE_ID	GENE_SYMBOL	GENE_NAME
100	ADA	adenosine deaminase
1000	CDH2	cadherin 2, type 1, N-cadherin (neuronal)
100049587	SIGLEC14	sialic acid binding Ig-like lectin 14
1001	CDH3	cadherin 3, type 1, P-cadherin (placental)
100101148	LOC100101148	FK506 binding protein 6, 36kDa pseudogene
100101246	LOC100101246	interferon induced transmembrane protein pseudogene
100101247	LOC100101247	interferon induced transmembrane protein pseudogene
100101266	LOC100101266	hepatitis A virus cellular receptor 1 pseudogene
100101629	GAGE8	G antigen 8
10011	SRA1	steroid receptor RNA activator 1
100124401	LOC100124401	cytokine receptor-like factor 3 pseudogene
100126791	EGOT	eosinophil granule ontogeny transcript (non-protein coding)
100127173	HLA-Y	major histocompatibility complex, class I, Y (pseudogene)
100127945	LOC100127945	suppressor of cytokine signaling 5 pseudogene
100128009	LOC100128009	similar to hCG1642538
100128043	LOC100128043	Fas associated factor family member 2 pseudogene
100128135	GVINP2	GTPase, very large interferon inducible pseudogene 2
100128174	LOC100128174	beta-defensin 131-like
100128218	PSMC6P3	proteasome 26S subunit, ATPase, 6 pseudogene 3
100128299	LOC100128299	suppressor of cytokine signaling 5 pseudogene
100128356	LOC100128356	protein transactivated by hepatitis B virus E antigen
100128365	LOC100128365	interferon stimulated exonuclease gene 20kDa-like 2 pseudogene
100128413	LOC100128413	X-linked inhibitor of apoptosis pseudogene
100128449	LOC100128449	interleukin-9 receptor-like
100128465	IFIT6P	interferon-induced protein with tetratricopeptide repeats 6, pseudogene
100128686	LOC100128686	programmed cell death 10 pseudogene
100128733	LOC100128733	FCF1 small subunit (SSU) processome component homolog (S. cerevisiae) pseudogene
100128764	PRKRIRP6	protein-kinase, interferon-inducible double stranded RNA dependent inhibitor, repressor of (P58 repressor) pseudogene 6
100129032	GLYATL1P1	glycine-N-acyltransferase-like 1 pseudogene 1
100129074	PRKRIRP5	protein-kinase, interferon-inducible double stranded RNA dependent inhibitor, repressor of (P58 repressor) pseudogene 5
100129107	LOC100129107	transcription factor 3 (E2A immunoglobulin enhancer binding factors E12/E47) pseudogene
100129137	LOC100129137	leukocyte surface antigen CD53-like

100129138	LOC100129138	THAP domain containing, apoptosis associated protein 3 pseudogene
100129192	MICC	MHC class I polypeptide-related sequence C (pseudogene)
100129216	LOC100129216	beta-defensin 131-like
100129382	PRKRIRP4	protein-kinase, interferon-inducible double stranded RNA dependent inhibitor, repressor of (P58 repressor) pseudogene 4
100129404	LOC100129404	matrix metallopeptidase 12 (macrophage elastase) pseudogene
100129575	MR1P1	major histocompatibility complex, class I-related pseudogene
100129631	IGHV2OR16-5	immunoglobulin heavy variable 2/OR16-5 (non-functional)
100129641	BTG1P1	B-cell translocation gene 1 pseudogene 1
100129646	LOC100129646	intraflagellar transport 57 homolog (Chlamydomonas) pseudogene
100129725	LOC100129725	PERP, TP53 apoptosis effector pseudogene
100129933	GLYATL1P2	glycine-N-acyltransferase-like 1 pseudogene 2
10013	HDAC6	histone deacetylase 6
100130018	LOC100130018	prostaglandin reductase 1 pseudogene
100130137	LOC100130137	integrin beta 1 binding protein 1 pseudogene
100130180	LOC100130180	nuclear factor, erythroid-derived 2, like 3 pseudogene
100130485	LOC100130485	FK506 binding protein 3, 25kDa pseudogene
100130671	IFNWP9	interferon, omega 1 pseudogene 9
100130674	LOC100130674	cathepsin B pseudogene
100130731	LOC100130731	proteasome (prosome, macropain) subunit, beta type, 1 pseudogene
100130758	METTL15P1	methyltransferase like 15 pseudogene 1
100130866	IFNWP15	interferon, omega 1 pseudogene 15
100130901	LOC100130901	leukocyte immunoglobulin-like receptor subfamily B member 5-like
100130923	LOC100130923	proteasome (prosome, macropain) subunit, alpha type, 2 pseudogene
100131041	LOC100131041	FK506 binding protein pseudogene
100131159	LOC100131159	PERP, TP53 apoptosis effector pseudogene
100131234	LOC100131234	familial acute myelogenous leukemia related factor
100131280	LOC100131280	integrin alpha FG-GAP repeat containing 2 pseudogene
100131284	LOC100131284	proteasome (prosome, macropain) 26S subunit, non-ATPase, 14 pseudogene
100131309	PRKRIRP9	protein-kinase, interferon-inducible double stranded RNA dependent inhibitor, repressor of (P58 repressor) pseudogene 9
100131439	CD300LD	CD300 molecule-like family member d
100131451	TLR12P	toll-like receptor 12, pseudogene
100131454	DBIL5P	diazepam binding inhibitor-like 5, pseudogene
100131553	LOC100131553	disintegrin and metalloproteinase domain-containing protein 21-like
100131557	LOC100131557	programmed cell death 2-like pseudogene
100131593	LOC100131593	cytokine induced protein 29 kDa pseudogene

100131616	LOC100131616	caspase 3, apoptosis-related cysteine peptidase pseudogene
100131775	LOC100131775	proteasome (prosome, macropain) subunit, alpha type, 2 pseudogene
100131810	ADH5P3	alcohol dehydrogenase 5 (class III), chi polypeptide, pseudogene 3
100131859	LOC100131859	cytokine induced protein 29 kDa pseudogene
100132027	LOC100132027	putative apoptosis-related protein 2-like
100132108	LOC100132108	proteasome (prosome, macropain) 26S subunit, non-ATPase, 7 pseudogene
100132166	LOC100132166	interleukin-9 receptor-like
100132268	LOC100132268	FK506 binding protein 4, 59kDa pseudogene
100132285	KIR2DS2	killer cell immunoglobulin-like receptor, two domains, short cytoplasmic tail, 2
100132310	LOC100132310	FCF1 small subunit (SSU) processome component homolog (S. cerevisiae) pseudogene
100132415	LOC100132415	suppressor of cytokine signaling 5-like
100132417	FCGR1C	Fc fragment of IgG, high affinity Ic, receptor (CD64)
100132537	LOC100132537	dedicator of cytokinesis protein 11-like
100132609	LOC100132609	programmed cell death 2 pseudogene
100132656	LOC100132656	FK506 binding protein 4, 59kDa pseudogene
100132705	LOC100132705	immunoglobulin superfamily member 3-like
100132900	LOC100132900	immunoglobulin superfamily member 3-like
100133046	KIR3DL3	killer cell immunoglobulin-like receptor, three domains, long cytoplasmic tail, 3
100133128	LOC100133128	Beta-defensin 108B-like
100133189	CTSL1P7	cathepsin L1 pseudogene 7
100133267	LOC100133267	defensin, beta 130-like
100133300	LOC100133300	FK506 binding protein 4, 59kDa pseudogene
100133302	LOC100133302	FK506 binding protein 4, 59kDa pseudogene
100133511	LOC100133511	complement C3-like
100133583	LOC100133583	HLA class II histocompatibility antigen, DQ beta 1 chain-like
100133661	LOC100133661	HLA class II histocompatibility antigen, DR beta 4 chain-like
100133678	LOC100133678	HLA class II histocompatibility antigen, DQ alpha 1 chain-like
100133763	LOC100133763	HLA class II histocompatibility antigen, DQ alpha 1 chain-like
100133810	LOC100133810	lymphocyte antigen 75-like
100133811	LOC100133811	HLA class II histocompatibility antigen, DRB1-4 beta chain-like
100133941	CD24	CD24 molecule
100133944	LOC100133944	IgGFc-binding protein-like
100134397	LOC100134397	putative V-set and immunoglobulin domain-containing protein 6-like
10014	HDAC5	histone deacetylase 5
100144748	KLLN	killin, p53-regulated DNA replication inhibitor
10015	PDCD6IP	programmed cell death 6 interacting protein
10016	PDCD6	programmed cell death 6

100169989	DBIL5P2	diazepam binding inhibitor-like 5 pseudogene 2
10017	BCL2L10	BCL2-like 10 (apoptosis facilitator)
10018	BCL2L11	BCL2-like 11 (apoptosis facilitator)
100187761	AN	blood group Ahonen
100188278	FECD2	corneal dystrophy, Fuchs endothelial 2
100188782	NIDDM4	Diabetes mellitus, noninsulin-dependent
100188791	CLLS1	Leukemia, chronic lymphocytic, susceptibility to, 1
100188798	SLEB5	Systemic lupus erythematosus, susceptibility to, 5
100188801	ASRT3	Asthma-related traits, susceptibility to, 3
100188803	SLEB7	Systemic lupus erythematosus, susceptibility to, 7
100188804	SLEB8	Systemic lupus erythematosus, susceptibility to, 8
100188821	GOA1	Osteoarthritis, generalized, without dysplasia, susceptibility to
100188823	ASRT4	Asthma-related traits, susceptibility to, 4
100188827	SQTL1	Smoking as a quantitative trait locus 1
100188840	ASRT6	Asthma-related traits, susceptibility to, 6
100188860	CPROTQ	C-reactive protein QTL
100188865	LMPH1B	Lymphedema, hereditary, IB
100190789	TSHQTL1	Thyroid-stimulating hormone level QTL 1
100190928	SLEB12	Systemic lupus erythematosus, susceptibility to, 12
100190934	LOC100190934	alcohol dehydrogenase 5 (class III), chi polypeptide pseudogene
100191040	C2CD4D	C2 calcium-dependent domain containing 4D
100192307	MUSQTL1	Musical aptitude QTL 1
100192311	MUSTQTL1	Muscle strength quantitative trait locus 1
100194425	LOC100194425	B-cell receptor-associated protein 31 pseudogene
100194426	LOC100194426	B-cell receptor-associated protein 31 pseudogene
100196909	ALPQTL1	Alkaline phosphatase, plasma level of, QTL1
100196918	SLEB13	Systemic lupus erythematosus, susceptibility to, 13
1002	CDH4	cadherin 4, type 1, R-cadherin (retinal)
10020	GNE	glucosamine (UDP-N-acetyl)-2-epimerase/N-acetylmannosamine kinase
10023	FRAT1	frequently rearranged in advanced T-cell lymphomas
100233159	WAGRO	WAGRO syndrome
10024	TROAP	trophinin associated protein (tastin)
100270642	CLLS3	Leukemia, chronic lymphocytic, susceptibility to, 3
100270643	CLLS4	Leukemia, chronic lymphocytic susceptibility to, 4
100270644	CLLS5	Leukemia, chronic lymphocytic susceptiblity to, 5
100270752	MFRP-C1QTNF5	membrane frizzled-related protein, C1q and tumor necrosis factor related protein 5 transcription unit
100270794	STQTL10	Stature QTL 10

100270795	STQTL11	Stature QTL 11
100270796	STQTL13	Stature QTL 13
100270797	STQTL14	Stature QTL 14
100270801	STQTL15	Stature QTL 15
100270802	STQTL16	Stature QTL 16
100271694	MS2	Multiple sclerosis, susceptibility to, 2
100271695	MS3	Multiple sclerosis, susceptibility to, 3
100271696	MS4	Multiple sclerosis, susceptiblity to, 4
100271697	IDDM23	Diabetes mellitus, insulin-dependent, 23
100271698	ADIPQTL4	Adiponectin, serum level of, QTL4
100271867	MPVQTL1	Mean platelet volume QTL1
100271868	MPVQTL2	Mean platelet volume QTL2
100271869	MPVQTL3	Mean platelet volume QTL3
100271873	AIRN	antisense of IGF2R RNA (non-protein coding)
100271918	LOC100271918	antigen identified by monoclonal antibody Ki-67 pseudogene
100272146	LOC100272146	nuclear factor (erythroid-derived 2)-like 3 pseudogene
100272147	MTCP1NB	mature T-cell proliferation 1 neighbor
100272224	UAQTL3	Uric acid concentration, serum, QTL3
100272225	UAQTL4	Uric acid concentration, serum, QTL4
100286836	OS6	Osteoarthritis susceptibility 6
100286963	DEFB109	defensin, beta 109
100287066	LOC100287066	beta-defensin 131-like
100287083	DEFT1P2	defensin, theta 1 pseudogene 2
100287128	IGHV1OR15-2	immunoglobulin heavy variable 1/OR15-2 (pseudogene)
100287290	LOC100287290	cytokine receptor CRL2
100287372	IGHV3OR16-13	immunoglobulin heavy variable 3/OR16-13 (non-functional)
100287457	KIR2DL2	killer cell immunoglobulin-like receptor, two domains, long cytoplasmic tail, 2
100287520	GLYATL1P3	glycine-N-acyltransferase-like 1 pseudogene 3
100287528	IGLL4P	immunoglobulin lambda-like polypeptide 4, pseudogene
100287534	LOC100287534	killer cell immunoglobulin-like receptor 2DL4-like
100288590	LOC100288590	cutaneous T-cell lymphoma-associated antigen 1 pseudogene
100288695	LIMS3L	LIM and senescent cell antigen-like domains 3-like
100288719	LOC100288719	prostaglandin E synthase 3-like
100288978	LOC100288978	myeloma-overexpressed gene 2 protein homolog
100289350	LOC100289350	immunoglobulin superfamily, member 3 pseudogene
100289462	DEFB4B	defensin, beta 4B
100289619	LOC100289619	TP53 regulated inhibitor of apoptosis 1 pseudogene

100289853	LOC100289853	integrin alpha-X-like
100290309	LOC100290309	IgGFc-binding protein-like
100290366	LOC100290366	disintegrin and metalloproteinase domain-containing protein 29-like
100290481	CYAT1	immunoglobulin lambda light chain-like
100290966	HLA-DRB1	major histocompatibility complex, class II, DR beta 1
100291699	LOC100291699	A disintegrin and metalloproteinase with thrombospondin motifs 7-like
100293534	LOC100293534	complement C4-B-like
100293977	LOC100293977	HLA class II histocompatibility antigen, DQ beta 1 chain-like
100294036	LOC100294036	HLA class II histocompatibility antigen, DRB1-9 beta chain-like
100294044	LOC100294044	ig heavy chain V region 5A-like
100294276	LOC100294276	HLA class II histocompatibility antigen, DRB1-3 chain-like
100294339	LOC100294339	putative apoptosis-related protein 2-like
100294468	LOC100294468	HLA class II histocompatibility antigen, DRB1-9 beta chain-like
100294719	LBMQTL1	Lean body mass QTL 1
1003	CDH5	cadherin 5, type 2 (vascular endothelium)
100301523	STQTL12	Stature QTL 12
100301526	STQTL17	Stature QTL 17
100302511	EE	Esophagitis, eosinophilic
100302559	PURAQTL1	Polyunsaturated fatty acids plasma level QTL1
100302683	STQTL18	Stature QTL 18
100302684	STQTL19	Stature QTL 19
100302718	B6QTL1	Vitamin B6 plasma level QTL 1
100302740	FAS-AS1	FAS antisense RNA 1 (non-protein coding)
100303715	IDDM24	Diabetes mellitus, insulin-dependent, 24
100306940	FL1	Follicular lymphoma, susceptibility to, 1
100310755	ATOD7	Dermatitis, atopic, susceptibility to, 7
100310785	ALL1	Leukemia, acute lymphocytic, susceptibility to, 1
100310786	ALL2	Leukemia, acute lymphoblastic, susceptibility to, 2
100310869	IGKV2OR2-7D	immunoglobulin kappa variable 2/OR2-7D (pseudogene)
100312985	IGKV1OR15-118	immunoglobulin kappa variable 1/OR15-118 (pseudogene)
100313773	MIR548H2	microRNA 548h-2
100322878	SLEB14	Systemic lupus erythematosus, susceptiblity to, 14
100379216	FGQTL2	Fasting plasma glucose level QTL2
100379217	FGQTL3	Fasting plasma glucose level QTL3
100380874	FECD5	Corneal dystrophy, Fuchs endothelial, 5
100380875	FECD7	Corneal dystrophy, Fuchs endothelial, 7
100384884	IGHV1OR15-5	immunoglobulin heavy variable 1/OR15-5 (pseudogene)

1004	CDH6	cadherin 6, type 2, K-cadherin (fetal kidney)
100415896	ASRT8	Asthma-related traits, susceptibility to, 8
100418597	LOC100418597	MHC class I polypeptide-related sequence B pseudogene
100418598	LOC100418598	MHC class I polypeptide-related sequence B pseudogene
100418599	LOC100418599	MHC class I polypeptide-related sequence B pseudogene
100418600	LOC100418600	MHC class I polypeptide-related sequence B pseudogene
100418601	LOC100418601	MHC class I polypeptide-related sequence B pseudogene
100418602	LOC100418602	MHC class I polypeptide-related sequence B pseudogene
100418603	LOC100418603	MHC class I polypeptide-related sequence B pseudogene
100418604	LOC100418604	MHC class I polypeptide-related sequence B pseudogene
100418605	LOC100418605	MHC class I polypeptide-related sequence B pseudogene
100418606	LOC100418606	MHC class I polypeptide-related sequence B pseudogene
100418607	LOC100418607	MHC class I polypeptide-related sequence B pseudogene
100418617	LOC100418617	major histocompatibility complex, class I, B pseudogene
100418693	LOC100418693	protein kinase, interferon-inducible double stranded RNA dependent activator pseudogene
100418694	LOC100418694	protein kinase, interferon-inducible double stranded RNA dependent activator pseudogene
100418874	LOC100418874	cathepsin B pseudogene
100419077	LOC100419077	Fc receptor-like 6 pseudogene
100419149	HCFC2P1	host cell factor C2 pseudogene 1
100419318	LOC100419318	FAST kinase domains 3 pseudogene
100419324	LOC100419324	B-cell CLL/lymphoma 9 pseudogene
100419615	LOC100419615	complement component 7 pseudogene
100419668	LOC100419668	protein kinase C, eta pseudogene
100419811	LOC100419811	interleukin 9 receptor pseudogene
100419977	LOC100419977	myeloid leukemia factor 2 pseudogene
100420027	LOC100420027	dedicator of cytokinesis 9 pseudogene
100420406	IRF5P1	interferon regulatory factor 5 pseudogene 1
100420423	LOC100420423	FK506 binding protein 8, 38kDa pseudogene
100420484	LOC100420484	thymocyte selection associated pseudogene
100420509	LOC100420509	X-linked inhibitor of apoptosis pseudogene
100420513	LOC100420513	TNF receptor-associated factor 4 pseudogene
100420526	LOC100420526	PRKC, apoptosis, WT1, regulator pseudogene
100420535	LOC100420535	PRKC, apoptosis, WT1, regulator pseudogene
100420536	LOC100420536	islet cell autoantigen 1, 69kDa pseudogene
100420539	LOC100420539	FK506 binding protein 10, 65 kDa pseudogene
100420641	LOC100420641	TNF receptor-associated factor 7 pseudogene
100420748	LOC100420748	suppressor of cytokine signaling 5 pseudogene

100420754	LOC100420754	suppressor of cytokine signaling 5 pseudogene
100420759	LOC100420759	suppressor of cytokine signaling 6 pseudogene
100420797	LOC100420797	Fc fragment of IgG binding protein pseudogene
100420800	LOC100420800	suppressor of cytokine signaling 6 pseudogene
100420885	LOC100420885	suppressor of cytokine signaling 2 pseudogene
100420968	LOC100420968	Ly1 antibody reactive homolog (mouse) pseudogene
100421002	LOC100421002	hepatitis A virus cellular receptor 1 pseudogene
100421061	LOC100421061	hepatitis A virus cellular receptor 1 pseudogene
100421129	LOC100421129	interleukin 28B (interferon, lambda 3) pseudogene
100421207	LOC100421207	Fanconi anemia, complementation group L pseudogene
100421239	LOC100421239	Fanconi anemia, complementation group D2 pseudogene
100421484	LOC100421484	MAP/microtubule affinity-regulating kinase 2 pseudogene
100421485	MARK2P5	MAP/microtubule affinity-regulating kinase 2 pseudogene 5
100421494	LOC100421494	MAP/microtubule affinity-regulating kinase 2 pseudogene
100421496	MARK2P4	MAP/microtubule affinity-regulating kinase 2 pseudogene 4
100421498	MARK2P8	MAP/microtubule affinity-regulating kinase 2 pseudogene 8
100421499	LOC100421499	MAP/microtubule affinity-regulating kinase 4 pseudogene
100421500	MARK2P6	MAP/microtubule affinity-regulating kinase 2 pseudogene 6
100421501	MARK2P1	MAP/microtubule affinity-regulating kinase 2 pseudogene 1
100421502	MARK2P2	MAP/microtubule affinity-regulating kinase 2 pseudogene 2
100421503	LOC100421503	MAP/microtubule affinity-regulating kinase 3 pseudogene
100421504	LOC100421504	MAP/microtubule affinity-regulating kinase 4 pseudogene
100421505	LOC100421505	MAP/microtubule affinity-regulating kinase 4 pseudogene
100421506	LOC100421506	interleukin enhancer binding factor 2, 45kDa pseudogene
100421507	MARK2P3	MAP/microtubule affinity-regulating kinase 2 pseudogene 3
100421508	LOC100421508	MAP/microtubule affinity-regulating kinase 2 pseudogene
100421511	LOC100421511	MAP/microtubule affinity-regulating kinase 2 pseudogene
100421531	MARK2P7	MAP/microtubule affinity-regulating kinase 2 pseudogene 7
100421550	LOC100421550	MAP/microtubule affinity-regulating kinase 3 pseudogene
100421557	LOC100421557	caspase recruitment domain family, member 16 pseudogene
100421558	LOC100421558	MAP/microtubule affinity-regulating kinase 2 pseudogene
100421565	MARK2P12	MAP/microtubule affinity-regulating kinase 2 pseudogene 12
100421610	LOC100421610	complement component 4 binding protein, alpha pseudogene
100421630	LOC100421630	cutaneous T-cell lymphoma-associated antigen 1 pseudogene
100421663	LOC100421663	cutaneous T-cell lymphoma-associated antigen 1 pseudogene
100421901	LOC100421901	immunoglobulin (CD79A) binding protein 1 pseudogene pseudogene
100421976	LOC100421976	interferon stimulated exonuclease gene 20kDa-like 2 pseudogene

100422032	LOC100422032	intraflagellar transport 80 homolog (Chlamydomonas) pseudogene
100422053	LOC100422053	intraflagellar transport 57 homolog (Chlamydomonas) pseudogene
100422271	LOC100422271	proteasome (prosome, macropain) 26S subunit, ATPase, 2 pseudogene
100422275	LOC100422275	proteasome (prosome, macropain) subunit, beta type, 5 pseudogene
100422286	LOC100422286	proteasome (prosome, macropain) subunit, alpha type, 1 pseudogene
100422287	LOC100422287	proteasome (prosome, macropain) subunit, alpha type, 3 pseudogene
100422289	LOC100422289	proteasome (prosome, macropain) subunit, beta type, 7 pseudogene
100422299	LOC100422299	complement component 1, q subcomponent binding protein pseudogene
100422305	PSMC1P13	proteasome (prosome, macropain) 26S subunit, ATPase, 1 pseudogene 13
100422310	LOC100422310	complement component 1, q subcomponent binding protein pseudogene
100422317	LOC100422317	proteasome (prosome, macropain) 26S subunit, ATPase, 5 pseudogene
100422386	LOC100422386	adenosine deaminase domain containing 1 (testis-specific) pseudogene
100422407	LOC100422407	adenosine deaminase domain containing 1 (testis-specific) pseudogene
100422411	LOC100422411	proteasome (prosome, macropain) 26S subunit, non-ATPase, 8 pseudogene
100422413	LOC100422413	proteasome (prosome, macropain) 26S subunit, non-ATPase, 7 pseudogene
100422414	LOC100422414	proteasome (prosome, macropain) 26S subunit, non-ATPase, 7 pseudogene
100422418	LOC100422418	proteasome (prosome, macropain) 26S subunit, non-ATPase, 12 pseudogene
100422434	LOC100422434	complement component (3b/4b) receptor 1 (Knops blood group) pseudogene
100422479	LOC100422479	integrin, alpha X (complement component 3 receptor 4 subunit) pseudogene
100422526	LOC100422526	proteasome (prosome, macropain) activator subunit 3 (PA28 gamma; Ki) pseudogene
100422531	LOC100422531	FCF1 small subunit (SSU) processome component homolog (S. cerevisiae) pseudogene
100422533	LOC100422533	FCF1 small subunit (SSU) processome component homolog (S. cerevisiae) pseudogene
100422535	LOC100422535	FCF1 small subunit (SSU) processome component homolog (S. cerevisiae) pseudogene
100422536	LOC100422536	FCF1 small subunit (SSU) processome component homolog (S. cerevisiae) pseudogene
100422538	LOC100422538	recombination signal binding protein for immunoglobulin kappa J region pseudogene
100422546	LOC100422546	X-ray repair complementing defective repair in Chinese hamster cells 6 pseudogene
100422548	LOC100422548	solute carrier family 5 (low affinity glucose cotransporter), member 4 pseudogene
100422549	LOC100422549	solute carrier family 5 (low affinity glucose cotransporter), member 4 pseudogene
100422550	LOC100422550	solute carrier family 5 (low affinity glucose cotransporter), member 4 pseudogene
100422564	LOC100422564	glucosaminyl (N-acetyl) transferase 2, I-branching enzyme (I blood group) pseudogene
100422581	LOC100422581	DNA fragmentation factor, 40kDa, beta polypeptide (caspase-activated DNase) pseudogene
100422587	LOC100422587	platelet-activating factor acetylhydrolase 1b, regulatory subunit 1 (45kDa) pseudogene
100422604	LOC100422604	leukocyte immunoglobulin-like receptor, subfamily A (with TM domain), member 2 pseudogene
100422611	LOC100422611	leukocyte immunoglobulin-like receptor, subfamily A (with TM domain), member 2 pseudogene
100422612	LOC100422612	leukocyte immunoglobulin-like receptor, subfamily A (with TM domain), member 6 pseudogene
100422623	LOC100422623	carcinoembryonic antigen-related cell adhesion molecule 1 (biliary glycoprotein) pseudogene
100422634	LOC100422634	caspase 1, apoptosis-related cysteine peptidase (interleukin 1, beta, convertase) pseudogene

100422637	LOC100422637	myxovirus (influenza virus) resistance 1, interferon-inducible protein p78 (mouse) pseudogene
100422638	LOC100422638	myxovirus (influenza virus) resistance 1, interferon-inducible protein p78 (mouse) pseudogene
100422639	LOC100422639	myxovirus (influenza virus) resistance 1, interferon-inducible protein p78 (mouse) pseudogene
100422691	ITGB1P1	integrin beta 1 pseudogene 1
100422711	PRKRIRP2	protein-kinase, interferon-inducible double stranded RNA dependent inhibitor, repressor of (P58 repressor) pseudogene 2
100422712	PRKRIRP7	protein-kinase, interferon-inducible double stranded RNA dependent inhibitor, repressor of (P58 repressor) pseudogene 7
100422715	PRKRIRP10	protein-kinase, interferon-inducible double stranded RNA dependent inhibitor, repressor of (P58 repressor) pseudogene 10
100423062	IGLL5	immunoglobulin lambda-like polypeptide 5
10043	TOM1	target of myb1 (chicken)
100431172	KLRF2	killer cell lectin-like receptor subfamily F, member 2
100463528	LSP1P1	lymphocyte-specific protein 1 pseudogene 1
10049	DNAJB6	DnaJ (Hsp40) homolog, subfamily B, member 6
100499165	FGQTL1	Fasting plasma glucose level QTL 1
100499167	EOE2	Esophagitis, eosinophilic, 2
100499168	FGQTL6	Fasting plasma glucose level QTL 6
100499169	FGQTL4	Fasting plasma glucose level QTL 4
100499223	LOC100499223	beta-1,3-N-acetylgalactosaminyltransferase 1 (globoside blood group) pseudogene
1005	CDH7	cadherin 7, type 2
100505873	LOC100505873	putative apoptosis-related protein 2-like
100505995	LOC100505995	apoptosis-inducing, TAF9-like domain 1 pseudogene
100506173	LOC100506173	putative killer cell immunoglobulin-like receptor like protein KIR3DP1-like
100506197	LOC100506197	rRNA-processing protein FCF1 homolog
100506215	GCFC1-AS1	GCFC1 antisense RNA 1 (non-protein coding)
100506311	HOTAIRM1	HOXA transcript antisense RNA, myeloid-specific 1 (non-protein coding)
100506330	LOC100506330	outcome predictor in acute leukemia 1-like
100506658	OCLN	occludin
100506736	SLFN12L	schlafen family member 12-like
100506742	CASP12	caspase 12 (gene/pseudogene)
100507399	HCG8	HLA complex group 8
100507436	MICA	MHC class I polypeptide-related sequence A
100507674	MARK2P9	MAP/microtubule affinity-regulating kinase 2 pseudogene 9
100507680	LOC100507680	HLA class I histocompatibility antigen, A-74 alpha chain-like
100507685	LOC100507685	complement C4-B-like
100507686	LOC100507686	HLA class II histocompatibility antigen, DQ alpha 1 chain-like

100507687	LOC100507687	HLA class II histocompatibility antigen, DQ beta 1 chain-like
100507703	LOC100507703	HLA class I histocompatibility antigen, A-69 alpha chain-like
100507709	LOC100507709	HLA class II histocompatibility antigen, DRB1-7 beta chain-like
100507710	LOC100507710	HLA class II histocompatibility antigen, DQ beta 1 chain-like
100507712	LOC100507712	HLA class II histocompatibility antigen, DRB1-15 beta chain-like
100507714	LOC100507714	HLA class II histocompatibility antigen, DRB1-7 beta chain-like
100507716	LOC100507716	HLA class II histocompatibility antigen, DR beta 4 chain-like
100507718	LOC100507718	HLA class II histocompatibility antigen, DQ alpha 1 chain-like
100507719	LOC100507719	HLA class II histocompatibility antigen, DQ beta 1 chain-like
100507738	LOC100507738	A disintegrin and metalloproteinase with thrombospondin motifs 7-like
100507758	LOC100507758	rRNA-processing protein FCF1 homolog
100508148	LOC100508148	immunoglobulin-binding protein 1-like
100508251	LOC100508251	putative V-set and immunoglobulin domain-containing protein 6-like
100508392	LOC100508392	putative V-set and immunoglobulin domain-containing protein 7-like
100508509	LOC100508509	putative apoptosis-related protein 2-like
100509001	LOC100509001	complement C4-B-like
100509003	LOC100509003	putative T-cell surface glycoprotein CD8 beta-2 chain-like
100509246	LOC100509246	HLA class II histocompatibility antigen, DRB1-10 beta chain-like
100509296	LOC100509296	HLA class II histocompatibility antigen, DR beta 3 chain-like
100509301	LOC100509301	HLA class II histocompatibility antigen, DR beta 4 chain-like
100509325	LOC100509325	HLA class II histocompatibility antigen, DQ beta 1 chain-like
100509457	LOC100509457	HLA class II histocompatibility antigen, DQ alpha 1 chain-like
100509582	LOC100509582	HLA class II histocompatibility antigen, DR beta 4 chain-like
100509607	LOC100509607	HLA class II histocompatibility antigen, DRB1-4 beta chain-like
100509670	LOC100509670	complement factor H-related protein 3-like
100509765	LOC100509765	MHC class I polypeptide-related sequence B-like
100510044	LOC100510044	immunoglobulin kappa locus-like
100510059	LOC100510059	HLA class II histocompatibility antigen, DQ alpha 1 chain-like
100510144	LOC100510144	leukocyte immunoglobulin-like receptor subfamily B member 3-like
100510200	LOC100510200	leukocyte immunoglobulin-like receptor subfamily B member 3-like
100510280	LOC100510280	HLA class II histocompatibility antigen, DRB1-4 beta chain-like
100510306	LOC100510306	HLA class II histocompatibility antigen, DQ beta 1 chain-like
100510327	LOC100510327	HLA class I histocompatibility antigen, A-30 alpha chain-like
100510361	LOC100510361	putative V-set and immunoglobulin domain-containing protein 6-like
100510436	LOC100510436	HLA class I histocompatibility antigen, B-44 alpha chain-like
100510454	LOC100510454	integrin alpha-X-like
100510495	LOC100510495	HLA class II histocompatibility antigen, DR beta 3 chain-like

100510517	LOC100510517	HLA class II histocompatibility antigen, DQ beta 1 chain-like
100510519	LOC100510519	HLA class II histocompatibility antigen, DR beta 4 chain-like
100510524	LOC100510524	TP53-regulated inhibitor of apoptosis 1-like
100510559	LOC100510559	HLA class II histocompatibility antigen, DP alpha 1 chain-like
100510623	LOC100510623	HLA class II histocompatibility antigen, DQ alpha 1 chain-like
100510687	LOC100510687	HLA class II histocompatibility antigen, DR beta 3 chain-like
100510688	LOC100510688	HLA class II histocompatibility antigen, DQ beta 1 chain-like
100510689	LOC100510689	HLA class II histocompatibility antigen, DQ beta 1 chain-like
100526664	LY75-CD302	LY75-CD302 readthrough
100527946	LOC100527946	programmed cell death 5 pseudogene
100527947	LOC100527947	programmed cell death 5 pseudogene
100528025	SXGQTL1	Sex hormone-binding blobulin circulating level QTL 1
100528032	KLRC4-KLRK1	KLRC4-KLRK1 readthrough
100532726	NDUFC2-KCTD14	NDUFC2-KCTD14 readthrough
100533107	RTEL1-TNFRSF6B	RTEL1-TNFRSF6B readthrough
100533629	LOC100533629	FAST kinase domains 1 pseudogene
100533747	MARK2P11	MAP/microtubule affinity-regulating kinase 2 pseudogene 11
100533754	LOC100533754	MAP/microtubule affinity-regulating kinase 3 pseudogene
100533755	LOC100533755	MAP/microtubule affinity-regulating kinase 2 pseudogene
100533794	LOC100533794	MAP/microtubule affinity-regulating kinase 2 pseudogene
100533795	LOC100533795	MAP/microtubule affinity-regulating kinase 2 pseudogene
100533797	LOC100533797	MAP/microtubule affinity-regulating kinase 2 pseudogene
100533852	LOC100533852	nuclear factor of kappa light polypeptide gene enhancer in B-cells 2 (p49/p100) pseudogene
100533853	LOC100533853	protein-kinase, interferon-inducible double stranded RNA dependent inhibitor, repressor of (P58 repressor) pseudogene
100533936	LOC100533936	major histocompatibility complex, class I, B pseudogene
100534012	TNFAIP8L2-SCNM1	TNFAIP8L2-SCNM1 readthrough
100534612	C1QTNF3-AMACR	C1QTNF3-AMACR readthrough
100568447	IGHV3OR16-14	immunoglobulin heavy variable 3/OR16-14 (pseudogene)
10057	ABCC5	ATP-binding cassette, sub-family C (CFTR/MRP), member 5
10059	DNM1L	dynamin 1-like
1006	CDH8	cadherin 8, type 2
10062	NR1H3	nuclear receptor subfamily 1, group H, member 3
10068	IL18BP	interleukin 18 binding protein
1007	CDH9	cadherin 9, type 2 (T1-cadherin)
10076	PTPRU	protein tyrosine phosphatase, receptor type, U
1008	CDH10	cadherin 10, type 2 (T2-cadherin)

10081	PDCD7	programmed cell death 7
10085	EDIL3	EGF-like repeats and discoidin I-like domains 3
1009	CDH11	cadherin 11, type 2, OB-cadherin (osteoblast)
100918	NRCLP	Narcolepsy, HLA-associated
101	ADAM8	ADAM metallopeptidase domain 8
1010	CDH12	cadherin 12, type 2 (N-cadherin 2)
10105	PPIF	peptidylprolyl isomerase F
10110	SGK2	serum/glucocorticoid regulated kinase 2
10112	KIF20A	kinesin family member 20A
10114	HIPK3	homeodomain interacting protein kinase 3
10116	FEM1B	fem-1 homolog b (C. elegans)
1012	CDH13	cadherin 13, H-cadherin (heart)
10124	ARL4A	ADP-ribosylation factor-like 4A
10125	RASGRP1	RAS guanyl releasing protein 1 (calcium and DAG-regulated)
1013	CDH15	cadherin 15, type 1, M-cadherin (myotubule)
10131	TRAP1	TNF receptor-associated protein 1
10134	BCAP31	B-cell receptor-associated protein 31
10135	NAMPT	nicotinamide phosphoribosyltransferase
1014	CDH16	cadherin 16, KSP-cadherin
10148	EBI3	Epstein-Barr virus induced 3
1015	CDH17	cadherin 17, LI cadherin (liver-intestine)
10154	PLXNC1	plexin C1
1016	CDH18	cadherin 18, type 2
10163	WASF2	WAS protein family, member 2
10164	CHST4	carbohydrate (N-acetylglucosamine 6-O) sulfotransferase 4
10174	SORBS3	sorbin and SH3 domain containing 3
10181	RBM5	RNA binding motif protein 5
10193	RNF41	ring finger protein 41
10197	PSME3	proteasome (prosome, macropain) activator subunit 3 (PA28 gamma; Ki)
102	ADAM10	ADAM metallopeptidase domain 10
1020	CDK5	cyclin-dependent kinase 5
10201	NME6	non-metastatic cells 6, protein expressed in (nucleoside-diphosphate kinase)
10202	DHRS2	dehydrogenase/reductase (SDR family) member 2
10205	MPZL2	myelin protein zero-like 2
10210	TOPORS	topoisomerase I binding, arginine/serine-rich, E3 ubiquitin protein ligase
10213	PSMD14	proteasome (prosome, macropain) 26S subunit, non-ATPase, 14
10219	KLRG1	killer cell lectin-like receptor subfamily G, member 1

10220	GDF11	growth differentiation factor 11
10221	TRIB1	tribbles homolog 1 (Drosophila)
10224	ZNF443	zinc finger protein 443
10225	CD96	CD96 molecule
10227	MFSD10	major facilitator superfamily domain containing 10
10232	MSLN	mesothelin
10234	LRRC17	leucine rich repeat containing 17
10235	RASGRP2	RAS guanyl releasing protein 2 (calcium and DAG-regulated)
10241	CALCOCO2	calcium binding and coiled-coil domain 2
10242	KCNMB2	potassium large conductance calcium-activated channel, subfamily M, beta member 2
10244	RABEPK	Rab9 effector protein with kelch motifs
10253	SPRY2	sprouty homolog 2 (Drosophila)
10255	HCG9	HLA complex group 9 (non-protein coding)
10257	ABCC4	ATP-binding cassette, sub-family C (CFTR/MRP), member 4
1026	CDKN1A	cyclin-dependent kinase inhibitor 1A (p21, Cip1)
10261	IGSF6	immunoglobulin superfamily, member 6
10266	RAMP2	receptor (G protein-coupled) activity modifying protein 2
10268	RAMP3	receptor (G protein-coupled) activity modifying protein 3
1027	CDKN1B	cyclin-dependent kinase inhibitor 1B (p27, Kip1)
10272	FSTL3	follistatin-like 3 (secreted glycoprotein)
10276	NET1	neuroepithelial cell transforming 1
10277	UBE4B	ubiquitination factor E4B
10278	EFS	embryonal Fyn-associated substrate
10279	PRSS16	protease, serine, 16 (thymus)
1028	CDKN1C	cyclin-dependent kinase inhibitor 1C (p57, Kip2)
10285	SMNDC1	survival motor neuron domain containing 1
10288	LILRB2	leukocyte immunoglobulin-like receptor, subfamily B (with TM and ITIM domains), member 2
1029	CDKN2A	cyclin-dependent kinase inhibitor 2A (melanoma, p16, inhibits CDK4)
10293	TRAIP	TRAF interacting protein
10296	MAEA	macrophage erythroblast attacher
103	ADAR	adenosine deaminase, RNA-specific
1030	CDKN2B	cyclin-dependent kinase inhibitor 2B (p15, inhibits CDK4)
10301	DLEU1	deleted in lymphocytic leukemia 1 (non-protein coding)
1031	CDKN2C	cyclin-dependent kinase inhibitor 2C (p18, inhibits CDK4)
10312	TCIRG1	T-cell, immune regulator 1, ATPase, H+ transporting, lysosomal V0 subunit A3
10313	RTN3	reticulon 3
10318	TNIP1	TNFAIP3 interacting protein 1

10319	LAMC3	laminin, gamma 3
1032	CDKN2D	cyclin-dependent kinase inhibitor 2D (p19, inhibits CDK4)
10320	IKZF1	IKAROS family zinc finger 1 (Ikaros)
10332	CLEC4M	C-type lectin domain family 4, member M
10333	TLR6	toll-like receptor 6
10335	MRVI1	murine retrovirus integration site 1 homolog
10344	CCL26	chemokine (C-C motif) ligand 26
10347	ABCA7	ATP-binding cassette, sub-family A (ABC1), member 7
10365	KLF2	Kruppel-like factor 2 (lung)
10367	CBARA1	calcium binding atopy-related autoantigen 1
10370	CITED2	Cbp/p300-interacting transactivator, with Glu/Asp-rich carboxy-terminal domain, 2
10371	SEMA3A	sema domain, immunoglobulin domain (Ig), short basic domain, secreted, (semaphorin) 3A
10379	IRF9	interferon regulatory factor 9
10383	TUBB2C	tubulin, beta 2C
10385	BTN2A2	butyrophilin, subfamily 2, member A2
10388	SYCP2	synaptonemal complex protein 2
10392	NOD1	nucleotide-binding oligomerization domain containing 1
10394	PRG3	proteoglycan 3
10395	DLC1	deleted in liver cancer 1
10397	NDRG1	N-myc downstream regulated 1
10399	GNB2L1	guanine nucleotide binding protein (G protein), beta polypeptide 2-like 1
104	ADARB1	adenosine deaminase, RNA-specific, B1
1041	CDSN	corneodesmosin
10410	IFITM3	interferon induced transmembrane protein 3
10411	RAPGEF3	Rap guanine nucleotide exchange factor (GEF) 3
10417	SPON2	spondin 2, extracellular matrix protein
10418	SPON1	spondin 1, extracellular matrix protein
10421	CD2BP2	CD2 (cytoplasmic tail) binding protein 2
10428	CFDP1	craniofacial development protein 1
1043	CD52	CD52 molecule
10437	IFI30	interferon, gamma-inducible protein 30
10438	C1D	C1D nuclear receptor corepressor
10446	LRRN2	leucine rich repeat neuronal 2
10447	FAM3C	family with sequence similarity 3, member C
10449	ACAA2	acetyl-CoA acyltransferase 2
10450	PPIE	peptidylprolyl isomerase E (cyclophilin E)
10451	VAV3	vav 3 guanine nucleotide exchange factor

10454	TAB1	TGF-beta activated kinase 1/MAP3K7 binding protein 1
10456	HAX1	HCLS1 associated protein X-1
10457	GPNMB	glycoprotein (transmembrane) nmb
10461	MERTK	c-mer proto-oncogene tyrosine kinase
10462	CLEC10A	C-type lectin domain family 10, member A
10465	PPIH	peptidylprolyl isomerase H (cyclophilin H)
1048	CEACAM5	carcinoembryonic antigen-related cell adhesion molecule 5
10487	CAP1	CAP, adenylate cyclase-associated protein 1 (yeast)
10488	CREB3	cAMP responsive element binding protein 3
10490	VTI1B	vesicle transport through interaction with t-SNAREs homolog 1B (yeast)
10497	UNC13B	unc-13 homolog B (C. elegans)
10498	CARM1	coactivator-associated arginine methyltransferase 1
105	ADARB2	adenosine deaminase, RNA-specific, B2
1050	CEBPA	CCAAT/enhancer binding protein (C/EBP), alpha
10505	SEMA4F	sema domain, immunoglobulin domain (Ig), transmembrane domain (TM) and short cytoplasmic domain, (semaphorin) 4F
10507	SEMA4D	sema domain, immunoglobulin domain (Ig), transmembrane domain (TM) and short cytoplasmic domain, (semaphorin) 4D
10509	SEMA4B	sema domain, immunoglobulin domain (Ig), transmembrane domain (TM) and short cytoplasmic domain, (semaphorin) 4B
1051	CEBPB	CCAAT/enhancer binding protein (C/EBP), beta
10512	SEMA3C	sema domain, immunoglobulin domain (Ig), short basic domain, secreted, (semaphorin) 3C
10516	FBLN5	fibulin 5
10518	CIB2	calcium and integrin binding family member 2
10519	CIB1	calcium and integrin binding 1 (calmyrin)
10524	KAT5	K(lysine) acetyltransferase 5
1053	CEBPE	CCAAT/enhancer binding protein (C/EBP), epsilon
10537	UBD	ubiquitin D
10539	GLRX3	glutaredoxin 3
1054	CEBPG	CCAAT/enhancer binding protein (C/EBP), gamma
10542	HBXIP	hepatitis B virus x interacting protein
10544	PROCR	protein C receptor, endothelial
10553	HTATIP2	HIV-1 Tat interactive protein 2, 30kDa
10554	AGPAT1	1-acylglycerol-3-phosphate O-acyltransferase 1 (lysophosphatidic acid acyltransferase, alpha)
10555	AGPAT2	1-acylglycerol-3-phosphate O-acyltransferase 2 (lysophosphatidic acid acyltransferase, beta)
10561	IFI44	interferon-induced protein 44
10562	OLFM4	olfactomedin 4

10563	CXCL13	chemokine (C-X-C motif) ligand 13
10564	ARFGEF2	ADP-ribosylation factor guanine nucleotide-exchange factor 2 (brefeldin A-inhibited)
10565	ARFGEF1	ADP-ribosylation factor guanine nucleotide-exchange factor 1(brefeldin A-inhibited)
10572	SIVA1	SIVA1, apoptosis-inducing factor
10578	GNLY	granulysin
10581	IFITM2	interferon induced transmembrane protein 2 (1-8D)
10595	ERN2	endoplasmic reticulum to nucleus signaling 2
106	ADCP1	adenosine deaminase complexing protein 1
10611	PDLIM5	PDZ and LIM domain 5
10616	RBCK1	RanBP-type and C3HC4-type zinc finger containing 1
10621	POLR3F	polymerase (RNA) III (DNA directed) polypeptide F, 39 kDa
10622	POLR3G	polymerase (RNA) III (DNA directed) polypeptide G (32kD)
10623	POLR3C	polymerase (RNA) III (DNA directed) polypeptide C (62kD)
10626	TRIM16	tripartite motif containing 16
10628	TXNIP	thioredoxin interacting protein
10630	PDPN	podoplanin
10631	POSTN	periostin, osteoblast specific factor
10636	RGS14	regulator of G-protein signaling 14
10637	LEFTY1	left-right determination factor 1
10639	HHCM	Mahlavu hepatocellular carcinoma
10640	EXOC5	exocyst complex component 5
10642	IGF2BP1	insulin-like growth factor 2 mRNA binding protein 1
10643	IGF2BP3	insulin-like growth factor 2 mRNA binding protein 3
10644	IGF2BP2	insulin-like growth factor 2 mRNA binding protein 2
10652	YKT6	YKT6 v-SNARE homolog (S. cerevisiae)
10661	KLF1	Kruppel-like factor 1 (erythroid)
10663	CXCR6	chemokine (C-X-C motif) receptor 6
10666	CD226	CD226 molecule
10669	CGREF1	cell growth regulator with EF-hand domain 1
10670	RRAGA	Ras-related GTP binding A
10672	GNA13	guanine nucleotide binding protein (G protein), alpha 13
10673	TNFSF13B	tumor necrosis factor (ligand) superfamily, member 13b
10686	CLDN16	claudin 16
10687	PNMA2	paraneoplastic antigen MA2
1069	CETN2	centrin, EF-hand protein, 2
1071	CETP	cholesteryl ester transfer protein, plasma
1072	CFL1	cofilin 1 (non-muscle)

10725	NFAT5	nuclear factor of activated T-cells 5, tonicity-responsive
10726	NUDC	nuclear distribution gene C homolog (A. nidulans)
10728	PTGES3	prostaglandin E synthase 3 (cytosolic)
10733	PLK4	polo-like kinase 4
10747	MASP2	mannan-binding lectin serine peptidase 2
1075	CTSC	cathepsin C
10752	CHL1	cell adhesion molecule with homology to L1CAM (close homolog of L1)
10755	GIPC1	GIPC PDZ domain containing family, member 1
10763	NES	nestin
10783	NEK6	NIMA (never in mitosis gene a)-related kinase 6
10787	NCKAP1	NCK-associated protein 1
1080	CFTR	cystic fibrosis transmembrane conductance regulator (ATP-binding cassette sub-family C, member 7)
10800	CYSLTR1	cysteinyl leukotriene receptor 1
10803	CCR9	chemokine (C-C motif) receptor 9
10808	HSPH1	heat shock 105kDa/110kDa protein 1
10811	NOXA1	NADPH oxidase activator 1
10814	CPLX2	complexin 2
10815	CPLX1	complexin 1
1082	CGB	chorionic gonadotropin, beta polypeptide
10824	EPAG	early lymphoid activation protein
1084	CEACAM3	carcinoembryonic antigen-related cell adhesion molecule 3
10842	C7orf16	chromosome 7 open reading frame 16
10846	PDE10A	phosphodiesterase 10A
10848	PPP1R13L	protein phosphatase 1, regulatory (inhibitor) subunit 13 like
10849	CD3EAP	CD3e molecule, epsilon associated protein
1085	CEACAMP5	carcinoembryonic antigen-related cell adhesion molecule pseudogene 5
10850	CCL27	chemokine (C-C motif) ligand 27
10859	LILRB1	leukocyte immunoglobulin-like receptor, subfamily B (with TM and ITIM domains), member 1
1086	CEACAMP11	carcinoembryonic antigen-related cell adhesion molecule pseudogene 11
10866	HCP5	HLA complex P5
10868	USP20	ubiquitin specific peptidase 20
1087	CEACAM7	carcinoembryonic antigen-related cell adhesion molecule 7
10871	CD300C	CD300c molecule
10877	CFHR4	complement factor H-related 4
10878	CFHR3	complement factor H-related 3
1088	CEACAM8	carcinoembryonic antigen-related cell adhesion molecule 8
10882	C1QL1	complement component 1, q subcomponent-like 1

10884	MRPS30	mitochondrial ribosomal protein S30
10887	PROKR1	prokineticin receptor 1
1089	CEACAM4	carcinoembryonic antigen-related cell adhesion molecule 4
10891	PPARGC1A	peroxisome proliferator-activated receptor gamma, coactivator 1 alpha
10892	MALT1	mucosa associated lymphoid tissue lymphoma translocation gene 1
10894	LYVE1	lymphatic vessel endothelial hyaluronan receptor 1
10895	PPBPL2	pro-platelet basic protein-like 2
10898	CPSF4	cleavage and polyadenylation specific factor 4, 30kDa
10899	JTB	jumping translocation breakpoint
1090	CEACAMP1	carcinoembryonic antigen-related cell adhesion molecule pseudogene 1
10904	BLCAP	bladder cancer associated protein
10906	TRAFD1	TRAF-type zinc finger domain containing 1
1091	CEACAMP2	carcinoembryonic antigen-related cell adhesion molecule pseudogene 2
10912	GADD45G	growth arrest and DNA-damage-inducible, gamma
10913	EDAR	ectodysplasin A receptor
1092	CEACAMP3	carcinoembryonic antigen-related cell adhesion molecule pseudogene 3
10922	FASTK	Fas-activated serine/threonine kinase
10928	RALBP1	ralA binding protein 1
1093	CEACAMP4	carcinoembryonic antigen-related cell adhesion molecule pseudogene 4
10935	PRDX3	peroxiredoxin 3
10938	EHD1	EH-domain containing 1
1094	CEACAMP6	carcinoembryonic antigen-related cell adhesion molecule pseudogene 6
1095	CEACAMP7	carcinoembryonic antigen-related cell adhesion molecule pseudogene 7
10955	SERINC3	serine incorporator 3
1096	CEACAMP8	carcinoembryonic antigen-related cell adhesion molecule pseudogene 8
10962	MLLT11	myeloid/lymphoid or mixed-lineage leukemia (trithorax homolog, Drosophila); translocated to, 11
10964	IFI44L	interferon-induced protein 44-like
1097	CEACAMP9	carcinoembryonic antigen-related cell adhesion molecule pseudogene 9
10979	FERMT2	fermitin family member 2
1098	CEACAMP10	carcinoembryonic antigen-related cell adhesion molecule pseudogene 10
10986	IGAD1	immunoglobulin A (IgA) deficiency susceptibility 1
10990	LILRB5	leukocyte immunoglobulin-like receptor, subfamily B (with TM and ITIM domains), member 5
11005	SPINK5	serine peptidase inhibitor, Kazal type 5
11006	LILRB4	leukocyte immunoglobulin-like receptor, subfamily B (with TM and ITIM domains), member 4
11007	CCDC85B	coiled-coil domain containing 85B
11009	IL24	interleukin 24
11019	LIAS	lipoic acid synthetase

11020	IFT27	intraflagellar transport 27 homolog (Chlamydomonas)
11021	RAB35	RAB35, member RAS oncogene family
11024	LILRA1	leukocyte immunoglobulin-like receptor, subfamily A (with TM domain), member 1
11025	LILRB3	leukocyte immunoglobulin-like receptor, subfamily B (with TM and ITIM domains), member 3
11026	LILRA3	leukocyte immunoglobulin-like receptor, subfamily A (without TM domain), member 3
11027	LILRA2	leukocyte immunoglobulin-like receptor, subfamily A (with TM domain), member 2
11034	DSTN	destrin (actin depolymerizing factor)
11035	RIPK3	receptor-interacting serine-threonine kinase 3
11037	STON1	stonin 1
11040	PIM2	pim-2 oncogene
11047	ADRM1	adhesion regulating molecule 1
11059	WWP1	WW domain containing E3 ubiquitin protein ligase 1
11060	WWP2	WW domain containing E3 ubiquitin protein ligase 2
11061	LECT1	leukocyte cell derived chemotaxin 1
11069	RAPGEF4	Rap guanine nucleotide exchange factor (GEF) 4
11083	DIDO1	death inducer-obliterator 1
11093	ADAMTS13	ADAM metallopeptidase with thrombospondin type 1 motif, 13
11095	ADAMTS8	ADAM metallopeptidase with thrombospondin type 1 motif, 8
11096	ADAMTS5	ADAM metallopeptidase with thrombospondin type 1 motif, 5
1110	CHE2	cholinesterase (serum) 2
11113	CIT	citron (rho-interacting, serine/threonine kinase 21)
11117	EMILIN1	elastin microfibril interfacer 1
11122	PTPRT	protein tyrosine phosphatase, receptor type, T
11124	FAF1	Fas (TNFRSF6) associated factor 1
11126	CD160	CD160 molecule
11128	POLR3A	polymerase (RNA) III (DNA directed) polypeptide A, 155kDa
11132	CAPN10	calpain 10
11136	SLC7A9	solute carrier family 7 (cationic amino acid transporter, y+ system), member 9
11140	CDC37	cell division cycle 37 homolog (S. cerevisiae)
11141	IL1RAPL1	interleukin 1 receptor accessory protein-like 1
11146	GLMN	glomulin, FKBP associated protein
11149	BVES	blood vessel epicardial substance
11151	CORO1A	coronin, actin binding protein, 1A
11155	LDB3	LIM domain binding 3
11167	FSTL1	follistatin-like 1
11171	STRAP	serine/threonine kinase receptor associated protein
11172	INSL6	insulin-like 6

11183	MAP4K5	mitogen-activated protein kinase kinase kinase kinase 5
11184	MAP4K1	mitogen-activated protein kinase kinase kinase kinase 1
11187	PKP3	plakophilin 3
11188	NISCH	nischarin
11197	WIF1	WNT inhibitory factor 1
11200	CHEK2	CHK2 checkpoint homolog (S. pombe)
11209	MST1P2	macrophage stimulating 1 (hepatocyte growth factor-like) pseudogene 2
11213	IRAK3	interleukin-1 receptor-associated kinase 3
11214	AKAP13	A kinase (PRKA) anchor protein 13
11215	AKAP11	A kinase (PRKA) anchor protein 11
11218	DDX20	DEAD (Asp-Glu-Ala-Asp) box polypeptide 20
11221	DUSP10	dual specificity phosphatase 10
11223	MST1P9	macrophage stimulating 1 (hepatocyte growth factor-like) pseudogene 9
11235	PDCD10	programmed cell death 10
112399	EGLN3	egl nine homolog 3 (C. elegans)
112401	BIRC8	baculoviral IAP repeat containing 8
112464	PRKCDBP	protein kinase C, delta binding protein
11251	GPR44	G protein-coupled receptor 44
11252	PACSIN2	protein kinase C and casein kinase substrate in neurons 2
11255	HRH3	histamine receptor H3
112574	SNX18	sorting nexin 18
11258	DCTN3	dynactin 3 (p22)
112609	MRAP2	melanocortin 2 receptor accessory protein 2
11261	CHP	calcium binding protein P22
112616	CMTM7	CKLF-like MARVEL transmembrane domain containing 7
11274	USP18	ubiquitin specific peptidase 18
112744	IL17F	interleukin 17F
112755	STX1B	syntaxin 1B
11276	SYNRG	synergin, gamma
1128	CHRM1	cholinergic receptor, muscarinic 1
11283	CYP4F8	cytochrome P450, family 4, subfamily F, polypeptide 8
112858	TP53RK	TP53 regulating kinase
112939	NACC1	nucleus accumbens associated 1, BEN and BTB (POZ) domain containing
1130	LYST	lysosomal trafficking regulator
11311	VPS45	vacuolar protein sorting 45 homolog (S. cerevisiae)
11314	CD300A	CD300a molecule
11317	RBPJL	recombination signal binding protein for immunoglobulin kappa J region-like

113174	SAAL1	serum amyloid A-like 1
113179	ADAT3	adenosine deaminase, tRNA-specific 3
11325	DDX42	DEAD (Asp-Glu-Ala-Asp) box polypeptide 42
11326	VSIG4	V-set and immunoglobulin domain containing 4
11328	FKBP9	FK506 binding protein 9, 63 kDa
11329	STK38	serine/threonine kinase 38
11336	EXOC3	exocyst complex component 3
113419	TEX261	testis expressed 261
11343	MGLL	monoglyceride lipase
113540	CMTM1	CKLF-like MARVEL transmembrane domain containing 1
1137	CHRNA4	cholinergic receptor, nicotinic, alpha 4
1139	CHRNA7	cholinergic receptor, nicotinic, alpha 7
1141	CHRNB2	cholinergic receptor, nicotinic, beta 2 (neuronal)
114132	SIGLEC11	sialic acid binding Ig-like lectin 11
114477	ATOD6	Dermatitis, atopic, 6
114548	NLRP3	NLR family, pyrin domain containing 3
114569	MAL2	mal, T-cell differentiation protein 2 (gene/pseudogene)
114609	TIRAP	toll-interleukin 1 receptor (TIR) domain containing adaptor protein
114625	ERMAP	erythroblast membrane-associated protein (Scianna blood group)
1147	CHUK	conserved helix-loop-helix ubiquitous kinase
114769	CARD16	caspase recruitment domain family, member 16
114781	BTBD9	BTB (POZ) domain containing 9
114786	XKR4	XK, Kell blood group complex subunit-related family, member 4
114823	LENG8	leukocyte receptor cluster (LRC) member 8
114824	PNMA5	paraneoplastic antigen like 5
114897	C1QTNF1	C1q and tumor necrosis factor related protein 1
114898	C1QTNF2	C1q and tumor necrosis factor related protein 2
114899	C1QTNF3	C1q and tumor necrosis factor related protein 3
1149	CIDEA	cell death-inducing DFFA-like effector a
114900	C1QTNF4	C1q and tumor necrosis factor related protein 4
114902	C1QTNF5	C1q and tumor necrosis factor related protein 5
114904	C1QTNF6	C1q and tumor necrosis factor related protein 6
114905	C1QTNF7	C1q and tumor necrosis factor related protein 7
115123	MARCH3	membrane-associated ring finger (C3HC4) 3
115350	FCRL1	Fc receptor-like 1
115352	FCRL3	Fc receptor-like 3
1154	CISH	cytokine inducible SH2-containing protein

115548	FCHO2	FCH domain only 2
115584	SLC5A11	solute carrier family 5 (sodium/glucose cotransporter), member 11
115650	TNFRSF13C	tumor necrosis factor receptor superfamily, member 13C
115653	KIR3DL3	killer cell immunoglobulin-like receptor, three domains, long cytoplasmic tail, 3
115677	NOSTRIN	nitric oxide synthase trafficker
115727	RASGRP4	RAS guanyl releasing protein 4
115827	RAB3C	RAB3C, member RAS oncogene family
1161	ERCC8	excision repair cross-complementing rodent repair deficiency, complementation group 8
116143	WDR92	WD repeat domain 92
116173	CMTM5	CKLF-like MARVEL transmembrane domain containing 5
116379	IL22RA2	interleukin 22 receptor, alpha 2
116449	CLNK	cytokine-dependent hematopoietic cell linker
116519	APOA5	apolipoprotein A-V
116840	CNTROB	centrobin, centrosomal BRCA2 interacting protein
116842	LEAP2	liver expressed antimicrobial peptide 2
116844	LRG1	leucine-rich alpha-2-glycoprotein 1
116986	AGAP2	ArfGAP with GTPase domain, ankyrin repeat and PH domain 2
117166	WFIKKN1	WAP, follistatin/kazal, immunoglobulin, kunitz and netrin domain containing 1
117178	SSX2IP	synovial sarcoma, X breakpoint 2 interacting protein
117187	ATOD3	Dermatitis, atopic, 3
117188	ATOD5	Dermatitis, atopic, 5
117285	DEFB118	defensin, beta 118
117286	CIB3	calcium and integrin binding family member 3
117289	TAGAP	T-cell activation RhoGTPase activating protein
1174	AP1S1	adaptor-related protein complex 1, sigma 1 subunit
1175	AP2S1	adaptor-related protein complex 2, sigma 1 subunit
117581	TWIST2	twist homolog 2 (Drosophila)
117584	RFFL	ring finger and FYVE-like domain containing 1
118	ADD1	adducin 1 (alpha)
1184	CLCN5	chloride channel 5
118460	EXOSC6	exosome component 6
118945	CTSL1P1	cathepsin L1 pseudogene 1
1191	CLU	clusterin
119587	CPXM2	carboxypeptidase X (M14 family), member 2
12	SERPINA3	serpin peptidase inhibitor, clade A (alpha-1 antiproteinase, antitrypsin), member 3
120	ADD3	adducin 3 (gamma)
120071	GYLTL1B	glycosyltransferase-like 1B

1201	CLN3	ceroid-lipofuscinosis, neuronal 3
120114	FAT3	FAT tumor suppressor homolog 3 (Drosophila)
120425	AMICA1	adhesion molecule, interacts with CXADR antigen 1
120892	LRRK2	leucine-rich repeat kinase 2
1209	CLPTM1	cleft lip and palate associated transmembrane protein 1
1211	CLTA	clathrin, light chain A
121131	PSMB3P	proteasome (prosome, macropain) subunit, beta type, 3 pseudogene
121227	LRIG3	leucine-rich repeats and immunoglobulin-like domains 3
1213	CLTC	clathrin, heavy chain (Hc)
121457	IKBIP	IKBKB interacting protein
1215	CMA1	chymase 1, mast cell
121512	FGD4	FYVE, RhoGEF and PH domain containing 4
121906	PSMA6P4	proteasome (prosome, macropain) subunit, alpha type, 6 pseudogene 4
122042	RXFP2	relaxin/insulin-like family peptide receptor 2
122509	IFI27L1	interferon, alpha-inducible protein 27-like 1
122706	PSMB11	proteasome (prosome, macropain) subunit, beta type, 11
122809	SOCS4	suppressor of cytokine signaling 4
1230	CCR1	chemokine (C-C motif) receptor 1
1232	CCR3	chemokine (C-C motif) receptor 3
1233	CCR4	chemokine (C-C motif) receptor 4
1234	CCR5	chemokine (C-C motif) receptor 5
1235	CCR6	chemokine (C-C motif) receptor 6
1236	CCR7	chemokine (C-C motif) receptor 7
1237	CCR8	chemokine (C-C motif) receptor 8
1238	CCBP2	chemokine binding protein 2
123862	LOC123862	interferon induced transmembrane protein pseudogene
123879	DCUN1D3	DCN1, defective in cullin neddylation 1, domain containing 3 (S. cerevisiae)
123920	CMTM3	CKLF-like MARVEL transmembrane domain containing 3
1240	CMKLR1	chemokine-like receptor 1
124056	NOXO1	NADPH oxidase organizer 1
124599	CD300LB	CD300 molecule-like family member b
124857	WFIKKN2	WAP, follistatin/kazal, immunoglobulin, kunitz and netrin domain containing 2
124872	B4GALNT2	beta-1,4-N-acetyl-galactosaminyl transferase 2
124912	SPACA3	sperm acrosome associated 3
125931	CEACAM20	carcinoembryonic antigen-related cell adhesion molecule 20
126014	OSCAR	osteoclast associated, immunoglobulin-like receptor
126259	TMIGD2	transmembrane and immunoglobulin domain containing 2

126282	TNFAIP8L1	tumor necrosis factor, alpha-induced protein 8-like 1
126298	IRGQ	immunity-related GTPase family, Q
126382	NR2C2AP	nuclear receptor 2C2-associated protein
1265	CNN2	calponin 2
126567	C2CD4C	C2 calcium-dependent domain containing 4C
1268	CNR1	cannabinoid receptor 1 (brain)
1269	CNR2	cannabinoid receptor 2 (macrophage)
1270	CNTF	ciliary neurotrophic factor
127086	LOC127086	X-ray repair complementing defective repair in Chinese hamster cells 6 pseudogene
1271	CNTFR	ciliary neurotrophic factor receptor
1272	CNTN1	contactin 1
127281	C1orf93	chromosome 1 open reading frame 93
1277	COL1A1	collagen, type I, alpha 1
1278	COL1A2	collagen, type I, alpha 2
127943	FCRLB	Fc receptor-like B
128	ADH5	alcohol dehydrogenase 5 (class III), chi polypeptide
1280	COL2A1	collagen, type II, alpha 1
1281	COL3A1	collagen, type III, alpha 1
1282	COL4A1	collagen, type IV, alpha 1
128338	DRAM2	DNA-damage regulated autophagy modulator 2
128408	BHLHE23	basic helix-loop-helix family, member e23
1285	COL4A3	collagen, type IV, alpha 3 (Goodpasture antigen)
1288	COL4A6	collagen, type IV, alpha 6
128817	CSTL1	cystatin-like 1
128859	BPIL3	bactericidal/permeability-increasing protein-like 3
1289	COL5A1	collagen, type V, alpha 1
1291	COL6A1	collagen, type VI, alpha 1
129138	ANKRD54	ankyrin repeat domain 54
1292	COL6A2	collagen, type VI, alpha 2
1293	COL6A3	collagen, type VI, alpha 3
1294	COL7A1	collagen, type VII, alpha 1
1295	COL8A1	collagen, type VIII, alpha 1
1296	COL8A2	collagen, type VIII, alpha 2
129607	CMPK2	cytidine monophosphate (UMP-CMP) kinase 2, mitochondrial
129684	CNTNAP5	contactin associated protein-like 5
129685	TAF8	TAF8 RNA polymerase II, TATA box binding protein (TBP)-associated factor, 43kDa
1297	COL9A1	collagen, type IX, alpha 1

129804	FBLN7	fibulin 7
130026	ICA1L	islet cell autoantigen 1,69kDa-like
1301	COL11A1	collagen, type XI, alpha 1
130106	CIB4	calcium and integrin binding family member 4
130120	REG3G	regenerating islet-derived 3 gamma
1302	COL11A2	collagen, type XI, alpha 2
1303	COL12A1	collagen, type XII, alpha 1
130340	AP1S3	adaptor-related protein complex 1, sigma 3 subunit
130399	ACVR1C	activin A receptor, type IC
130497	OSR1	odd-skipped related 1 (Drosophila)
1305	COL13A1	collagen, type XIII, alpha 1
130560	SPATA3	spermatogenesis associated 3
1306	COL15A1	collagen, type XV, alpha 1
1307	COL16A1	collagen, type XVI, alpha 1
130700	PSMB3P2	proteasome (prosome, macropain) subunit, beta type, 3 pseudogene 2
1310	COL19A1	collagen, type XIX, alpha 1
131096	KCNH8	potassium voltage-gated channel, subfamily H (eag-related), member 8
1311	COMP	cartilage oligomeric matrix protein
131177	FAM3D	family with sequence similarity 3, member D
1312	COMT	catechol-O-methyltransferase
131450	CD200R1	CD200 receptor 1
131566	DCBLD2	discoidin, CUB and LCCL domain containing 2
1316	KLF6	Kruppel-like factor 6
131873	COL6A6	collagen, type VI, alpha 6
132	ADK	adenosine kinase
132014	IL17RE	interleukin 17 receptor E
1326	MAP3K8	mitogen-activated protein kinase kinase kinase 8
132612	ADAD1	adenosine deaminase domain containing 1 (testis-specific)
132851	SPATA4	spermatogenesis associated 4
133	ADM	adrenomedullin
133396	IL31RA	interleukin 31 receptor A
133418	EMB	embigin
133746	JMY	junction mediating and regulatory protein, p53 cofactor
134	ADORA1	adenosine A1 receptor
134359	POC5	POC5 centriolar protein homolog (Chlamydomonas)
134492	NUDCD2	NudC domain containing 2
134637	ADAT2	adenosine deaminase, tRNA-specific 2

134728	IRAK1BP1	interleukin-1 receptor-associated kinase 1 binding protein 1
134957	STXBP5	syntaxin binding protein 5 (tomosyn)
135	ADORA2A	adenosine A2a receptor
135228	CD109	CD109 molecule
135250	RAET1E	retinoic acid early transcript 1E
1359	CPA3	carboxypeptidase A3 (mast cell)
136	ADORA2B	adenosine A2b receptor
1362	CPD	carboxypeptidase D
1364	CLDN4	claudin 4
1365	CLDN3	claudin 3
1366	CLDN7	claudin 7
137075	CLDN23	claudin 23
1373	CPS1	carbamoyl-phosphate synthase 1, mitochondrial
1378	CR1	complement component (3b/4b) receptor 1 (Knops blood group)
137814	NKX2-6	NK2 homeobox 6
1379	CR1L	complement component (3b/4b) receptor 1-like
137970	UNC5D	unc-5 homolog D (C. elegans)
1380	CR2	complement component (3d/Epstein Barr virus) receptor 2
1385	CREB1	cAMP responsive element binding protein 1
1386	ATF2	activating transcription factor 2
139189	DGKK	diacylglycerol kinase, kappa
1392	CRH	corticotropin releasing hormone
139818	DOCK11	dedicator of cytokinesis 11
1399	CRKL	v-crk sarcoma virus CT10 oncogene homolog (avian)-like
139957	LOC139957	platelet-activating factor acetylhydrolase, isoform Ib, beta subunit 30kDa pseudogene
1401	CRP	C-reactive protein, pentraxin-related
1404	HAPLN1	hyaluronan and proteoglycan link protein 1
140596	DEFB104A	defensin, beta 104A
140678	MLLT10P1	myeloid/lymphoid or mixed-lineage leukemia (trithorax homolog, Drosophila); translocated to, 10 pseudogene 1
140691	TRIM69	tripartite motif containing 69
140730	RIMS4	regulating synaptic membrane exocytosis 4
140735	DYNLL2	dynein, light chain, LC8-type 2
140850	DEFB127	defensin, beta 127
140881	DEFB129	defensin, beta 129
140885	SIRPA	signal-regulatory protein alpha
1409	CRYAA	crystallin, alpha A

1410	CRYAB	crystallin, alpha B
1432	MAPK14	mitogen-activated protein kinase 14
143244	EIF5AL1	eukaryotic translation initiation factor 5A-like 1
1434	CSE1L	CSE1 chromosome segregation 1-like (yeast)
143425	SYT9	synaptotagmin IX
143471	PSMA8	proteasome (prosome, macropain) subunit, alpha type, 8
1435	CSF1	colony stimulating factor 1 (macrophage)
1436	CSF1R	colony stimulating factor 1 receptor
143689	PIWIL4	piwi-like 4 (Drosophila)
1437	CSF2	colony stimulating factor 2 (granulocyte-macrophage)
1438	CSF2RA	colony stimulating factor 2 receptor, alpha, low-affinity (granulocyte-macrophage)
1439	CSF2RB	colony stimulating factor 2 receptor, beta, low-affinity (granulocyte-macrophage)
1440	CSF3	colony stimulating factor 3 (granulocyte)
1441	CSF3R	colony stimulating factor 3 receptor (granulocyte)
144100	PLEKHA7	pleckstrin homology domain containing, family A member 7
144383	LOC144383	interferon induced transmembrane protein pseudogene
1445	CSK	c-src tyrosine kinase
145482	PTGR2	prostaglandin reductase 2
145741	C2CD4A	C2 calcium-dependent domain containing 4A
145864	HAPLN3	hyaluronan and proteoglycan link protein 3
145908	LOC145908	similar to CHK2 checkpoint homolog (S. pombe); protein kinase Chk2; CHK2 (checkpoint, S.pombe) homolog; RAD53 homolog (S.cerevisiae)
145989	LOC145989	fascin homolog 1, actin-bundling protein (Strongylocentrotus purpuratus) pseudogene
1460	CSNK2B	casein kinase 2, beta polypeptide
1462	VCAN	versican
146223	CMTM4	CKLF-like MARVEL transmembrane domain containing 4
146225	CMTM2	CKLF-like MARVEL transmembrane domain containing 2
1463	NCAN	neurocan
146433	IL34	interleukin 34
146712	B3GNTL1	UDP-GlcNAc:betaGal beta-1,3-N-acetylglucosaminyltransferase-like 1
146722	CD300LF	CD300 molecule-like family member f
146771	TCAM1P	testicular cell adhesion molecule 1 homolog (mouse), pseudogene
146850	PIK3R6	phosphoinositide-3-kinase, regulatory subunit 6
146857	SLFN13	schlafen family member 13
146894	CD300LG	CD300 molecule-like family member g
147	ADRA1B	adrenergic, alpha-1B-, receptor
147372	CCBE1	collagen and calcium binding EGF domains 1

147409	DSG4	desmoglein 4
1476	CSTB	cystatin B (stefin B)
147645	VSIG10L	V-set and immunoglobulin domain containing 10 like
147710	IGSF23	immunoglobulin superfamily, member 23
148	ADRA1A	adrenergic, alpha-1A-, receptor
148022	TICAM1	toll-like receptor adaptor molecule 1
1482	NKX2-5	NK2 homeobox 5
148203	ZNF738	zinc finger protein 738
1487	CTBP1	C-terminal binding protein 1
1488	CTBP2	C-terminal binding protein 2
1489	CTF1	cardiotrophin 1
1490	CTGF	connective tissue growth factor
149233	IL23R	interleukin 23 receptor
149281	METTL11B	methyltransferase like 11B
1493	CTLA4	cytotoxic T-lymphocyte-associated protein 4
149371	EXOC8	exocyst complex component 8
149428	BNIPL	BCL2/adenovirus E1B 19kD interacting protein like
149461	CLDN19	claudin 19
1495	CTNNA1	catenin (cadherin-associated protein), alpha 1, 102kDa
1496	CTNNA2	catenin (cadherin-associated protein), alpha 2
149685	ADIG	adipogenin
1499	CTNNB1	catenin (cadherin-associated protein), beta 1, 88kDa
149951	COMMD7	COMM domain containing 7
149986	LSM14B	LSM14B, SCD6 homolog B (S. cerevisiae)
15	AANAT	aralkylamine N-acetyltransferase
150	ADRA2A	adrenergic, alpha-2A-, receptor
1500	CTNND1	catenin (cadherin-associated protein), delta 1
150084	IGSF5	immunoglobulin superfamily, member 5
150094	SIK1	salt-inducible kinase 1
1501	CTNND2	catenin (cadherin-associated protein), delta 2 (neural plakophilin-related arm-repeat protein)
150165	XKR3	XK, Kell blood group complex subunit-related family, member 3
150209	AIFM3	apoptosis-inducing factor, mitochondrion-associated, 3
150372	NFAM1	NFAT activating protein with ITAM motif 1
150678	MYEOV2	myeloma overexpressed 2
1508	CTSB	cathepsin B
1509	CTSD	cathepsin D
1510	CTSE	cathepsin E

1511	CTSG	cathepsin G
151112	ZSWIM2	zinc finger, SWIM-type containing 2
1512	CTSH	cathepsin H
1513	CTSK	cathepsin K
151325	MYADML	myeloid-associated differentiation marker-like
1514	CTSL1	cathepsin L1
151449	GDF7	growth differentiation factor 7
1515	CTSL2	cathepsin L2
151645	PSMC1P1	proteasome (prosome, macropain) 26S subunit, ATPase, 1 pseudogene 1
151647	FAM19A4	family with sequence similarity 19 (chemokine (C-C motif)-like), member A4
1517	CTSL1P2	cathepsin L1 pseudogene 2
1518	CTSL1P8	cathepsin L1 pseudogene 8
151888	BTLA	B and T lymphocyte associated
1519	CTSO	cathepsin O
1520	CTSS	cathepsin S
152015	ROPN1B	rhophilin associated tail protein 1B
1521	CTSW	cathepsin W
152189	CMTM8	CKLF-like MARVEL transmembrane domain containing 8
1522	CTSZ	cathepsin Z
152330	CNTN4	contactin 4
1524	CX3CR1	chemokine (C-X3-C motif) receptor 1
152404	IGSF11	immunoglobulin superfamily, member 11
1525	CXADR	coxsackie virus and adenovirus receptor
152579	SCFD2	sec1 family domain containing 2
152789	JAKMIP1	janus kinase and microtubule interacting protein 1
152992	METTL19	methyltransferase like 19
153	ADRB1	adrenergic, beta-1-, receptor
153090	DAB2IP	DAB2 interacting protein
153443	SRFBP1	serum response factor binding protein 1
1535	CYBA	cytochrome b-245, alpha polypeptide
1536	CYBB	cytochrome b-245, beta polypeptide
154	ADRB2	adrenergic, beta-2-, receptor, surface
1540	CYLD	cylindromatosis (turban tumor syndrome)
154064	RAET1L	retinoic acid early transcript 1L
1545	CYP1B1	cytochrome P450, family 1, subfamily B, polypeptide 1
154796	AMOT	angiomotin
154810	AMOTL1	angiomotin like 1

155	ADRB3	adrenergic, beta-3-, receptor
156	ADRBK1	adrenergic, beta, receptor kinase 1
157506	RDH10	retinol dehydrogenase 10 (all-trans)
157916	IL6RP1	interleukin 6 receptor pseudogene 1
158011	IFNA12P	interferon, alpha 12, pseudogene
158056	MAMDC4	MAM domain containing 4
158326	FREM1	FRAS1 related extracellular matrix 1
158441	PSMA7P	proteasome (prosome, macropain) subunit, alpha type, 7 pseudogene
158471	PRUNE2	prune homolog 2 (Drosophila)
158948	LOC158948	protein kinase C, iota pseudogene
1592	CYP26A1	cytochrome P450, family 26, subfamily A, polypeptide 1
159296	NKX2-3	NK2 homeobox 3
1594	CYP27B1	cytochrome P450, family 27, subfamily B, polypeptide 1
160	AP2A1	adaptor-related protein complex 2, alpha 1 subunit
1603	DAD1	defender against cell death 1
1604	CD55	CD55 molecule, decay accelerating factor for complement (Cromer blood group)
160410	PSMC6P2	proteasome 26S subunit, ATPase, 6 pseudogene 2
1606	DGKA	diacylglycerol kinase, alpha 80kDa
1607	DGKB	diacylglycerol kinase, beta 90kDa
160728	SLC5A8	solute carrier family 5 (iodide transporter), member 8
1608	DGKG	diacylglycerol kinase, gamma 90kDa
160851	DGKH	diacylglycerol kinase, eta
1609	DGKQ	diacylglycerol kinase, theta 110kDa
161	AP2A2	adaptor-related protein complex 2, alpha 2 subunit
1611	DAP	death-associated protein
1612	DAPK1	death-associated protein kinase 1
1613	DAPK3	death-associated protein kinase 3
1616	DAXX	death-domain associated protein
161823	ADAL	adenosine deaminase-like
161931	ADAD2	adenosine deaminase domain containing 2
162	AP1B1	adaptor-related protein complex 1, beta 1 subunit
1621	DBH	dopamine beta-hydroxylase (dopamine beta-monooxygenase)
162394	SLFN5	schlafen family member 5
162989	DEDD2	death effector domain containing 2
1630	DCC	deleted in colorectal carcinoma
163126	EID2	EP300 interacting inhibitor of differentiation 2
1634	DCN	decorin

1636	ACE	angiotensin I converting enzyme (peptidyl-dipeptidase A) 1
163702	IL28RA	interleukin 28 receptor, alpha (interferon, lambda receptor)
164	AP1G1	adaptor-related protein complex 1, gamma 1 subunit
1646	AKR1C2	aldo-keto reductase family 1, member C2 (dihydrodiol dehydrogenase 2; bile acid binding protein; 3-alpha hydroxysteroid dehydrogenase, type III)
1647	GADD45A	growth arrest and DNA-damage-inducible, alpha
1649	DDIT3	DNA-damage-inducible transcript 3
165	AEBP1	AE binding protein 1
1650	DDOST	dolichyl-diphosphooligosaccharide--protein glycosyltransferase
165257	C1QL2	complement component 1, q subcomponent-like 2
165530	CLEC4F	C-type lectin domain family 4, member F
165904	XIRP1	xin actin-binding repeat containing 1
166	AES	amino-terminal enhancer of split
1667	DEFA1	defensin, alpha 1
166752	FREM3	FRAS1 related extracellular matrix 3
1668	DEFA3	defensin, alpha 3, neutrophil-specific
166824	RASSF6	Ras association (RalGDS/AF-6) domain family member 6
1669	DEFA4	defensin, alpha 4, corticostatin
1670	DEFA5	defensin, alpha 5, Paneth cell-specific
1671	DEFA6	defensin, alpha 6, Paneth cell-specific
1672	DEFB1	defensin, beta 1
1673	DEFB4A	defensin, beta 4A
1674	DES	desmin
1675	CFD	complement factor D (adipsin)
1676	DFFA	DNA fragmentation factor, 45kDa, alpha polypeptide
1677	DFFB	DNA fragmentation factor, 40kDa, beta polypeptide (caspase-activated DNase)
168507	PKD1L1	polycystic kidney disease 1 like 1
169	AF8T	AF8 temperature sensitivity complementing
170541	PSMD10P1	proteasome 26S subunit, non-ATPase, 10 pseudogene 1
170547	SCYE1P	small inducible cytokine subfamily E, member 1 (endothelial monocyte-activating) pseudogene
170682	SLEH1	systemic lupus erythematosus with hemolytic anemia 1
170949	DEFT1P	defensin, theta 1 pseudogene
171169	SPACA4	sperm acrosome associated 4
171392	ZNF675	zinc finger protein 675
1714	DGCR	DiGeorge syndrome chromosome region
171422	CRPP1	C-reactive protein pseudogene 1
171558	PTCRA	pre T-cell antigen receptor alpha

1718	DHCR24	24-dehydrocholesterol reductase
1723	DHODH	dihydroorotate dehydrogenase
1728	NQO1	NAD(P)H dehydrogenase, quinone 1
1730	DIAPH2	diaphanous homolog 2 (Drosophila)
1739	DLG1	discs, large homolog 1 (Drosophila)
1745	DLX1	distal-less homeobox 1
1755	DMBT1	deleted in malignant brain tumors 1
1756	DMD	dystrophin
1758	DMP1	dentin matrix acidic phosphoprotein 1
1759	DNM1	dynamin 1
176	ACAN	aggrecan
177	AGER	advanced glycosylation end product-specific receptor
1773	DNASE1	deoxyribonuclease I
1776	DNASE1L3	deoxyribonuclease I-like 3
1777	DNASE2	deoxyribonuclease II, lysosomal
1785	DNM2	dynamin 2
179	AGMX2	agammaglobulinemia, X-linked 2 (with growth hormone deficiency)
1793	DOCK1	dedicator of cytokinesis 1
1794	DOCK2	dedicator of cytokinesis 2
1795	DOCK3	dedicator of cytokinesis 3
1803	DPP4	dipeptidyl-peptidase 4
1805	DPT	dermatopontin
1812	DRD1	dopamine receptor D1
1813	DRD2	dopamine receptor D2
1814	DRD3	dopamine receptor D3
1815	DRD4	dopamine receptor D4
1816	DRD5	dopamine receptor D5
182	JAG1	jagged 1
1823	DSC1	desmocollin 1
1824	DSC2	desmocollin 2
1825	DSC3	desmocollin 3
1826	DSCAM	Down syndrome cell adhesion molecule
1828	DSG1	desmoglein 1
1829	DSG2	desmoglein 2
183	AGT	angiotensinogen (serpin peptidase inhibitor, clade A, member 8)
1830	DSG3	desmoglein 3
1831	TSC22D3	TSC22 domain family, member 3

1832	DSP	desmoplakin
1840	DTX1	deltex homolog 1 (Drosophila)
1842	ECM2	extracellular matrix protein 2, female organ and adipocyte specific
1843	DUSP1	dual specificity phosphatase 1
1844	DUSP2	dual specificity phosphatase 2
1845	DUSP3	dual specificity phosphatase 3
1848	DUSP6	dual specificity phosphatase 6
185	AGTR1	angiotensin II receptor, type 1
186	AGTR2	angiotensin II receptor, type 2
1869	E2F1	E2F transcription factor 1
1879	EBF1	early B-cell factor 1
1880	GPR183	G protein-coupled receptor 183
1888	EBVS1	Epstein Barr virus integration site 1
1889	ECE1	endothelin converting enzyme 1
1890	TYMP	thymidine phosphorylase
1894	ECT2	epithelial cell transforming sequence 2 oncogene
1896	EDA	ectodysplasin A
19	ABCA1	ATP-binding cassette, sub-family A (ABC1), member 1
1901	S1PR1	sphingosine-1-phosphate receptor 1
1902	LPAR1	lysophosphatidic acid receptor 1
1903	S1PR3	sphingosine-1-phosphate receptor 3
1906	EDN1	endothelin 1
1907	EDN2	endothelin 2
1908	EDN3	endothelin 3
1909	EDNRA	endothelin receptor type A
1910	EDNRB	endothelin receptor type B
1917	EEF1A2	eukaryotic translation elongation factor 1 alpha 2
192221	TLR7-like	toll-like receptor 7-like
192677	SLEN1	systemic lupus erythematosus with nephritis 1
192679	SLEN2	systemic lupus erythematosus with nephritis 2
192683	SCAMP5	secretory carrier membrane protein 5
1942	EFNA1	ephrin-A1
1947	EFNB1	ephrin-B1
1948	EFNB2	ephrin-B2
1950	EGF	epidermal growth factor
1951	CELSR3	cadherin, EGF LAG seven-pass G-type receptor 3 (flamingo homolog, Drosophila)
1952	CELSR2	cadherin, EGF LAG seven-pass G-type receptor 2 (flamingo homolog, Drosophila)

1956	EGFR	epidermal growth factor receptor
1958	EGR1	early growth response 1
1959	EGR2	early growth response 2
196	AHR	aryl hydrocarbon receptor
196074	METTL15	methyltransferase like 15
196264	MPZL3	myelin protein zero-like 3
196527	ANO6	anoctamin 6
1969	EPHA2	EPH receptor A2
197	AHSG	alpha-2-HS-glycoprotein
197131	UBR1	ubiquitin protein ligase E3 component n-recognin 1
197322	ACSF3	acyl-CoA synthetase family member 3
197350	CASP14L	putative caspase-14-like protein
197358	NLRC3	NLR family, CARD domain containing 3
1984	EIF5A	eukaryotic translation initiation factor 5A
199	AIF1	allograft inflammatory factor 1
1991	ELANE	elastase, neutrophil expressed
199699	DAND5	DAN domain family, member 5
1997	ELF1	E74-like factor 1 (ets domain transcription factor)
199731	CADM4	cell adhesion molecule 4
2	A2M	alpha-2-macroglobulin
2000	ELF4	E74-like factor 4 (ets domain transcription factor)
200010	SLC5A9	solute carrier family 5 (sodium/glucose cotransporter), member 9
200081	TXLNA	taxilin alpha
200172	SLFNL1	schlafen-like 1
200350	FOXD4L1	forkhead box D4-like 1
200576	PIKFYVE	phosphoinositide kinase, FYVE finger containing
2011	MARK2	MAP/microtubule affinity-regulating kinase 2
201161	CENPV	centromere protein V
201176	ARHGAP27	Rho GTPase activating protein 27
201294	UNC13D	unc-13 homolog D (C. elegans)
2015	EMR1	egf-like module containing, mucin-like, hormone receptor-like 1
201633	TIGIT	T cell immunoreceptor with Ig and ITIM domains
2021	ENDOG	endonuclease G
2022	ENG	endoglin
202309	GAPT	GRB2-binding adaptor protein, transmembrane
203068	TUBB	tubulin, beta
203190	LGI3	leucine-rich repeat LGI family, member 3

203245	NAIF1	nuclear apoptosis inducing factor 1
2033	EP300	E1A binding protein p300
2034	EPAS1	endothelial PAS domain protein 1
2042	EPHA3	EPH receptor A3
2045	EPHA7	EPH receptor A7
204777	IGLJCOR18	immunoglobulin lambda joining-constant/OR18 (pseudogene)
2054	STX2	syntaxin 2
2055	CLN8	ceroid-lipofuscinosis, neuronal 8 (epilepsy, progressive with mental retardation)
2056	EPO	erythropoietin
2057	EPOR	erythropoietin receptor
2064	ERBB2	v-erb-b2 erythroblastic leukemia viral oncogene homolog 2, neuro/glioblastoma derived oncogene homolog (avian)
2065	ERBB3	v-erb-b2 erythroblastic leukemia viral oncogene homolog 3 (avian)
2066	ERBB4	v-erb-a erythroblastic leukemia viral oncogene homolog 4 (avian)
2067	ERCC1	excision repair cross-complementing rodent repair deficiency, complementation group 1 (includes overlapping antisense sequence)
2068	ERCC2	excision repair cross-complementing rodent repair deficiency, complementation group 2
2069	EREG	epiregulin
207	AKT1	v-akt murine thymoma viral oncogene homolog 1
2071	ERCC3	excision repair cross-complementing rodent repair deficiency, complementation group 3 (xeroderma pigmentosum group B complementing)
2072	ERCC4	excision repair cross-complementing rodent repair deficiency, complementation group 4
2073	ERCC5	excision repair cross-complementing rodent repair deficiency, complementation group 5
2074	ERCC6	excision repair cross-complementing rodent repair deficiency, complementation group 6
2076	ERCM1	excision repair complementing defective repair in mouse cells
208	AKT2	v-akt murine thymoma viral oncogene homolog 2
2081	ERN1	endoplasmic reticulum to nucleus signaling 1
2100	ESR2	estrogen receptor 2 (ER beta)
2113	ETS1	v-ets erythroblastosis virus E26 oncogene homolog 1 (avian)
2122	MECOM	MDS1 and EVI1 complex locus
213	ALB	albumin
2134	EXTL1	exostoses (multiple)-like 1
2138	EYA1	eyes absent homolog 1 (Drosophila)
2139	EYA2	eyes absent homolog 2 (Drosophila)
214	ALCAM	activated leukocyte cell adhesion molecule
2147	F2	coagulation factor II (thrombin)
2149	F2R	coagulation factor II (thrombin) receptor

2150	F2RL1	coagulation factor II (thrombin) receptor-like 1
2151	F2RL2	coagulation factor II (thrombin) receptor-like 2
2152	F3	coagulation factor III (thromboplastin, tissue factor)
2153	F5	coagulation factor V (proaccelerin, labile factor)
2155	F7	coagulation factor VII (serum prothrombin conversion accelerator)
2157	F8	coagulation factor VIII, procoagulant component
2162	F13A1	coagulation factor XIII, A1 polypeptide
2167	FABP4	fatty acid binding protein 4, adipocyte
217	ALDH2	aldehyde dehydrogenase 2 family (mitochondrial)
2175	FANCA	Fanconi anemia, complementation group A
2176	FANCC	Fanconi anemia, complementation group C
2177	FANCD2	Fanconi anemia, complementation group D2
2178	FANCE	Fanconi anemia, complementation group E
2182	ACSL4	acyl-CoA synthetase long-chain family member 4
2185	PTK2B	PTK2B protein tyrosine kinase 2 beta
2187	FANCB	Fanconi anemia, complementation group B
2188	FANCF	Fanconi anemia, complementation group F
2189	FANCG	Fanconi anemia, complementation group G
2195	FAT1	FAT tumor suppressor homolog 1 (Drosophila)
2196	FAT2	FAT tumor suppressor homolog 2 (Drosophila)
219699	UNC5B	unc-5 homolog B (C. elegans)
219972	MPEG1	macrophage expressed 1
219988	PATL1	protein associated with topoisomerase II homolog 1 (yeast)
220	ALDH1A3	aldehyde dehydrogenase 1 family, member A3
2200	FBN1	fibrillin 1
220042	C11orf82	chromosome 11 open reading frame 82
220077	LOC220077	dedicator of cytokinesis 1 pseudogene
2201	FBN2	fibrillin 2
220107	DLEU7	deleted in lymphocytic leukemia, 7
220296	HEPACAM	hepatic and glial cell adhesion molecule
2204	FCAR	Fc fragment of IgA, receptor for
220441	RNF152	ring finger protein 152
2205	FCER1A	Fc fragment of IgE, high affinity I, receptor for; alpha polypeptide
2206	MS4A2	membrane-spanning 4-domains, subfamily A, member 2 (Fc fragment of IgE, high affinity I, receptor for; beta polypeptide)
2207	FCER1G	Fc fragment of IgE, high affinity I, receptor for; gamma polypeptide
2208	FCER2	Fc fragment of IgE, low affinity II, receptor for (CD23)

2209	FCGR1A	Fc fragment of IgG, high affinity Ia, receptor (CD64)
220972	MARCH8	membrane-associated ring finger (C3HC4) 8
2210	FCGR1B	Fc fragment of IgG, high affinity Ib, receptor (CD64)
2212	FCGR2A	Fc fragment of IgG, low affinity IIa, receptor (CD32)
2213	FCGR2B	Fc fragment of IgG, low affinity IIb, receptor (CD32)
2214	FCGR3A	Fc fragment of IgG, low affinity IIIa, receptor (CD16a)
221421	RSPH9	radial spoke head 9 homolog (Chlamydomonas)
221438	TREML2P1	triggering receptor expressed on myeloid cells-like 2 pseudogene 1
221472	FGD2	FYVE, RhoGEF and PH domain containing 2
2215	FCGR3B	Fc fragment of IgG, low affinity IIIb, receptor (CD16b)
2217	FCGRT	Fc fragment of IgG, receptor, transporter, alpha
2219	FCN1	ficolin (collagen/fibrinogen domain containing) 1
221935	SDK1	sidekick homolog 1, cell adhesion molecule (chicken)
221938	MMD2	monocyte to macrophage differentiation-associated 2
221955	DAGLB	diacylglycerol lipase, beta
2220	FCN2	ficolin (collagen/fibrinogen domain containing lectin) 2 (hucolin)
222256	CDHR3	cadherin-related family member 3
222344	LOC222344	TNF receptor-associated factor 6
222537	HS3ST5	heparan sulfate (glucosamine) 3-O-sulfotransferase 5
222967	RSPH10B	radial spoke head 10 homolog B (Chlamydomonas)
223117	SEMA3D	sema domain, immunoglobulin domain (Ig), short basic domain, secreted, (semaphorin) 3D
2241	FER	fer (fps/fes related) tyrosine kinase
2243	FGA	fibrinogen alpha chain
2244	FGB	fibrinogen beta chain
2245	FGD1	FYVE, RhoGEF and PH domain containing 1
2246	FGF1	fibroblast growth factor 1 (acidic)
2247	FGF2	fibroblast growth factor 2 (basic)
2248	FGF3	fibroblast growth factor 3
2249	FGF4	fibroblast growth factor 4
225	ABCD2	ATP-binding cassette, sub-family D (ALD), member 2
2252	FGF7	fibroblast growth factor 7
2255	FGF10	fibroblast growth factor 10
226	ALDOA	aldolase A, fructose-bisphosphate
2261	FGFR3	fibroblast growth factor receptor 3
2263	FGFR2	fibroblast growth factor receptor 2
2266	FGG	fibrinogen gamma chain
2268	FGR	Gardner-Rasheed feline sarcoma viral (v-fgr) oncogene homolog

2277	FIGF	c-fos induced growth factor (vascular endothelial growth factor D)
2278	FIM1	Friend-murine leukemia virus integration site 1 homolog
2279	FIM3	Friend-murine leukemia virus integration site 3 homolog
22795	NID2	nidogen 2 (osteonidogen)
22798	LAMB4	laminin, beta 4
2280	FKBP1A	FK506 binding protein 1A, 12kDa
22801	ITGA11	integrin, alpha 11
22806	IKZF3	IKAROS family zinc finger 3 (Aiolos)
22809	ATF5	activating transcription factor 5
2281	FKBP1B	FK506 binding protein 1B, 12.6 kDa
2282	FKBP1AP1	FK506 binding protein 1A, 12kDa pseudogene 1
22820	COPG	coatomer protein complex, subunit gamma
22822	PHLDA1	pleckstrin homology-like domain, family A, member 1
22827	PUF60	poly-U binding splicing factor 60KDa
22829	NLGN4Y	neuroligin 4, Y-linked
2283	FKBP1AP2	FK506 binding protein 1A, 12kDa pseudogene 2
2284	FKBP1AP3	FK506 binding protein 1A, 12kDa pseudogene 3
2285	FKBP1AP4	FK506 binding protein 1A, 12kDa pseudogene 4
22858	ICK	intestinal cell (MAK-like) kinase
2286	FKBP2	FK506 binding protein 2, 13kDa
22861	NLRP1	NLR family, pyrin domain containing 1
22862	FNDC3A	fibronectin type III domain containing 3A
22868	FASTKD2	FAST kinase domains 2
2287	FKBP3	FK506 binding protein 3, 25kDa
22871	NLGN1	neuroligin 1
2288	FKBP4	FK506 binding protein 4, 59kDa
22883	CLSTN1	calsyntenin 1
2289	FKBP5	FK506 binding protein 5
229	ALDOB	aldolase B, fructose-bisphosphate
22900	CARD8	caspase recruitment domain family, member 8
22904	SBNO2	strawberry notch homolog 2 (Drosophila)
22905	EPN2	epsin 2
22909	FAN1	FANCD2/FANCI-associated nuclease 1
22915	MMRN1	multimerin 1
22918	CD93	CD93 molecule
22920	KIFAP3	kinesin-associated protein 3
22925	PLA2R1	phospholipase A2 receptor 1, 180kDa

22927	HABP4	hyaluronan binding protein 4
22931	RAB18	RAB18, member RAS oncogene family
2294	FOXF1	forkhead box F1
22949	PTGR1	prostaglandin reductase 1
22954	TRIM32	tripartite motif containing 32
2296	FOXC1	forkhead box C1
2297	FOXD1	forkhead box D1
22974	TPX2	TPX2, microtubule-associated, homolog (Xenopus laevis)
22976	PAXIP1	PAX interacting (with transcription-activation domain) protein 1
2298	FOXD4	forkhead box D4
22983	MAST1	microtubule associated serine/threonine kinase 1
22984	PDCD11	programmed cell death 11
22985	ACIN1	apoptotic chromatin condensation inducer 1
22997	IGSF9B	immunoglobulin superfamily, member 9B
22999	RIMS1	regulating synaptic membrane exocytosis 1
230	ALDOC	aldolase C, fructose-bisphosphate
23012	STK38L	serine/threonine kinase 38 like
23017	FAIM2	Fas apoptotic inhibitory molecule 2
2302	FOXJ1	forkhead box J1
23025	UNC13A	unc-13 homolog A (C. elegans)
23028	KDM1A	lysine (K)-specific demethylase 1A
2303	FOXC2	forkhead box C2 (MFH-1, mesenchyme forkhead 1)
23032	USP33	ubiquitin specific peptidase 33
23037	PDZD2	PDZ domain containing 2
2304	FOXE1	forkhead box E1 (thyroid transcription factor 2)
23043	TNIK	TRAF2 and NCK interacting kinase
23048	FNBP1	formin binding protein 1
23054	NCOA6	nuclear receptor coactivator 6
2306	FOXD2	forkhead box D2
2307	FOXS1	forkhead box S1
23075	SWAP70	SWAP switching B-cell complex 70kDa subunit
2308	FOXO1	forkhead box O1
23085	ERC1	ELKS/RAB6-interacting/CAST family member 1
23087	TRIM35	tripartite motif containing 35
23089	PEG10	paternally expressed 10
2309	FOXO3	forkhead box O3
23095	KIF1B	kinesin family member 1B

23114	NFASC	neurofascin
23118	TAB2	TGF-beta activated kinase 1/MAP3K7 binding protein 2
23127	GLT25D2	glycosyltransferase 25 domain containing 2
2313	FLI1	Friend leukemia virus integration 1
23139	MAST2	microtubule associated serine/threonine kinase 2
23145	SSPO	SCO-spondin homolog (Bos taurus)
23149	FCHO1	FCH domain only 1
23151	GRAMD4	GRAM domain containing 4
23157	SEPT6	septin 6
2316	FLNA	filamin A, alpha
23166	STAB1	stabilin 1
23175	LPIN1	lipin 1
2319	FLOT2	flotillin 2
23197	FAF2	Fas associated factor family member 2
23198	PSME4	proteasome (prosome, macropain) activator subunit 4
23210	JMJD6	jumonji domain containing 6
23213	SULF1	sulfatase 1
2322	FLT3	fms-related tyrosine kinase 3
23229	ARHGEF9	Cdc42 guanine nucleotide exchange factor (GEF) 9
2323	FLT3LG	fms-related tyrosine kinase 3 ligand
23233	EXOC6B	exocyst complex component 6B
23235	SIK2	salt-inducible kinase 2
23236	PLCB1	phospholipase C, beta 1 (phosphoinositide-specific)
23237	ARC	activity-regulated cytoskeleton-associated protein
23239	PHLPP1	PH domain and leucine rich repeat protein phosphatase 1
23241	PACS2	phosphofurin acidic cluster sorting protein 2
23256	SCFD1	sec1 family domain containing 1
2326	FMO1	flavin containing monooxygenase 1
23263	MCF2L	MCF.2 cell line derived transforming sequence-like
23265	EXOC7	exocyst complex component 7
2327	FMO2	flavin containing monooxygenase 2 (non-functional)
23299	BICD2	bicaudal D homolog 2 (Drosophila)
23303	KIF13B	kinesin family member 13B
23307	FKBP15	FK506 binding protein 15, 133kDa
23308	ICOSLG	inducible T-cell co-stimulator ligand
2331	FMOD	fibromodulin
23318	ZCCHC11	zinc finger, CCHC domain containing 11

23327	NEDD4L	neural precursor cell expressed, developmentally down-regulated 4-like
23348	DOCK9	dedicator of cytokinesis 9
2335	FN1	fibronectin 1
23365	ARHGEF12	Rho guanine nucleotide exchange factor (GEF) 12
23368	PPP1R13B	protein phosphatase 1, regulatory (inhibitor) subunit 13B
23370	ARHGEF18	Rho/Rac guanine nucleotide exchange factor (GEF) 18
23385	NCSTN	nicastrin
2339	FNTA	farnesyltransferase, CAAX box, alpha
23392	KIAA0368	KIAA0368
23394	ADNP	activity-dependent neuroprotector homeobox
23401	FRAT2	frequently rearranged in advanced T-cell lymphomas 2
23406	COTL1	coactosin-like 1 (Dictyostelium)
23411	SIRT1	sirtuin 1
23413	NCS1	neuronal calcium sensor 1
23414	ZFPM2	zinc finger protein, multitype 2
2342	FNTB	farnesyltransferase, CAAX box, beta
23421	ITGB3BP	integrin beta 3 binding protein (beta3-endonexin)
23428	SLC7A8	solute carrier family 7 (amino acid transporter, L-type), member 8
23429	RYBP	RING1 and YY1 binding protein
23444	CDW12	CDw12 antigen
23447	CDW93	CDw93 antigen
23448	CD139	CD139 antigen
23449	CD165	CD165 antigen
23457	ABCB9	ATP-binding cassette, sub-family B (MDR/TAP), member 9
23461	ABCA5	ATP-binding cassette, sub-family A (ABC1), member 5
2348	FOLR1	folate receptor 1 (adult)
23484	LEPROTL1	leptin receptor overlapping transcript-like 1
23495	TNFRSF13B	tumor necrosis factor receptor superfamily, member 13B
23503	ZFYVE26	zinc finger, FYVE domain containing 26
23513	SCRIB	scribbled homolog (Drosophila)
23522	MYST4	MYST histone acetyltransferase (monocytic leukemia) 4
23526	HMHA1	histocompatibility (minor) HA-1
23529	CLCF1	cardiotrophin-like cytokine factor 1
2353	FOS	FBJ murine osteosarcoma viral oncogene homolog
23531	MMD	monocyte to macrophage differentiation-associated
23532	PRAME	preferentially expressed antigen in melanoma
23533	PIK3R5	phosphoinositide-3-kinase, regulatory subunit 5

23536	ADAT1	adenosine deaminase, tRNA-specific 1
23539	SLC16A8	solute carrier family 16, member 8 (monocarboxylic acid transporter 3)
23542	MAPK8IP2	mitogen-activated protein kinase 8 interacting protein 2
23547	LILRA4	leukocyte immunoglobulin-like receptor, subfamily A (with TM domain), member 4
23557	SNAPIN	SNAP-associated protein
23560	GTPBP4	GTP binding protein 4
23562	CLDN14	claudin 14
23564	DDAH2	dimethylarginine dimethylaminohydrolase 2
23567	ZNF346	zinc finger protein 346
2357	FPR1	formyl peptide receptor 1
2358	FPR2	formyl peptide receptor 2
23581	CASP14	caspase 14, apoptosis-related cysteine peptidase
23584	VSIG2	V-set and immunoglobulin domain containing 2
23586	DDX58	DEAD (Asp-Glu-Ala-Asp) box polypeptide 58
2359	FPR3	formyl peptide receptor 3
23591	C17orf88	chromosome 17 open reading frame 88
23592	LEMD3	LEM domain containing 3
23598	PATZ1	POZ (BTB) and AT hook containing zinc finger 1
23601	CLEC5A	C-type lectin domain family 5, member A
23603	CORO1C	coronin, actin binding protein, 1C
23604	DAPK2	death-associated protein kinase 2
23607	CD2AP	CD2-associated protein
23612	PHLDA3	pleckstrin homology-like domain, family A, member 3
23636	NUP62	nucleoporin 62kDa
23643	LY96	lymphocyte antigen 96
23645	PPP1R15A	protein phosphatase 1, regulatory (inhibitor) subunit 15A
23657	SLC7A11	solute carrier family 7, (cationic amino acid transporter, y+ system) member 11
23677	SH3BP4	SH3-domain binding protein 4
23678	SGK3	serum/glucocorticoid regulated kinase family, member 3
23683	PRKD3	protein kinase D3
23705	CADM1	cell adhesion molecule 1
23746	AIPL1	aryl hydrocarbon receptor interacting protein-like 1
23753	SDF2L1	stromal cell-derived factor 2-like 1
23759	PPIL2	peptidylprolyl isomerase (cyclophilin)-like 2
23765	IL17RA	interleukin 17 receptor A
23767	FLRT3	fibronectin leucine rich transmembrane protein 3
23768	FLRT2	fibronectin leucine rich transmembrane protein 2

23769	FLRT1	fibronectin leucine rich transmembrane protein 1
23770	FKBP8	FK506 binding protein 8, 38kDa
23772	CSF2RBP1	colony stimulating factor 2 receptor, beta, low-affinity (granulocyte-macrophage) pseudogene 1
23780	APOL2	apolipoprotein L, 2
23786	BCL2L13	BCL2-like 13 (apoptosis facilitator)
23787	MTCH1	mitochondrial carrier 1
238	ALK	anaplastic lymphoma receptor tyrosine kinase
239	ALOX12	arachidonate 12-lipoxygenase
2395	FXN	frataxin
24138	IFIT5	interferon-induced protein with tetratricopeptide repeats 5
24145	PANX1	pannexin 1
24146	CLDN15	claudin 15
2444	FRK	fyn-related kinase
245908	DEFB105A	defensin, beta 105A
245909	DEFB106A	defensin, beta 106A
245910	DEFB107A	defensin, beta 107A
245911	DEFB108B	defensin, beta 108B
245912	DEFB109P1	defensin, beta 109, pseudogene 1
245913	DEFB110	defensin, beta 110 locus
245915	DEFB112	defensin, beta 112
245927	DEFB113	defensin, beta 113
245928	DEFB114	defensin, beta 114
245929	DEFB115	defensin, beta 115
245930	DEFB116	defensin, beta 116
245931	DEFB117	defensin, beta 117
245932	DEFB119	defensin, beta 119
245934	DEFB121	defensin, beta 121
245935	DEFB122	defensin, beta 122 (pseudogene)
245936	DEFB123	defensin, beta 123
245937	DEFB124	defensin, beta 124
245938	DEFB125	defensin, beta 125
245939	DEFB128	defensin, beta 128
245940	DEFB130	defensin, beta 130
246210	IKBKGP1	inhibitor of kappa light polypeptide gene enhancer in B-cells, kinase gamma pseudogene 1
246283	TRBV22OR9-2	T cell receptor beta variable 22/OR9-2 (pseudogene)
246285	TRBV26OR9-2	T cell receptor beta variable 26/OR9-2 (pseudogene)
246286	TRBVAOR9-2	T cell receptor beta variable A/OR9-2 (pseudogene)

246750	MYAS1	Myasthenia gravis with thymus hyperplasia
246778	IL27	interleukin 27
247	ALOX15B	arachidonate 15-lipoxygenase, type B
2475	MTOR	mechanistic target of rapamycin (serine/threonine kinase)
2487	FRZB	frizzled-related protein
2488	FSHB	follicle stimulating hormone, beta polypeptide
249	ALPL	alkaline phosphatase, liver/bone/kidney
25	ABL1	c-abl oncogene 1, non-receptor tyrosine kinase
2515	ADAM2	ADAM metallopeptidase domain 2
2523	FUT1	fucosyltransferase 1 (galactoside 2-alpha-L-fucosyltransferase, H blood group)
2525	FUT3	fucosyltransferase 3 (galactoside 3(4)-L-fucosyltransferase, Lewis blood group)
2526	FUT4	fucosyltransferase 4 (alpha (1,3) fucosyltransferase, myeloid-specific)
2529	FUT7	fucosyltransferase 7 (alpha (1,3) fucosyltransferase)
252969	NEIL2	nei endonuclease VIII-like 2 (E. coli)
2530	FUT8	fucosyltransferase 8 (alpha (1,6) fucosyltransferase)
253018	HCG27	HLA complex group 27
2532	DARC	Duffy blood group, chemokine receptor
253260	RICTOR	RPTOR independent companion of MTOR, complex 2
2533	FYB	FYN binding protein
2534	FYN	FYN oncogene related to SRC, FGR, YES
253559	CADM2	cell adhesion molecule 2
2537	IFI6	interferon, alpha-inducible protein 6
253738	EBF3	early B-cell factor 3
2539	G6PD	glucose-6-phosphate dehydrogenase
254240	BPIL2	bactericidal/permeability-increasing protein-like 2
2543	GAGE1	G antigen 1
2547	XRCC6	X-ray repair complementing defective repair in Chinese hamster cells 6
255275	MYADML2	myeloid-associated differentiation marker-like 2
255488	RNF144B	ring finger protein 144B
255520	ELMOD2	ELMO/CED-12 domain containing 2
255631	COL24A1	collagen, type XXIV, alpha 1
255738	PCSK9	proprotein convertase subtilisin/kexin type 9
255743	NPNT	nephronectin
255877	BCL6B	B-cell CLL/lymphoma 6, member B
256076	COL6A5	collagen, type VI, alpha 5
256691	MAMDC2	MAM domain containing 2
257	ALX3	ALX homeobox 3

257093	PSME2P1	proteasome activator subunit 2 pseudogene 1
257144	GCET2	germinal center expressed transcript 2
257194	NEGR1	neuronal growth regulator 1
25776	CBY1	chibby homolog 1 (Drosophila)
25784	DGCR12	DiGeorge syndrome critical region gene 12
25786	DGCR11	DiGeorge syndrome critical region gene 11
25787	DGCR9	DiGeorge syndrome critical region gene 9
25791	NGEF	neuronal guanine nucleotide exchange factor
25794	FSCN2	fascin homolog 2, actin-bundling protein, retinal (Strongylocentrotus purpuratus)
258	AMBN	ameloblastin (enamel matrix protein)
25803	SPDEF	SAM pointed domain containing ets transcription factor
25805	BAMBI	BMP and activin membrane-bound inhibitor homolog (Xenopus laevis)
25816	TNFAIP8	tumor necrosis factor, alpha-induced protein 8
25817	FAM19A5	family with sequence similarity 19 (chemokine (C-C motif)-like), member A5
25824	PRDX5	peroxiredoxin 5
25836	NIPBL	Nipped-B homolog (Drosophila)
25837	RAB26	RAB26, member RAS oncogene family
25855	BRMS1	breast cancer metastasis suppressor 1
25865	PRKD2	protein kinase D2
25886	POC1A	POC1 centriolar protein homolog A (Chlamydomonas)
259	AMBP	alpha-1-microglobulin/bikunin precursor
2590	GALNT2	UDP-N-acetyl-alpha-D-galactosamine:polypeptide N-acetylgalactosaminyltransferase 2 (GalNAc-T2)
25909	AHCTF1	AT hook containing transcription factor 1
259197	NCR3	natural cytotoxicity triggering receptor 3
259215	LY6G6F	lymphocyte antigen 6 complex, locus G6F
259230	SGMS1	sphingomyelin synthase 1
259249	MRGPRX1	MAS-related GPR, member X1
259307	IL4I1	interleukin 4 induced 1
25937	WWTR1	WW domain containing transcription regulator 1
25945	PVRL3	poliovirus receptor-related 3
2596	GAP43	growth associated protein 43
25960	GPR124	G protein-coupled receptor 124
25966	C2CD2	C2 calcium-dependent domain containing 2
2597	GAPDH	glyceraldehyde-3-phosphate dehydrogenase
25975	EGFL6	EGF-like-domain, multiple 6
25976	TIPARP	TCDD-inducible poly(ADP-ribose) polymerase
25977	NECAP1	NECAP endocytosis associated 1

25998	IBTK	inhibitor of Bruton agammaglobulinemia tyrosine kinase
25999	CLIP3	CAP-GLY domain containing linker protein 3
26	ABP1	amiloride binding protein 1 (amine oxidase (copper-containing))
26013	L3MBTL1	l(3)mbt-like 1 (Drosophila)
26018	LRIG1	leucine-rich repeats and immunoglobulin-like domains 1
26020	LRP10	low density lipoprotein receptor-related protein 10
26025	PCDHGA12	protocadherin gamma subfamily A, 12
26032	SUSD5	sushi domain containing 5
260328	ALRH	Allergic rhinitis
260425	MAGI3	membrane associated guanylate kinase, WW and PDZ domain containing 3
260434	PYDC1	PYD (pyrin domain) containing 1
26046	LTN1	listerin E3 ubiquitin protein ligase 1
26047	CNTNAP2	contactin associated protein-like 2
26052	DNM3	dynamin 3
26060	APPL1	adaptor protein, phosphotyrosine interaction, PH domain and leucine zipper containing 1
26065	LSM14A	LSM14A, SCD6 homolog A (S. cerevisiae)
26103	LRIT1	leucine-rich repeat, immunoglobulin-like and transmembrane domains 1
26119	LDLRAP1	low density lipoprotein receptor adaptor protein 1
26123	TCTN3	tectonic family member 3
26130	GAPVD1	GTPase activating protein and VPS9 domains 1
26135	SERBP1	SERPINE1 mRNA binding protein 1
26145	IRF2BP1	interferon regulatory factor 2 binding protein 1
26146	TRAF3IP1	TNF receptor-associated factor 3 interacting protein 1
26160	IFT172	intraflagellar transport 172 homolog (Chlamydomonas)
26167	PCDHB5	protocadherin beta 5
261729	STEAP2	six transmembrane epithelial antigen of the prostate 2
261734	NPHP4	nephronophthisis 4
2619	GAS1	growth arrest-specific 1
26191	PTPN22	protein tyrosine phosphatase, non-receptor type 22 (lymphoid)
2620	GAS2	growth arrest-specific 2
2621	GAS6	growth arrest-specific 6
26220	DGCR5	DiGeorge syndrome critical region gene 5 (non-protein coding)
26222	DGCR10	DiGeorge syndrome critical region gene 10
2623	GATA1	GATA binding protein 1 (globin transcription factor 1)
26230	TIAM2	T-cell lymphoma invasion and metastasis 2
2624	GATA2	GATA binding protein 2
2625	GATA3	GATA binding protein 3

26258	PLDN	pallidin homolog (mouse)
2627	GATA6	GATA binding protein 6
26276	VPS33B	vacuolar protein sorting 33 homolog B (yeast)
26278	SACS	spastic ataxia of Charlevoix-Saguenay (sacsin)
26280	IL1RAPL2	interleukin 1 receptor accessory protein-like 2
26285	CLDN17	claudin 17
2633	GBP1	guanylate binding protein 1, interferon-inducible
2634	GBP2	guanylate binding protein 2, interferon-inducible
2637	GBX2	gastrulation brain homeobox 2
2642	GCGR	glucagon receptor
2643	GCH1	GTP cyclohydrolase 1
2647	BLOC1S1	biogenesis of lysosomal organelles complex-1, subunit 1
26471	NUPR1	nuclear protein, transcriptional regulator, 1
2648	KAT2A	K(lysine) acetyltransferase 2A
265	AMELX	amelogenin, X-linked
2651	GCNT2	glucosaminyl (N-acetyl) transferase 2, I-branching enzyme (I blood group)
26524	LATS2	LATS, large tumor suppressor, homolog 2 (Drosophila)
26525	IL36RN	interleukin 36 receptor antagonist
26548	ITGB1BP2	integrin beta 1 binding protein (melusin) 2
2657	GDF1	growth differentiation factor 1
26574	AATF	apoptosis antagonizing transcription factor
26579	MYEOV	myeloma overexpressed (in a subset of t(11;14) positive multiple myelomas)
2658	GDF2	growth differentiation factor 2
26585	GREM1	gremlin 1
26586	CKAP2	cytoskeleton associated protein 2
2660	MSTN	myostatin
2661	GDF9	growth differentiation factor 9
2662	GDF10	growth differentiation factor 10
266621	DGCR7	DiGeorge syndrome critical region gene 7
266683	LOC266683	dendritic cell protein pseudogene
266701	SOCS2P2	suppressor of cytokine signaling 2 pseudogene 2
266723	PSMC6P1	proteasome 26S subunit, ATPase, 6 pseudogene 1
266783	PSMD2P1	proteasome 26S subunit, non-ATPase, 2 pseudogene 1
2668	GDNF	glial cell derived neurotrophic factor
267013	HLA-DPA3	major histocompatibility complex, class II, DP alpha 3 (pseudogene)
267014	HLA-N	major histocompatibility complex, class I, N (pseudogene)
267015	HLA-S	major histocompatibility complex, class I, S (pseudogene)

267016	HLA-X	major histocompatibility complex, class I, X (pseudogene)
267017	HLA-Z	major histocompatibility complex, class I, Z (pseudogene)
2674	GFRA1	GDNF family receptor alpha 1
26762	HAVCR1	hepatitis A virus cellular receptor 1
268	AMH	anti-Mullerian hormone
2683	B4GALT1	UDP-Gal:betaGlcNAc beta 1,4- galactosyltransferase, polypeptide 1
269	AMHR2	anti-Mullerian hormone receptor, type II
2690	GHR	growth hormone receptor
2691	GHRH	growth hormone releasing hormone
2693	GHSR	growth hormone secretagogue receptor
26958	COPG2	coatomer protein complex, subunit gamma 2
2696	GIPR	gastric inhibitory polypeptide receptor
2697	GJA1	gap junction protein, alpha 1, 43kDa
26999	CYFIP2	cytoplasmic FMR1 interacting protein 2
27	ABL2	v-abl Abelson murine leukemia viral oncogene homolog 2
27002	PAFAH1B1P2	platelet-activating factor acetylhydrolase 1b, regulatory subunit 1 pseudogene 2
27004	TCL6	T-cell leukemia/lymphoma 6 (non-protein coding)
27018	NGFRAP1	nerve growth factor receptor (TNFRSF16) associated protein 1
27020	NPTN	neuroplastin
27022	FOXD3	forkhead box D3
27023	FOXB1	forkhead box B1
27032	ATP2C1	ATPase, Ca++ transporting, type 2C, member 1
27033	ZBTB32	zinc finger and BTB domain containing 32
27035	NOX1	NADPH oxidase 1
27036	SIGLEC7	sialic acid binding Ig-like lectin 7
27040	LAT	linker for activation of T cells
27069	GHITM	growth hormone inducible transmembrane protein
27086	FOXP1	forkhead box P1
27094	KCNMB3	potassium large conductance calcium-activated channel, subfamily M beta member 3
27113	BBC3	BCL2 binding component 3
27120	DKKL1	dickkopf-like 1
27128	CYTH4	cytohesin 4
27141	CIDEB	cell death-inducing DFFA-like effector b
27154	BRPF3	bromodomain and PHD finger containing, 3
27159	CHIA	chitinase, acidic
27170	COTL1P1	coactosin-like 1 (Dictyostelium) pseudogene 1
27177	IL36B	interleukin 36, beta

27178	IL37	interleukin 37
27179	IL36A	interleukin 36, alpha
27180	SIGLEC9	sialic acid binding Ig-like lectin 9
27181	SIGLEC8	sialic acid binding Ig-like lectin 8
27183	VPS4A	vacuolar protein sorting 4 homolog A (S. cerevisiae)
27189	IL17C	interleukin 17C
2719	GPC3	glypican 3
27190	IL17B	interleukin 17B
27202	GPR77	G protein-coupled receptor 77
27231	ITGB1BP3	integrin beta 1 binding protein 3
27237	ARHGEF16	Rho guanine nucleotide exchange factor (GEF) 16
27240	SIT1	signaling threshold regulating transmembrane adaptor 1
27241	BBS9	Bardet-Biedl syndrome 9
27242	TNFRSF21	tumor necrosis factor receptor superfamily, member 21
27250	PDCD4	programmed cell death 4 (neoplastic transformation inhibitor)
27252	KLHL20	kelch-like 20 (Drosophila)
27253	PCDH17	protocadherin 17
27255	CNTN6	contactin 6
27259	HPLH1	hemophagocytic lymphohistiocytosis 1
27283	TINAG	tubulointerstitial nephritis antigen
27286	SRPX2	sushi-repeat containing protein, X-linked 2
27289	RND1	Rho family GTPase 1
2729	GCLC	glutamate-cysteine ligase, catalytic subunit
27299	ADAMDEC1	ADAM-like, decysin 1
273	AMPH	amphiphysin
2730	GCLM	glutamate-cysteine ligase, modifier subunit
27302	BMP10	bone morphogenetic protein 10
27306	HPGDS	hematopoietic prostaglandin D synthase
27315	PGAP2	post-GPI attachment to proteins 2
27324	TOX3	TOX high mobility group box family member 3
27328	PCDH11X	protocadherin 11 X-linked
27329	ANGPTL3	angiopoietin-like 3
27330	RPS6KA6	ribosomal protein S6 kinase, 90kDa, polypeptide 6
27336	HTATSF1	HIV-1 Tat specific factor 1
2734	GLG1	golgi glycoprotein 1
27342	RABGEF1	RAB guanine nucleotide exchange factor (GEF) 1
27343	POLL	polymerase (DNA directed), lambda

27345	KCNMB4	potassium large conductance calcium-activated channel, subfamily M, beta member 4
2736	GLI2	GLI family zinc finger 2
2737	GLI3	GLI family zinc finger 3
2739	GLO1	glyoxalase I
274	BIN1	bridging integrator 1
27429	HTRA2	HtrA serine peptidase 2
27434	POLM	polymerase (DNA directed), mu
27443	CECR2	cat eye syndrome chromosome region, candidate 2
27445	PCLO	piccolo (presynaptic cytomatrix protein)
2757	GLYB	glycine B complementing
2762	GMDS	GDP-mannose 4,6-dehydratase
2765	GML	glycosylphosphatidylinositol anchored molecule like protein
2767	GNA11	guanine nucleotide binding protein (G protein), alpha 11 (Gq class)
276719	SOCS2P1	suppressor of cytokine signaling 2 pseudogene 1
276721	PSMD8P1	proteasome 26S subunit, non-ATPase, 8 pseudogene 1
2768	GNA12	guanine nucleotide binding protein (G protein) alpha 12
2769	GNA15	guanine nucleotide binding protein (G protein), alpha 15 (Gq class)
2770	GNAI1	guanine nucleotide binding protein (G protein), alpha inhibiting activity polypeptide 1
2771	GNAI2	guanine nucleotide binding protein (G protein), alpha inhibiting activity polypeptide 2
2773	GNAI3	guanine nucleotide binding protein (G protein), alpha inhibiting activity polypeptide 3
2775	GNAO1	guanine nucleotide binding protein (G protein), alpha activating activity polypeptide O
2776	GNAQ	guanine nucleotide binding protein (G protein), q polypeptide
2778	GNAS	GNAS complex locus
2782	GNB1	guanine nucleotide binding protein (G protein), beta polypeptide 1
2794	GNL1	guanine nucleotide binding protein-like 1
2796	GNRH1	gonadotropin-releasing hormone 1 (luteinizing-releasing hormone)
28	ABO	ABO blood group (transferase A, alpha 1-3-N-acetylgalactosaminyltransferase; transferase B, alpha 1-3-galactosyltransferase)
280637	PSMD7P1	proteasome (prosome, macropain) 26S subunit, non-ATPase, 7 pseudogene 1
280644	PSMD10P2	proteasome 26S subunit, non-ATPase, 10 pseudogene 2
280655	C14orf19	immunoglobulin (CD79A) binding protein 1 pseudogene
2810	SFN	stratifin
2811	GP1BA	glycoprotein Ib (platelet), alpha polypeptide
2812	GP1BB	glycoprotein Ib (platelet), beta polypeptide
2814	GP5	glycoprotein V (platelet)
2815	GP9	glycoprotein IX (platelet)
2821	GPI	glucose-6-phosphate isomerase

2826	CCR10	chemokine (C-C motif) receptor 10
282616	IL28A	interleukin 28A (interferon, lambda 2)
282617	IL28B	interleukin 28B (interferon, lambda 3)
282618	IL29	interleukin 29 (interferon, lambda 1)
282631	GCCD2	Glucocorticoid deficiency 2
282809	POC1B	POC1 centriolar protein homolog B (Chlamydomonas)
282849	SLEN3	systemic lupus erythematosus with nephritis 3
2829	XCR1	chemokine (C motif) receptor 1
282973	JAKMIP3	Janus kinase and microtubule interacting protein 3
28299	IGKV1-5	immunoglobulin kappa variable 1-5
282991	BLOC1S2	biogenesis of lysosomal organelles complex-1, subunit 2
28300	IGHV3OR16-16	immunoglobulin heavy variable 3/OR16-16 (pseudogene)
28301	IGHV3OR16-15	immunoglobulin heavy variable 3/OR16-15 (pseudogene)
28304	IGHV3OR16-12	immunoglobulin heavy variable 3/OR16-12 (non-functional)
28305	IGHV3OR16-11	immunoglobulin heavy variable 3/OR16-11 (pseudogene)
28306	IGHV3OR16-10	immunoglobulin heavy variable 3/OR16-10 (non-functional)
28307	IGHV3OR16-9	immunoglobulin heavy variable 3/OR16-9 (non-functional)
28309	IGHV3OR16-7	immunoglobulin heavy variable 3/OR16-7 (pseudogene)
28312	IGHV1OR16-4	immunoglobulin heavy variable 1/OR16-4 (pseudogene)
28313	IGHV1OR16-3	immunoglobulin heavy variable 1/OR16-3 (pseudogene)
28314	IGHV1OR16-2	immunoglobulin heavy variable 1/OR16-2 (pseudogene)
283149	BCL9L	B-cell CLL/lymphoma 9-like
28315	IGHV1OR16-1	immunoglobulin heavy variable 1/OR16-1 (pseudogene)
28316	CDH20	cadherin 20, type 2
28317	IGHV4OR15-8	immunoglobulin heavy variable 4/OR15-8 (non-functional)
28318	IGHV3OR15-7	immunoglobulin heavy variable 3/OR15-7 (pseudogene)
28320	IGHV1OR15-6	immunoglobulin heavy variable 1/OR15-6 (pseudogene)
28326	IGHD5OR15-5B	immunoglobulin heavy diversity 5/OR15-5B (non-functional)
28327	IGHD5OR15-5A	immunoglobulin heavy diversity 5/OR15-5A (non-functional)
28328	IGHD4OR15-4B	immunoglobulin heavy diversity 4/OR15-4B (non-functional)
283284	IGSF22	immunoglobulin superfamily, member 22
28329	IGHD4OR15-4A	immunoglobulin heavy diversity 4/OR15-4A (non-functional)
2833	CXCR3	chemokine (C-X-C motif) receptor 3
28330	IGHD3OR15-3B	immunoglobulin heavy diversity 3/OR15-3B (non-functional)
28331	IGHD3OR15-3A	immunoglobulin heavy diversity 3/OR15-3A (non-functional)
283316	CD163L1	CD163 molecule-like 1
28332	IGHD2OR15-2B	immunoglobulin heavy diversity 2/OR15-2B (non-functional)

28333	IGHD2OR15-2A	immunoglobulin heavy diversity 2/OR15-2A (non-functional)
28334	IGHD1OR15-1B	immunoglobulin heavy diversity 1/OR15-1B (non-functional)
28335	IGHD1OR15-1A	immunoglobulin heavy diversity 1/OR15-1A (non-functional)
28337	IGHVIV-44-1	immunoglobulin heavy variable (IV)-44-1 (pseudogene)
28338	IGHVIII-82	immunoglobulin heavy variable (III)-82 (pseudogene)
28339	IGHVIII-76-1	immunoglobulin heavy variable (III)-76-1 (pseudogene)
28340	IGHVIII-67-4	immunoglobulin heavy variable (III)-67-4 (pseudogene)
28341	IGHVIII-67-3	immunoglobulin heavy variable (III)-67-3 (pseudogene)
28342	IGHVIII-67-2	immunoglobulin heavy variable (III)-67-2 (pseudogene)
283420	CLEC9A	C-type lectin domain family 9, member A
28343	IGHVIII-51-1	immunoglobulin heavy variable (III)-51-1 (pseudogene)
28344	IGHVIII-47-1	immunoglobulin heavy variable (III)-47-1 (pseudogene)
28345	IGHVIII-44	immunoglobulin heavy variable (III)-44 (pseudogene)
28346	IGHVIII-38-1	immunoglobulin heavy variable (III)-38-1 (pseudogene)
28347	IGHVIII-26-1	immunoglobulin heavy variable (III)-26-1 (pseudogene)
28348	IGHVIII-25-1	immunoglobulin heavy variable (III)-25-1 (pseudogene)
28349	IGHVIII-22-2	immunoglobulin heavy variable (III)-22-2 (pseudogene)
28350	IGHVIII-16-1	immunoglobulin heavy variable (III)-16-1 (pseudogene)
28351	IGHVIII-13-1	immunoglobulin heavy variable (III)-13-1 (pseudogene)
28352	IGHVIII-11-1	immunoglobulin heavy variable (III)-11-1 (pseudogene)
28353	IGHVIII-5-2	immunoglobulin heavy variable (III)-5-2 (pseudogene)
28354	IGHVIII-5-1	immunoglobulin heavy variable (III)-5-1 (pseudogene)
28355	IGHVIII-2-1	immunoglobulin heavy variable (III)-2-1 (pseudogene)
28356	IGHVII-78-1	immunoglobulin heavy variable (II)-78-1 (pseudogene)
28357	IGHVII-74-1	immunoglobulin heavy variable (II)-74-1 (pseudogene)
28358	IGHVII-67-1	immunoglobulin heavy variable (II)-67-1 (pseudogene)
28359	IGHVII-65-1	immunoglobulin heavy variable (II)-65-1 (pseudogene)
28360	IGHVII-62-1	immunoglobulin heavy variable (II)-62-1 (pseudogene)
28361	IGHVII-60-1	immunoglobulin heavy variable (II)-60-1 (pseudogene)
28362	IGHVII-53-1	immunoglobulin heavy variable (II)-53-1 (pseudogene)
28363	IGHVII-51-2	immunoglobulin heavy variable (II)-51-2 (pseudogene)
28364	IGHVII-49-1	immunoglobulin heavy variable (II)-49-1 (pseudogene)
28365	IGHVII-46-1	immunoglobulin heavy variable (II)-46-1 (pseudogene)
28366	IGHVII-44-2	immunoglobulin heavy variable (II)-44-2 (pseudogene)
28367	IGHVII-43-1	immunoglobulin heavy variable (II)-43-1 (pseudogene)
28368	IGHVII-40-1	immunoglobulin heavy variable (II)-40-1 (pseudogene)
28369	IGHVII-33-1	immunoglobulin heavy variable (II)-33-1 (pseudogene)

28370	IGHVII-31-1	immunoglobulin heavy variable (II)-31-1 (pseudogene)
28371	IGHVII-30-1	immunoglobulin heavy variable (II)-30-1 (pseudogene)
28372	IGHVII-28-1	immunoglobulin heavy variable (II)-28-1 (pseudogene)
28373	IGHVII-26-2	immunoglobulin heavy variable (II)-26-2 (pseudogene)
28374	IGHVII-22-1	immunoglobulin heavy variable (II)-22-1 (pseudogene)
28375	IGHVII-20-1	immunoglobulin heavy variable (II)-20-1 (pseudogene)
28376	IGHVII-15-1	immunoglobulin heavy variable (II)-15-1 (pseudogene)
28377	IGHVII-1-1	immunoglobulin heavy variable (II)-1-1 (pseudogene)
28378	IGHV7-81	immunoglobulin heavy variable 7-81 (non-functional)
28380	IGHV7-56	immunoglobulin heavy variable 7-56 (pseudogene)
283804	LOC283804	disintegrin and metalloproteinase domain-containing protein 21-like
28381	IGHV7-40	immunoglobulin heavy variable 7-40 (pseudogene)
28382	IGHV7-34-1	immunoglobulin heavy variable 7-34-1 (pseudogene)
28383	IGHV7-27	immunoglobulin heavy variable 7-27 (pseudogene)
283849	EXOC3L1	exocyst complex component 3-like 1
28385	IGHV6-1	immunoglobulin heavy variable 6-1
28386	IGHV5-A	immunoglobulin heavy variable 5-A (provisional, gene/pseudogene)
28387	IGHV5-78	immunoglobulin heavy variable 5-78 (pseudogene)
28388	IGHV5-51	immunoglobulin heavy variable 5-51
28389	IGHV4-B	immunoglobulin heavy variable 4-B (provisional)
28390	IGHV4-80	immunoglobulin heavy variable 4-80 (pseudogene)
28391	IGHV4-61	immunoglobulin heavy variable 4-61
28392	IGHV4-59	immunoglobulin heavy variable 4-59
283927	NUDT7	nudix (nucleoside diphosphate linked moiety X)-type motif 7
28393	IGHV4-55	immunoglobulin heavy variable 4-55 (pseudogene)
28394	IGHV4-39	immunoglobulin heavy variable 4-39
28395	IGHV4-34	immunoglobulin heavy variable 4-34
28396	IGHV4-31	immunoglobulin heavy variable 4-31
28397	IGHV4-30-4	immunoglobulin heavy variable 4-30-4
28398	IGHV4-30-2	immunoglobulin heavy variable 4-30-2
28399	IGHV4-30-1	immunoglobulin heavy variable 4-30-1
284	ANGPT1	angiopoietin 1
2840	GPR17	G protein-coupled receptor 17
28400	IGHV4-28	immunoglobulin heavy variable 4-28
28401	IGHV4-4	immunoglobulin heavy variable 4-4
28402	IGHV3-H	immunoglobulin heavy variable 3-H (pseudogene)
28404	IGHV3-D	immunoglobulin heavy variable 3-D (provisional)

28405	IGHV3-79	immunoglobulin heavy variable 3-79 (pseudogene)
28406	IGHV3-76	immunoglobulin heavy variable 3-76 (pseudogene)
28407	IGHV3-75	immunoglobulin heavy variable 3-75 (pseudogene)
28408	IGHV3-74	immunoglobulin heavy variable 3-74
28409	IGHV3-73	immunoglobulin heavy variable 3-73
28410	IGHV3-72	immunoglobulin heavy variable 3-72
28411	IGHV3-71	immunoglobulin heavy variable 3-71 (pseudogene)
284110	GSDMA	gasdermin A
284114	TMEM102	transmembrane protein 102
28412	IGHV3-66	immunoglobulin heavy variable 3-66
28413	IGHV3-65	immunoglobulin heavy variable 3-65 (pseudogene)
28414	IGHV3-64	immunoglobulin heavy variable 3-64
28415	IGHV3-63	immunoglobulin heavy variable 3-63 (pseudogene)
28416	IGHV3-62	immunoglobulin heavy variable 3-62 (pseudogene)
28417	IGHV3-60	immunoglobulin heavy variable 3-60 (pseudogene)
28418	IGHV3-57	immunoglobulin heavy variable 3-57 (pseudogene)
28419	IGHV3-54	immunoglobulin heavy variable 3-54 (pseudogene)
28420	IGHV3-53	immunoglobulin heavy variable 3-53
28421	IGHV3-52	immunoglobulin heavy variable 3-52 (pseudogene)
284217	LAMA1	laminin, alpha 1
28422	IGHV3-50	immunoglobulin heavy variable 3-50 (pseudogene)
28423	IGHV3-49	immunoglobulin heavy variable 3-49
28424	IGHV3-48	immunoglobulin heavy variable 3-48
28425	IGHV3-47	immunoglobulin heavy variable 3-47 (pseudogene)
28426	IGHV3-43	immunoglobulin heavy variable 3-43
28427	IGHV3-42	immunoglobulin heavy variable 3-42 (pseudogene)
28428	IGHV3-41	immunoglobulin heavy variable 3-41 (pseudogene)
28429	IGHV3-38	immunoglobulin heavy variable 3-38 (non-functional)
284293	HMSD	histocompatibility (minor) serpin domain containing
28430	IGHV3-37	immunoglobulin heavy variable 3-37 (pseudogene)
28431	IGHV3-36	immunoglobulin heavy variable 3-36 (pseudogene)
28432	IGHV3-35	immunoglobulin heavy variable 3-35 (non-functional)
28433	IGHV3-33-2	immunoglobulin heavy variable 3-33-2 (pseudogene)
28434	IGHV3-33	immunoglobulin heavy variable 3-33
284340	CXCL17	chemokine (C-X-C motif) ligand 17
28435	IGHV3-32	immunoglobulin heavy variable 3-32 (pseudogene)
284359	IZUMO1	izumo sperm-egg fusion 1

28438	IGHV3-30-2	immunoglobulin heavy variable 3-30-2 (pseudogene)
28439	IGHV3-30	immunoglobulin heavy variable 3-30
28440	IGHV3-29	immunoglobulin heavy variable 3-29 (pseudogene)
28441	IGHV3-25	immunoglobulin heavy variable 3-25 (pseudogene)
28442	IGHV3-23	immunoglobulin heavy variable 3-23
28443	IGHV3-22	immunoglobulin heavy variable 3-22 (pseudogene)
28444	IGHV3-21	immunoglobulin heavy variable 3-21
28445	IGHV3-20	immunoglobulin heavy variable 3-20
28446	IGHV3-19	immunoglobulin heavy variable 3-19 (pseudogene)
284467	FAM19A3	family with sequence similarity 19 (chemokine (C-C motif)-like), member A3
28447	IGHV3-16	immunoglobulin heavy variable 3-16 (non-functional)
28448	IGHV3-15	immunoglobulin heavy variable 3-15
28449	IGHV3-13	immunoglobulin heavy variable 3-13
28450	IGHV3-11	immunoglobulin heavy variable 3-11 (gene/pseudogene)
28451	IGHV3-9	immunoglobulin heavy variable 3-9
28452	IGHV3-7	immunoglobulin heavy variable 3-7
28453	IGHV3-6	immunoglobulin heavy variable 3-6 (pseudogene)
28454	IGHV2-70	immunoglobulin heavy variable 2-70
28455	IGHV2-26	immunoglobulin heavy variable 2-26
28456	IGHV2-10	immunoglobulin heavy variable 2-10 (pseudogene)
28457	IGHV2-5	immunoglobulin heavy variable 2-5
28458	IGHV1-F	immunoglobulin heavy variable 1-F (provisional)
28460	IGHV1-C	immunoglobulin heavy variable 1-C (provisional, non-functional)
28461	IGHV1-69	immunoglobulin heavy variable 1-69
28462	IGHV1-68	immunoglobulin heavy variable 1-68 (pseudogene)
28463	IGHV1-67	immunoglobulin heavy variable 1-67 (pseudogene)
28464	IGHV1-58	immunoglobulin heavy variable 1-58
28465	IGHV1-46	immunoglobulin heavy variable 1-46
28466	IGHV1-45	immunoglobulin heavy variable 1-45
28467	IGHV1-24	immunoglobulin heavy variable 1-24
284672	PTGES3P	prostaglandin E synthase 3 (cytosolic) pseudogene
28468	IGHV1-18	immunoglobulin heavy variable 1-18
28469	IGHV1-17	immunoglobulin heavy variable 1-17 (pseudogene)
28470	IGHV1-14	immunoglobulin heavy variable 1-14 (pseudogene)
28471	IGHV1-12	immunoglobulin heavy variable 1-12 (pseudogene)
28472	IGHV1-8	immunoglobulin heavy variable 1-8
28473	IGHV1-3	immunoglobulin heavy variable 1-3

28474	IGHV1-2	immunoglobulin heavy variable 1-2
28475	IGHJ6	immunoglobulin heavy joining 6
28476	IGHJ5	immunoglobulin heavy joining 5
28477	IGHJ4	immunoglobulin heavy joining 4
28478	IGHJ3P	immunoglobulin heavy joining 3P (pseudogene)
28479	IGHJ3	immunoglobulin heavy joining 3
28480	IGHJ2P	immunoglobulin heavy joining 2P (pseudogene)
28481	IGHJ2	immunoglobulin heavy joining 2
28482	IGHJ1P	immunoglobulin heavy joining 1P (pseudogene)
28483	IGHJ1	immunoglobulin heavy joining 1
28484	IGHD7-27	immunoglobulin heavy diversity 7-27
28485	IGHD6-25	immunoglobulin heavy diversity 6-25
28486	IGHD6-19	immunoglobulin heavy diversity 6-19
28487	IGHD6-13	immunoglobulin heavy diversity 6-13
28488	IGHD6-6	immunoglobulin heavy diversity 6-6
28489	IGHD5-24	immunoglobulin heavy diversity 5-24 (non-functional)
28490	IGHD5-18	immunoglobulin heavy diversity 5-18
28491	IGHD5-12	immunoglobulin heavy diversity 5-12
28492	IGHD5-5	immunoglobulin heavy diversity 5-5
28493	IGHD4-23	immunoglobulin heavy diversity 4-23 (non-functional)
28494	IGHD4-17	immunoglobulin heavy diversity 4-17
28495	IGHD4-11	immunoglobulin heavy diversity 4-11 (non-functional)
28496	IGHD4-4	immunoglobulin heavy diversity 4-4
28497	IGHD3-22	immunoglobulin heavy diversity 3-22
28498	IGHD3-16	immunoglobulin heavy diversity 3-16
28499	IGHD3-10	immunoglobulin heavy diversity 3-10
285	ANGPT2	angiopoietin 2
28500	IGHD3-9	immunoglobulin heavy diversity 3-9
28501	IGHD3-3	immunoglobulin heavy diversity 3-3
28502	IGHD2-21	immunoglobulin heavy diversity 2-21
28503	IGHD2-15	immunoglobulin heavy diversity 2-15
28504	IGHD2-8	immunoglobulin heavy diversity 2-8
28505	IGHD2-2	immunoglobulin heavy diversity 2-2
28506	IGHD1-26	immunoglobulin heavy diversity 1-26
28507	IGHD1-20	immunoglobulin heavy diversity 1-20
28508	IGHD1-14	immunoglobulin heavy diversity 1-14 (non-functional)
28509	IGHD1-7	immunoglobulin heavy diversity 1-7

28510	IGHD1-1	immunoglobulin heavy diversity 1-1
28513	CDH19	cadherin 19, type 2
28514	DLL1	delta-like 1 (Drosophila)
28516	TRDV3	T cell receptor delta variable 3
28517	TRDV2	T cell receptor delta variable 2
28518	TRDV1	T cell receptor delta variable 1
28519	TRDJ4	T cell receptor delta joining 4
28520	TRDJ3	T cell receptor delta joining 3
28521	TRDJ2	T cell receptor delta joining 2
28522	TRDJ1	T cell receptor delta joining 1
28523	TRDD3	T cell receptor delta diversity 3
285237	C3orf38	chromosome 3 open reading frame 38
28524	TRDD2	T cell receptor delta diversity 2
28525	TRDD1	T cell receptor delta diversity 1
28526	TRDC	T cell receptor delta constant
285313	IGSF10	immunoglobulin superfamily, member 10
28552	TRBV23OR9-2	T cell receptor beta variable 23/OR9-2 (non-functional)
28555	TRBVB	T cell receptor beta variable B (pseudogene)
28556	TRBVA	T cell receptor beta variable A (pseudogene)
28557	TRBV30	T cell receptor beta variable 30 (gene/pseudogene)
28558	TRBV29-1	T cell receptor beta variable 29-1
28559	TRBV28	T cell receptor beta variable 28
285590	SH3PXD2B	SH3 and PX domains 2B
28560	TRBV27	T cell receptor beta variable 27
28561	TRBV26	T cell receptor beta variable 26 (pseudogene)
28562	TRBV25-1	T cell receptor beta variable 25-1
28563	TRBV24-1	T cell receptor beta variable 24-1
28564	TRBV23-1	T cell receptor beta variable 23-1 (non-functional)
28565	TRBV22-1	T cell receptor beta variable 22-1 (pseudogene)
28566	TRBV21-1	T cell receptor beta variable 21-1 (pseudogene)
28567	TRBV20-1	T cell receptor beta variable 20-1
28568	TRBV19	T cell receptor beta variable 19
28569	TRBV18	T cell receptor beta variable 18
28570	TRBV17	T cell receptor beta variable 17 (non-functional)
285704	RGMB	RGM domain family, member B
285706	LOC285706	cytokine receptor-like factor 3 pseudogene
28571	TRBV16	T cell receptor beta variable 16 (gene/pseudogene)

28572	TRBV15	T cell receptor beta variable 15
28573	TRBV14	T cell receptor beta variable 14
285737	CCRL1P1	chemokine (C-C motif) receptor-like 1 pseudogene
28574	TRBV13	T cell receptor beta variable 13
28575	TRBV12-5	T cell receptor beta variable 12-5
285755	PPIL6	peptidylprolyl isomerase (cyclophilin)-like 6
28576	TRBV12-4	T cell receptor beta variable 12-4
285761	DCBLD1	discoidin, CUB and LCCL domain containing 1
28577	TRBV12-3	T cell receptor beta variable 12-3
28578	TRBV12-2	T cell receptor beta variable 12-2 (pseudogene)
28579	TRBV12-1	T cell receptor beta variable 12-1 (pseudogene)
28580	TRBV11-3	T cell receptor beta variable 11-3
28581	TRBV11-2	T cell receptor beta variable 11-2
28582	TRBV11-1	T cell receptor beta variable 11-1
28583	TRBV10-3	T cell receptor beta variable 10-3
285830	HLA-F-AS1	HLA-F antisense RNA 1 (non-protein coding)
285834	HCG22	HLA complex group 22
28584	TRBV10-2	T cell receptor beta variable 10-2
28585	TRBV10-1	T cell receptor beta variable 10-1(gene/pseudogene)
285852	TREML4	triggering receptor expressed on myeloid cells-like 4
28586	TRBV9	T cell receptor beta variable 9
28587	TRBV8-2	T cell receptor beta variable 8-2 (pseudogene)
28588	TRBV8-1	T cell receptor beta variable 8-1 (pseudogene)
28589	TRBV7-9	T cell receptor beta variable 7-9
28590	TRBV7-8	T cell receptor beta variable 7-8
28591	TRBV7-7	T cell receptor beta variable 7-7
28592	TRBV7-6	T cell receptor beta variable 7-6
28593	TRBV7-5	T cell receptor beta variable 7-5 (pseudogene)
28594	TRBV7-4	T cell receptor beta variable 7-4 (gene/pseudogene)
28595	TRBV7-3	T cell receptor beta variable 7-3
28596	TRBV7-2	T cell receptor beta variable 7-2
28597	TRBV7-1	T cell receptor beta variable 7-1 (non-functional)
28598	TRBV6-9	T cell receptor beta variable 6-9
28599	TRBV6-8	T cell receptor beta variable 6-8
286	ANK1	ankyrin 1, erythrocytic
28600	TRBV6-7	T cell receptor beta variable 6-7 (non-functional)
28601	TRBV6-6	T cell receptor beta variable 6-6

28602	TRBV6-5	T cell receptor beta variable 6-5
28603	TRBV6-4	T cell receptor beta variable 6-4
28604	TRBV6-3	T cell receptor beta variable 6-3
286046	XKR6	XK, Kell blood group complex subunit-related family, member 6
28605	TRBV6-2	T cell receptor beta variable 6-2 (gene/pseudogene)
28606	TRBV6-1	T cell receptor beta variable 6-1
28607	TRBV5-8	T cell receptor beta variable 5-8
286076	BREA2	breast cancer estrogen-induced apoptosis 2
28608	TRBV5-7	T cell receptor beta variable 5-7 (non-functional)
28609	TRBV5-6	T cell receptor beta variable 5-6
28610	TRBV5-5	T cell receptor beta variable 5-5
28611	TRBV5-4	T cell receptor beta variable 5-4
28612	TRBV5-3	T cell receptor beta variable 5-3 (non-functional)
28613	TRBV5-2	T cell receptor beta variable 5-2 (pseudogene)
286133	SCARA5	scavenger receptor class A, member 5 (putative)
28614	TRBV5-1	T cell receptor beta variable 5-1
28615	TRBV4-3	T cell receptor beta variable 4-3
28616	TRBV4-2	T cell receptor beta variable 4-2
28617	TRBV4-1	T cell receptor beta variable 4-1
28618	TRBV3-2	T cell receptor beta variable 3-2 (pseudogene)
28619	TRBV3-1	T cell receptor beta variable 3-1
28620	TRBV2	T cell receptor beta variable 2
28621	TRBV1	T cell receptor beta variable 1 (pseudogene)
28622	TRBJ2-7	T cell receptor beta joining 2-7
28623	TRBJ2-6	T cell receptor beta joining 2-6
28624	TRBJ2-5	T cell receptor beta joining 2-5
28625	TRBJ2-4	T cell receptor beta joining 2-4
28626	TRBJ2-3	T cell receptor beta joining 2-3
28627	TRBJ2-2P	T cell receptor beta joining 2-2P (non-functional)
28628	TRBJ2-2	T cell receptor beta joining 2-2
28629	TRBJ2-1	T cell receptor beta joining 2-1
28630	TRBJ1-6	T cell receptor beta joining 1-6
28631	TRBJ1-5	T cell receptor beta joining 1-5
28632	TRBJ1-4	T cell receptor beta joining 1-4
28633	TRBJ1-3	T cell receptor beta joining 1-3
28634	TRBJ1-2	T cell receptor beta joining 1-2
28635	TRBJ1-1	T cell receptor beta joining 1-1

28636	TRBD2	T cell receptor beta diversity 2
28637	TRBD1	T cell receptor beta diversity 1
28638	TRBC2	T cell receptor beta constant 2
286380	FOXD4L3	forkhead box D4-like 3
28639	TRBC1	T cell receptor beta constant 1
28640	TRAV41	T cell receptor alpha variable 41
28641	TRAV40	T cell receptor alpha variable 40
28642	TRAV39	T cell receptor alpha variable 39
28643	TRAV38-2DV8	T cell receptor alpha variable 38-2/delta variable 8
28644	TRAV38-1	T cell receptor alpha variable 38-1
28645	TRAV37	T cell receptor alpha variable 37 (pseudogene)
28646	TRAV36DV7	T cell receptor alpha variable 36/delta variable 7
28647	TRAV35	T cell receptor alpha variable 35
28648	TRAV34	T cell receptor alpha variable 34
28649	TRAV33	T cell receptor alpha variable 33 (pseudogene)
28650	TRAV32	T cell receptor alpha variable 32 (pseudogene)
28651	TRAV31	T cell receptor alpha variable 31 (pseudogene)
28652	TRAV30	T cell receptor alpha variable 30
28653	TRAV29DV5	T cell receptor alpha variable 29/delta variable 5 (gene/pseudogene)
28654	TRAV28	T cell receptor alpha variable 28 (pseudogene)
28655	TRAV27	T cell receptor alpha variable 27
28656	TRAV26-2	T cell receptor alpha variable 26-2
28657	TRAV26-1	T cell receptor alpha variable 26-1
28658	TRAV25	T cell receptor alpha variable 25
28659	TRAV24	T cell receptor alpha variable 24
28660	TRAV23DV6	T cell receptor alpha variable 23/delta variable 6
28661	TRAV22	T cell receptor alpha variable 22
28662	TRAV21	T cell receptor alpha variable 21
28663	TRAV20	T cell receptor alpha variable 20
28664	TRAV19	T cell receptor alpha variable 19
28665	TRAV18	T cell receptor alpha variable 18
28666	TRAV17	T cell receptor alpha variable 17
28667	TRAV16	T cell receptor alpha variable 16
286676	ILDR1	immunoglobulin-like domain containing receptor 1
28668	TRAV15	T cell receptor alpha variable 15 (pseudogene)
28669	TRAV14DV4	T cell receptor alpha variable 14/delta variable 4
28670	TRAV13-2	T cell receptor alpha variable 13-2

28671	TRAV13-1	T cell receptor alpha variable 13-1
28672	TRAV12-3	T cell receptor alpha variable 12-3
28673	TRAV12-2	T cell receptor alpha variable 12-2
28674	TRAV12-1	T cell receptor alpha variable 12-1
28675	TRAV11	T cell receptor alpha variable 11 (pseudogene)
28676	TRAV10	T cell receptor alpha variable 10
28677	TRAV9-2	T cell receptor alpha variable 9-2
28678	TRAV9-1	T cell receptor alpha variable 9-1
28679	TRAV8-7	T cell receptor alpha variable 8-7 (non-functional)
28680	TRAV8-6	T cell receptor alpha variable 8-6
28681	TRAV8-5	T cell receptor alpha variable 8-5 (pseudogene)
28682	TRAV8-4	T cell receptor alpha variable 8-4
28683	TRAV8-3	T cell receptor alpha variable 8-3
28684	TRAV8-2	T cell receptor alpha variable 8-2
28685	TRAV8-1	T cell receptor alpha variable 8-1
28686	TRAV7	T cell receptor alpha variable 7
28688	TRAV5	T cell receptor alpha variable 5
28689	TRAV4	T cell receptor alpha variable 4
2869	GRK5	G protein-coupled receptor kinase 5
28690	TRAV3	T cell receptor alpha variable 3 (gene/pseudogene)
28691	TRAV2	T cell receptor alpha variable 2
28692	TRAV1-2	T cell receptor alpha variable 1-2
28693	TRAV1-1	T cell receptor alpha variable 1-1
28694	TRAJ61	T cell receptor alpha joining 61 (non-functional)
28695	TRAJ60	T cell receptor alpha joining 60 (pseudogene)
28696	TRAJ59	T cell receptor alpha joining 59 (non-functional)
28697	TRAJ58	T cell receptor alpha joining 58 (non-functional)
28698	TRAJ57	T cell receptor alpha joining 57
28699	TRAJ56	T cell receptor alpha joining 56
28700	TRAJ55	T cell receptor alpha joining 55 (pseudogene)
28701	TRAJ54	T cell receptor alpha joining 54
28702	TRAJ53	T cell receptor alpha joining 53
28703	TRAJ52	T cell receptor alpha joining 52
28704	TRAJ51	T cell receptor alpha joining 51 (pseudogene)
28705	TRAJ50	T cell receptor alpha joining 50
28706	TRAJ49	T cell receptor alpha joining 49
28707	TRAJ48	T cell receptor alpha joining 48

28708	TRAJ47	T cell receptor alpha joining 47
28709	TRAJ46	T cell receptor alpha joining 46
28710	TRAJ45	T cell receptor alpha joining 45
28711	TRAJ44	T cell receptor alpha joining 44
28712	TRAJ43	T cell receptor alpha joining 43
28713	TRAJ42	T cell receptor alpha joining 42
28714	TRAJ41	T cell receptor alpha joining 41
28715	TRAJ40	T cell receptor alpha joining 40
28716	TRAJ39	T cell receptor alpha joining 39
28717	TRAJ38	T cell receptor alpha joining 38
28718	TRAJ37	T cell receptor alpha joining 37
28719	TRAJ36	T cell receptor alpha joining 36
2872	MKNK2	MAP kinase interacting serine/threonine kinase 2
28720	TRAJ35	T cell receptor alpha joining 35 (non-functional)
28721	TRAJ34	T cell receptor alpha joining 34
28722	TRAJ33	T cell receptor alpha joining 33
28723	TRAJ32	T cell receptor alpha joining 32
28724	TRAJ31	T cell receptor alpha joining 31
28725	TRAJ30	T cell receptor alpha joining 30
28726	TRAJ29	T cell receptor alpha joining 29
28727	TRAJ28	T cell receptor alpha joining 28
28728	TRAJ27	T cell receptor alpha joining 27
28729	TRAJ26	T cell receptor alpha joining 26
28730	TRAJ25	T cell receptor alpha joining 25 (non-functional)
28731	TRAJ24	T cell receptor alpha joining 24
28732	TRAJ23	T cell receptor alpha joining 23
28733	TRAJ22	T cell receptor alpha joining 22
28734	TRAJ21	T cell receptor alpha joining 21
28735	TRAJ20	T cell receptor alpha joining 20
28736	TRAJ19	T cell receptor alpha joining 19 (non-functional)
28737	TRAJ18	T cell receptor alpha joining 18
28738	TRAJ17	T cell receptor alpha joining 17
28739	TRAJ16	T cell receptor alpha joining 16
28740	TRAJ15	T cell receptor alpha joining 15
28741	TRAJ14	T cell receptor alpha joining 14
28742	TRAJ13	T cell receptor alpha joining 13
28743	TRAJ12	T cell receptor alpha joining 12

28744	TRAJ11	T cell receptor alpha joining 11
28745	TRAJ10	T cell receptor alpha joining 10
28746	TRAJ9	T cell receptor alpha joining 9
28747	TRAJ8	T cell receptor alpha joining 8
28748	TRAJ7	T cell receptor alpha joining 7
28749	TRAJ6	T cell receptor alpha joining 6
28750	TRAJ5	T cell receptor alpha joining 5
28751	TRAJ4	T cell receptor alpha joining 4
28752	TRAJ3	T cell receptor alpha joining 3
28753	TRAJ2	T cell receptor alpha joining 2 (non-functional)
28754	TRAJ1	T cell receptor alpha joining 1 (non-functional)
28755	TRAC	T cell receptor alpha constant
28756	IGLV8OR8-1	immunoglobulin lambda variable 8/OR8-1 (pseudogene)
28757	IGLVV-66	immunoglobulin lambda variable (V)-66 (pseudogene)
28758	IGLVV-58	immunoglobulin lambda variable (V)-58 (pseudogene)
28759	IGLVIV-65	immunoglobulin lambda variable (IV)-65 (pseudogene)
2876	GPX1	glutathione peroxidase 1
28760	IGLVIV-64	immunoglobulin lambda variable (IV)-64 (pseudogene)
28761	IGLVIV-59	immunoglobulin lambda variable (IV)-59 (pseudogene)
28762	IGLVIV-53	immunoglobulin lambda variable (IV)-53 (pseudogene)
28763	IGLVI-70	immunoglobulin lambda variable (I)-70 (pseudogene)
28764	IGLVI-68	immunoglobulin lambda variable (I)-68 (pseudogene)
28765	IGLVI-63	immunoglobulin lambda variable (I)-63 (pseudogene)
28766	IGLVI-56	immunoglobulin lambda variable (I)-56 (pseudogene)
28767	IGLVI-42	immunoglobulin lambda variable (I)-42 (pseudogene)
28768	IGLVI-38	immunoglobulin lambda variable (I)-38 (pseudogene)
28769	IGLVI-20	immunoglobulin lambda variable (I)-20 (pseudogene)
28770	IGLV11-55	immunoglobulin lambda variable 11-55 (non-functional)
28771	IGLV10-67	immunoglobulin lambda variable 10-67 (pseudogene)
28772	IGLV10-54	immunoglobulin lambda variable 10-54
28773	IGLV9-49	immunoglobulin lambda variable 9-49
28774	IGLV8-61	immunoglobulin lambda variable 8-61
28775	IGLV7-46	immunoglobulin lambda variable 7-46 (gene/pseudogene)
28776	IGLV7-43	immunoglobulin lambda variable 7-43
28777	IGLV7-35	immunoglobulin lambda variable 7-35 (pseudogene)
28778	IGLV6-57	immunoglobulin lambda variable 6-57
28779	IGLV5-52	immunoglobulin lambda variable 5-52

28780	IGLV5-48	immunoglobulin lambda variable 5-48 (non-functional)
28781	IGLV5-45	immunoglobulin lambda variable 5-45
28782	IGLV5-39	immunoglobulin lambda variable 5-39
28783	IGLV5-37	immunoglobulin lambda variable 5-37
28784	IGLV4-69	immunoglobulin lambda variable 4-69
28785	IGLV4-60	immunoglobulin lambda variable 4-60
28786	IGLV4-3	immunoglobulin lambda variable 4-3
28787	IGLV3-32	immunoglobulin lambda variable 3-32 (non-functional)
28788	IGLV3-31	immunoglobulin lambda variable 3-31 (pseudogene)
28789	IGLV3-30	immunoglobulin lambda variable 3-30 (pseudogene)
28790	IGLV3-29	immunoglobulin lambda variable 3-29 (pseudogene)
28791	IGLV3-27	immunoglobulin lambda variable 3-27
28792	IGLV3-26	immunoglobulin lambda variable 3-26 (pseudogene)
28793	IGLV3-25	immunoglobulin lambda variable 3-25
28794	IGLV3-24	immunoglobulin lambda variable 3-24 (pseudogene)
28795	IGLV3-22	immunoglobulin lambda variable 3-22 (gene/pseudogene)
28796	IGLV3-21	immunoglobulin lambda variable 3-21
28797	IGLV3-19	immunoglobulin lambda variable 3-19
28798	IGLV3-17	immunoglobulin lambda variable 3-17 (pseudogene)
28799	IGLV3-16	immunoglobulin lambda variable 3-16
28800	IGLV3-15	immunoglobulin lambda variable 3-15 (pseudogene)
28801	IGLV3-13	immunoglobulin lambda variable 3-13 (pseudogene)
28802	IGLV3-12	immunoglobulin lambda variable 3-12
28803	IGLV3-10	immunoglobulin lambda variable 3-10
28804	IGLV3-9	immunoglobulin lambda variable 3-9 (gene/pseudogene)
28805	IGLV3-7	immunoglobulin lambda variable 3-7 (pseudogene)
28806	IGLV3-6	immunoglobulin lambda variable 3-6 (pseudogene)
28807	IGLV3-4	immunoglobulin lambda variable 3-4 (pseudogene)
28808	IGLV3-2	immunoglobulin lambda variable 3-2 (pseudogene)
28809	IGLV3-1	immunoglobulin lambda variable 3-1
28810	IGLV2-34	immunoglobulin lambda variable 2-34 (pseudogene)
28811	IGLV2-33	immunoglobulin lambda variable 2-33 (non-functional)
28812	IGLV2-28	immunoglobulin lambda variable 2-28 (pseudogene)
28813	IGLV2-23	immunoglobulin lambda variable 2-23
28814	IGLV2-18	immunoglobulin lambda variable 2-18
28815	IGLV2-14	immunoglobulin lambda variable 2-14
28816	IGLV2-11	immunoglobulin lambda variable 2-11

28817	IGLV2-8	immunoglobulin lambda variable 2-8
28818	IGLV2-5	immunoglobulin lambda variable 2-5 (pseudogene)
28819	IGLV1-62	immunoglobulin lambda variable 1-62 (pseudogene)
28820	IGLV1-51	immunoglobulin lambda variable 1-51
28821	IGLV1-50	immunoglobulin lambda variable 1-50 (non-functional)
28822	IGLV1-47	immunoglobulin lambda variable 1-47
28823	IGLV1-44	immunoglobulin lambda variable 1-44
28824	IGLV1-41	immunoglobulin lambda variable 1-41 (pseudogene)
28825	IGLV1-40	immunoglobulin lambda variable 1-40
28826	IGLV1-36	immunoglobulin lambda variable 1-36
28827	IGLJ7	immunoglobulin lambda joining 7
28828	IGLJ6	immunoglobulin lambda joining 6
28829	IGLJ5	immunoglobulin lambda joining 5 (non-functional)
28830	IGLJ4	immunoglobulin lambda joining 4 (non-functional)
28831	IGLJ3	immunoglobulin lambda joining 3
28832	IGLJ2	immunoglobulin lambda joining 2
28833	IGLJ1	immunoglobulin lambda joining 1
28834	IGLC7	immunoglobulin lambda constant 7
28847	IGKV2OR22-4	immunoglobulin kappa variable 2/OR22-4 (pseudogene)
2885	GRB2	growth factor receptor-bound protein 2
28850	IGKV1OR22-5	immunoglobulin kappa variable 1/OR22-5 (pseudogene)
28854	IGKV3OR2-5	immunoglobulin kappa variable 3/OR2-5 (pseudogene)
28855	IGKV2OR2-10	immunoglobulin kappa variable 2/OR2-10 (pseudogene)
28856	IGKV2OR2-8	immunoglobulin kappa variable 2/OR2-8 (pseudogene)
28858	IGKV2OR2-4	immunoglobulin kappa variable 2/OR2-4 (pseudogene)
28859	IGKV2OR2-2	immunoglobulin kappa variable 2/OR2-2 (pseudogene)
2886	GRB7	growth factor receptor-bound protein 7
28861	IGKV2OR2-1	immunoglobulin kappa variable 2/OR2-1 (pseudogene)
28862	IGKV1OR2-108	immunoglobulin kappa variable 1/OR2-108 (non-functional)
28863	IGKV1OR2-11	immunoglobulin kappa variable 1/OR2-11 (pseudogene)
28864	IGKV1OR2-9	immunoglobulin kappa variable 1/OR2-9 (pseudogene)
28865	IGKV1OR2-6	immunoglobulin kappa variable 1/OR2-6 (pseudogene)
28866	IGKV1OR2-3	immunoglobulin kappa variable 1/OR2-3 (pseudogene)
28867	IGKV1OR2-0	immunoglobulin kappa variable 1/OR2-0 (non-functional)
28869	IGKV6D-41	immunoglobulin kappa variable 6D-41 (non-functional)
28870	IGKV6D-21	immunoglobulin kappa variable 6D-21 (non-functional)
28871	IGKV3D-34	immunoglobulin kappa variable 3D-34 (pseudogene)

28872	IGKV3D-31	immunoglobulin kappa variable 3D-31 (pseudogene)
28873	IGKV3D-25	immunoglobulin kappa variable 3D-25 (pseudogene)
28874	IGKV3D-20	immunoglobulin kappa variable 3D-20
28875	IGKV3D-15	immunoglobulin kappa variable 3D-15 (gene/pseudogene)
28876	IGKV3D-11	immunoglobulin kappa variable 3D-11
28877	IGKV3D-7	immunoglobulin kappa variable 3D-7
28878	IGKV2D-40	immunoglobulin kappa variable 2D-40
28879	IGKV2D-38	immunoglobulin kappa variable 2D-38 (pseudogene)
2888	GRB14	growth factor receptor-bound protein 14
28880	IGKV2D-36	immunoglobulin kappa variable 2D-36 (pseudogene)
28881	IGKV2D-30	immunoglobulin kappa variable 2D-30
28882	IGKV2D-29	immunoglobulin kappa variable 2D-29
28883	IGKV2D-28	immunoglobulin kappa variable 2D-28
28884	IGKV2D-26	immunoglobulin kappa variable 2D-26
28885	IGKV2D-24	immunoglobulin kappa variable 2D-24 (non-functional)
28886	IGKV2D-23	immunoglobulin kappa variable 2D-23 (pseudogene)
28887	IGKV2D-19	immunoglobulin kappa variable 2D-19 (pseudogene)
28888	IGKV2D-18	immunoglobulin kappa variable 2D-18 (pseudogene)
28889	IGKV2D-14	immunoglobulin kappa variable 2D-14 (pseudogene)
28890	IGKV2D-10	immunoglobulin kappa variable 2D-10 (pseudogene)
28891	IGKV1D-43	immunoglobulin kappa variable 1D-43
28892	IGKV1D-42	immunoglobulin kappa variable 1D-42 (non-functional)
28893	IGKV1D-39	immunoglobulin kappa variable 1D-39
28894	IGKV1D-37	immunoglobulin kappa variable 1D-37 (non-functional)
28895	IGKV1D-35	immunoglobulin kappa variable 1D-35 (pseudogene)
28896	IGKV1D-33	immunoglobulin kappa variable 1D-33
28897	IGKV1D-32	immunoglobulin kappa variable 1D-32 (pseudogene)
28898	IGKV1D-27	immunoglobulin kappa variable 1D-27 (pseudogene)
28899	IGKV1D-22	immunoglobulin kappa variable 1D-22 (pseudogene)
28900	IGKV1D-17	immunoglobulin kappa variable 1D-17
28901	IGKV1D-16	immunoglobulin kappa variable 1D-16
28902	IGKV1D-13	immunoglobulin kappa variable 1D-13
28903	IGKV1D-12	immunoglobulin kappa variable 1D-12
28904	IGKV1D-8	immunoglobulin kappa variable 1D-8
28905	IGKV7-3	immunoglobulin kappa variable 7-3 (pseudogene)
28906	IGKV6-21	immunoglobulin kappa variable 6-21 (non-functional)
28907	IGKV5-2	immunoglobulin kappa variable 5-2

28908	IGKV4-1	immunoglobulin kappa variable 4-1
28909	IGKV3-34	immunoglobulin kappa variable 3-34 (pseudogene)
28910	IGKV3-31	immunoglobulin kappa variable 3-31 (pseudogene)
28911	IGKV3-25	immunoglobulin kappa variable 3-25 (pseudogene)
28912	IGKV3-20	immunoglobulin kappa variable 3-20
28913	IGKV3-15	immunoglobulin kappa variable 3-15
28914	IGKV3-11	immunoglobulin kappa variable 3-11
28915	IGKV3-7	immunoglobulin kappa variable 3-7 (non-functional)
28916	IGKV2-40	immunoglobulin kappa variable 2-40
28917	IGKV2-38	immunoglobulin kappa variable 2-38 (pseudogene)
28918	IGKV2-36	immunoglobulin kappa variable 2-36 (pseudogene)
28919	IGKV2-30	immunoglobulin kappa variable 2-30
28920	IGKV2-29	immunoglobulin kappa variable 2-29 (gene/pseudogene)
28921	IGKV2-28	immunoglobulin kappa variable 2-28
28922	IGKV2-26	immunoglobulin kappa variable 2-26 (pseudogene)
28923	IGKV2-24	immunoglobulin kappa variable 2-24
28924	IGKV2-23	immunoglobulin kappa variable 2-23 (pseudogene)
28925	IGKV2-19	immunoglobulin kappa variable 2-19 (pseudogene)
28926	IGKV2-18	immunoglobulin kappa variable 2-18 (pseudogene)
28927	IGKV2-14	immunoglobulin kappa variable 2-14 (pseudogene)
28928	IGKV2-10	immunoglobulin kappa variable 2-10 (pseudogene)
28929	IGKV2-4	immunoglobulin kappa variable 2-4 (pseudogene)
28930	IGKV1-39	immunoglobulin kappa variable 1-39 (gene/pseudogene)
28931	IGKV1-37	immunoglobulin kappa variable 1-37 (non-functional)
28932	IGKV1-35	immunoglobulin kappa variable 1-35 (pseudogene)
28933	IGKV1-33	immunoglobulin kappa variable 1-33
28934	IGKV1-32	immunoglobulin kappa variable 1-32 (pseudogene)
28935	IGKV1-27	immunoglobulin kappa variable 1-27
28936	IGKV1-22	immunoglobulin kappa variable 1-22 (pseudogene)
28937	IGKV1-17	immunoglobulin kappa variable 1-17
28938	IGKV1-16	immunoglobulin kappa variable 1-16
28939	IGKV1-13	immunoglobulin kappa variable 1-13 (gene/pseudogene)
28940	IGKV1-12	immunoglobulin kappa variable 1-12
28941	IGKV1-9	immunoglobulin kappa variable 1-9
28942	IGKV1-8	immunoglobulin kappa variable 1-8
28943	IGKV1-6	immunoglobulin kappa variable 1-6
28946	IGKJ5	immunoglobulin kappa joining 5

28947	IGKJ4	immunoglobulin kappa joining 4
28948	IGKJ3	immunoglobulin kappa joining 3
28949	IGKJ2	immunoglobulin kappa joining 2
28950	IGKJ1	immunoglobulin kappa joining 1
28951	TRIB2	tribbles homolog 2 (Drosophila)
2896	GRN	granulin
2898	GRIK2	glutamate receptor, ionotropic, kainate 2
28981	IFT81	intraflagellar transport 81 homolog (Chlamydomonas)
28982	FLVCR1	feline leukemia virus subgroup C cellular receptor 1
28985	MCTS1	malignant T cell amplified sequence 1
28986	MAGEH1	melanoma antigen family H, 1
28988	DBNL	drebrin-like
28989	METTL11A	methyltransferase like 11A
28996	HIPK2	homeodomain interacting protein kinase 2
29	ABR	active BCR-related gene
2902	GRIN1	glutamate receptor, ionotropic, N-methyl D-aspartate 1
2903	GRIN2A	glutamate receptor, ionotropic, N-methyl D-aspartate 2A
2904	GRIN2B	glutamate receptor, ionotropic, N-methyl D-aspartate 2B
29087	THYN1	thymocyte nuclear protein 1
29106	SCG3	secretogranin III
29108	PYCARD	PYD and CARD domain containing
29110	TBK1	TANK-binding kinase 1
29115	SAP30BP	SAP30 binding protein
29119	CTNNA3	catenin (cadherin-associated protein), alpha 3
29126	CD274	CD274 molecule
29127	RACGAP1	Rac GTPase activating protein 1
2915	GRM5	glutamate receptor, metabotropic 5
2919	CXCL1	chemokine (C-X-C motif) ligand 1 (melanoma growth stimulating activity, alpha)
2920	CXCL2	chemokine (C-X-C motif) ligand 2
2921	CXCL3	chemokine (C-X-C motif) ligand 3
2923	PDIA3	protein disulfide isomerase family A, member 3
293	SLC25A6	solute carrier family 25 (mitochondrial carrier; adenine nucleotide translocator), member 6
2932	GSK3B	glycogen synthase kinase 3 beta
2934	GSN	gelsolin
2935	GSPT1	G1 to S phase transition 1
2937	GSS	glutathione synthetase
2950	GSTP1	glutathione S-transferase pi 1

2956	MSH6	mutS homolog 6 (E. coli)
29760	BLNK	B-cell linker
29763	PACSIN3	protein kinase C and casein kinase substrate in neurons 3
2977	GUCY1A2	guanylate cyclase 1, soluble, alpha 2
29775	CARD10	caspase recruitment domain family, member 10
29780	PARVB	parvin, beta
29802	VPREB3	pre-B lymphocyte 3
2982	GUCY1A3	guanylate cyclase 1, soluble, alpha 3
2983	GUCY1B3	guanylate cyclase 1, soluble, beta 3
29843	SENP1	SUMO1/sentrin specific peptidase 1
29844	TFPT	TCF3 (E2A) fusion partner (in childhood Leukemia)
29851	ICOS	inducible T-cell co-stimulator
29895	MYLPF	myosin light chain, phosphorylatable, fast skeletal muscle
29911	HOOK2	hook homolog 2 (Drosophila)
29915	HCFC2	host cell factor C2
29924	EPN1	epsin 1
29927	SEC61A1	Sec61 alpha 1 subunit (S. cerevisiae)
2993	GYPA	glycophorin A (MNS blood group)
29930	PCDHB1	protocadherin beta 1
2994	GYPB	glycophorin B (MNS blood group)
29941	PKN3	protein kinase N3
29944	PNMA3	paraneoplastic antigen MA3
29949	IL19	interleukin 19
2995	GYPC	glycophorin C (Gerbich blood group)
2996	GYPE	glycophorin E (MNS blood group)
29965	C16orf5	chromosome 16 open reading frame 5
29967	LRP12	low density lipoprotein receptor-related protein 12
29974	A1CF	APOBEC1 complementation factor
29979	UBQLN1	ubiquilin 1
2999	GZMH	granzyme H (cathepsin G-like 2, protein h-CCPX)
29993	PACSIN1	protein kinase C and casein kinase substrate in neurons 1
29999	FSCN3	fascin homolog 3, actin-bundling protein, testicular (Strongylocentrotus purpuratus)
30001	ERO1L	ERO1-like (S. cerevisiae)
30009	TBX21	T-box 21
3001	GZMA	granzyme A (granzyme 1, cytotoxic T-lymphocyte-associated serine esterase 3)
30011	SH3KBP1	SH3-domain kinase binding protein 1
30012	TLX3	T-cell leukemia homeobox 3

3002	GZMB	granzyme B (granzyme 2, cytotoxic T-lymphocyte-associated serine esterase 1)
3004	GZMM	granzyme M (lymphocyte met-ase 1)
3005	H1F0	H1 histone family, member 0
301	ANXA1	annexin A1
3026	HABP2	hyaluronan binding protein 2
3036	HAS1	hyaluronan synthase 1
3053	SERPIND1	serpin peptidase inhibitor, clade D (heparin cofactor), member 1
3054	HCFC1	host cell factor C1 (VP16-accessory protein)
3055	HCK	hemopoietic cell kinase
306	ANXA3	annexin A3
3060	HCRT	hypocretin (orexin) neuropeptide precursor
3064	HTT	huntingtin
3065	HDAC1	histone deacetylase 1
3066	HDAC2	histone deacetylase 2
3067	HDC	histidine decarboxylase
307	ANXA4	annexin A4
3070	HELLS	helicase, lymphoid-specific
3071	NCKAP1L	NCK-associated protein 1-like
3075	CFH	complement factor H
3077	HFE	hemochromatosis
3078	CFHR1	complement factor H-related 1
308	ANXA5	annexin A5
3080	CFHR2	complement factor H-related 2
30812	SOX8	SRY (sex determining region Y)-box 8
30817	EMR2	egf-like module containing, mucin-like, hormone receptor-like 2
30818	KCNIP3	Kv channel interacting protein 3, calsenilin
3082	HGF	hepatocyte growth factor (hepapoietin A; scatter factor)
30835	CD209	CD209 molecule
30837	SOCS7	suppressor of cytokine signaling 7
3084	NRG1	neuregulin 1
30844	EHD4	EH-domain containing 4
30846	EHD2	EH-domain containing 2
30851	TAX1BP3	Tax1 (human T-cell leukemia virus type I) binding protein 3
3087	HHEX	hematopoietically expressed homeobox
3091	HIF1A	hypoxia inducible factor 1, alpha subunit (basic helix-loop-helix transcription factor)
3092	HIP1	huntingtin interacting protein 1
3094	HINT1	histidine triad nucleotide binding protein 1

3096	HIVEP1	human immunodeficiency virus type I enhancer binding protein 1
3097	HIVEP2	human immunodeficiency virus type I enhancer binding protein 2
3105	HLA-A	major histocompatibility complex, class I, A
3106	HLA-B	major histocompatibility complex, class I, B
3107	HLA-C	major histocompatibility complex, class I, C
3108	HLA-DMA	major histocompatibility complex, class II, DM alpha
3109	HLA-DMB	major histocompatibility complex, class II, DM beta
311	ANXA11	annexin A11
3111	HLA-DOA	major histocompatibility complex, class II, DO alpha
3112	HLA-DOB	major histocompatibility complex, class II, DO beta
3113	HLA-DPA1	major histocompatibility complex, class II, DP alpha 1
3115	HLA-DPB1	major histocompatibility complex, class II, DP beta 1
3116	HLA-DPB2	major histocompatibility complex, class II, DP beta 2 (pseudogene)
3117	HLA-DQA1	major histocompatibility complex, class II, DQ alpha 1
3118	HLA-DQA2	major histocompatibility complex, class II, DQ alpha 2
3119	HLA-DQB1	major histocompatibility complex, class II, DQ beta 1
3120	HLA-DQB2	major histocompatibility complex, class II, DQ beta 2
3121	HLA-DQB3	major histocompatibility complex, class II, DQ beta 3
3122	HLA-DRA	major histocompatibility complex, class II, DR alpha
3123	HLA-DRB1	major histocompatibility complex, class II, DR beta 1
3124	HLA-DRB2	major histocompatibility complex, class II, DR beta 2 (pseudogene)
3125	HLA-DRB3	major histocompatibility complex, class II, DR beta 3
3126	HLA-DRB4	major histocompatibility complex, class II, DR beta 4
3127	HLA-DRB5	major histocompatibility complex, class II, DR beta 5
3128	HLA-DRB6	major histocompatibility complex, class II, DR beta 6 (pseudogene)
3129	HLA-DRB7	major histocompatibility complex, class II, DR beta 7 (pseudogene)
3130	HLA-DRB8	major histocompatibility complex, class II, DR beta 8 (pseudogene)
3131	HLF	hepatic leukemia factor
3132	HLA-DRB9	major histocompatibility complex, class II, DR beta 9 (pseudogene)
3133	HLA-E	major histocompatibility complex, class I, E
3134	HLA-F	major histocompatibility complex, class I, F
3135	HLA-G	major histocompatibility complex, class I, G
3136	HLA-H	major histocompatibility complex, class I, H (pseudogene)
3137	HLA-J	major histocompatibility complex, class I, J (pseudogene)
3138	HLA-K	major histocompatibility complex, class I, K (pseudogene)
3139	HLA-L	major histocompatibility complex, class I, L, pseudogene
3140	MR1	major histocompatibility complex, class I-related

3146	HMGB1	high mobility group box 1
3148	HMGB2	high mobility group box 2
3158	HMGCS2	3-hydroxy-3-methylglutaryl-CoA synthase 2 (mitochondrial)
3162	HMOX1	heme oxygenase (decycling) 1
3164	NR4A1	nuclear receptor subfamily 4, group A, member 1
317	APAF1	apoptotic peptidase activating factor 1
3170	FOXA2	forkhead box A2
3175	ONECUT1	one cut homeobox 1
3176	HNMT	histamine N-methyltransferase
317753	PSMD12P	proteasome 26S subunit, non-ATPase, 12 pseudogene
317760	C14orf55	metalloproteinase-disintegrin 1-2 pseudogene
318	NUDT2	nudix (nucleoside diphosphate linked moiety X)-type motif 2
3190	HNRNPK	heterogeneous nuclear ribonucleoprotein K
3195	TLX1	T-cell leukemia homeobox 1
3196	TLX2	T-cell leukemia homeobox 2
320	APBA1	amyloid beta (A4) precursor protein-binding, family A, member 1
3200	HOXA3	homeobox A3
3202	HOXA5	homeobox A5
3204	HOXA7	homeobox A7
3209	HOXA13	homeobox A13
3214	HOXB4	homeobox B4
3217	HOXB7	homeobox B7
3219	HOXB9	homeobox B9
322	APBB1	amyloid beta (A4) precursor protein-binding, family B, member 1 (Fe65)
324	APC	adenomatous polyposis coli
3248	HPGD	hydroxyprostaglandin dehydrogenase 15-(NAD)
325	APCS	amyloid P component, serum
3251	HPRT1	hypoxanthine phosphoribosyltransferase 1
326	AIRE	autoimmune regulator
3263	HPX	hemopexin
3265	HRAS	v-Ha-ras Harvey rat sarcoma viral oncogene homolog
326617	PSMA3P	proteasome (prosome, macropain) subunit, alpha type, 3 pseudogene
3269	HRH1	histamine receptor H1
3273	HRG	histidine-rich glycoprotein
3274	HRH2	histamine receptor H2
3275	PRMT2	protein arginine methyltransferase 2
328	APEX1	APEX nuclease (multifunctional DNA repair enzyme) 1

3280	HES1	hairy and enhancer of split 1, (Drosophila)
329	BIRC2	baculoviral IAP repeat containing 2
3297	HSF1	heat shock transcription factor 1
330	BIRC3	baculoviral IAP repeat containing 3
3300	DNAJB2	DnaJ (Hsp40) homolog, subfamily B, member 2
3304	HSPA1B	heat shock 70kDa protein 1B
3309	HSPA5	heat shock 70kDa protein 5 (glucose-regulated protein, 78kDa)
331	XIAP	X-linked inhibitor of apoptosis
3313	HSPA9	heat shock 70kDa protein 9 (mortalin)
3315	HSPB1	heat shock 27kDa protein 1
332	BIRC5	baculoviral IAP repeat containing 5
3321	IGSF3	immunoglobulin superfamily, member 3
3326	HSP90AB1	heat shock protein 90kDa alpha (cytosolic), class B member 1
3329	HSPD1	heat shock 60kDa protein 1 (chaperonin)
333	APLP1	amyloid beta (A4) precursor-like protein 1
3336	HSPE1	heat shock 10kDa protein 1 (chaperonin 10)
3339	HSPG2	heparan sulfate proteoglycan 2
3345	HTLVR	human T-cell leukemia virus (I and II) receptor
335	APOA1	apolipoprotein A-I
3351	HTR1B	5-hydroxytryptamine (serotonin) receptor 1B
3357	HTR2B	5-hydroxytryptamine (serotonin) receptor 2B
336	APOA2	apolipoprotein A-II
3362	HTR6	5-hydroxytryptamine (serotonin) receptor 6
3369	HVBS7	hepatitis B virus integration site 7
337	APOA4	apolipoprotein A-IV
3370	HVBS8	hepatitis B virus integration site 8
3371	TNC	tenascin C
3375	IAPP	islet amyloid polypeptide
338	APOB	apolipoprotein B (including Ag(x) antigen)
338091	PSMD10P3	proteasome 26S subunit, non-ATPase, 10 pseudogene 3
338095	PSME2P3	proteasome activator subunit 2 pseudogene 3
338096	PSME2P4	proteasome activator subunit 2 pseudogene 4
338097	PSME2P5	proteasome activator subunit 2 pseudogene 5
338098	PSME2P6	proteasome activator subunit 2 pseudogene 6
338099	PSME2P2	proteasome activator subunit 2 pseudogene 2
3381	IBSP	integrin-binding sialoprotein
3382	ICA1	islet cell autoantigen 1, 69kDa

3383	ICAM1	intercellular adhesion molecule 1
338376	IFNE	interferon, epsilon
3384	ICAM2	intercellular adhesion molecule 2
338436	BLACE	B-cell acute lymphoblastic leukemia expressed
338442	HCAR2	hydroxycarboxylic acid receptor 2
3385	ICAM3	intercellular adhesion molecule 3
338557	O3FAR1	omega-3 fatty acid receptor 1
3386	ICAM4	intercellular adhesion molecule 4 (Landsteiner-Wiener blood group)
338761	C1QL4	complement component 1, q subcomponent-like 4
338811	FAM19A2	family with sequence similarity 19 (chemokine (C-C motif)-like), member A2
338872	C1QTNF9	C1q and tumor necrosis factor related protein 9
339302	CPLX4	complexin 4
3394	IRF8	interferon regulatory factor 8
339562	IGKV1OR2-118	immunoglobulin kappa variable 1/OR2-118 (pseudogene)
3397	ID1	inhibitor of DNA binding 1, dominant negative helix-loop-helix protein
3398	ID2	inhibitor of DNA binding 2, dominant negative helix-loop-helix protein
3399	ID3	inhibitor of DNA binding 3, dominant negative helix-loop-helix protein
3400	ID4	inhibitor of DNA binding 4, dominant negative helix-loop-helix protein
340061	TMEM173	transmembrane protein 173
3401	IDDM2	insulin-dependent diabetes mellitus 2
340198	IFITM4P	interferon induced transmembrane protein 4 pseudogene
3402	IDDM3	insulin-dependent diabetes mellitus 3
340205	TREML1	triggering receptor expressed on myeloid cells-like 1
340206	TREML3	triggering receptor expressed on myeloid cells-like 3
340267	COL28A1	collagen, type XXVIII, alpha 1
3403	IDDM4	insulin-dependent diabetes mellitus 4
340371	NRBP2	nuclear receptor binding protein 2
340485	ACER2	alkaline ceramidase 2
3405	IDDM6	insulin-dependent diabetes mellitus 6
340547	VSIG1	V-set and immunoglobulin domain containing 1
340562	SATL1	spermidine/spermine N1-acetyl transferase-like 1
3406	IDDM7	insulin-dependent diabetes mellitus 7
340665	CYP26C1	cytochrome P450, family 26, subfamily C, polypeptide 1
3407	IDDM8	insulin-dependent diabetes mellitus 8
340745	LRIT2	leucine-rich repeat, immunoglobulin-like and transmembrane domains 2
3408	IDDM9	insulin-dependent diabetes mellitus 9
3409	IDDM10	insulin-dependent diabetes mellitus 10

341	APOC1	apolipoprotein C-I
3410	IDDM11	insulin-dependent diabetes mellitus 11
3412	IDDM13	insulin-dependent diabetes mellitus 13
3413	IDDM14	insulin-dependent diabetes mellitus 14
3414	IDDM15	insulin-dependent diabetes mellitus 15
3415	IDDM16	insulin-dependent diabetes mellitus 16
3416	IDE	insulin-degrading enzyme
341640	FREM2	FRAS1 related extracellular matrix protein 2
342510	CD300E	CD300e molecule
3426	CFI	complement factor I
342618	SLFN14	schlafen family member 14
3428	IFI16	interferon, gamma-inducible protein 16
342898	SYCN	syncollin
3429	IFI27	interferon, alpha-inducible protein 27
342977	NANOS3	nanos homolog 3 (Drosophila)
342979	PALM3	paralemmin 3
3430	IFI35	interferon-induced protein 35
343296	ADH5P2	alcohol dehydrogenase 5 (class III), chi polypeptide, pseudogene 2
3433	IFIT2	interferon-induced protein with tetratricopeptide repeats 2
3434	IFIT1	interferon-induced protein with tetratricopeptide repeats 1
343413	FCRL6	Fc receptor-like 6
3437	IFIT3	interferon-induced protein with tetratricopeptide repeats 3
343702	XKR7	XK, Kell blood group complex subunit-related family, member 7
3438	IFN1@	interferon, type 1, cluster
3439	IFNA1	interferon, alpha 1
344	APOC2	apolipoprotein C-II
3440	IFNA2	interferon, alpha 2
3441	IFNA4	interferon, alpha 4
3442	IFNA5	interferon, alpha 5
3443	IFNA6	interferon, alpha 6
3444	IFNA7	interferon, alpha 7
3445	IFNA8	interferon, alpha 8
3446	IFNA10	interferon, alpha 10
3447	IFNA13	interferon, alpha 13
3448	IFNA14	interferon, alpha 14
344807	CD200R1L	CD200 receptor 1-like
3449	IFNA16	interferon, alpha 16

345	APOC3	apolipoprotein C-III
3451	IFNA17	interferon, alpha 17
345193	LRIT3	leucine-rich repeat, immunoglobulin-like and transmembrane domains 3
3452	IFNA21	interferon, alpha 21
3453	IFNA22P	interferon, alpha 22, pseudogene
3454	IFNAR1	interferon (alpha, beta and omega) receptor 1
3455	IFNAR2	interferon (alpha, beta and omega) receptor 2
3456	IFNB1	interferon, beta 1, fibroblast
345611	IRGM	immunity-related GTPase family, M
345645	PSMC1P4	proteasome (prosome, macropain) 26S subunit, ATPase, 1 pseudogene 4
3458	IFNG	interferon, gamma
345895	RSPH4A	radial spoke head 4 homolog A (Chlamydomonas)
3459	IFNGR1	interferon gamma receptor 1
3460	IFNGR2	interferon gamma receptor 2 (interferon gamma transducer 1)
3461	IFNA11P	interferon, alpha 11, pseudogene
3463	IFNA20P	interferon, alpha 20, pseudogene
346547	ERVFC1-1	endogenous retrovirus group FC1, member 1
3466	IFNR	interferon production regulator
3467	IFNW1	interferon, omega 1
3474	IFNWP19	interferon, omega 1 pseudogene 19
3475	IFRD1	interferon-related developmental regulator 1
3476	IGBP1	immunoglobulin (CD79A) binding protein 1
347720	COTL1P2	coactosin-like 1 (Dictyostelium) pseudogene 2
347747	HPCQTL19	Prostate cancer aggressiveness quantitative trait locus on chromosome 19
3478	IGES	immunoglobulin E concentration, serum
3479	IGF1	insulin-like growth factor 1 (somatomedin C)
347902	AMIGO2	adhesion molecule with Ig-like domain 2
348	APOE	apolipoprotein E
3480	IGF1R	insulin-like growth factor 1 receptor
3481	IGF2	insulin-like growth factor 2 (somatomedin A)
3482	IGF2R	insulin-like growth factor 2 receptor
3483	IGFALS	insulin-like growth factor binding protein, acid labile subunit
3485	IGFBP2	insulin-like growth factor binding protein 2, 36kDa
3486	IGFBP3	insulin-like growth factor binding protein 3
348801	LNP1	leukemia NUP98 fusion partner 1
348910	RAF1P1	v-raf-1 murine leukemia viral oncogene homolog 1 pseudogene 1
3490	IGFBP7	insulin-like growth factor binding protein 7

3491	CYR61	cysteine-rich, angiogenic inducer, 61
3492	IGH@	immunoglobulin heavy locus
3493	IGHA1	immunoglobulin heavy constant alpha 1
349334	FOXD4L4	forkhead box D4-like 4
3494	IGHA2	immunoglobulin heavy constant alpha 2 (A2m marker)
3495	IGHD	immunoglobulin heavy constant delta
3496	IGHDOR15@	immunoglobulin heavy diversity orphans on chromosome 15
3497	IGHE	immunoglobulin heavy constant epsilon
3498	IGHEP1	immunoglobulin heavy constant epsilon P1 (pseudogene)
3499	IGHEP2	immunoglobulin heavy constant epsilon P2 (pseudogene)
350	APOH	apolipoprotein H (beta-2-glycoprotein I)
3500	IGHG1	immunoglobulin heavy constant gamma 1 (G1m marker)
3501	IGHG2	immunoglobulin heavy constant gamma 2 (G2m marker)
3502	IGHG3	immunoglobulin heavy constant gamma 3 (G3m marker)
3503	IGHG4	immunoglobulin heavy constant gamma 4 (G4m marker)
3505	IGHGP	immunoglobulin heavy constant gamma P (non-functional)
3506	IGHJ@	immunoglobulin heavy joining group
3507	IGHM	immunoglobulin heavy constant mu
3508	IGHMBP2	immunoglobulin mu binding protein 2
3509	IGHV@	immunoglobulin heavy variable group
351	APP	amyloid beta (A4) precursor protein
3512	IGJ	immunoglobulin J polypeptide, linker protein for immunoglobulin alpha and mu polypeptides
3513	IGJP1	immunoglobulin J polypeptide pseudogene 1
3514	IGKC	immunoglobulin kappa constant
3515	IGKDEL	immunoglobulin kappa deleting element or like
3516	RBPJ	recombination signal binding protein for immunoglobulin kappa J region
3519	IGKV@	immunoglobulin kappa variable group
3523	IGKV3OR2-268	immunoglobulin kappa variable 3/OR2-268 (non-functional)
3525	IGKV1OR1-1	immunoglobulin kappa variable 1/OR1-1 (pseudogene)
3527	IGKV3OR22-2	immunoglobulin kappa variable 3/OR22-2 (pseudogene)
3529	IGKV2OR22-3	immunoglobulin kappa variable 2/OR22-3 (pseudogene)
352957	MICF	MHC class I polypeptide-related sequence F (pseudogene)
352961	HCG26	HLA complex group 26 (non-protein coding)
352962	HLA-V	major histocompatibility complex, class I, V (pseudogene)
352963	HLA-P	major histocompatibility complex, class I, P (pseudogene)
352964	HLA-T	major histocompatibility complex, class I, T (pseudogene)
352965	HLA-U	major histocompatibility complex, class I, U (pseudogene)

352966	HLA-W	major histocompatibility complex, class I, W (pseudogene)
352967	MICG	MHC class I polypeptide-related sequence G (pseudogene)
352989	P5-11	HLA complex P5 pseudogene
352990	HCP5P10	HLA complex P5 pseudogene 10
352991	P5-09	HLA complex P5 pseudogene
352992	P5-07	HLA complex P5 pseudogene
352993	HCP5P6	HLA complex P5 pseudogene 6
352994	P5-05	HLA complex P5 pseudogene
352995	P5.8	HLA complex P5 pseudogene
352996	P5-04	HLA complex P5 pseudogene
352997	HCP5P3	HLA complex P5 pseudogene 3
352998	HCP5P2	HLA complex P5 pseudogene 2
3530	IGKV1OR22-1	immunoglobulin kappa variable 1/OR22-1 (pseudogene)
353001	HCG4P3	HLA complex group 4 pseudogene 3
353002	HCG4P4	HLA complex group 4 pseudogene 4
353003	HCG4P5	HLA complex group 4 pseudogene 5
353004	HCG4P7	HLA complex group 4 pseudogene 7
353005	HCG4P8	HLA complex group 4 pseudogene 8
353006	HCG2P6	HLA complex group 2 pseudogene 6
353007	3.8-1.2	HLA complex group 26 (non-protein coding) pseudogene
353008	3.8-1.3	HLA complex group 26 (non-protein coding) pseudogene
353009	3.8-1.4	HLA complex group 26 (non-protein coding) pseudogene
353010	3.8-1.5	HLA complex group 26 (non-protein coding) pseudogene
353011	HCP5P12	HLA complex P5 pseudogene 12
353012	HCG2P8	HLA complex group 2 pseudogene 8
353014	HCG4P9	HLA complex group 4 pseudogene 9
353016	HCP5P13	HLA complex P5 pseudogene 13
353018	HCP5P14	HLA complex P5 pseudogene 14
353019	HCG9P5	HLA complex group 9 pseudogene 5
353020	HCG4P11	HLA complex group 4 pseudogene 11
353021	HCP5P15	HLA complex P5 pseudogene 15
353091	RAET1G	retinoic acid early transcript 1G
3531	IGKV1OR-1	immunoglobulin kappa variable 1/OR-1 (pseudogene)
353125	HDLC3	High density lipoprotein cholesterol, low serum, 3
3532	IGKV1OR-2	immunoglobulin kappa variable 1/OR-2 (pseudogene)
3534	IGKV1OR-4	immunoglobulin kappa variable 1/OR-4 (pseudogene)
3535	IGL@	immunoglobulin lambda locus

353500	BMP8A	bone morphogenetic protein 8a
353514	LILRA5	leukocyte immunoglobulin-like receptor, subfamily A (with TM domain), member 5
353515	XKRY2	XK, Kell blood group complex subunit-related, Y-linked 2
3536	IGLC@	immunoglobulin lambda constant group
3537	IGLC1	immunoglobulin lambda constant 1 (Mcg marker)
3538	IGLC2	immunoglobulin lambda constant 2 (Kern-Oz- marker)
3539	IGLC3	immunoglobulin lambda constant 3 (Kern-Oz+ marker)
3540	IGLC4	immunoglobulin lambda constant 4 (pseudogene)
3541	IGLC5	immunoglobulin lambda constant 5 (pseudogene)
3542	IGLC6	immunoglobulin lambda constant 6 (Kern+Oz- marker, gene/pseudogene)
3543	IGLL1	immunoglobulin lambda-like polypeptide 1
3544	IGLL2P	immunoglobulin lambda-like polypeptide 2, pseudogene
3546	IGLV@	immunoglobulin lambda variable group
3547	IGSF1	immunoglobulin superfamily, member 1
3549	IHH	Indian hedgehog
355	FAS	Fas (TNF receptor superfamily, member 6)
3550	IK	IK cytokine, down-regulator of HLA II
3551	IKBKB	inhibitor of kappa light polypeptide gene enhancer in B-cells, kinase beta
3552	IL1A	interleukin 1, alpha
3553	IL1B	interleukin 1, beta
3554	IL1R1	interleukin 1 receptor, type I
3556	IL1RAP	interleukin 1 receptor accessory protein
3557	IL1RN	interleukin 1 receptor antagonist
3558	IL2	interleukin 2
3559	IL2RA	interleukin 2 receptor, alpha
356	FASLG	Fas ligand (TNF superfamily, member 6)
3560	IL2RB	interleukin 2 receptor, beta
3561	IL2RG	interleukin 2 receptor, gamma
3562	IL3	interleukin 3 (colony-stimulating factor, multiple)
3563	IL3RA	interleukin 3 receptor, alpha (low affinity)
3565	IL4	interleukin 4
3566	IL4R	interleukin 4 receptor
3567	IL5	interleukin 5 (colony-stimulating factor, eosinophil)
3568	IL5RA	interleukin 5 receptor, alpha
3569	IL6	interleukin 6 (interferon, beta 2)
3570	IL6R	interleukin 6 receptor
3572	IL6ST	interleukin 6 signal transducer (gp130, oncostatin M receptor)

3573	IL6STP1	interleukin 6 signal transducer (gp130, oncostatin M receptor) pseudogene 1
3574	IL7	interleukin 7
3575	IL7R	interleukin 7 receptor
3576	IL8	interleukin 8
3577	CXCR1	chemokine (C-X-C motif) receptor 1
3578	IL9	interleukin 9
3579	CXCR2	chemokine (C-X-C motif) receptor 2
358	AQP1	aquaporin 1 (Colton blood group)
3580	CXCR2P1	chemokine (C-X-C motif) receptor 2 pseudogene 1
3581	IL9R	interleukin 9 receptor
3582	IL9RP1	interleukin 9 receptor pseudogene 1
3585	IL9RP4	interleukin 9 receptor pseudogene 4
3586	IL10	interleukin 10
3587	IL10RA	interleukin 10 receptor, alpha
3588	IL10RB	interleukin 10 receptor, beta
3589	IL11	interleukin 11
359	AQP2	aquaporin 2 (collecting duct)
3590	IL11RA	interleukin 11 receptor, alpha
3591	IL11RB	interleukin 11 receptor, beta
3592	IL12A	interleukin 12A (natural killer cell stimulatory factor 1, cytotoxic lymphocyte maturation factor 1, p35)
3593	IL12B	interleukin 12B (natural killer cell stimulatory factor 2, cytotoxic lymphocyte maturation factor 2, p40)
3594	IL12RB1	interleukin 12 receptor, beta 1
3595	IL12RB2	interleukin 12 receptor, beta 2
3596	IL13	interleukin 13
3597	IL13RA1	interleukin 13 receptor, alpha 1
3598	IL13RA2	interleukin 13 receptor, alpha 2
359948	IRF2BP2	interferon regulatory factor 2 binding protein 2
360	AQP3	aquaporin 3 (Gill blood group)
3600	IL15	interleukin 15
360001	IFNWP18	interferon, omega 1 pseudogene 18
3601	IL15RA	interleukin 15 receptor, alpha
360132	FKBP9L	FK506 binding protein 9-like
3603	IL16	interleukin 16 (lymphocyte chemoattractant factor)
3604	TNFRSF9	tumor necrosis factor receptor superfamily, member 9
3605	IL17A	interleukin 17A
3606	IL18	interleukin 18 (interferon-gamma-inducing factor)
3608	ILF2	interleukin enhancer binding factor 2, 45kDa

3609	ILF3	interleukin enhancer binding factor 3, 90kDa
361	AQP4	aquaporin 4
3611	ILK	integrin-linked kinase
3614	IMPDH1	IMP (inosine 5'-monophosphate) dehydrogenase 1
3615	IMPDH2	IMP (inosine 5'-monophosphate) dehydrogenase 2
3619	INCENP	inner centromere protein antigens 135/155kDa
3622	ING2	inhibitor of growth family, member 2
3623	INHA	inhibin, alpha
3624	INHBA	inhibin, beta A
3625	INHBB	inhibin, beta B
3626	INHBC	inhibin, beta C
3627	CXCL10	chemokine (C-X-C motif) ligand 10
3630	INS	insulin
3635	INPP5D	inositol polyphosphate-5-phosphatase, 145kDa
3636	INPPL1	inositol polyphosphate phosphatase-like 1
3638	INSIG1	insulin induced gene 1
3640	INSL3	insulin-like 3 (Leydig cell)
3643	INSR	insulin receptor
3654	IRAK1	interleukin-1 receptor-associated kinase 1
3655	ITGA6	integrin, alpha 6
3656	IRAK2	interleukin-1 receptor-associated kinase 2
3659	IRF1	interferon regulatory factor 1
3660	IRF2	interferon regulatory factor 2
3661	IRF3	interferon regulatory factor 3
3662	IRF4	interferon regulatory factor 4
3663	IRF5	interferon regulatory factor 5
3664	IRF6	interferon regulatory factor 6
3665	IRF7	interferon regulatory factor 7
3667	IRS1	insulin receptor substrate 1
3669	ISG20	interferon stimulated exonuclease gene 20kDa
367	AR	androgen receptor
3671	ISLR	immunoglobulin superfamily containing leucine-rich repeat
3672	ITGA1	integrin, alpha 1
3673	ITGA2	integrin, alpha 2 (CD49B, alpha 2 subunit of VLA-2 receptor)
3674	ITGA2B	integrin, alpha 2b (platelet glycoprotein IIb of IIb/IIIa complex, antigen CD41)
3675	ITGA3	integrin, alpha 3 (antigen CD49C, alpha 3 subunit of VLA-3 receptor)
3676	ITGA4	integrin, alpha 4 (antigen CD49D, alpha 4 subunit of VLA-4 receptor)

3678	ITGA5	integrin, alpha 5 (fibronectin receptor, alpha polypeptide)
3679	ITGA7	integrin, alpha 7
3680	ITGA9	integrin, alpha 9
3681	ITGAD	integrin, alpha D
3682	ITGAE	integrin, alpha E (antigen CD103, human mucosal lymphocyte antigen 1; alpha polypeptide)
3683	ITGAL	integrin, alpha L (antigen CD11A (p180), lymphocyte function-associated antigen 1; alpha polypeptide)
3684	ITGAM	integrin, alpha M (complement component 3 receptor 3 subunit)
3685	ITGAV	integrin, alpha V (vitronectin receptor, alpha polypeptide, antigen CD51)
3686	ITGAW	integrin, alpha W
3687	ITGAX	integrin, alpha X (complement component 3 receptor 4 subunit)
3688	ITGB1	integrin, beta 1 (fibronectin receptor, beta polypeptide, antigen CD29 includes MDF2, MSK12)
3689	ITGB2	integrin, beta 2 (complement component 3 receptor 3 and 4 subunit)
369	ARAF	v-raf murine sarcoma 3611 viral oncogene homolog
3690	ITGB3	integrin, beta 3 (platelet glycoprotein IIIa, antigen CD61)
3691	ITGB4	integrin, beta 4
3693	ITGB5	integrin, beta 5
3694	ITGB6	integrin, beta 6
3695	ITGB7	integrin, beta 7
3696	ITGB8	integrin, beta 8
3697	ITIH1	inter-alpha (globulin) inhibitor H1
3700	ITIH4	inter-alpha (globulin) inhibitor H4 (plasma Kallikrein-sensitive glycoprotein)
3702	ITK	IL2-inducible T-cell kinase
3707	ITPKB	inositol-trisphosphate 3-kinase B
3708	ITPR1	inositol 1,4,5-trisphosphate receptor, type 1
3709	ITPR2	inositol 1,4,5-trisphosphate receptor, type 2
3710	ITPR3	inositol 1,4,5-trisphosphate receptor, type 3
3714	JAG2	jagged 2
3716	JAK1	Janus kinase 1
3717	JAK2	Janus kinase 2
3718	JAK3	Janus kinase 3
3720	JARID2	jumonji, AT rich interactive domain 2
3725	JUN	jun proto-oncogene
3726	JUNB	jun B proto-oncogene
3728	JUP	junction plakoglobin
3729	K12T	K12 temperature sensitivity complementing
3730	KAL1	Kallmann syndrome 1 sequence
373071	AIS1	autoimmune susceptibility 1

3732	CD82	CD82 molecule
374407	DNAJB13	DnaJ (Hsp40) homolog, subfamily B, member 13
374462	PTPRQ	protein tyrosine phosphatase, receptor type, Q
375033	PEAR1	platelet endothelial aggregation receptor 1
375056	MIA3	melanoma inhibitory activity family, member 3
375298	CERKL	ceramide kinase-like
3764	KCNJ8	potassium inwardly-rectifying channel, subfamily J, member 8
377630	USP17L2	ubiquitin specific peptidase 17-like 2
3778	KCNMA1	potassium large conductance calcium-activated channel, subfamily M, alpha member 1
3779	KCNMB1	potassium large conductance calcium-activated channel, subfamily M, beta member 1
378426	AIS2	autoimmune susceptibility 2
378427	AIS3	autoimmune susceptibility 3 (vitiligo specific)
378708	APITD1	apoptosis-inducing, TAF9-like domain 1
379028	XKRYP1	XK, Kell blood group complex subunit-related, Y-linked pseudogene 1
379029	XKRYP2	XK, Kell blood group complex subunit-related, Y-linked pseudogene 2
379030	XKRYP3	XK, Kell blood group complex subunit-related, Y-linked pseudogene 3
379031	XKRYP4	XK, Kell blood group complex subunit-related, Y-linked pseudogene 4
379032	XKRYP5	XK, Kell blood group complex subunit-related, Y-linked pseudogene 5
379033	XKRYP6	XK, Kell blood group complex subunit-related, Y-linked pseudogene 6
3791	KDR	kinase insert domain receptor (a type III receptor tyrosine kinase)
3792	KEL	Kell blood group, metallo-endopeptidase
3801	KIFC3	kinesin family member C3
3802	KIR2DL1	killer cell immunoglobulin-like receptor, two domains, long cytoplasmic tail, 1
3803	KIR2DL2	killer cell immunoglobulin-like receptor, two domains, long cytoplasmic tail, 2
3804	KIR2DL3	killer cell immunoglobulin-like receptor, two domains, long cytoplasmic tail, 3
3805	KIR2DL4	killer cell immunoglobulin-like receptor, two domains, long cytoplasmic tail, 4
3806	KIR2DS1	killer cell immunoglobulin-like receptor, two domains, short cytoplasmic tail, 1
3808	KIR2DS3	killer cell immunoglobulin-like receptor, two domains, short cytoplasmic tail, 3
3809	KIR2DS4	killer cell immunoglobulin-like receptor, two domains, short cytoplasmic tail, 4
3810	KIR2DS5	killer cell immunoglobulin-like receptor, two domains, short cytoplasmic tail, 5
3811	KIR3DL1	killer cell immunoglobulin-like receptor, three domains, long cytoplasmic tail, 1
3812	KIR3DL2	killer cell immunoglobulin-like receptor, three domains, long cytoplasmic tail, 2
3813	KIR3DS1	killer cell immunoglobulin-like receptor, three domains, short cytoplasmic tail, 1
3815	KIT	v-kit Hardy-Zuckerman 4 feline sarcoma viral oncogene homolog
382	ARF6	ADP-ribosylation factor 6
3822	KLRC2	killer cell lectin-like receptor subfamily C, member 2
3823	KLRC3	killer cell lectin-like receptor subfamily C, member 3

3827	KNG1	kininogen 1
383	ARG1	arginase, liver
3836	KPNA1	karyopherin alpha 1 (importin alpha 5)
3837	KPNB1	karyopherin (importin) beta 1
384	ARG2	arginase, type II
3845	KRAS	v-Ki-ras2 Kirsten rat sarcoma viral oncogene homolog
3848	KRT1	keratin 1
386653	IL31	interleukin 31
386724	AMIGO3	adhesion molecule with Ig-like domain 3
387	RHOA	ras homolog gene family, member A
387033	CD83P1	CD83 molecule pseudogene 1
387039	HTATSF1P1	HIV-1 Tat specific factor 1 pseudogene 1
387357	THEMIS	thymocyte selection associated
3875	KRT18	keratin 18
387503	HCG4P1	HLA complex group 4 pseudogene 1
387504	HCG4P2	HLA complex group 4 pseudogene 2
387505	HCG9P1	HLA complex group 9 pseudogene 1
387506	HCG9P2	HLA complex group 9 pseudogene 2
387507	HCG9P3	HLA complex group 9 pseudogene 3
387508	HCP5P8	HLA complex P5 pseudogene 8
387578	AITD1	Autoimmune thyroid disease, susceptibility to, 1
387579	AITD2	Autoimmune thyroid disease, susceptibility to, 2
387581	AITD4	Autoimmune thyroid disease, susceptibility to, 4
387597	ILDR2	immunoglobulin-like domain containing receptor 2
387703	LOC387703	x-ray repair cross-complementing protein 6-like
387733	IFITM5	interferon induced transmembrane protein 5
387751	GVINP1	GTPase, very large interferon inducible pseudogene 1
387836	CLEC2A	C-type lectin domain family 2, member A
387911	C1QTNF9B	C1q and tumor necrosis factor related protein 9B
388	RHOB	ras homolog gene family, member B
388015	RTL1	retrotransposon-like 1
388077	IGHV1OR15-1	immunoglobulin heavy variable 1/OR15-1 (pseudogene)
388078	VSIG6	V-set and immunoglobulin domain containing 6
388121	TNFAIP8L3	tumor necrosis factor, alpha-induced protein 8-like 3
388125	C2CD4B	C2 calcium-dependent domain containing 4B
388255	IGHV3OR16-8	immunoglobulin heavy variable 3/OR16-8 (non-functional)
388364	TMIGD1	transmembrane and immunoglobulin domain containing 1

388372	CCL4L2	chemokine (C-C motif) ligand 4-like 2
388503	C3P1	complement component 3 precursor pseudogene
388550	CEACAM22P	carcinoembryonic antigen-related cell adhesion molecule 2, pseudogene
388551	CEACAM16	carcinoembryonic antigen-related cell adhesion molecule 16
388552	BLOC1S3	biogenesis of lysosomal organelles complex-1, subunit 3
388585	HES5	hairy and enhancer of split 5 (Drosophila)
388611	CD164L2	CD164 sialomucin-like 2
388925	PSMC1P10	proteasome (prosome, macropain) 26S subunit, ATPase, 1 pseudogene 10
389	RHOC	ras homolog gene family, member C
389118	CDHR4	cadherin-related family member 4
389362	PSMG4	proteasome (prosome, macropain) assembly chaperone 4
389400	GFRAL	GDNF family receptor alpha like
389610	XKR5	XK, Kell blood group complex subunit-related family, member 5
389668	XKR9	XK, Kell blood group complex subunit-related family, member 9
3897	L1CAM	L1 cell adhesion molecule
389727	LOC389727	FK506 binding protein 4, 59kDa pseudogene
389901	LOC389901	x-ray repair cross-complementing protein 6-like
389941	C1QL3	complement component 1, q subcomponent-like 3
390	RND3	Rho family GTPase 3
3902	LAG3	lymphocyte-activation gene 3
390218	IFITM9P	interferon induced transmembrane protein 9 pseudogene
390297	PSMC1P8	proteasome (prosome, macropain) 26S subunit, ATPase, 1 pseudogene 8
3903	LAIR1	leukocyte-associated immunoglobulin-like receptor 1
3904	LAIR2	leukocyte-associated immunoglobulin-like receptor 2
3905	LAKLG	lymphokine-activated killer cell ligand
390530	IGHV1OR21-1	immunoglobulin heavy variable 1/OR21-1 (non-functional)
390531	VSIG7	V-set and immunoglobulin domain containing 7
390598	SKOR1	SKI family transcriptional corepressor 1
3906	LALBA	lactalbumin, alpha-
390600	LOC390600	cytokine induced apoptosis inhibitor 1 pseudogene
390664	C1QTNF8	C1q and tumor necrosis factor related protein 8
390788	CCL3P1	chemokine (C-C motif) ligand 3 pseudogene 1
3908	LAMA2	laminin, alpha 2
3909	LAMA3	laminin, alpha 3
391	RHOG	ras homolog gene family, member G (rho G)
3910	LAMA4	laminin, alpha 4
391020	LOC391020	interferon induced transmembrane protein pseudogene

3911	LAMA5	laminin, alpha 5
391123	VSIG8	V-set and immunoglobulin domain containing 8
3912	LAMB1	laminin, beta 1
391239	LOC391239	V-set domain containing T cell activation inhibitor 1 pseudogene
3913	LAMB2	laminin, beta 2 (laminin S)
3914	LAMB3	laminin, beta 3
3915	LAMC1	laminin, gamma 1 (formerly LAMB2)
391526	LOC391526	immunoglobulin (CD79A) binding protein 1 pseudogene
391627	USP17	ubiquitin specific peptidase 17
391642	LOC391642	immunoglobulin (CD79A) binding protein 1 pseudogene pseudogene
391672	LOC391672	immunoglobulin (CD79A) binding protein 1 pseudogene
3918	LAMC2	laminin, gamma 2
3920	LAMP2	lysosomal-associated membrane protein 2
3921	RPSA	ribosomal protein SA
392100	PSMC1P3	proteasome (prosome, macropain) 26S subunit, ATPase, 1 pseudogene 3
392217	LOC392217	ig lambda chain V region 4A-like
392255	GDF6	growth differentiation factor 6
392292	IFNWP5	interferon, omega 1 pseudogene 5
392360	CTSL3	cathepsin L family member 3
3929	LBP	lipopolysaccharide binding protein
393	ARHGAP4	Rho GTPase activating protein 4
3932	LCK	lymphocyte-specific protein tyrosine kinase
3934	LCN2	lipocalin 2
3936	LCP1	lymphocyte cytosolic protein 1 (L-plastin)
3937	LCP2	lymphocyte cytosolic protein 2 (SH2 domain containing leukocyte protein of 76kDa)
394	ARHGAP5	Rho GTPase activating protein 5
3949	LDLR	low density lipoprotein receptor
3950	LECT2	leukocyte cell-derived chemotaxin 2
3952	LEP	leptin
3953	LEPR	leptin receptor
3956	LGALS1	lectin, galactoside-binding, soluble, 1
3959	LGALS3BP	lectin, galactoside-binding, soluble, 3 binding protein
396	ARHGDIA	Rho GDP dissociation inhibitor (GDI) alpha
3960	LGALS4	lectin, galactoside-binding, soluble, 4
397	ARHGDIB	Rho GDP dissociation inhibitor (GDI) beta
3976	LIF	leukemia inhibitory factor (cholinergic differentiation factor)
3977	LIFR	leukemia inhibitory factor receptor alpha

398	ARHGDIG	Rho GDP dissociation inhibitor (GDI) gamma
3981	LIG4	ligase IV, DNA, ATP-dependent
3987	LIMS1	LIM and senescent cell antigen-like domains 1
3988	LIPA	lipase A, lysosomal acid, cholesterol esterase
399	RHOH	ras homolog gene family, member H
3993	LLGL2	lethal giant larvae homolog 2 (Drosophila)
3996	LLGL1	lethal giant larvae homolog 1 (Drosophila)
399664	MEX3D	mex-3 homolog D (C. elegans)
399687	MYO18A	myosin XVIIIA
399774	PRKRIRP3	protein-kinase, interferon-inducible double stranded RNA dependent inhibitor, repressor of (P58 repressor) pseudogene 3
399818	METTL10	methyltransferase like 10
4000	LMNA	lamin A/C
400026	LOC400026	protein-kinase, interferon-inducible double stranded RNA dependent inhibitor, repressor of (P58 repressor) pseudogene
4001	LMNB1	lamin B1
400446	LOC400446	PERP, TP53 apoptosis effector pseudogene
400709	SIGLEC16	sialic acid binding Ig-like lectin 16 (gene/pseudogene)
400759	GBP1P1	guanylate binding protein 1, interferon-inducible pseudogene 1
400830	DEFB132	defensin, beta 132
400935	IL17REL	interleukin 17 receptor E-like
401138	AMTN	amelotin
401233	LOC401233	HIV-1 Tat specific factor 1 pseudogene
401447	USP17L1P	ubiquitin specific peptidase 17-like 1, pseudogene
401577	CD99P1	CD99 molecule pseudogene 1
4017	LOXL2	lysyl oxidase-like 2
4018	LPA	lipoprotein, Lp(a)
401827	MSLNL	mesothelin-like
401913	LOC401913	ABO blood group (transferase A, alpha 1-3-N-acetylgalactosaminyltransferase; transferase B, alpha 1-3-galactosyltransferase) pseudogene
4023	LPL	lipoprotein lipase
402415	XKRX	XK, Kell blood group complex subunit-related, X-linked
4026	LPP	LIM domain containing preferred translocation partner in lipoma
402641	LOC402641	v-ral simian leukemia viral oncogene homolog A (ras related) pseudogene
402679	MARK2P10	MAP/microtubule affinity-regulating kinase 2 pseudogene 10
402778	IFITM10	interferon induced transmembrane protein 10
403	ARL3	ADP-ribosylation factor-like 3

4033	LRMP	lymphoid-restricted membrane protein
403339	DEFB133	defensin, beta 133
4035	LRP1	low density lipoprotein receptor-related protein 1
4036	LRP2	low density lipoprotein receptor-related protein 2
4037	LRP3	low density lipoprotein receptor-related protein 3
4038	LRP4	low density lipoprotein receptor-related protein 4
4040	LRP6	low density lipoprotein receptor-related protein 6
404037	HAPLN4	hyaluronan and proteoglycan link protein 4
4041	LRP5	low density lipoprotein receptor-related protein 5
4045	LSAMP	limbic system-associated membrane protein
4046	LSP1	lymphocyte-specific protein 1
404714	SLEB4	systemic lupus erythematosus, susceptibility to, 4
404744	AAA1	asthma-associated alternatively spliced gene 1
4049	LTA	lymphotoxin alpha (TNF superfamily, member 1)
4050	LTB	lymphotoxin beta (TNF superfamily, member 3)
4052	LTBP1	latent transforming growth factor beta binding protein 1
4053	LTBP2	latent transforming growth factor beta binding protein 2
4054	LTBP3	latent transforming growth factor beta binding protein 3
4055	LTBR	lymphotoxin beta receptor (TNFR superfamily, member 3)
4056	LTC4S	leukotriene C4 synthase
4058	LTK	leukocyte receptor tyrosine kinase
4059	BCAM	basal cell adhesion molecule (Lutheran blood group)
4061	LY6E	lymphocyte antigen 6 complex, locus E
4062	LY6H	lymphocyte antigen 6 complex, locus H
4063	LY9	lymphocyte antigen 9
4064	CD180	CD180 molecule
4065	LY75	lymphocyte antigen 75
4066	LYL1	lymphoblastic leukemia derived sequence 1
4067	LYN	v-yes-1 Yamaguchi sarcoma viral related oncogene homolog
4068	SH2D1A	SH2 domain containing 1A
407	ARR3	arrestin 3, retinal (X-arrestin)
4072	EPCAM	epithelial cell adhesion molecule
4074	M6PR	mannose-6-phosphate receptor (cation dependent)
407738	FAM19A1	family with sequence similarity 19 (chemokine (C-C motif)-like), member A1
407977	TNFSF12-TNFSF13	TNFSF12-TNFSF13 readthrough
408	ARRB1	arrestin, beta 1
4082	MARCKS	myristoylated alanine-rich protein kinase C substrate

4083	MARCKSP1	myristoylated alanine-rich protein kinase C substrate pseudogene 1
4085	MAD2L1	MAD2 mitotic arrest deficient-like 1 (yeast)
4086	SMAD1	SMAD family member 1
4087	SMAD2	SMAD family member 2
4088	SMAD3	SMAD family member 3
4089	SMAD4	SMAD family member 4
409	ARRB2	arrestin, beta 2
4090	SMAD5	SMAD family member 5
4091	SMAD6	SMAD family member 6
4092	SMAD7	SMAD family member 7
4093	SMAD9	SMAD family member 9
4099	MAG	myelin associated glycoprotein
4118	MAL	mal, T-cell differentiation protein
4129	MAOB	monoamine oxidase B
4137	MAPT	microtubule-associated protein tau
4139	MARK1	MAP/microtubule affinity-regulating kinase 1
4140	MARK3	MAP/microtubule affinity-regulating kinase 3
414062	CCL3L3	chemokine (C-C motif) ligand 3-like 3
4142	MAS1	MAS1 oncogene
414325	DEFB103A	defensin, beta 103A
414760	HCG14	HLA complex group 14
414761	HCG15	HLA complex group 15
414764	HCG23	HLA complex group 23
414765	HCG25	HLA complex group 25
414766	HCG16	HLA complex group 16
414768	HCG24	HLA complex group 24
414774	HCG20	HLA complex group 20 (non-protein coding)
414775	HCG21	HLA complex group 21 (non-protein coding)
414776	HCG19P	HLA complex group 19 pseudogene
414777	HCG18	HLA complex group 18
414778	HCG17	HLA complex group 17 (non-protein coding)
414887	PTCD2P1	pentatricopeptide repeat domain 2 pseudogene 1
414888	DOCK11P1	dedicator of cytokinesis 11 pseudogene 1
414899	BLID	BH3-like motif containing, cell death inducer
4149	MAX	MYC associated factor X
4151	MB	myoglobin
415116	PIM3	pim-3 oncogene

4153	MBL2	mannose-binding lectin (protein C) 2, soluble
4157	MC1R	melanocortin 1 receptor (alpha melanocyte stimulating hormone receptor)
4162	MCAM	melanoma cell adhesion molecule
4168	MCF2	MCF.2 cell line derived transforming sequence
4170	MCL1	myeloid cell leukemia sequence 1 (BCL2-related)
4179	CD46	CD46 molecule, complement regulatory protein
4182	CD46P1	CD46 molecule, complement regulatory protein pseudogene 1
4185	ADAM11	ADAM metallopeptidase domain 11
4188	MDFI	MyoD family inhibitor
4193	MDM2	Mdm2 p53 binding protein homolog (mouse)
4194	MDM4	Mdm4 p53 binding protein homolog (mouse)
420	ART4	ADP-ribosyltransferase 4 (Dombrock blood group)
4205	MEF2A	myocyte enhancer factor 2A
4208	MEF2C	myocyte enhancer factor 2C
4209	MEF2D	myocyte enhancer factor 2D
421	ARVCF	armadillo repeat gene deleted in velocardiofacial syndrome
4214	MAP3K1	mitogen-activated protein kinase kinase kinase 1
4215	MAP3K3	mitogen-activated protein kinase kinase kinase 3
4216	MAP3K4	mitogen-activated protein kinase kinase kinase 4
4217	MAP3K5	mitogen-activated protein kinase kinase kinase 5
4218	RAB8A	RAB8A, member RAS oncogene family
4221	MEN1	multiple endocrine neoplasia I
4228	MER5	antigen identified by monoclonal antibody 2D8
4234	METTL1	methyltransferase like 1
4237	MFAP2	microfibrillar-associated protein 2
4239	MFAP4	microfibrillar-associated protein 4
4240	MFGE8	milk fat globule-EGF factor 8 protein
4254	KITLG	KIT ligand
4255	MGMT	O-6-methylguanine-DNA methyltransferase
4257	MGST1	microsomal glutathione S-transferase 1
4261	CIITA	class II, major histocompatibility complex, transactivator
4267	CD99	CD99 molecule
4270	MIC7	antigen identified by monoclonal antibody 28.3.7
4273	MIC12	antigen identified by monoclonal antibody 30.2A8
4277	MICB	MHC class I polypeptide-related sequence B
4279	MICD	MHC class I polypeptide-related sequence D (pseudogene)
4280	MICE	MHC class I polypeptide-related sequence E (pseudogene)

4282	MIF	macrophage migration inhibitory factor (glycosylation-inhibiting factor)
4283	CXCL9	chemokine (C-X-C motif) ligand 9
4288	MKI67	antigen identified by monoclonal antibody Ki-67
429	ASCL1	achaete-scute complex homolog 1 (Drosophila)
4291	MLF1	myeloid leukemia factor 1
4292	MLH1	mutL homolog 1, colon cancer, nonpolyposis type 2 (E. coli)
4294	MAP3K10	mitogen-activated protein kinase kinase kinase 10
4296	MAP3K11	mitogen-activated protein kinase kinase kinase 11
4297	MLL	myeloid/lymphoid or mixed-lineage leukemia (trithorax homolog, Drosophila)
4298	MLLT1	myeloid/lymphoid or mixed-lineage leukemia (trithorax homolog, Drosophila); translocated to, 1
43	ACHE	acetylcholinesterase
4300	MLLT3	myeloid/lymphoid or mixed-lineage leukemia (trithorax homolog, Drosophila); translocated to, 3
4301	MLLT4	myeloid/lymphoid or mixed-lineage leukemia (trithorax homolog, Drosophila); translocated to, 4
4302	MLLT6	myeloid/lymphoid or mixed-lineage leukemia (trithorax homolog, Drosophila); translocated to, 6
4309	MLVI2	Moloney murine leukemia virus (MoMuLV) integration site 2 homolog
4312	MMP1	matrix metallopeptidase 1 (interstitial collagenase)
4314	MMP3	matrix metallopeptidase 3 (stromelysin 1, progelatinase)
4318	MMP9	matrix metallopeptidase 9 (gelatinase B, 92kDa gelatinase, 92kDa type IV collagenase)
432	ASGR1	asialoglycoprotein receptor 1
4321	MMP12	matrix metallopeptidase 12 (macrophage elastase)
4323	MMP14	matrix metallopeptidase 14 (membrane-inserted)
4329	ALDH6A1	aldehyde dehydrogenase 6 family, member A1
433	ASGR2	asialoglycoprotein receptor 2
4331	MNAT1	menage a trois homolog 1, cyclin H assembly factor (Xenopus laevis)
4332	MNDA	myeloid cell nuclear differentiation antigen
4335	MNT	MAX binding protein
4340	MOG	myelin oligodendrocyte glycoprotein
4343	MOV10	Mov10, Moloney leukemia virus 10, homolog (mouse)
4345	CD200	CD200 molecule
4352	MPL	myeloproliferative leukemia virus oncogene
4353	MPO	myeloperoxidase
4354	MPP1	membrane protein, palmitoylated 1, 55kDa
4360	MRC1	mannose receptor, C type 1
4397	MS	multiple sclerosis
439945	IL9RP2	interleukin 9 receptor pseudogene 2
439957	IGKV1ORY-1	immunoglobulin kappa variable 1/ORY-1 (pseudogene)
439996	IFIT1B	interferon-induced protein with tetratricopeptide repeats 1B

440	ASNS	asparagine synthetase (glutamine-hydrolyzing)
440067	LOC440067	caspase-1-like
440068	CARD17	caspase recruitment domain family, member 17
440279	UNC13C	unc-13 homolog C (C. elegans)
440498	HSBP1L1	heat shock factor binding protein 1-like 1
440603	BCL2L15	BCL2-like 15
440786	LOC440786	hypothetical LOC440786
440822	PIWIL3	piwi-like 3 (Drosophila)
440915	POTEKP	POTE ankyrin domain family, member K, pseudogene
441050	PTGES3P	prostaglandin E synthase 3 (cytosolic) pseudogene
441194	PMS2CL	PMS2 C-terminal like pseudogene
441410	LOC441410	FK506 binding protein 4, 59kDa pseudogene
441478	NRARP	NOTCH-regulated ankyrin repeat protein
441730	LOC441730	A disintegrin and metalloproteinase with thrombospondin motifs 7-like
441806	LOC441806	proteasome (prosome, macropain) 26S subunit, non-ATPase, 8 pseudogene
441864	TARM1	T cell-interacting, activating receptor on myeloid cells 1
442017	LOC442017	interferon induced transmembrane protein pseudogene
442153	PSMC1P11	proteasome (prosome, macropain) 26S subunit, ATPase, 1 pseudogene 11
442291	PSMC1P2	proteasome (prosome, macropain) 26S subunit, ATPase, 1 pseudogene 2
442309	LOC442309	interferon induced transmembrane protein pseudogene
442389	LOC442389	interferon induced transmembrane protein pseudogene
442459	LOC442459	X-ray repair complementing defective repair pseudogene
442668	LOC442668	NECAP endocytosis associated 1 pseudogene
4435	CITED1	Cbp/p300-interacting transactivator, with Glu/Asp-rich carboxy-terminal domain, 1
4436	MSH2	mutS homolog 2, colon cancer, nonpolyposis type 1 (E. coli)
4437	MSH3	mutS homolog 3 (E. coli)
4447	MSK9	antigen identified by monoclonal antibody K15
4448	MSK10	antigen identified by monoclonal antibody AJ425
445	ASS1	argininosuccinate synthase 1
4467	MSK32	antigen identified by monoclonal antibody K66
4473	MSK38	antigen identified by monoclonal antibody O5
4478	MSN	moesin
4481	MSR1	macrophage scavenger receptor 1
4485	MST1	macrophage stimulating 1 (hepatocyte growth factor-like)
4486	MST1R	macrophage stimulating 1 receptor (c-met-related tyrosine kinase)
4487	MSX1	msh homeobox 1
4488	MSX2	msh homeobox 2

449491	DEFA8P	defensin, alpha 8 pseudogene
449492	DEFA9P	defensin, alpha 9 pseudogene
449493	DEFA10P	defensin, alpha 10 pseudogene
4502	MT2A	metallothionein 2A
4515	MTCP1	mature T-cell proliferation 1
4585	MUC4	mucin 4, cell surface associated
4586	MUC5AC	mucin 5AC, oligomeric mucus/gel-forming
4593	MUSK	muscle, skeletal, receptor tyrosine kinase
4599	MX1	myxovirus (influenza virus) resistance 1, interferon-inducible protein p78 (mouse)
460	ASTN1	astrotactin 1
4600	MX2	myxovirus (influenza virus) resistance 2 (mouse)
4604	MYBPC1	myosin binding protein C, slow type
4606	MYBPC2	myosin binding protein C, fast type
4607	MYBPC3	myosin binding protein C, cardiac
4608	MYBPH	myosin binding protein H
4615	MYD88	myeloid differentiation primary response gene (88)
4616	GADD45B	growth arrest and DNA-damage-inducible, beta
4627	MYH9	myosin, heavy chain 9, non-muscle
4628	MYH10	myosin, heavy chain 10, non-muscle
4632	MYL1	myosin, light chain 1, alkali; skeletal, fast
4643	MYO1E	myosin IE
4646	MYO6	myosin VI
4654	MYOD1	myogenic differentiation 1
4671	NAIP	NLR family, apoptosis inhibitory protein
4680	CEACAM6	carcinoembryonic antigen-related cell adhesion molecule 6 (non-specific cross reacting antigen)
4683	NBN	nibrin
4684	NCAM1	neural cell adhesion molecule 1
4685	NCAM2	neural cell adhesion molecule 2
4688	NCF2	neutrophil cytosolic factor 2
4690	NCK1	NCK adaptor protein 1
4692	NDN	necdin homolog (mouse)
4693	NDP	Norrie disease (pseudoglioma)
4719	NDUFS1	NADH dehydrogenase (ubiquinone) Fe-S protein 1, 75kDa (NADH-coenzyme Q reductase)
472	ATM	ataxia telangiectasia mutated
4722	NDUFS3	NADH dehydrogenase (ubiquinone) Fe-S protein 3, 30kDa (NADH-coenzyme Q reductase)
4739	NEDD9	neural precursor cell expressed, developmentally down-regulated 9
474	ATOH1	atonal homolog 1 (Drosophila)

474168	WG	Wegener granulomatosis
474185	OA29	Osteoarthritis QTL 29
474186	OA30	Osteoarthritis QTL 29
474188	OA24	Osteoarthritis QTL 24
474195	OA16	Osteoarthritis QTL 16
474196	OA25	Osteoarthritis QTL 25
474221	RA1	Rheumatoid arthritis QTL 01
474222	RA2	Rheumatoid arthritis QTL 01
474223	RA3	Rheumatoid arthritis QTL 02
474224	RA4	Rheumatoid arthritis QTL 04
474225	RA5	Rheumatoid arthritis QTL 01
474226	RA6	Rheumatoid arthritis QTL 06
474227	RA7	Rheumatoid arthritis QTL 07
474228	RA8	Rheumatoid arthritis QTL 08
474229	RA9	Rheumatoid arthritis QTL 09
474230	RA10	Rheumatoid arthritis QTL 10
474231	RA11	Rheumatoid arthritis QTL 11
474232	RA12	Rheumatoid arthritis QTL 12
474233	RA13	Rheumatoid arthritis QTL 13
474234	RA14	Rheumatoid arthritis QTL 14
474235	RA15	Rheumatoid arthritis QTL 15
474236	RA16	Rheumatoid arthritis QTL 16
474237	RA17	Rheumatoid arthritis QTL 17
474238	RA31	Rheumatoid arthritis QTL 31
474239	RA18	Rheumatoid arthritis QTL 18
474240	RA19	Rheumatoid arthritis QTL 19
474241	RA20	Rheumatoid arthritis QTL 20
474242	RA21	Rheumatoid arthritis QTL 21
474243	RA22	Rheumatoid arthritis QTL 22
474244	RA23	Rheumatoid arthritis QTL 23
474245	RA24	Rheumatoid arthritis QTL 24
474246	RA25	Rheumatoid arthritis QTL 25
474247	RA26	Rheumatoid arthritis QTL 26
474248	RA27	Rheumatoid arthritis QTL 27
474249	RA28	Rheumatoid arthritis QTL 28
474250	RA29	Rheumatoid arthritis QTL 29
474251	RA30	Rheumatoid arthritis QTL 30

474285	OA1	Osteoarthritis QTL 1
474286	OA2	Osteoarthritis QTL 2
474287	OA3	Osteoarthritis QTL 3
474288	OA4	Osteoarthritis QTL 4
474289	OA5	Osteoarthritis QTL 5
474290	OA6	Osteoarthritis QTL 6
474291	OA7	Osteoarthritis QTL 7
474292	OA8	Osteoarthritis QTL 8
474293	OA9	Osteoarthritis QTL 9
474310	OA26	Osteoarthritis QTL 26
474343	SPIN2B	spindlin family, member 2B
4745	NELL1	NEL-like 1 (chicken)
4747	NEFL	neurofilament, light polypeptide
4753	NELL2	NEL-like 2 (chicken)
4756	NEO1	neogenin 1
4760	NEUROD1	neurogenic differentiation 1
4763	NF1	neurofibromin 1
4771	NF2	neurofibromin 2 (merlin)
4772	NFATC1	nuclear factor of activated T-cells, cytoplasmic, calcineurin-dependent 1
4773	NFATC2	nuclear factor of activated T-cells, cytoplasmic, calcineurin-dependent 2
4775	NFATC3	nuclear factor of activated T-cells, cytoplasmic, calcineurin-dependent 3
4776	NFATC4	nuclear factor of activated T-cells, cytoplasmic, calcineurin-dependent 4
4778	NFE2	nuclear factor (erythroid-derived 2), 45kDa
4779	NFE2L1	nuclear factor (erythroid-derived 2)-like 1
4780	NFE2L2	nuclear factor (erythroid-derived 2)-like 2
4783	NFIL3	nuclear factor, interleukin 3 regulated
4790	NFKB1	nuclear factor of kappa light polypeptide gene enhancer in B-cells 1
4791	NFKB2	nuclear factor of kappa light polypeptide gene enhancer in B-cells 2 (p49/p100)
4792	NFKBIA	nuclear factor of kappa light polypeptide gene enhancer in B-cells inhibitor, alpha
4793	NFKBIB	nuclear factor of kappa light polypeptide gene enhancer in B-cells inhibitor, beta
4794	NFKBIE	nuclear factor of kappa light polypeptide gene enhancer in B-cells inhibitor, epsilon
4795	NFKBIL1	nuclear factor of kappa light polypeptide gene enhancer in B-cells inhibitor-like 1
4799	NFX1	nuclear transcription factor, X-box binding 1
4803	NGF	nerve growth factor (beta polypeptide)
4804	NGFR	nerve growth factor receptor
481	ATP1B1	ATPase, Na+/K+ transporting, beta 1 polypeptide
4811	NID1	nidogen 1

4812	NIDDM1	non-insulin-dependent diabetes mellitus (common, type 2) 1
4813	NIDDM2	non-insulin-dependent diabetes mellitus (common, type 2) 2
4814	NINJ1	ninjurin 1
4815	NINJ2	ninjurin 2
4818	NKG7	natural killer cell group 7 sequence
4819	NKS1	natural killer cell susceptibility 1
482	ATP1B2	ATPase, Na+/K+ transporting, beta 2 polypeptide
4820	NKTR	natural killer-tumor recognition sequence
4825	NKX6-1	NK6 homeobox 1
483	ATP1B3	ATPase, Na+/K+ transporting, beta 3 polypeptide
4830	NME1	non-metastatic cells 1, protein (NM23A) expressed in
4831	NME2	non-metastatic cells 2, protein (NM23B) expressed in
4832	NME3	non-metastatic cells 3, protein expressed in
4836	NMT1	N-myristoyltransferase 1
4838	NODAL	nodal homolog (mouse)
4842	NOS1	nitric oxide synthase 1 (neuronal)
4843	NOS2	nitric oxide synthase 2, inducible
4846	NOS3	nitric oxide synthase 3 (endothelial cell)
4851	NOTCH1	notch 1
4853	NOTCH2	notch 2
4860	PNP	purine nucleoside phosphorylase
4864	NPC1	Niemann-Pick disease, type C1
4867	NPHP1	nephronophthisis 1 (juvenile)
4868	NPHS1	nephrosis 1, congenital, Finnish type (nephrin)
4869	NPM1	nucleophosmin (nucleolar phosphoprotein B23, numatrin)
487	ATP2A1	ATPase, Ca++ transporting, cardiac muscle, fast twitch 1
4878	NPPA	natriuretic peptide A
488	ATP2A2	ATPase, Ca++ transporting, cardiac muscle, slow twitch 2
4889	NPY5R	neuropeptide Y receptor Y5
489	ATP2A3	ATPase, Ca++ transporting, ubiquitous
4891	SLC11A2	solute carrier family 11 (proton-coupled divalent metal ion transporters), member 2
4892	NRAP	nebulin-related anchoring protein
4893	NRAS	neuroblastoma RAS viral (v-ras) oncogene homolog
4897	NRCAM	neuronal cell adhesion molecule
4899	NRF1	nuclear respiratory factor 1
490	ATP2B1	ATPase, Ca++ transporting, plasma membrane 1
4900	NRGN	neurogranin (protein kinase C substrate, RC3)

4902	NRTN	neurturin
4905	NSF	N-ethylmaleimide-sensitive factor
4907	NT5E	5'-nucleotidase, ecto (CD73)
4908	NTF3	neurotrophin 3
491	ATP2B2	ATPase, Ca++ transporting, plasma membrane 2
492	ATP2B3	ATPase, Ca++ transporting, plasma membrane 3
4921	DDR2	discoidin domain receptor tyrosine kinase 2
4923	NTSR1	neurotensin receptor 1 (high affinity)
4929	NR4A2	nuclear receptor subfamily 4, group A, member 2
493	ATP2B4	ATPase, Ca++ transporting, plasma membrane 4
4938	OAS1	2',5'-oligoadenylate synthetase 1, 40/46kDa
493812	HCG11	HLA complex group 11
493813	HCG13P	HLA complex group 13 pseudogene
493819	HCG10P	HLA complex group 10 pseudogene
493829	TRIM72	tripartite motif containing 72
4939	OAS2	2'-5'-oligoadenylate synthetase 2, 69/71kDa
4940	OAS3	2'-5'-oligoadenylate synthetase 3, 100kDa
4958	OMD	osteomodulin
496	ATP4B	ATPase, H+/K+ exchanging, beta polypeptide
4973	OLR1	oxidized low density lipoprotein (lectin-like) receptor 1
4974	OMG	oligodendrocyte myelin glycoprotein
4976	OPA1	optic atrophy 1 (autosomal dominant)
4978	OPCML	opioid binding protein/cell adhesion molecule-like
498	ATP5A1	ATP synthase, H+ transporting, mitochondrial F1 complex, alpha subunit 1, cardiac muscle
4982	TNFRSF11B	tumor necrosis factor receptor superfamily, member 11b
4983	OPHN1	oligophrenin 1
49860	CRNN	cornulin
49861	CLDN20	claudin 20
4988	OPRM1	opioid receptor, mu 1
4991	OR1D2	olfactory receptor, family 1, subfamily D, member 2
5004	ORM1	orosomucoid 1
5005	ORM2	orosomucoid 2
5008	OSM	oncostatin M
5010	CLDN11	claudin 11
5020	OXT	oxytocin, prepropeptide
5021	OXTR	oxytocin receptor
5023	P2RX1	purinergic receptor P2X, ligand-gated ion channel, 1

5025	P2RX4	purinergic receptor P2X, ligand-gated ion channel, 4
5027	P2RX7	purinergic receptor P2X, ligand-gated ion channel, 7
5028	P2RY1	purinergic receptor P2Y, G-protein coupled, 1
5029	P2RY2	purinergic receptor P2Y, G-protein coupled, 2
503614	DEFB107B	defensin, beta 107B
503618	DEFB104B	defensin, beta 104B
503694	DEFB108P1	defensin, beta 108, pseudogene 1
503837	DEFB108P2	defensin, beta 108, pseudogene 2
503838	DEFB109P3	defensin, beta 109, pseudogene 3
503839	DEFB109P2	defensin, beta 109, pseudogene 2
503840	DEFB108P4	defensin, beta 108, pseudogene 4
503841	DEFB106B	defensin, beta 106B
504180	DEFB105B	defensin, beta 105B
504185	DEFB108P3	defensin, beta 108, pseudogene 3
5045	FURIN	furin (paired basic amino acid cleaving enzyme)
5048	PAFAH1B1	platelet-activating factor acetylhydrolase 1b, regulatory subunit 1 (45kDa)
50484	RRM2B	ribonucleotide reductase M2 B (TP53 inducible)
50488	MINK1	misshapen-like kinase 1
50489	CD207	CD207 molecule, langerin
5049	PAFAH1B2	platelet-activating factor acetylhydrolase 1b, catalytic subunit 2 (30kDa)
5050	PAFAH1B3	platelet-activating factor acetylhydrolase 1b, catalytic subunit 3 (29kDa)
50506	DUOX2	dual oxidase 2
50509	COL5A3	collagen, type V, alpha 3
5051	PAFAH2	platelet-activating factor acetylhydrolase 2, 40kDa
50512	PODXL2	podocalyxin-like 2
50515	CHST11	carbohydrate (chondroitin 4) sulfotransferase 11
5054	SERPINE1	serpin peptidase inhibitor, clade E (nexin, plasminogen activator inhibitor type 1), member 1
5055	SERPINB2	serpin peptidase inhibitor, clade B (ovalbumin), member 2
5058	PAK1	p21 protein (Cdc42/Rac)-activated kinase 1
506	ATP5B	ATP synthase, H+ transporting, mitochondrial F1 complex, beta polypeptide
50604	IL20	interleukin 20
50612	CXCL1P	chemokine (C-X-C motif) ligand 1 pseudogene
50615	IL21R	interleukin 21 receptor
50616	IL22	interleukin 22
50618	ITSN2	intersectin 2
5062	PAK2	p21 protein (Cdc42/Rac)-activated kinase 2
50624	CUZD1	CUB and zona pellucida-like domains 1

50632	CALY	calcyon neuron-specific vesicular protein
50648	IGHD@	immunoglobulin heavy diversity group
50649	ARHGEF4	Rho guanine nucleotide exchange factor (GEF) 4
50650	ARHGEF3	Rho guanine nucleotide exchange factor (GEF) 3
5067	CNTN3	contactin 3 (plasmacytoma associated)
5068	REG3A	regenerating islet-derived 3 alpha
5074	PAWR	PRKC, apoptosis, WT1, regulator
5076	PAX2	paired box 2
5077	PAX3	paired box 3
50802	IGK@	immunoglobulin kappa locus
5081	PAX7	paired box 7
50848	F11R	F11 receptor
50852	TRAT1	T cell receptor associated transmembrane adaptor 1
50855	PARD6A	par-6 partitioning defective 6 homolog alpha (C. elegans)
50856	CLEC4A	C-type lectin domain family 4, member A
50863	NTM	neurotrimin
5087	PBX1	pre-B-cell leukemia homeobox 1
5088	PBX2P1	pre-B-cell leukemia homeobox 2 pseudogene 1
5089	PBX2	pre-B-cell leukemia homeobox 2
5090	PBX3	pre-B-cell leukemia homeobox 3
50937	CDON	Cdon homolog (mouse)
5094	PCBP2	poly(rC) binding protein 2
50940	PDE11A	phosphodiesterase 11A
50943	FOXP3	forkhead box P3
5097	PCDH1	protocadherin 1
5098	PCDHGC3	protocadherin gamma subfamily C, 3
50982	NIDDM3	Noninsulin-dependent diabetes mellitus 3
5099	PCDH7	protocadherin 7
51	ACOX1	acyl-CoA oxidase 1, palmitoyl
5100	PCDH8	protocadherin 8
5101	PCDH9	protocadherin 9
51010	EXOSC3	exosome component 3
51022	GLRX2	glutaredoxin 2
51024	FIS1	fission 1 (mitochondrial outer membrane) homolog (S. cerevisiae)
51035	UBXN1	UBX domain protein 1
51062	ATL1	atlastin GTPase 1
51065	RPS27L	ribosomal protein S27-like

51074	APIP	APAF1 interacting protein
51077	FCF1	FCF1 small subunit (SSU) processome component homolog (S. cerevisiae)
51079	NDUFA13	NADH dehydrogenase (ubiquinone) 1 alpha subcomplex, 13
51083	GAL	galanin prepropeptide
51085	MLXIPL	MLX interacting protein-like
51098	IFT52	intraflagellar transport 52 homolog (Chlamydomonas)
51100	SH3GLB1	SH3-domain GRB2-like endophilin B1
51107	APH1A	anterior pharynx defective 1 homolog A (C. elegans)
51118	UTP11L	UTP11-like, U3 small nucleolar ribonucleoprotein, (yeast)
51119	SBDS	Shwachman-Bodian-Diamond syndrome
51129	ANGPTL4	angiopoietin-like 4
51135	IRAK4	interleukin-1 receptor-associated kinase 4
51147	ING4	inhibitor of growth family, member 4
51148	CERCAM	cerebral endothelial cell adhesion molecule
51150	SDF4	stromal cell derived factor 4
5116	PCNT	pericentrin
51176	LEF1	lymphoid enhancer-binding factor 1
51191	HERC5	hect domain and RLD 5
51192	CKLF	chemokine-like factor
51196	PLCE1	phospholipase C, epsilon 1
51203	NUSAP1	nucleolar and spindle associated protein 1
51206	GP6	glycoprotein VI (platelet)
51208	CLDN18	claudin 18
51214	IGF2AS	insulin-like growth factor 2 antisense
5122	PCSK1	proprotein convertase subtilisin/kexin type 1
51222	ZNF219	zinc finger protein 219
51234	TMEM85	transmembrane protein 85
51237	MZB1	marginal zone B and B1 cell-specific protein
51246	SHISA5	shisa homolog 5 (Xenopus laevis)
5125	PCSK5	proprotein convertase subtilisin/kexin type 5
51257	MARCH2	membrane-associated ring finger (C3HC4) 2
51279	C1RL	complement component 1, r subcomponent-like
51283	BFAR	bifunctional apoptosis regulator
51284	TLR7	toll-like receptor 7
51293	CD320	CD320 molecule
51294	PCDH12	protocadherin 12
51303	FKBP11	FK506 binding protein 11, 19 kDa

51311	TLR8	toll-like receptor 8
51324	SPG21	spastic paraplegia 21 (autosomal recessive, Mast syndrome)
5133	PDCD1	programmed cell death 1
51330	TNFRSF12A	tumor necrosis factor receptor superfamily, member 12A
5134	PDCD2	programmed cell death 2
51348	KLRF1	killer cell lectin-like receptor subfamily F, member 1
5136	PDE1A	phosphodiesterase 1A, calmodulin-dependent
51371	POMP	proteasome maturation protein
51377	UCHL5	ubiquitin carboxyl-terminal hydrolase L5
51378	ANGPT4	angiopoietin 4
51379	CRLF3	cytokine receptor-like factor 3
5138	PDE2A	phosphodiesterase 2A, cGMP-stimulated
5139	PDE3A	phosphodiesterase 3A, cGMP-inhibited
5140	PDE3B	phosphodiesterase 3B, cGMP-inhibited
51422	PRKAG2	protein kinase, AMP-activated, gamma 2 non-catalytic subunit
51428	DDX41	DEAD (Asp-Glu-Ala-Asp) box polypeptide 41
51429	SNX9	sorting nexin 9
51447	IP6K2	inositol hexakisphosphate kinase 2
5145	PDE6A	phosphodiesterase 6A, cGMP-specific, rod, alpha
51454	GULP1	GULP, engulfment adaptor PTB domain containing 1
51473	DCDC2	doublecortin domain containing 2
5148	PDE6G	phosphodiesterase 6G, cGMP-specific, rod, gamma
51493	C22orf28	chromosome 22 open reading frame 28
51497	TH1L	TH1-like (Drosophila)
51499	TRIAP1	TP53 regulated inhibitor of apoptosis 1
5152	PDE9A	phosphodiesterase 9A
5153	PDE1B	phosphodiesterase 1B, calmodulin-dependent
51537	MTFP1	mitochondrial fission process 1
5154	PDGFA	platelet-derived growth factor alpha polypeptide
5155	PDGFB	platelet-derived growth factor beta polypeptide
51554	CCRL1	chemokine (C-C motif) receptor-like 1
5156	PDGFRA	platelet-derived growth factor receptor, alpha polypeptide
51561	IL23A	interleukin 23, alpha subunit p19
51564	HDAC7	histone deacetylase 7
5157	PDGFRL	platelet-derived growth factor receptor-like
5158	PDE6B	phosphodiesterase 6B, cGMP-specific, rod, beta
5159	PDGFRB	platelet-derived growth factor receptor, beta polypeptide

51592	TRIM33	tripartite motif containing 33
51596	CUTA	cutA divalent cation tolerance homolog (E. coli)
51603	METTL13	methyltransferase like 13
51604	PIGT	phosphatidylinositol glycan anchor biosynthesis, class T
51606	ATP6V1H	ATPase, H+ transporting, lysosomal 50/57kDa, V1 subunit H
51616	TAF9B	TAF9B RNA polymerase II, TATA box binding protein (TBP)-associated factor, 31kDa
51651	PTRH2	peptidyl-tRNA hydrolase 2
51661	FKBP7	FK506 binding protein 7
51665	ASB1	ankyrin repeat and SOCS box containing 1
51667	NUB1	negative regulator of ubiquitin-like proteins 1
51668	HSPB11	heat shock protein family B (small), member 11
5167	ENPP1	ectonucleotide pyrophosphatase/phosphodiesterase 1
5168	ENPP2	ectonucleotide pyrophosphatase/phosphodiesterase 2
5170	PDPK1	3-phosphoinositide dependent protein kinase-1
51701	NLK	nemo-like kinase
51738	GHRL	ghrelin/obestatin prepropeptide
51741	WWOX	WW domain containing oxidoreductase
51744	CD244	CD244 molecule, natural killer cell receptor 2B4
51747	LUC7L3	LUC7-like 3 (S. cerevisiae)
5175	PECAM1	platelet/endothelial cell adhesion molecule
51752	ERAP1	endoplasmic reticulum aminopeptidase 1
51754	TMEM8B	transmembrane protein 8B
51765	MST4	serine/threonine protein kinase MST4
51776	ZAK	sterile alpha motif and leucine zipper containing kinase AZK
5178	PEG3	paternally expressed 3
51804	SIX4	SIX homeobox 4
51816	CECR1	cat eye syndrome chromosome region, candidate 1
5196	PF4	platelet factor 4
5197	PF4V1	platelet factor 4 variant 1
5199	CFP	complement factor properdin
5207	PFKFB1	6-phosphofructo-2-kinase/fructose-2,6-biphosphatase 1
5214	PFKP	phosphofructokinase, platelet
5216	PFN1	profilin 1
5223	PGAM1	phosphoglycerate mutase 1 (brain)
5229	PGGT1B	protein geranylgeranyltransferase type I, beta subunit
5239	PGM5	phosphoglucomutase 5
5245	PHB	prohibitin

5265	SERPINA1	serpin peptidase inhibitor, clade A (alpha-1 antiproteinase, antitrypsin), member 1
5270	SERPINE2	serpin peptidase inhibitor, clade E (nexin, plasminogen activator inhibitor type 1), member 2
5272	SERPINB9	serpin peptidase inhibitor, clade B (ovalbumin), member 9
5274	SERPINI1	serpin peptidase inhibitor, clade I (neuroserpin), member 1
5284	PIGR	polymeric immunoglobulin receptor
5289	PIK3C3	phosphoinositide-3-kinase, class 3
5290	PIK3CA	phosphoinositide-3-kinase, catalytic, alpha polypeptide
5291	PIK3CB	phosphoinositide-3-kinase, catalytic, beta polypeptide
5292	PIM1	pim-1 oncogene
5293	PIK3CD	phosphoinositide-3-kinase, catalytic, delta polypeptide
5294	PIK3CG	phosphoinositide-3-kinase, catalytic, gamma polypeptide
5295	PIK3R1	phosphoinositide-3-kinase, regulatory subunit 1 (alpha)
5296	PIK3R2	phosphoinositide-3-kinase, regulatory subunit 2 (beta)
5298	PI4KB	phosphatidylinositol 4-kinase, catalytic, beta
5300	PIN1	peptidylprolyl cis/trans isomerase, NIMA-interacting 1
5307	PITX1	paired-like homeodomain 1
5310	PKD1	polycystic kidney disease 1 (autosomal dominant)
5314	PKHD1	polycystic kidney and hepatic disease 1 (autosomal recessive)
5315	PKM2	pyruvate kinase, muscle
5316	PKNOX1	PBX/knotted 1 homeobox 1
5317	PKP1	plakophilin 1 (ectodermal dysplasia/skin fragility syndrome)
5318	PKP2	plakophilin 2
5319	PLA2G1B	phospholipase A2, group IB (pancreas)
5320	PLA2G2A	phospholipase A2, group IIA (platelets, synovial fluid)
5321	PLA2G4A	phospholipase A2, group IVA (cytosolic, calcium-dependent)
5322	PLA2G5	phospholipase A2, group V
5325	PLAGL1	pleiomorphic adenoma gene-like 1
5328	PLAU	plasminogen activator, urokinase
5329	PLAUR	plasminogen activator, urokinase receptor
53335	BCL11A	B-cell CLL/lymphoma 11A (zinc finger protein)
53342	IL17D	interleukin 17D
53347	UBASH3A	ubiquitin associated and SH3 domain containing A
5335	PLCG1	phospholipase C, gamma 1
53353	LRP1B	low density lipoprotein receptor-related protein 1B
5336	PLCG2	phospholipase C, gamma 2 (phosphatidylinositol-specific)
53368	MDC1B	Muscular dystrophy, congenital, 1B
5337	PLD1	phospholipase D1, phosphatidylcholine-specific

5338	PLD2	phospholipase D2
5339	PLEC	plectin
5340	PLG	plasminogen
5341	PLEK	pleckstrin
5345	SERPINF2	serpin peptidase inhibitor, clade F (alpha-2 antiplasmin, pigment epithelium derived factor), member 2
5347	PLK1	polo-like kinase 1
5354	PLP1	proteolipid protein 1
5355	PLP2	proteolipid protein 2 (colonic epithelium-enriched)
5359	PLSCR1	phospholipid scramblase 1
53616	ADAM22	ADAM metallopeptidase domain 22
53632	PRKAG3	protein kinase, AMP-activated, gamma 3 non-catalytic subunit
5366	PMAIP1	phorbol-12-myristate-13-acetate-induced protein 1
5371	PML	promyelocytic leukemia
5379	PMS2P1	postmeiotic segregation increased 2 pseudogene 1
5380	PMS2L2	postmeiotic segregation increased 2-like 2 pseudogene
53827	FXYD5	FXYD domain containing ion transport regulator 5
5383	PMS2P5	postmeiotic segregation increased 2 pseudogene 5
53832	IL20RA	interleukin 20 receptor, alpha
53833	IL20RB	interleukin 20 receptor beta
53841	CDHR5	cadherin-related family member 5
53842	CLDN22	claudin 22
53844	COPG2IT1	COPG2 imprinted transcript 1 (non-protein coding)
53905	DUOX1	dual oxidase 1
53938	PPIL3	peptidylprolyl isomerase (cyclophilin)-like 3
53942	CNTN5	contactin 5
5395	PMS2	PMS2 postmeiotic segregation increased 2 (S. cerevisiae)
54	ACP5	acid phosphatase 5, tartrate resistant
54035	PSMD4P1	proteasome (prosome, macropain) 26S subunit, non-ATPase, 4 pseudogene 1
54084	TSPEAR	thrombospondin-type laminin G domain and EAR repeats
54097	FAM3B	family with sequence similarity 3, member B
54098	C1QBPP	complement component 1, q subcomponent binding protein, pseudogene
54106	TLR9	toll-like receptor 9
5411	PNN	pinin, desmosome associated protein
5413	SEPT5	septin 5
5414	SEPT4	septin 4
541468	C1orf190	chromosome 1 open reading frame 190
541473	LOC541473	FK506 binding protein 6, 36kDa pseudogene

54187	NANS	N-acetylneuraminic acid synthase
5420	PODXL	podocalyxin-like
54205	CYCS	cytochrome c, somatic
54209	TREM2	triggering receptor expressed on myeloid cells 2
54210	TREM1	triggering receptor expressed on myeloid cells 1
5423	POLB	polymerase (DNA directed), beta
54331	GNG2	guanine nucleotide binding protein (G protein), gamma 2
54345	SOX18	SRY (sex determining region Y)-box 18
5436	POLR2G	polymerase (RNA) II (DNA directed) polypeptide G
54360	CYTL1	cytokine-like 1
54361	WNT4	wingless-type MMTV integration site family, member 4
54413	NLGN3	neuroligin 3
5443	POMC	proopiomelanocortin
54431	DNAJC10	DnaJ (Hsp40) homolog, subfamily C, member 10
544317	AASTH1	Allergic/atopic asthma related QTL 1
544318	OA19	Osteoarthritis QTL 19
544320	OA18	Osteoarthritis QTL 18
544321	OA13	Osteoarthritis QTL 13
544322	OA28	Osteoarthritis QTL 28
544323	OA20	Osteoarthritis QTL 20
544324	OA23	Osteoarthritis QTL 23
544325	OA21	Osteoarthritis QTL 21
544327	OA10	Osteoarthritis QTL 10
544328	OA11	Osteoarthritis QTL 11
544329	OA17	Osteoarthritis QTL 17
544331	OA14	Osteoarthritis QTL 14
544332	OA22	Osteoarthritis QTL 22
544333	OA12	Osteoarthritis QTL 12
544334	OA27	Osteoarthritis QTL 27
544335	OA15	Osteoarthritis QTL 15
54435	HCG4	HLA complex group 4 (non-protein coding)
54440	SASH3	SAM and SH3 domain containing 3
544423	AASTH6	Allergic/atopic asthma related QTL 6
544425	AASTH7	Allergic/atopic asthma related QTL 7
54443	ANLN	anillin, actin binding protein
544434	AASTH5	Allergic/atopic asthma related QTL 5
544440	AASTH4	Allergic/atopic asthma related QTL 4

544441	AASTH3	Allergic/atopic asthma related QTL 3
544446	AASTH2	Allergic/atopic asthma related QTL 2
54453	RIN2	Ras and Rab interactor 2
54456	MOV10L1	Mov10l1, Moloney leukemia virus 10-like 1, homolog (mouse)
544560	AASTH30	Allergic/atopic asthma related QTL 30
544561	AASTH31	Allergic/atopic asthma related QTL 31
544562	AASTH52	Allergic/atopic asthma related QTL 52
544563	AASTH48	Allergic/atopic asthma related QTL 48
544564	AASTH34	Allergic/atopic asthma related QTL 34
544565	AASTH20	Allergic/atopic asthma related QTL 20
544566	AASTH40	Allergic/atopic asthma related QTL 40
544567	AASTH14	Allergic/atopic asthma related QTL 14
544568	AASTH9	Allergic/atopic asthma related QTL 9
544569	AASTH21	Allergic/atopic asthma related QTL 21
544570	AASTH25	Allergic/atopic asthma related QTL 25
544571	AASTH47	Allergic/atopic asthma related QTL 47
544572	AASTH39	Allergic/atopic asthma related QTL 39
544573	AASTH17	Allergic/atopic asthma related QTL 17
544574	AASTH35	Allergic/atopic asthma related QTL 35
544575	AASTH38	Allergic/atopic asthma related QTL 38
544576	AASTH26	Allergic/atopic asthma related QTL 25
544577	AASTH49	Allergic/atopic asthma related QTL 49
544578	AASTH50	Allergic/atopic asthma related QTL 50
544579	AASTH12	Allergic/atopic asthma related QTL 12
544580	AASTH23	Allergic/atopic asthma related QTL 23
544581	AASTH55	Allergic/atopic asthma related QTL 5
544582	AASTH42	Allergic/atopic asthma related QTL 42
544583	AASTH13	Allergic/atopic asthma related QTL 13
544584	AASTH11	Allergic/atopic asthma related QTL 11
544585	AASTH36	Allergic/atopic asthma related QTL 36
544586	AASTH54	Allergic/atopic asthma related QTL 54
544587	AASTH51	Allergic/atopic asthma related QTL 51
544588	AASTH33	Allergic/atopic asthma related QTL 33
544589	AASTH19	Allergic/atopic asthma related QTL 19
544590	AASTH43	Allergic/atopic asthma related QTL 43
544591	AASTH8	Allergic/atopic asthma related QTL 8
544592	AASTH27	Allergic/atopic asthma related QTL 27

544593	AASTH53	Allergic/atopic asthma related QTL 53
544594	AASTH44	Allergic/atopic asthma related QTL 44
544595	AASTH18	Allergic/atopic asthma related QTL 18
544596	AASTH16	Allergic/atopic asthma related QTL 16
544597	AASTH15	Allergic/atopic asthma related QTL 15
544598	AASTH37	Allergic/atopic asthma related QTL 37
544599	AASTH45	Allergic/atopic asthma related QTL 45
544600	AASTH24	Allergic/atopic asthma related QTL 24
544601	AASTH46	Allergic/atopic asthma related QTL 46
544602	AASTH22	Allergic/atopic asthma related QTL 22
544603	AASTH10	Allergic/atopic asthma related QTL 10
544631	SLEP2	Serum leptin concentration QTL 2
544650	SLEP1	Serum leptin concentration QTL 1
54472	TOLLIP	toll interacting protein
54474	KRT20	keratin 20
54476	RNF216	ring finger protein 216
54487	DGCR8	DiGeorge syndrome critical region gene 8
545	ATR	ataxia telangiectasia and Rad3 related
54507	ADAMTSL4	ADAMTS-like 4
54510	PCDH18	protocadherin 18
54536	EXOC6	exocyst complex component 6
54541	DDIT4	DNA-damage-inducible transcript 4
54549	SDK2	sidekick homolog 2 (chicken)
5455	POU3F3	POU class 3 homeobox 3
54556	ING3	inhibitor of growth family, member 3
5457	POU4F1	POU class 4 homeobox 1
54575	UGT1A10	UDP glucuronosyltransferase 1 family, polypeptide A10
54577	UGT1A7	UDP glucuronosyltransferase 1 family, polypeptide A7
5459	POU4F3	POU class 4 homeobox 3
54621	VSIG10	V-set and immunoglobulin domain containing 10
5465	PPARA	peroxisome proliferator-activated receptor alpha
54658	UGT1A1	UDP glucuronosyltransferase 1 family, polypeptide A1
54660	PCDHB18	protocadherin beta 18 pseudogene
5467	PPARD	peroxisome proliferator-activated receptor delta
5468	PPARG	peroxisome proliferator-activated receptor gamma
5469	MED1	mediator complex subunit 1
5473	PPBP	pro-platelet basic protein (chemokine (C-X-C motif) ligand 7)

54739	XAF1	XIAP associated factor 1
54742	LY6K	lymphocyte antigen 6 complex, locus K
54751	FBLIM1	filamin binding LIM protein 1
54756	IL17RD	interleukin 17 receptor D
5476	CTSA	cathepsin A
54766	BTG4	B-cell translocation gene 4
5478	PPIA	peptidylprolyl isomerase A (cyclophilin A)
54790	TET2	tet oncogene family member 2
54795	TRPM4	transient receptor potential cation channel, subfamily M, member 4
54798	DCHS2	dachsous 2 (Drosophila)
5480	PPIC	peptidylprolyl isomerase C (cyclophilin C)
5481	PPID	peptidylprolyl isomerase D
54815	GATAD2A	GATA zinc finger domain containing 2A
54821	ERCC6L	excision repair cross-complementing rodent repair deficiency, complementation group 6-like
54825	CDHR2	cadherin-related family member 2
54829	ASPN	asporin
54841	BIVM	basic, immunoglobulin-like variable motif containing
54843	SYTL2	synaptotagmin-like 2
548594	KIR3DP1	killer cell immunoglobulin-like receptor, three domains, pseudogene 1
54861	SNRK	SNF related kinase
54874	FNBP1L	formin binding protein 1-like
54900	LAX1	lymphocyte transmembrane adaptor 1
54902	TTC19	tetratricopeptide repeat domain 19
54910	SEMA4C	sema domain, immunoglobulin domain (Ig), transmembrane domain (TM) and short cytoplasmic domain, (semaphorin) 4C
54918	CMTM6	CKLF-like MARVEL transmembrane domain containing 6
54929	TMEM161A	transmembrane protein 161A
54941	RNF125	ring finger protein 125
54947	LPCAT2	lysophosphatidylcholine acyltransferase 2
54985	HCFC1R1	host cell factor C1 regulator 1 (XPO1 dependent)
54997	TESC	tescalcin
55023	PHIP	pleckstrin homology domain interacting protein
55024	BANK1	B-cell scaffold protein with ankyrin repeats 1
55031	USP47	ubiquitin specific peptidase 47
55033	FKBP14	FK506 binding protein 14, 22 kDa
55055	ZWILCH	Zwilch, kinetochore associated, homolog (Drosophila)
550641	TELM	telomere length, mean leukocyte

55072	RNF31	ring finger protein 31
55075	UACA	uveal autoantigen with coiled-coil domains and ankyrin repeats
55079	FEZF2	FEZ family zinc finger 2
55080	TAPBPL	TAP binding protein-like
55081	IFT57	intraflagellar transport 57 homolog (Chlamydomonas)
551	AVP	arginine vasopressin
55106	SLFN12	schlafen family member 12
55109	AGGF1	angiogenic factor with G patch and FHA domains 1
55113	XKR8	XK, Kell blood group complex subunit-related family, member 8
55114	ARHGAP17	Rho GTPase activating protein 17
55120	FANCL	Fanconi anemia, complementation group L
55122	AKIRIN2	akirin 2
55124	PIWIL2	piwi-like 2 (Drosophila)
55145	THAP1	THAP domain containing, apoptosis associated protein 1
5515	PPP2CA	protein phosphatase 2, catalytic subunit, alpha isozyme
5516	PPP2CB	protein phosphatase 2, catalytic subunit, beta isozyme
55177	FAM82A2	family with sequence similarity 82, member A2
55178	RNMTL1	RNA methyltransferase like 1
55179	FAIM	Fas apoptotic inhibitory molecule
5518	PPP2R1A	protein phosphatase 2, regulatory subunit A, alpha
552	AVPR1A	arginine vasopressin receptor 1A
55201	MAP1S	microtubule-associated protein 1S
5521	PPP2R2B	protein phosphatase 2, regulatory subunit B, beta
55212	BBS7	Bardet-Biedl syndrome 7
55215	FANCI	Fanconi anemia, complementation group I
5524	PPP2R4	protein phosphatase 2A activator, regulatory subunit 4
55240	STEAP3	STEAP family member 3
55247	NEIL3	nei endonuclease VIII-like 3 (E. coli)
5527	PPP2R5C	protein phosphatase 2, regulatory subunit B', gamma
55288	RHOT1	ras homolog gene family, member T1
553	AVPR1B	arginine vasopressin receptor 1B
553128	KIR2DL5B	killer cell immunoglobulin-like receptor, two domains, long cytoplasmic tail, 5B
553140	SLEP3	Serum leptin concentration QTL 3
553142	AASTH29	Allergic/Atopic asthma related QTL 29
553144	AASTH56	Allergic/Atopic asthma related QTL 56
553145	AASTH32	Allergic/Atopic asthma related QTL 32
553146	AASTH41	Allergic/Atopic asthma related QTL 41

553147	AASTH28	Allergic/Atopic asthma related QTL 28
55327	LIN7C	lin-7 homolog C (C. elegans)
5533	PPP3CC	protein phosphatase 3, catalytic subunit, gamma isozyme
55332	DRAM1	DNA-damage regulated autophagy modulator 1
5534	PPP3R1	protein phosphatase 3, regulatory subunit B, alpha
55340	GIMAP5	GTPase, IMAP family member 5
55367	PIDD	p53-induced death domain protein
5538	PPT1	palmitoyl-protein thioesterase 1
554	AVPR2	arginine vasopressin receptor 2
554223	LOC554223	histocompatibility antigen-related
55423	SIRPG	signal-regulatory protein gamma
554300	KIR2DP1	killer cell immunoglobulin-like receptor, two domains, pseudogene 1
55437	STRADB	STE20-related kinase adaptor beta
55503	TRPV6	transient receptor potential cation channel, subfamily V, member 6
55504	TNFRSF19	tumor necrosis factor receptor superfamily, member 19
5551	PRF1	perforin 1 (pore forming protein)
5552	SRGN	serglycin
5553	PRG2	proteoglycan 2, bone marrow (natural killer cell activator, eosinophil granule major basic protein)
55531	ELMOD1	ELMO/CED-12 domain containing 1
55540	IL17RB	interleukin 17 receptor B
55576	STAB2	stabilin 2
55591	VEZT	vezatin, adherens junctions transmembrane protein
55593	OTUD5	OTU domain containing 5
55612	FERMT1	fermitin family member 1
55619	DOCK10	dedicator of cytokinesis 10
5562	PRKAA1	protein kinase, AMP-activated, alpha 1 catalytic subunit
5563	PRKAA2	protein kinase, AMP-activated, alpha 2 catalytic subunit
55632	G2E3	G2/M-phase specific E3 ubiquitin protein ligase
55636	CHD7	chromodomain helicase DNA binding protein 7
5564	PRKAB1	protein kinase, AMP-activated, beta 1 non-catalytic subunit
55640	FLVCR2	feline leukemia virus subgroup C cellular receptor family, member 2
55646	LYAR	Ly1 antibody reactive homolog (mouse)
5565	PRKAB2	protein kinase, AMP-activated, beta 2 non-catalytic subunit
55655	NLRP2	NLR family, pyrin domain containing 2
5566	PRKACA	protein kinase, cAMP-dependent, catalytic, alpha
5567	PRKACB	protein kinase, cAMP-dependent, catalytic, beta
55679	LIMS2	LIM and senescent cell antigen-like domains 2

5568	PRKACG	protein kinase, cAMP-dependent, catalytic, gamma
55698	RADIL	Ras association and DIL domains
55703	POLR3B	polymerase (RNA) III (DNA directed) polypeptide B
55707	NECAP2	NECAP endocytosis associated 2
5571	PRKAG1	protein kinase, AMP-activated, gamma 1 non-catalytic subunit
55716	LMBR1L	limb region 1 homolog (mouse)-like
5573	PRKAR1A	protein kinase, cAMP-dependent, regulatory, type I, alpha (tissue specific extinguisher 1)
55740	ENAH	enabled homolog (Drosophila)
55742	PARVA	parvin, alpha
55749	CCAR1	cell division cycle and apoptosis regulator 1
5575	PRKAR1B	protein kinase, cAMP-dependent, regulatory, type I, beta
55750	AGK	acylglycerol kinase
5576	PRKAR2A	protein kinase, cAMP-dependent, regulatory, type II, alpha
55763	EXOC1	exocyst complex component 1
55764	IFT122	intraflagellar transport 122 homolog (Chlamydomonas)
5577	PRKAR2B	protein kinase, cAMP-dependent, regulatory, type II, beta
55770	EXOC2	exocyst complex component 2
5578	PRKCA	protein kinase C, alpha
5579	PRKCB	protein kinase C, beta
55795	PCID2	PCI domain containing 2
558	AXL	AXL receptor tyrosine kinase
5580	PRKCD	protein kinase C, delta
55801	IL26	interleukin 26
5581	PRKCE	protein kinase C, epsilon
55811	ADCY10	adenylate cyclase 10 (soluble)
55819	RNF130	ring finger protein 130
5582	PRKCG	protein kinase C, gamma
55824	PAG1	phosphoprotein associated with glycosphingolipid microdomains 1
55825	PECR	peroxisomal trans-2-enoyl-CoA reductase
55829	SELS	selenoprotein S
5583	PRKCH	protein kinase C, eta
5584	PRKCI	protein kinase C, iota
55840	EAF2	ELL associated factor 2
55846	ITFG2	integrin alpha FG-GAP repeat containing 2
5585	PKN1	protein kinase N1
55851	PSENEN	presenilin enhancer 2 homolog (C. elegans)
55854	ZC3H15	zinc finger CCCH-type containing 15

5586	PKN2	protein kinase N2
5587	PRKD1	protein kinase D1
5588	PRKCQ	protein kinase C, theta
5589	PRKCSH	protein kinase C substrate 80K-H
55894	DEFB103B	defensin, beta 103B
5590	PRKCZ	protein kinase C, zeta
55904	MLL5	myeloid/lymphoid or mixed-lineage leukemia 5 (trithorax homolog, Drosophila)
55907	CMAS	cytidine monophosphate N-acetylneuraminic acid synthetase
55909	BIN3	bridging integrator 3
5591	PRKDC	protein kinase, DNA-activated, catalytic polypeptide
55914	ERBB2IP	erbb2 interacting protein
5592	PRKG1	protein kinase, cGMP-dependent, type I
5593	PRKG2	protein kinase, cGMP-dependent, type II
5594	MAPK1	mitogen-activated protein kinase 1
5595	MAPK3	mitogen-activated protein kinase 3
55958	KLHL9	kelch-like 9 (Drosophila)
55964	SEPT3	septin 3
55966	AJAP1	adherens junctions associated protein 1
55970	GNG12	guanine nucleotide binding protein (G protein), gamma 12
55973	BCAP29	B-cell receptor-associated protein 29
5598	MAPK7	mitogen-activated protein kinase 7
5599	MAPK8	mitogen-activated protein kinase 8
5600	MAPK11	mitogen-activated protein kinase 11
5601	MAPK9	mitogen-activated protein kinase 9
5603	MAPK13	mitogen-activated protein kinase 13
56033	BARX1	BARX homeobox 1
56034	PDGFC	platelet derived growth factor C
5604	MAP2K1	mitogen-activated protein kinase kinase 1
56052	ALG1	asparagine-linked glycosylation 1, beta-1,4-mannosyltransferase homolog (S. cerevisiae)
5606	MAP2K3	mitogen-activated protein kinase kinase 3
5608	MAP2K6	mitogen-activated protein kinase kinase 6
56097	PCDHGC5	protocadherin gamma subfamily C, 5
56098	PCDHGC4	protocadherin gamma subfamily C, 4
56099	PCDHGB7	protocadherin gamma subfamily B, 7
5610	EIF2AK2	eukaryotic translation initiation factor 2-alpha kinase 2
56100	PCDHGB6	protocadherin gamma subfamily B, 6
56101	PCDHGB5	protocadherin gamma subfamily B, 5

56102	PCDHGB3	protocadherin gamma subfamily B, 3
56103	PCDHGB2	protocadherin gamma subfamily B, 2
56104	PCDHGB1	protocadherin gamma subfamily B, 1
56105	PCDHGA11	protocadherin gamma subfamily A, 11
56106	PCDHGA10	protocadherin gamma subfamily A, 10
56107	PCDHGA9	protocadherin gamma subfamily A, 9
56108	PCDHGA7	protocadherin gamma subfamily A, 7
56109	PCDHGA6	protocadherin gamma subfamily A, 6
56110	PCDHGA5	protocadherin gamma subfamily A, 5
56111	PCDHGA4	protocadherin gamma subfamily A, 4
56112	PCDHGA3	protocadherin gamma subfamily A, 3
56113	PCDHGA2	protocadherin gamma subfamily A, 2
56114	PCDHGA1	protocadherin gamma subfamily A, 1
5612	PRKRIR	protein-kinase, interferon-inducible double stranded RNA dependent inhibitor, repressor of (P58 repressor)
56121	PCDHB15	protocadherin beta 15
56122	PCDHB14	protocadherin beta 14
56123	PCDHB13	protocadherin beta 13
56124	PCDHB12	protocadherin beta 12
56125	PCDHB11	protocadherin beta 11
56126	PCDHB10	protocadherin beta 10
56127	PCDHB9	protocadherin beta 9
56128	PCDHB8	protocadherin beta 8
56129	PCDHB7	protocadherin beta 7
56130	PCDHB6	protocadherin beta 6
56131	PCDHB4	protocadherin beta 4
56132	PCDHB3	protocadherin beta 3
56133	PCDHB2	protocadherin beta 2
56134	PCDHAC2	protocadherin alpha subfamily C, 2
56135	PCDHAC1	protocadherin alpha subfamily C, 1
56136	PCDHA13	protocadherin alpha 13
56137	PCDHA12	protocadherin alpha 12
56138	PCDHA11	protocadherin alpha 11
56139	PCDHA10	protocadherin alpha 10
56140	PCDHA8	protocadherin alpha 8
56141	PCDHA7	protocadherin alpha 7
56142	PCDHA6	protocadherin alpha 6
56143	PCDHA5	protocadherin alpha 5

56144	PCDHA4	protocadherin alpha 4
56145	PCDHA3	protocadherin alpha 3
56146	PCDHA2	protocadherin alpha 2
56147	PCDHA1	protocadherin alpha 1
56179	SLEB2	systemic lupus erythematosus susceptibility 2
5618	PRLR	prolactin receptor
5621	PRNP	prion protein
5624	PROC	protein C (inactivator of coagulation factors Va and VIIIa)
56244	BTNL2	butyrophilin-like 2 (MHC class II associated)
56246	MRAP	melanocortin 2 receptor accessory protein
5625	PRODH	proline dehydrogenase (oxidase) 1
56253	CRTAM	cytotoxic and regulatory T cell molecule
56259	CTNNBL1	catenin, beta like 1
56262	LRRC8A	leucine rich repeat containing 8 family, member A
56265	CPXM1	carboxypeptidase X (M14 family), member 1
56269	IRGC	immunity-related GTPase family, cinema
5627	PROS1	protein S (alpha)
56286	DAD1P1	defender against cell death 1 pseudogene 1
56288	PARD3	par-3 partitioning defective 3 homolog (C. elegans)
5629	PROX1	prospero homeobox 1
563	AZGP1	alpha-2-glycoprotein 1, zinc-binding
56300	IL36G	interleukin 36, gamma
56301	SLC7A10	solute carrier family 7, (neutral amino acid transporter, y+ system) member 10
5641	LGMN	legumain
56413	LTB4R2	leukotriene B4 receptor 2
5645	PRSS2	protease, serine, 2 (trypsin 2)
56477	CCL28	chemokine (C-C motif) ligand 28
5648	MASP1	mannan-binding lectin serine peptidase 1 (C4/C2 activating component of Ra-reactive factor)
5649	RELN	reelin
5654	HTRA1	HtrA serine peptidase 1
566	AZU1	azurocidin 1
5660	PSAP	prosaposin
56616	DIABLO	diablo, IAP-binding mitochondrial protein
56624	ASAH2	N-acylsphingosine amidohydrolase (non-lysosomal ceramidase) 2
5663	PSEN1	presenilin 1
5664	PSEN2	presenilin 2 (Alzheimer disease 4)
56658	TRIM39	tripartite motif containing 39

56672	AKIP1	A kinase (PRKA) interacting protein 1
567	B2M	beta-2-microglobulin
56729	RETN	resistin
56751	BARHL1	BarH-like homeobox 1
5682	PSMA1	proteasome (prosome, macropain) subunit, alpha type, 1
5683	PSMA2	proteasome (prosome, macropain) subunit, alpha type, 2
56832	IFNK	interferon, kappa
5684	PSMA3	proteasome (prosome, macropain) subunit, alpha type, 3
56848	SPHK2	sphingosine kinase 2
5685	PSMA4	proteasome (prosome, macropain) subunit, alpha type, 4
5686	PSMA5	proteasome (prosome, macropain) subunit, alpha type, 5
5687	PSMA6	proteasome (prosome, macropain) subunit, alpha type, 6
5688	PSMA7	proteasome (prosome, macropain) subunit, alpha type, 7
56882	CDC42SE1	CDC42 small effector 1
5689	PSMB1	proteasome (prosome, macropain) subunit, beta type, 1
56892	C8orf4	chromosome 8 open reading frame 4
5690	PSMB2	proteasome (prosome, macropain) subunit, beta type, 2
5691	PSMB3	proteasome (prosome, macropain) subunit, beta type, 3
56912	IFT46	intraflagellar transport 46 homolog (Chlamydomonas)
5692	PSMB4	proteasome (prosome, macropain) subunit, beta type, 4
56920	SEMA3G	sema domain, immunoglobulin domain (Ig), short basic domain, secreted, (semaphorin) 3G
5693	PSMB5	proteasome (prosome, macropain) subunit, beta type, 5
5694	PSMB6	proteasome (prosome, macropain) subunit, beta type, 6
56940	DUSP22	dual specificity phosphatase 22
5695	PSMB7	proteasome (prosome, macropain) subunit, beta type, 7
56952	PRTFDC1	phosphoribosyl transferase domain containing 1
5696	PSMB8	proteasome (prosome, macropain) subunit, beta type, 8 (large multifunctional peptidase 7)
56971	CEACAM19	carcinoembryonic antigen-related cell adhesion molecule 19
5698	PSMB9	proteasome (prosome, macropain) subunit, beta type, 9 (large multifunctional peptidase 2)
56984	PSMG2	proteasome (prosome, macropain) assembly chaperone 2
5699	PSMB10	proteasome (prosome, macropain) subunit, beta type, 10
56990	CDC42SE2	CDC42 small effector 2
56994	CHPT1	choline phosphotransferase 1
56998	CTNNBIP1	catenin, beta interacting protein 1
5700	PSMC1	proteasome (prosome, macropain) 26S subunit, ATPase, 1
57007	CXCR7	chemokine (C-X-C motif) receptor 7
5701	PSMC2	proteasome (prosome, macropain) 26S subunit, ATPase, 2

57019	CIAPIN1	cytokine induced apoptosis inhibitor 1
5702	PSMC3	proteasome (prosome, macropain) 26S subunit, ATPase, 3
5703	PSMC3P	proteasome (prosome, macropain) 26S subunit, ATPase, 3 pseudogene
5704	PSMC4	proteasome (prosome, macropain) 26S subunit, ATPase, 4
57044	IDDM18	insulin-dependent diabetes mellitus 18
5705	PSMC5	proteasome (prosome, macropain) 26S subunit, ATPase, 5
5706	PSMC6	proteasome (prosome, macropain) 26S subunit, ATPase, 6
5707	PSMD1	proteasome (prosome, macropain) 26S subunit, non-ATPase, 1
5708	PSMD2	proteasome (prosome, macropain) 26S subunit, non-ATPase, 2
5709	PSMD3	proteasome (prosome, macropain) 26S subunit, non-ATPase, 3
57091	CASS4	Cas scaffolding protein family member 4
57095	PITHD1	PITH (C-terminal proteasome-interacting domain of thioredoxin-like) domain containing 1
57099	AVEN	apoptosis, caspase activation inhibitor
5710	PSMD4	proteasome (prosome, macropain) 26S subunit, non-ATPase, 4
5711	PSMD5	proteasome (prosome, macropain) 26S subunit, non-ATPase, 5
57113	TRPC7	transient receptor potential cation channel, subfamily C, member 7
57118	CAMK1D	calcium/calmodulin-dependent protein kinase ID
57124	CD248	CD248 molecule, endosialin
57126	CD177	CD177 molecule
5713	PSMD7	proteasome (prosome, macropain) 26S subunit, non-ATPase, 7
5714	PSMD8	proteasome (prosome, macropain) 26S subunit, non-ATPase, 8
57142	RTN4	reticulon 4
5715	PSMD9	proteasome (prosome, macropain) 26S subunit, non-ATPase, 9
57152	SLURP1	secreted LY6/PLAUR domain containing 1
57154	SMURF1	SMAD specific E3 ubiquitin protein ligase 1
5716	PSMD10	proteasome (prosome, macropain) 26S subunit, non-ATPase, 10
57162	PELI1	pellino homolog 1 (Drosophila)
5717	PSMD11	proteasome (prosome, macropain) 26S subunit, non-ATPase, 11
57172	CAMK1G	calcium/calmodulin-dependent protein kinase IG
5718	PSMD12	proteasome (prosome, macropain) 26S subunit, non-ATPase, 12
5719	PSMD13	proteasome (prosome, macropain) 26S subunit, non-ATPase, 13
572	BAD	BCL2-associated agonist of cell death
5720	PSME1	proteasome (prosome, macropain) activator subunit 1 (PA28 alpha)
5721	PSME2	proteasome (prosome, macropain) activator subunit 2 (PA28 beta)
57228	SMAGP	small cell adhesion glycoprotein
5724	PTAFR	platelet-activating factor receptor
5728	PTEN	phosphatase and tensin homolog

57289	IGHV7-4-1	immunoglobulin heavy variable 7-4-1
5729	PTGDR	prostaglandin D2 receptor (DP)
57290	IGHV3-30-3	immunoglobulin heavy variable 3-30-3
57292	KIR2DL5A	killer cell immunoglobulin-like receptor, two domains, long cytoplasmic tail, 5A
573	BAG1	BCL2-associated athanogene
5730	PTGDS	prostaglandin D2 synthase 21kDa (brain)
5731	PTGER1	prostaglandin E receptor 1 (subtype EP1), 42kDa
5732	PTGER2	prostaglandin E receptor 2 (subtype EP2), 53kDa
57326	PBXIP1	pre-B-cell leukemia homeobox interacting protein 1
5733	PTGER3	prostaglandin E receptor 3 (subtype EP3)
57336	ZNF287	zinc finger protein 287
5734	PTGER4	prostaglandin E receptor 4 (subtype EP4)
57348	TTYH1	tweety homolog 1 (Drosophila)
5735	PTGER4P1	prostaglandin E receptor 4 (subtype EP4) pseudogene 1
5736	PTGER4P2	prostaglandin E receptor 4 (subtype EP4) pseudogene 2
5737	PTGFR	prostaglandin F receptor (FP)
57379	AICDA	activation-induced cytidine deaminase
5738	PTGFRN	prostaglandin F2 receptor negative regulator
5739	PTGIR	prostaglandin I2 (prostacyclin) receptor (IP)
5740	PTGIS	prostaglandin I2 (prostacyclin) synthase
574016	CLLU1OS	chronic lymphocytic leukemia up-regulated 1 opposite strand
57402	S100A14	S100 calcium binding protein A14
574028	CLLU1	chronic lymphocytic leukemia up-regulated 1
57403	RAB22A	RAB22A, member RAS oncogene family
5741	PTH	parathyroid hormone
5742	PTGS1	prostaglandin-endoperoxide synthase 1 (prostaglandin G/H synthase and cyclooxygenase)
5743	PTGS2	prostaglandin-endoperoxide synthase 2 (prostaglandin G/H synthase and cyclooxygenase)
57447	NDRG2	NDRG family member 2
57448	BIRC6	baculoviral IAP repeat containing 6
57449	PLEKHG5	pleckstrin homology domain containing, family G (with RhoGef domain) member 5
57452	GALNTL1	UDP-N-acetyl-alpha-D-galactosamine:polypeptide N-acetylgalactosaminyltransferase-like 1
57453	DSCAML1	Down syndrome cell adhesion molecule like 1
57463	AMIGO1	adhesion molecule with Ig-like domain 1
5747	PTK2	PTK2 protein tyrosine kinase 2
57493	HEG1	HEG homolog 1 (zebrafish)
575	BAI1	brain-specific angiogenesis inhibitor 1
57502	NLGN4X	neuroligin 4, X-linked

57506	MAVS	mitochondrial antiviral signaling protein
57526	PCDH19	protocadherin 19
57534	MIB1	mindbomb homolog 1 (Drosophila)
5754	PTK7	PTK7 protein tyrosine kinase 7
57549	IGSF9	immunoglobulin superfamily, member 9
57555	NLGN2	neuroligin 2
57556	SEMA6A	sema domain, transmembrane domain (TM), and cytoplasmic domain, (semaphorin) 6A
57560	IFT80	intraflagellar transport 80 homolog (Chlamydomonas)
57572	DOCK6	dedicator of cytokinesis 6
57575	PCDH10	protocadherin 10
57580	PREX1	phosphatidylinositol-3,4,5-trisphosphate-dependent Rac exchange factor 1
57591	MKL1	megakaryoblastic leukemia (translocation) 1
57593	EBF4	early B-cell factor 4
57600	FNIP2	folliculin interacting protein 2
57611	ISLR2	immunoglobulin superfamily containing leucine-rich repeat 2
57630	SH3RF1	SH3 domain containing ring finger 1
57642	COL20A1	collagen, type XX, alpha 1
57646	USP28	ubiquitin specific peptidase 28
57662	KIAA1543	KIAA1543
57669	EPB41L5	erythrocyte membrane protein band 4.1 like 5
57678	GPAM	glycerol-3-phosphate acyltransferase, mitochondrial
57697	FANCM	Fanconi anemia, complementation group M
5770	PTPN1	protein tyrosine phosphatase, non-receptor type 1
57706	DENND1A	DENN/MADD domain containing 1A
5771	PTPN2	protein tyrosine phosphatase, non-receptor type 2
57715	SEMA4G	sema domain, immunoglobulin domain (Ig), transmembrane domain (TM) and short cytoplasmic domain, (semaphorin) 4G
57717	PCDHB16	protocadherin beta 16
57721	METTL14	methyltransferase like 14
57722	IGDCC4	immunoglobulin superfamily, DCC subclass, member 4
57761	TRIB3	tribbles homolog 3 (Drosophila)
5777	PTPN6	protein tyrosine phosphatase, non-receptor type 6
57787	MARK4	MAP/microtubule affinity-regulating kinase 4
578	BAK1	BCL2-antagonist/killer 1
57805	KIAA1967	KIAA1967
5781	PTPN11	protein tyrosine phosphatase, non-receptor type 11
57817	HAMP	hepcidin antimicrobial peptide

57820	CCNB1IP1	cyclin B1 interacting protein 1, E3 ubiquitin protein ligase
57823	SLAMF7	SLAM family member 7
57824	HMHB1	histocompatibility (minor) HB-1
57829	ZP4	zona pellucida glycoprotein 4
5783	PTPN13	protein tyrosine phosphatase, non-receptor type 13 (APO-1/CD95 (Fas)-associated phosphatase)
5784	PTPN14	protein tyrosine phosphatase, non-receptor type 14
57863	CADM3	cell adhesion molecule 3
5788	PTPRC	protein tyrosine phosphatase, receptor type, C
579	NKX3-2	NK3 homeobox 2
5791	PTPRE	protein tyrosine phosphatase, receptor type, E
5792	PTPRF	protein tyrosine phosphatase, receptor type, F
5794	PTPRH	protein tyrosine phosphatase, receptor type, H
5795	PTPRJ	protein tyrosine phosphatase, receptor type, J
5796	PTPRK	protein tyrosine phosphatase, receptor type, K
5797	PTPRM	protein tyrosine phosphatase, receptor type, M
5798	PTPRN	protein tyrosine phosphatase, receptor type, N
580	BARD1	BRCA1 associated RING domain 1
5802	PTPRS	protein tyrosine phosphatase, receptor type, S
5806	PTX3	pentraxin 3, long
581	BAX	BCL2-associated X protein
5814	PURB	purine-rich element binding protein B
5817	PVR	poliovirus receptor
5818	PVRL1	poliovirus receptor-related 1 (herpesvirus entry mediator C)
5819	PVRL2	poliovirus receptor-related 2 (herpesvirus entry mediator B)
58191	CXCL16	chemokine (C-X-C motif) ligand 16
5826	ABCD4	ATP-binding cassette, sub-family D (ALD), member 4
5829	PXN	paxillin
583	BBS2	Bardet-Biedl syndrome 2
58484	NLRC4	NLR family, CARD domain containing 4
58494	JAM2	junctional adhesion molecule 2
58495	OVOL2	ovo-like 2 (Drosophila)
58496	LY6G5B	lymphocyte antigen 6 complex, locus G5B
585	BBS4	Bardet-Biedl syndrome 4
58508	MLL3	myeloid/lymphoid or mixed-lineage leukemia 3
58513	EPS15L1	epidermal growth factor receptor pathway substrate 15-like 1
58517	RBM25	RNA binding motif protein 25
58530	LY6G6D	lymphocyte antigen 6 complex, locus G6D

58533	SNX6	sorting nexin 6
5864	RAB3A	RAB3A, member RAS oncogene family
5865	RAB3B	RAB3B, member RAS oncogene family
5867	RAB4A	RAB4A, member RAS oncogene family
5868	RAB5A	RAB5A, member RAS oncogene family
5869	RAB5B	RAB5B, member RAS oncogene family
5871	MAP4K2	mitogen-activated protein kinase kinase kinase kinase 2
5872	RAB13	RAB13, member RAS oncogene family
5873	RAB27A	RAB27A, member RAS oncogene family
5874	RAB27B	RAB27B, member RAS oncogene family
5878	RAB5C	RAB5C, member RAS oncogene family
5879	RAC1	ras-related C3 botulinum toxin substrate 1 (rho family, small GTP binding protein Rac1)
5880	RAC2	ras-related C3 botulinum toxin substrate 2 (rho family, small GTP binding protein Rac2)
5883	RAD9A	RAD9 homolog A (S. pombe)
5885	RAD21	RAD21 homolog (S. pombe)
5886	RAD23A	RAD23 homolog A (S. cerevisiae)
5887	RAD23B	RAD23 homolog B (S. cerevisiae)
5894	RAF1	v-raf-1 murine leukemia viral oncogene homolog 1
5896	RAG1	recombination activating gene 1
5897	RAG2	recombination activating gene 2
5898	RALA	v-ral simian leukemia viral oncogene homolog A (ras related)
58985	IL22RA1	interleukin 22 receptor, alpha 1
58986	TMEM8A	transmembrane protein 8A
5899	RALB	v-ral simian leukemia viral oncogene homolog B (ras related; GTP binding protein)
5901	RAN	RAN, member RAS oncogene family
59067	IL21	interleukin 21
59082	CARD18	caspase recruitment domain family, member 18
5913	RAPSN	receptor-associated protein of the synapse
5914	RARA	retinoic acid receptor, alpha
5916	RARG	retinoic acid receptor, gamma
5919	RARRES2	retinoic acid receptor responder (tazarotene induced) 2
5921	RASA1	RAS p21 protein activator (GTPase activating protein) 1
5923	RASGRF1	Ras protein-specific guanine nucleotide-releasing factor 1
5924	RASGRF2	Ras protein-specific guanine nucleotide-releasing factor 2
5925	RB1	retinoblastoma 1
59269	HIVEP3	human immunodeficiency virus type I enhancer binding protein 3
59272	ACE2	angiotensin I converting enzyme (peptidyl-dipeptidase A) 2

59307	SIGIRR	single immunoglobulin and toll-interleukin 1 receptor (TIR) domain
59338	PLEKHA1	pleckstrin homology domain containing, family A (phosphoinositide binding specific) member 1
59340	HRH4	histamine receptor H4
59341	TRPV4	transient receptor potential cation channel, subfamily V, member 4
59347	FKSG2	tumor protein, translationally-controlled 1 pseudogene
5947	RBP1	retinol binding protein 1, cellular
594855	CPLX3	complexin 3
595	CCND1	cyclin D1
5950	RBP4	retinol binding protein 4, plasma
596	BCL2	B-cell CLL/lymphoma 2
5961	PRPH2	peripherin 2 (retinal degeneration, slow)
5966	REL	v-rel reticuloendotheliosis viral oncogene homolog (avian)
597	BCL2A1	BCL2-related protein A1
5970	RELA	v-rel reticuloendotheliosis viral oncogene homolog A (avian)
5971	RELB	v-rel reticuloendotheliosis viral oncogene homolog B
5977	DPF2	D4, zinc and double PHD fingers family 2
5978	REST	RE1-silencing transcription factor
5979	RET	ret proto-oncogene
598	BCL2L1	BCL2-like 1
5989	RFX1	regulatory factor X, 1 (influences HLA class II expression)
599	BCL2L2	BCL2-like 2
5990	RFX2	regulatory factor X, 2 (influences HLA class II expression)
5991	RFX3	regulatory factor X, 3 (influences HLA class II expression)
5992	RFX4	regulatory factor X, 4 (influences HLA class II expression)
5993	RFX5	regulatory factor X, 5 (influences HLA class II expression)
5997	RGS2	regulator of G-protein signaling 2, 24kDa
6006	RHCE	Rh blood group, CcEe antigens
6007	RHD	Rh blood group, D antigen
6011	GRK1	G protein-coupled receptor kinase 1
602	BCL3	B-cell CLL/lymphoma 3
603	BCL5	B-cell CLL/lymphoma 5
6036	RNASE2	ribonuclease, RNase A family, 2 (liver, eosinophil-derived neurotoxin)
604	BCL6	B-cell CLL/lymphoma 6
60401	EDA2R	ectodysplasin A2 receptor
6041	RNASEL	ribonuclease L (2',5'-oligoisoadenylate synthetase-dependent)
60412	EXOC4	exocyst complex component 4
60437	CDH26	cadherin 26

60484	HAPLN2	hyaluronan and proteoglycan link protein 2
60493	FASTKD5	FAST kinase domains 5
605	BCL7A	B-cell CLL/lymphoma 7A
60529	ALX4	ALX homeobox 4
60675	PROK2	prokineticin 2
60681	FKBP10	FK506 binding protein 10, 65 kDa
607	BCL9	B-cell CLL/lymphoma 9
608	TNFRSF17	tumor necrosis factor receptor superfamily, member 17
6091	ROBO1	roundabout, axon guidance receptor, homolog 1 (Drosophila)
6092	ROBO2	roundabout, axon guidance receptor, homolog 2 (Drosophila)
6093	ROCK1	Rho-associated, coiled-coil containing protein kinase 1
6094	ROM1	retinal outer segment membrane protein 1
6095	RORA	RAR-related orphan receptor A
6097	RORC	RAR-related orphan receptor C
613204	IFITM8P	interferon induced transmembrane protein 8 pseudogene
613209	DEFB135	defensin, beta 135
613210	DEFB136	defensin, beta 136
613211	DEFB134	defensin, beta 134
613253	DEFA1A3	defensin, alpha 1 and alpha 3, variable copy number locus
6146	RPL22	ribosomal protein L22
6188	RPS3	ribosomal protein S3
6189	RPS3A	ribosomal protein S3A
6194	RPS6	ribosomal protein S6
619405	AIS4	autoimmune disease, susceptibility to, 4
6195	RPS6KA1	ribosomal protein S6 kinase, 90kDa, polypeptide 1
619503	FSI	fasting glucose and specific insulin levels
6196	RPS6KA2	ribosomal protein S6 kinase, 90kDa, polypeptide 2
6197	RPS6KA3	ribosomal protein S6 kinase, 90kDa, polypeptide 3
6198	RPS6KB1	ribosomal protein S6 kinase, 70kDa, polypeptide 1
623	BDKRB1	bradykinin receptor B1
6233	RPS27A	ribosomal protein S27a
6236	RRAD	Ras-related associated with diabetes
624	BDKRB2	bradykinin receptor B2
6242	RTKN	rhotekin
6247	RS1	retinoschisin 1
6256	RXRA	retinoid X receptor, alpha
6259	RYK	RYK receptor-like tyrosine kinase

6262	RYR2	ryanodine receptor 2 (cardiac)
627	BDNF	brain-derived neurotrophic factor
6272	SORT1	sortilin 1
6278	S100A7	S100 calcium binding protein A7
6279	S100A8	S100 calcium binding protein A8
6280	S100A9	S100 calcium binding protein A9
6284	S100A13	S100 calcium binding protein A13
6287	SAA@	serum amyloid A1 cluster
6288	SAA1	serum amyloid A1
6289	SAA2	serum amyloid A2
629	CFB	complement factor B
6290	SAA3P	serum amyloid A3 pseudogene
6291	SAA4	serum amyloid A4, constitutive
6304	SATB1	SATB homeobox 1
632	BGLAP	bone gamma-carboxyglutamate (gla) protein
6326	SCN2A	sodium channel, voltage-gated, type II, alpha subunit
634	CEACAM1	carcinoembryonic antigen-related cell adhesion molecule 1 (biliary glycoprotein)
6346	CCL1	chemokine (C-C motif) ligand 1
6347	CCL2	chemokine (C-C motif) ligand 2
6348	CCL3	chemokine (C-C motif) ligand 3
6349	CCL3L1	chemokine (C-C motif) ligand 3-like 1
6351	CCL4	chemokine (C-C motif) ligand 4
6352	CCL5	chemokine (C-C motif) ligand 5
6354	CCL7	chemokine (C-C motif) ligand 7
6355	CCL8	chemokine (C-C motif) ligand 8
6356	CCL11	chemokine (C-C motif) ligand 11
6357	CCL13	chemokine (C-C motif) ligand 13
6358	CCL14	chemokine (C-C motif) ligand 14
6359	CCL15	chemokine (C-C motif) ligand 15
636	BICD1	bicaudal D homolog 1 (Drosophila)
6360	CCL16	chemokine (C-C motif) ligand 16
6361	CCL17	chemokine (C-C motif) ligand 17
6362	CCL18	chemokine (C-C motif) ligand 18 (pulmonary and activation-regulated)
6363	CCL19	chemokine (C-C motif) ligand 19
6364	CCL20	chemokine (C-C motif) ligand 20
6366	CCL21	chemokine (C-C motif) ligand 21
6367	CCL22	chemokine (C-C motif) ligand 22

6368	CCL23	chemokine (C-C motif) ligand 23
6369	CCL24	chemokine (C-C motif) ligand 24
637	BID	BH3 interacting domain death agonist
6370	CCL25	chemokine (C-C motif) ligand 25
6372	CXCL6	chemokine (C-X-C motif) ligand 6 (granulocyte chemotactic protein 2)
6373	CXCL11	chemokine (C-X-C motif) ligand 11
6374	CXCL5	chemokine (C-X-C motif) ligand 5
6375	XCL1	chemokine (C motif) ligand 1
6376	CX3CL1	chemokine (C-X3-C motif) ligand 1
638	BIK	BCL2-interacting killer (apoptosis-inducing)
63826	SRR	serine racemase
63827	BCAN	brevican
6385	SDC4	syndecan 4
6386	SDCBP	syndecan binding protein (syntenin)
6387	CXCL12	chemokine (C-X-C motif) ligand 12
6388	SDF2	stromal cell-derived factor 2
63910	SLC17A9	solute carrier family 17, member 9
63916	ELMO2	engulfment and cell motility 2
63923	TNN	tenascin N
63924	CIDEC	cell death-inducing DFFA-like effector c
63943	FKBPL	FK506 binding protein like
63967	CLSPN	claspin
63970	TP53AIP1	tumor protein p53 regulated apoptosis inducing protein 1
63971	KIF13A	kinesin family member 13A
63976	PRDM16	PR domain containing 16
63979	FIGNL1	fidgetin-like 1
6398	SECTM1	secreted and transmembrane 1
640	BLK	B lymphoid tyrosine kinase
6401	SELE	selectin E
6402	SELL	selectin L
6403	SELP	selectin P (granule membrane protein 140kDa, antigen CD62)
6404	SELPLG	selectin P ligand
6405	SEMA3F	sema domain, immunoglobulin domain (Ig), short basic domain, secreted, (semaphorin) 3F
64065	PERP	PERP, TP53 apoptosis effector
64069	ATOD	Dermatitis, atopic
64072	CDH23	cadherin-related 23
64084	CLSTN2	calsyntenin 2

64092	SAMSN1	SAM domain, SH3 domain and nuclear localization signals 1
64093	SMOC1	SPARC related modular calcium binding 1
64094	SMOC2	SPARC related modular calcium binding 2
64098	PARVG	parvin, gamma
641	BLM	Bloom syndrome, RecQ helicase-like
64109	CRLF2	cytokine receptor-like factor 2
64112	MOAP1	modulator of apoptosis 1
64121	RRAGC	Ras-related GTP binding C
64127	NOD2	nucleotide-binding oligomerization domain containing 2
64130	LIN7B	lin-7 homolog B (C. elegans)
64131	XYLT1	xylosyltransferase I
64135	IFIH1	interferon induced with helicase C domain 1
641517	DEFB109P1B	defensin, beta 109, pseudogene 1B
64167	ERAP2	endoplasmic reticulum aminopeptidase 2
64170	CARD9	caspase recruitment domain family, member 9
641700	ECSCR	endothelial cell-specific chemotaxis regulator
6418	SET	SET nuclear oncogene
6420	SF	Stoltzfus blood group
64207	IRF2BPL	interferon regulatory factor 2 binding protein-like
642131	LOC642131	putative V-set and immunoglobulin domain-containing protein 6-like
64218	SEMA4A	sema domain, immunoglobulin domain (Ig), transmembrane domain (TM) and short cytoplasmic domain, (semaphorin) 4A
6422	SFRP1	secreted frizzled-related protein 1
64223	MLST8	MTOR associated protein, LST8 homolog (S. cerevisiae)
6423	SFRP2	secreted frizzled-related protein 2
6424	SFRP4	secreted frizzled-related protein 4
642413	CTSL1P6	cathepsin L1 pseudogene 6
642443	ADH5P4	alcohol dehydrogenase 5 (class III), chi polypeptide, pseudogene 4
642489	FKBP1C	FK506 binding protein 1C
6425	SFRP5	secreted frizzled-related protein 5
642550	LOC642550	v-ral simian leukemia viral oncogene homolog A (ras related) pseudogene
642869	LOC642869	SET translocation (myeloid leukemia-associated) pseudogene
642996	NFE2L3P1	nuclear factor (erythroid-derived 2)-like 3 pseudogene 1
643	CXCR5	chemokine (C-X-C motif) receptor 5
643058	LOC643058	interferon induced transmembrane protein pseudogene
64318	NOC3L	nucleolar complex associated 3 homolog (S. cerevisiae)
64332	NFKBIZ	nuclear factor of kappa light polypeptide gene enhancer in B-cells inhibitor, zeta

643332	ECRP	ribonuclease, RNase A family, 2 (liver, eosinophil-derived neurotoxin) pseudogene
643587	CTSL1P5	cathepsin L1 pseudogene 5
643637	GLYATL1P4	glycine-N-acyltransferase-like 1 pseudogene 4
643668	PSMC1P9	proteasome (prosome, macropain) 26S subunit, ATPase, 1 pseudogene 9
643709	LOC643709	macrophage expressed 1 pseudogene
643711	LOC643711	platelet-activating factor acetylhydrolase, isoform Ib, beta subunit 30kDa pseudogene
643733	LOC643733	caspase 4, apoptosis-related cysteine peptidase pseudogene
643766	PSMC1P6	proteasome (prosome, macropain) 26S subunit, ATPase, 1 pseudogene 6
643784	LOC643784	NLR family, apoptosis inhibitory protein pseudogene
64388	GREM2	gremlin 2
643880	LOC643880	FK506 binding protein 4, 59kDa pseudogene
643884	LOC643884	suppressor of cytokine signaling 5 pseudogene
6439	SFTPB	surfactant protein B
64393	ZMAT3	zinc finger, matrin-type 3
6440	SFTPC	surfactant protein C
64400	AKTIP	AKT interacting protein
644021	CTSL1P3	cathepsin L1 pseudogene 3
64403	CDH24	cadherin 24, type 2
64405	CDH22	cadherin 22, type 2
644076	GLYCAM1	glycosylation dependent cell adhesion molecule 1 (pseudogene)
644094	PSMC1P12	proteasome (prosome, macropain) 26S subunit, ATPase, 1 pseudogene 12
6441	SFTPD	surfactant protein D
64421	DCLRE1C	DNA cross-link repair 1C
64422	ATG3	ATG3 autophagy related 3 homolog (S. cerevisiae)
644325	LOC644325	disintegrin and metalloprotease domain protein pseudogene
644354	LOC644354	putative apoptosis-related protein 2-like
644414	DEFB131	defensin, beta 131
644456	LOC644456	IK cytokine, down-regulator of HLA II pseudogene
644496	CTSL1P4	cathepsin L1 pseudogene 4
6446	SGK1	serum/glucocorticoid regulated kinase 1
644616	LOC644616	interleukin enhancer binding factor 2, 45kDa pseudogene
644731	IGKV1OR-3	immunoglobulin kappa variable 1/OR-3 (pseudogene)
644871	LOC644871	TNF receptor-associated factor 6 pseudogene
644915	METTL15P2	methyltransferase like 15 pseudogene 2
645120	LOC645120	apoptosis-inducing factor, mitochondrion-associated, 1 pseudogene
645166	LOC645166	lymphocyte-specific protein 1 pseudogene
6453	ITSN1	intersectin 1 (SH3 domain protein)

6455	SH3GL1	SH3-domain GRB2-like 1
645545	LOC645545	immunoglobulin (CD79A) binding protein 1 pseudogene
6456	SH3GL2	SH3-domain GRB2-like 2
6457	SH3GL3	SH3-domain GRB2-like 3
64581	CLEC7A	C-type lectin domain family 7, member A
645836	USP17L3	ubiquitin specific peptidase 17-like 3
645939	PRKRIRP8	protein-kinase, interferon-inducible double stranded RNA dependent inhibitor, repressor of (P58 repressor) pseudogene 8
64596	PSMA6P1	proteasome (prosome, macropain) subunit, alpha type, 6 pseudogene 1
646057	LOC646057	ig heavy chain V region 5A-like
646071	LOC646071	disintegrin and metalloproteinase domain-containing protein 21-like
646085	PSMC1P7	proteasome (prosome, macropain) 26S subunit, ATPase, 1 pseudogene 7
646096	LOC646096	protein kinase CHK2-like
6461	SHB	Src homology 2 domain containing adaptor protein B
646278	LOC646278	programmed cell death 6 interacting protein pseudogene
646370	IGHV1OR15-3	immunoglobulin heavy variable 1/OR15-3 (pseudogene)
646379	IGHV1OR15-4	immunoglobulin heavy variable 1/OR15-4 (pseudogene)
6464	SHC1	SHC (Src homology 2 domain containing) transforming protein 1
64641	EBF2	early B-cell factor 2
646430	LOC646430	amyloid P component, serum pseudogene
64651	CSRNP1	cysteine-serine-rich nuclear protein 1
646581	IFNWP2	interferon, omega 1 pseudogene 2
646702	HLA-DPA2	major histocompatibility complex, class II, DP alpha 2 (pseudogene)
6469	SHH	sonic hedgehog
64693	CTAGE1	cutaneous T-cell lymphoma-associated antigen 1
64695	SLEB3	systemic lupus erythematosus susceptibility 3
647069	LOC647069	FK506 binding protein 4, 59kDa pseudogene
64714	PDIA2	protein disulfide isomerase family A, member 2
647187	IGHV3OR16-6	immunoglobulin heavy variable 3/OR16-6 (pseudogene)
64745	METTL17	methyltransferase like 17
64750	SMURF2	SMAD specific E3 ubiquitin protein ligase 2
6477	SIAH1	seven in absentia homolog 1 (Drosophila)
64778	FNDC3B	fibronectin type III domain containing 3B
6478	SIAH2	seven in absentia homolog 2 (Drosophila)
64781	CERK	ceramide kinase
64782	AEN	apoptosis enhancing nuclease
64783	RBM15	RNA binding motif protein 15

64798	DEPTOR	DEP domain containing MTOR-interacting protein
648	BMI1	BMI1 polycomb ring finger oncogene
64805	P2RY12	purinergic receptor P2Y, G-protein coupled, 12
64806	IL25	interleukin 25
64849	SLC13A3	solute carrier family 13 (sodium-dependent dicarboxylate transporter), member 3
64857	PLEKHG2	pleckstrin homology domain containing, family G (with RhoGef domain) member 2
648637	LOC648637	similar to Neutrophil defensin 4 precursor (HNP-4) (HP-4) (Defensin, alpha 4)
64881	PCDH20	protocadherin 20
649	BMP1	bone morphogenetic protein 1
64919	BCL11B	B-cell CLL/lymphoma 11B (zinc finger protein)
6494	SIPA1	signal-induced proliferation-associated 1
6495	SIX1	SIX homeobox 1
6497	SKI	v-ski sarcoma viral oncogene homolog (avian)
64975	MRPL41	mitochondrial ribosomal protein L41
649783	LOC649783	similar to HLA class II histocompatibility antigen, DRB1-8 beta chain precursor (MHC class I antigen DRB1*8) (DR-8) (DR8) (DRw8)
6498	SKIL	SKI-like oncogene
650	BMP2	bone morphogenetic protein 2
65018	PINK1	PTEN induced putative kinase 1
6504	SLAMF1	signaling lymphocytic activation molecule family member 1
6505	SLC1A1	solute carrier family 1 (neuronal/epithelial high affinity glutamate transporter, system Xag), member 1
6506	SLC1A2	solute carrier family 1 (glial high affinity glutamate transporter), member 2
6507	SLC1A3	solute carrier family 1 (glial high affinity glutamate transporter), member 3
65082	VPS33A	vacuolar protein sorting 33 homolog A (S. cerevisiae)
651	BMP3	bone morphogenetic protein 3
6511	SLC1A6	solute carrier family 1 (high affinity aspartate/glutamate transporter), member 6
6512	SLC1A7	solute carrier family 1 (glutamate transporter), member 7
65125	WNK1	WNK lysine deficient protein kinase 1
651536	LOC651536	immunoglobulin iota chain-like
651644	LOC651644	LIM and senescent cell antigen-like domains 2 pseudogene
6517	SLC2A4	solute carrier family 2 (facilitated glucose transporter), member 4
651963	IGHV1OR15-9	immunoglobulin heavy variable 1/OR15-9 (non-functional)
652	BMP4	bone morphogenetic protein 4
6520	SLC3A2	solute carrier family 3 (activators of dibasic and neutral amino acid transport), member 2
6521	SLC4A1	solute carrier family 4, anion exchanger, member 1 (erythrocyte membrane protein band 3, Diego blood group)
652102	LOC652102	similar to Ig heavy chain V-I region HG3 precursor

65217	PCDH15	protocadherin-related 15
6524	SLC5A2	solute carrier family 5 (sodium/glucose cotransporter), member 2
652614	LOC652614	BOLA class I histocompatibility antigen, alpha chain BL3-7-like
65264	UBE2Z	ubiquitin-conjugating enzyme E2Z
65266	WNK4	WNK lysine deficient protein kinase 4
65267	WNK3	WNK lysine deficient protein kinase 3
65268	WNK2	WNK lysine deficient protein kinase 2
652694	LOC652694	similar to Ig kappa chain V-I region HK102 precursor
6527	SLC5A4	solute carrier family 5 (low affinity glucose cotransporter), member 4
652873	IGKV2OR2-7	immunoglobulin kappa variable 2/OR2-7 (pseudogene)
652991	SKOR2	SKI family transcriptional corepressor 2
653	BMP5	bone morphogenetic protein 5
653361	NCF1	neutrophil cytosolic factor 1
653404	FOXD4L6	forkhead box D4-like 6
653406	LOC653406	NLR family, apoptosis inhibitory protein pseudogene
653427	FOXD4L5	forkhead box D4-like 5
653499	LGALS7B	lectin, galactoside-binding, soluble, 7B
653579	CD177P1	CD177 molecule pseudogene 1
653677	SEC1	secretory blood group 1
653712	LOC653712	intraflagellar transport 122 homolog (Chlamydomonas) pseudogene
654	BMP6	bone morphogenetic protein 6
6543	SLC8A2	solute carrier family 8 (sodium/calcium exchanger), member 2
654342	LOC654342	lymphocyte-specific protein 1 pseudogene
6546	SLC8A1	solute carrier family 8 (sodium/calcium exchanger), member 1
6547	SLC8A3	solute carrier family 8 (sodium/calcium exchanger), member 3
654783	IFNNP1	interferon, nu 1, pseudogene
6548	SLC9A1	solute carrier family 9 (sodium/hydrogen exchanger), member 1
655	BMP7	bone morphogenetic protein 7
6550	SLC9A3	solute carrier family 9 (sodium/hydrogen exchanger), member 3
6555	SLC10A2	solute carrier family 10 (sodium/bile acid cotransporter family), member 2
6556	SLC11A1	solute carrier family 11 (proton-coupled divalent metal ion transporters), member 1
656	BMP8B	bone morphogenetic protein 8b
6563	SLC14A1	solute carrier family 14 (urea transporter), member 1 (Kidd blood group)
6565	SLC15A2	solute carrier family 15 (H+/peptide transporter), member 2
6566	SLC16A1	solute carrier family 16, member 1 (monocarboxylic acid transporter 1)
657	BMPR1A	bone morphogenetic protein receptor, type IA
6574	SLC20A1	solute carrier family 20 (phosphate transporter), member 1

6578	SLCO2A1	solute carrier organic anion transporter family, member 2A1
658	BMPR1B	bone morphogenetic protein receptor, type IB
6585	SLIT1	slit homolog 1 (Drosophila)
6586	SLIT3	slit homolog 3 (Drosophila)
659	BMPR2	bone morphogenetic protein receptor, type II (serine/threonine kinase)
6590	SLPI	secretory leukocyte peptidase inhibitor
65978	IGSF6-DREV1	region containing immunoglobulin superfamily, member 6 and DREV1
65985	AACS	acetoacetyl-CoA synthetase
65989	DLK2	delta-like 2 homolog (Drosophila)
660	BMX	BMX non-receptor tyrosine kinase
66001	MLVI4	Moloney leukemia virus integration site 4, mouse, homolog of
6603	SMARCD2	SWI/SNF related, matrix associated, actin dependent regulator of chromatin, subfamily d, member 2
6608	SMO	smoothened, frizzled family receptor
6609	SMPD1	sphingomyelin phosphodiesterase 1, acid lysosomal
661	POLR3D	polymerase (RNA) III (DNA directed) polypeptide D, 44kDa
6610	SMPD2	sphingomyelin phosphodiesterase 2, neutral membrane (neutral sphingomyelinase)
6614	SIGLEC1	sialic acid binding Ig-like lectin 1, sialoadhesin
6615	SNAI1	snail homolog 1 (Drosophila)
6616	SNAP25	synaptosomal-associated protein, 25kDa
662	BNIP1	BCL2/adenovirus E1B 19kDa interacting protein 1
6620	SNCB	synuclein, beta
6622	SNCA	synuclein, alpha (non A4 component of amyloid precursor)
6624	FSCN1	fascin homolog 1, actin-bundling protein (Strongylocentrotus purpuratus)
663	BNIP2	BCL2/adenovirus E1B 19kDa interacting protein 2
664	BNIP3	BCL2/adenovirus E1B 19kDa interacting protein 3
6642	SNX1	sorting nexin 1
6643	SNX2	sorting nexin 2
6646	SOAT1	sterol O-acyltransferase 1
6647	SOD1	superoxide dismutase 1, soluble
6648	SOD2	superoxide dismutase 2, mitochondrial
665	BNIP3L	BCL2/adenovirus E1B 19kDa interacting protein 3-like
6651	SON	SON DNA binding protein
6653	SORL1	sortilin-related receptor, L(DLR class) A repeats containing
6654	SOS1	son of sevenless homolog 1 (Drosophila)
6655	SOS2	son of sevenless homolog 2 (Drosophila)
6657	SOX2	SRY (sex determining region Y)-box 2
6659	SOX4	SRY (sex determining region Y)-box 4

666	BOK	BCL2-related ovarian killer
6662	SOX9	SRY (sex determining region Y)-box 9
667	DST	dystonin
6670	SP3	Sp3 transcription factor
6672	SP100	SP100 nuclear antigen
6677	SPAM1	sperm adhesion molecule 1 (PH-20 hyaluronidase, zona pellucida binding)
6678	SPARC	secreted protein, acidic, cysteine-rich (osteonectin)
668	FOXL2	forkhead box L2
6683	SPAST	spastin
6688	SPI1	spleen focus forming virus (SFFV) proviral integration oncogene spi1
6689	SPIB	Spi-B transcription factor (Spi-1/PU.1 related)
6693	SPN	sialophorin
6695	SPOCK1	sparc/osteonectin, cwcv and kazal-like domains proteoglycan (testican) 1
6696	SPP1	secreted phosphoprotein 1
6709	SPTAN1	spectrin, alpha, non-erythrocytic 1 (alpha-fodrin)
671	BPI	bactericidal/permeability-increasing protein
6714	SRC	v-src sarcoma (Schmidt-Ruppin A-2) viral oncogene homolog (avian)
672	BRCA1	breast cancer 1, early onset
6722	SRF	serum response factor (c-fos serum response element-binding transcription factor)
673	BRAF	v-raf murine sarcoma viral oncogene homolog B1
6732	SRPK1	SRSF protein kinase 1
6733	SRPK2	SRSF protein kinase 2
6739	SSAV1	simian sarcoma-associated virus 1/gibbon ape leukemia virus-related endogenous retroviral element 1
675	BRCA2	breast cancer 2, early onset
6750	SST	somatostatin
6753	SSTR3	somatostatin receptor 3
6760	SS18	synovial sarcoma translocation, chromosome 18
677	ZFP36L1	zinc finger protein 36, C3H type-like 1
6770	STAR	steroidogenic acute regulatory protein
6772	STAT1	signal transducer and activator of transcription 1, 91kDa
6773	STAT2	signal transducer and activator of transcription 2, 113kDa
6774	STAT3	signal transducer and activator of transcription 3 (acute-phase response factor)
6775	STAT4	signal transducer and activator of transcription 4
6776	STAT5A	signal transducer and activator of transcription 5A
6777	STAT5B	signal transducer and activator of transcription 5B
6778	STAT6	signal transducer and activator of transcription 6, interleukin-4 induced
6786	STIM1	stromal interaction molecule 1

6788	STK3	serine/threonine kinase 3
6789	STK4	serine/threonine kinase 4
6794	STK11	serine/threonine kinase 11
6795	AURKC	aurora kinase C
6804	STX1A	syntaxin 1A (brain)
6809	STX3	syntaxin 3
6810	STX4	syntaxin 4
6812	STXBP1	syntaxin binding protein 1
6813	STXBP2	syntaxin binding protein 2
6814	STXBP3	syntaxin binding protein 3
682	BSG	basigin (Ok blood group)
683	BST1	bone marrow stromal cell antigen 1
6833	ABCC8	ATP-binding cassette, sub-family C (CFTR/MRP), member 8
684	BST2	bone marrow stromal cell antigen 2
6844	VAMP2	vesicle-associated membrane protein 2 (synaptobrevin 2)
6845	VAMP7	vesicle-associated membrane protein 7
6846	XCL2	chemokine (C motif) ligand 2
685	BTC	betacellulin
6850	SYK	spleen tyrosine kinase
6857	SYT1	synaptotagmin I
6863	TAC1	tachykinin, precursor 1
6868	ADAM17	ADAM metallopeptidase domain 17
6869	TACR1	tachykinin receptor 1
6880	TAF9	TAF9 RNA polymerase II, TATA box binding protein (TBP)-associated factor, 32kDa
6885	MAP3K7	mitogen-activated protein kinase kinase kinase 7
6886	TAL1	T-cell acute lymphocytic leukemia 1
6887	TAL2	T-cell acute lymphocytic leukemia 2
6890	TAP1	transporter 1, ATP-binding cassette, sub-family B (MDR/TAP)
6891	TAP2	transporter 2, ATP-binding cassette, sub-family B (MDR/TAP)
6892	TAPBP	TAP binding protein (tapasin)
6894	TARBP1	TAR (HIV-1) RNA binding protein 1
6895	TARBP2	TAR (HIV-1) RNA binding protein 2
6896	TARBP2P	TAR (HIV-1) RNA binding protein 2 pseudogene
6899	TBX1	T-box 1
6900	CNTN2	contactin 2 (axonal)
6906	SERPINA7	serpin peptidase inhibitor, clade A (alpha-1 antiproteinase, antitrypsin), member 7
6910	TBX5	T-box 5

6915	TBXA2R	thromboxane A2 receptor
6916	TBXAS1	thromboxane A synthase 1 (platelet)
6926	TBX3	T-box 3
6928	HNF1B	HNF1 homeobox B
6929	TCF3	transcription factor 3 (E2A immunoglobulin enhancer binding factors E12/E47)
6932	TCF7	transcription factor 7 (T-cell specific, HMG-box)
6934	TCF7L2	transcription factor 7-like 2 (T-cell specific, HMG-box)
6935	ZEB1	zinc finger E-box binding homeobox 1
694	BTG1	B-cell translocation gene 1, anti-proliferative
6943	TCF21	transcription factor 21
6946	TCL4	T-cell leukemia/lymphoma 4
695	BTK	Bruton agammaglobulinemia tyrosine kinase
6955	TRA@	T cell receptor alpha locus
6956	TRAV6	T cell receptor alpha variable 6
6957	TRB@	T cell receptor beta locus
6958	TRBV29OR9-2	T cell receptor beta variable 29/OR9-2 (non-functional)
6959	TRBV21OR9-2	T cell receptor beta variable 21/OR9-2 (non-functional)
6960	TRBV25OR9-2	T cell receptor beta variable 25/OR9-2 (pseudogene)
6961	TRBV24OR9-2	T cell receptor beta variable 24/OR9-2 (pseudogene)
6962	TRBV20OR9-2	T cell receptor beta variable 20/OR9-2 (non-functional)
6964	TRD@	T cell receptor delta locus
6965	TRG@	T cell receptor gamma locus
6966	TRGC1	T cell receptor gamma constant 1
6967	TRGC2	T cell receptor gamma constant 2
6968	TRGJ1	T cell receptor gamma joining 1
6969	TRGJ2	T cell receptor gamma joining 2
6970	TRGJP	T cell receptor gamma joining P
6971	TRGJP1	T cell receptor gamma joining P1
6972	TRGJP2	T cell receptor gamma joining P2
6973	TRGV1	T cell receptor gamma variable 1 (non-functional)
6974	TRGV2	T cell receptor gamma variable 2
6976	TRGV3	T cell receptor gamma variable 3
6977	TRGV4	T cell receptor gamma variable 4
6978	TRGV5	T cell receptor gamma variable 5
6979	TRGV5P	T cell receptor gamma variable 5P (pseudogene)
6980	TRGV6	T cell receptor gamma variable 6 (pseudogene)
6981	TRGV7	T cell receptor gamma variable 7 (pseudogene)

6982	TRGV8	T cell receptor gamma variable 8
6983	TRGV9	T cell receptor gamma variable 9
6984	TRGV10	T cell receptor gamma variable 10 (non-functional)
6985	TRGV11	T cell receptor gamma variable 11 (non-functional)
6986	TRGVA	T cell receptor gamma variable A (pseudogene)
6987	TRGVB	T cell receptor gamma variable B (pseudogene)
6988	TCTA	T-cell leukemia translocation altered gene
699	BUB1	budding uninhibited by benzimidazoles 1 homolog (yeast)
6993	DYNLT1	dynein, light chain, Tctex-type 1
6997	TDGF1	teratocarcinoma-derived growth factor 1
6998	TDGF3	teratocarcinoma-derived growth factor 3, pseudogene
70	ACTC1	actin, alpha, cardiac muscle 1
700	BVR1	Burkitt lymphoma variant rearranging region 1
7001	PRDX2	peroxiredoxin 2
7006	TEC	tec protein tyrosine kinase
7009	TMBIM6	transmembrane BAX inhibitor motif containing 6
701	BUB1B	budding uninhibited by benzimidazoles 1 homolog beta (yeast)
7010	TEK	TEK tyrosine kinase, endothelial
7013	TERF1	telomeric repeat binding factor (NIMA-interacting) 1
7015	TERT	telomerase reverse transcriptase
7018	TF	transferrin
7023	TFAP4	transcription factor AP-4 (activating enhancer binding protein 4)
7026	NR2F2	nuclear receptor subfamily 2, group F, member 2
7037	TFRC	transferrin receptor (p90, CD71)
7038	TG	thyroglobulin
7039	TGFA	transforming growth factor, alpha
7040	TGFB1	transforming growth factor, beta 1
7041	TGFB1I1	transforming growth factor beta 1 induced transcript 1
7042	TGFB2	transforming growth factor, beta 2
7043	TGFB3	transforming growth factor, beta 3
7044	LEFTY2	left-right determination factor 2
7045	TGFBI	transforming growth factor, beta-induced, 68kDa
7046	TGFBR1	transforming growth factor, beta receptor 1
7048	TGFBR2	transforming growth factor, beta receptor II (70/80kDa)
7049	TGFBR3	transforming growth factor, beta receptor III
705	BYSL	bystin-like
7052	TGM2	transglutaminase 2 (C polypeptide, protein-glutamine-gamma-glutamyltransferase)

7056	THBD	thrombomodulin
7057	THBS1	thrombospondin 1
7058	THBS2	thrombospondin 2
7059	THBS3	thrombospondin 3
706	TSPO	translocator protein (18kDa)
7060	THBS4	thrombospondin 4
7064	THOP1	thimet oligopeptidase 1
7066	THPO	thrombopoietin
7068	THRB	thyroid hormone receptor, beta (erythroblastic leukemia viral (v-erb-a) oncogene homolog 2, avian)
7070	THY1	Thy-1 cell surface antigen
7071	KLF10	Kruppel-like factor 10
7072	TIA1	TIA1 cytotoxic granule-associated RNA binding protein
7073	TIAL1	TIA1 cytotoxic granule-associated RNA binding protein-like 1
7074	TIAM1	T-cell lymphoma invasion and metastasis 1
7075	TIE1	tyrosine kinase with immunoglobulin-like and EGF-like domains 1
7076	TIMP1	TIMP metallopeptidase inhibitor 1
7077	TIMP2	TIMP metallopeptidase inhibitor 2
7079	TIMP4	TIMP metallopeptidase inhibitor 4
708	C1QBP	complement component 1, q subcomponent binding protein
7080	NKX2-1	NK2 homeobox 1
7082	TJP1	tight junction protein 1 (zona occludens 1)
7087	ICAM5	intercellular adhesion molecule 5, telencephalin
709	C1HR	C1AGOH temperature sensitivity complementing
7094	TLN1	talin 1
7096	TLR1	toll-like receptor 1
7097	TLR2	toll-like receptor 2
7098	TLR3	toll-like receptor 3
7099	TLR4	toll-like receptor 4
710	SERPING1	serpin peptidase inhibitor, clade G (C1 inhibitor), member 1
7100	TLR5	toll-like receptor 5
7101	NR2E1	nuclear receptor subfamily 2, group E, member 1
7114	TMSB4X	thymosin beta 4, X-linked
712	C1QA	complement component 1, q subcomponent, A chain
7122	CLDN5	claudin 5
7124	TNF	tumor necrosis factor
7125	TNNC2	troponin C type 2 (fast)
7126	TNFAIP1	tumor necrosis factor, alpha-induced protein 1 (endothelial)

7127	TNFAIP2	tumor necrosis factor, alpha-induced protein 2
7128	TNFAIP3	tumor necrosis factor, alpha-induced protein 3
713	C1QB	complement component 1, q subcomponent, B chain
7130	TNFAIP6	tumor necrosis factor, alpha-induced protein 6
7132	TNFRSF1A	tumor necrosis factor receptor superfamily, member 1A
7133	TNFRSF1B	tumor necrosis factor receptor superfamily, member 1B
7136	TNNI2	troponin I type 2 (skeletal, fast)
714	C1QC	complement component 1, q subcomponent, C chain
7140	TNNT3	troponin T type 3 (skeletal, fast)
7143	TNR	tenascin R (restrictin, janusin)
7148	TNXB	tenascin XB
715	C1R	complement component 1, r subcomponent
7150	TOP1	topoisomerase (DNA) I
7153	TOP2A	topoisomerase (DNA) II alpha 170kDa
7155	TOP2B	topoisomerase (DNA) II beta 180kDa
7157	TP53	tumor protein p53
7159	TP53BP2	tumor protein p53 binding protein, 2
716	C1S	complement component 1, s subcomponent
7161	TP73	tumor protein p73
7162	TPBG	trophoblast glycoprotein
7163	TPD52	tumor protein D52
7164	TPD52L1	tumor protein D52-like 1
7168	TPM1	tropomyosin 1 (alpha)
717	C2	complement component 2
7178	TPT1	tumor protein, translationally-controlled 1
718	C3	complement component 3
7184	HSP90B1	heat shock protein 90kDa beta (Grp94), member 1
7185	TRAF1	TNF receptor-associated factor 1
7186	TRAF2	TNF receptor-associated factor 2
7187	TRAF3	TNF receptor-associated factor 3
7188	TRAF5	TNF receptor-associated factor 5
7189	TRAF6	TNF receptor-associated factor 6
719	C3AR1	complement component 3a receptor 1
720	C4A	complement component 4A (Rodgers blood group)
7204	TRIO	triple functional domain (PTPRF interacting)
7205	TRIP6	thyroid hormone receptor interactor 6
721	C4B	complement component 4B (Chido blood group)

7216	TRO	trophinin
722	C4BPA	complement component 4 binding protein, alpha
7222	TRPC3	transient receptor potential cation channel, subfamily C, member 3
7225	TRPC6	transient receptor potential cation channel, subfamily C, member 6
723788	MIG7	mig-7
723805	LOC723805	interleukin-like
723961	INS-IGF2	INS-IGF2 readthrough
724	C4BPAP2	complement component 4 binding protein, alpha pseudogene 2
724067	DEFA7P	defensin, alpha 7 pseudogene
724068	DEFA11P	defensin, alpha 11 pseudogene
7249	TSC2	tuberous sclerosis 2
725	C4BPB	complement component 4 binding protein, beta
7253	TSHR	thyroid stimulating hormone receptor
7262	PHLDA2	pleckstrin homology-like domain, family A, member 2
7264	TSTA3	tissue specific transplantation antigen P35B
727	C5	complement component 5
7271	TTIM1	T-cell tumor invasion and metastasis 1
7273	TTN	titin
7275	TUB	tubby homolog (mouse)
7277	TUBA4A	tubulin, alpha 4a
727738	AREGB	amphiregulin B
727787	LOC727787	killer cell immunoglobulin-like receptor, three domains, long cytoplasmic tail, 2-like
727859	C4BPAP1	complement component 4 binding protein, alpha pseudogene 1
727897	MUC5B	mucin 5B, oligomeric mucus/gel-forming
727910	TLCD2	TLC domain containing 2
728	C5AR1	complement component 5a receptor 1
728045	PPBPL1	pro-platelet basic protein-like 1
728048	LOC728048	interferon induced transmembrane protein pseudogene
728194	RSPH10B2	radial spoke head 10 homolog B2 (Chlamydomonas)
728297	LOC728297	prostaglandin E2 receptor EP4 subtype-like
728358	DEFA1B	defensin, alpha 1B
728386	USP17L5	ubiquitin specific peptidase 17-like 5
728519	LOC728519	NLR family, apoptosis inhibitory protein pseudogene
728535	LOC728535	NLR family, apoptosis inhibitory protein pseudogene
728577	CNTNAP3B	contactin associated protein-like 3B
728599	CIAPIN1P	cytokine induced apoptosis inhibitor 1 pseudogene
728613	LOC728613	programmed cell death 6 pseudogene

728642	CDK11A	cyclin-dependent kinase 11A
7287	TULP1	tubby like protein 1
728739	LOC728739	programmed cell death 2 pseudogene
728748	PRKRIRP1	protein-kinase, interferon-inducible double stranded RNA dependent inhibitor, repressor of (P58 repressor) pseudogene 1
728815	LOC728815	cytokine receptor-like factor 3 pseudogene
729	C6	complement component 6
729008	LOC729008	putative apoptosis-related protein 2-like
7291	TWIST1	twist homolog 1 (Drosophila)
7292	TNFSF4	tumor necrosis factor (ligand) superfamily, member 4
729230	CCR2	chemokine (C-C motif) receptor 2
7293	TNFRSF4	tumor necrosis factor receptor superfamily, member 4
729412	PSMA6P2	proteasome (prosome, macropain) subunit, alpha type, 6 pseudogene 2
729486	IL9RP3	interleukin 9 receptor pseudogene 3
729650	METTL15P3	methyltransferase like 15 pseudogene 3
729674	LOC729674	proteasome (prosome, macropain) 26S subunit, ATPase, 2 pseudogene
7297	TYK2	tyrosine kinase 2
729767	CEACAM18	carcinoembryonic antigen-related cell adhesion molecule 18
7298	TYMS	thymidylate synthetase
7299	TYR	tyrosinase (oculocutaneous albinism IA)
730	C7	complement component 7
7301	TYRO3	TYRO3 protein tyrosine kinase
730130	TMEM229A	transmembrane protein 229A
730249	IRG1	immunoresponsive 1 homolog (mouse)
7305	TYROBP	TYRO protein tyrosine kinase binding protein
731	C8A	complement component 8, alpha polypeptide
731062	LOC731062	similar to Immunoglobulin omega chain precursor (VpreB2 protein)
7311	UBA52	ubiquitin A-52 residue ribosomal protein fusion product 1
7314	UBB	ubiquitin B
7316	UBC	ubiquitin C
731716	PRKRAP1	protein kinase, interferon-inducible double stranded RNA dependent activator pseudogene 1
731751	LOC731751	DNA-dependent protein kinase catalytic subunit-like
7318	UBA7	ubiquitin-like modifier activating enzyme 7
732	C8B	complement component 8, beta polypeptide
7320	UBE2B	ubiquitin-conjugating enzyme E2B
7321	UBE2D1	ubiquitin-conjugating enzyme E2D 1
7323	UBE2D3	ubiquitin-conjugating enzyme E2D 3

733	C8G	complement component 8, gamma polypeptide
7334	UBE2N	ubiquitin-conjugating enzyme E2N
7335	UBE2V1	ubiquitin-conjugating enzyme E2 variant 1
7337	UBE3A	ubiquitin protein ligase E3A
7341	SUMO1	SMT3 suppressor of mif two 3 homolog 1 (S. cerevisiae)
7342	UBP1	upstream binding protein 1 (LBP-1a)
7349	UCN	urocortin
735	C9	complement component 9
7350	UCP1	uncoupling protein 1 (mitochondrial, proton carrier)
7356	SCGB1A1	secretoglobin, family 1A, member 1 (uteroglobin)
7369	UMOD	uromodulin
7373	COL14A1	collagen, type XIV, alpha 1
7374	UNG	uracil-DNA glycosylase
7375	USP4	ubiquitin specific peptidase 4 (proto-oncogene)
7376	NR1H2	nuclear receptor subfamily 1, group H, member 2
7408	VASP	vasodilator-stimulated phosphoprotein
7409	VAV1	vav 1 guanine nucleotide exchange factor
7410	VAV2	vav 2 guanine nucleotide exchange factor
7412	VCAM1	vascular cell adhesion molecule 1
7414	VCL	vinculin
7415	VCP	valosin containing protein
7416	VDAC1	voltage-dependent anion channel 1
7421	VDR	vitamin D (1,25- dihydroxyvitamin D3) receptor
7422	VEGFA	vascular endothelial growth factor A
7423	VEGFB	vascular endothelial growth factor B
7424	VEGFC	vascular endothelial growth factor C
7428	VHL	von Hippel-Lindau tumor suppressor
7429	VIL1	villin 1
7430	EZR	ezrin
7431	VIM	vimentin
7436	VLDLR	very low density lipoprotein receptor
7441	VPREB1	pre-B lymphocyte 1
7442	TRPV1	transient receptor potential cation channel, subfamily V, member 1
7448	VTN	vitronectin
7450	VWF	von Willebrand factor
7454	WAS	Wiskott-Aldrich syndrome (eczema-thrombocytopenia)
7455	ZAN	zonadhesin

7462	LAT2	linker for activation of T cells family, member 2
7466	WFS1	Wolfram syndrome 1 (wolframin)
747	DAGLA	diacylglycerol lipase, alpha
7471	WNT1	wingless-type MMTV integration site family, member 1
7472	WNT2	wingless-type MMTV integration site family member 2
7473	WNT3	wingless-type MMTV integration site family, member 3
7474	WNT5A	wingless-type MMTV integration site family, member 5A
7476	WNT7A	wingless-type MMTV integration site family, member 7A
7477	WNT7B	wingless-type MMTV integration site family, member 7B
7478	WNT8A	wingless-type MMTV integration site family, member 8A
7480	WNT10B	wingless-type MMTV integration site family, member 10B
7481	WNT11	wingless-type MMTV integration site family, member 11
7483	WNT9A	wingless-type MMTV integration site family, member 9A
7486	WRN	Werner syndrome, RecQ helicase-like
7490	WT1	Wilms tumor 1
7499	XG	Xg blood group
7501	XGR	XG and CD99 regulator
7504	XK	X-linked Kx blood group (McLeod syndrome)
7507	XPA	xeroderma pigmentosum, complementation group A
7508	XPC	xeroderma pigmentosum, complementation group C
751071	METTL12	methyltransferase like 12
7514	XPO1	exportin 1 (CRM1 homolog, yeast)
7515	XRCC1	X-ray repair complementing defective repair in Chinese hamster cells 1
7516	XRCC2	X-ray repair complementing defective repair in Chinese hamster cells 2
7517	XRCC3	X-ray repair complementing defective repair in Chinese hamster cells 3
7518	XRCC4	X-ray repair complementing defective repair in Chinese hamster cells 4
752	FMNL1	formin-like 1
7520	XRCC5	X-ray repair complementing defective repair in Chinese hamster cells 5 (double-strand-break rejoining)
7521	XRCC8	X-ray repair complementing defective repair in Chinese hamster cells 8
7525	YES1	v-yes-1 Yamaguchi sarcoma viral oncogene homolog 1
7529	YWHAB	tyrosine 3-monooxygenase/tryptophan 5-monooxygenase activation protein, beta polypeptide
7531	YWHAE	tyrosine 3-monooxygenase/tryptophan 5-monooxygenase activation protein, epsilon polypeptide
7532	YWHAG	tyrosine 3-monooxygenase/tryptophan 5-monooxygenase activation protein, gamma polypeptide
7534	YWHAZ	tyrosine 3-monooxygenase/tryptophan 5-monooxygenase activation protein, zeta polypeptide
7535	ZAP70	zeta-chain (TCR) associated protein kinase 70kDa
7538	ZFP36	zinc finger protein 36, C3H type, homolog (mouse)
7551	ZNF3	zinc finger protein 3

7593	MZF1	myeloid zinc finger 1
7703	PCGF2	polycomb group ring finger 2
7704	ZBTB16	zinc finger and BTB domain containing 16
7706	TRIM25	tripartite motif containing 25
7707	ZNF148	zinc finger protein 148
7716	VEZF1	vascular endothelial zinc finger 1
773	CACNA1A	calcium channel, voltage-dependent, P/Q type, alpha 1A subunit
7763	ZFAND5	zinc finger, AN1-type domain 5
7784	ZP3	zona pellucida glycoprotein 3 (sperm receptor)
7786	MAP3K12	mitogen-activated protein kinase kinase kinase 12
7791	ZYX	zyxin
7795	MEMO1	Methylation modifier for class I HLA
780	DDR1	discoidin domain receptor tyrosine kinase 1
7804	LRP8	low density lipoprotein receptor-related protein 8, apolipoprotein e receptor
780917	NKCD	Natural killer cell deficiency, familial isolated
7818	DAP3	death associated protein 3
7832	BTG2	BTG family, member 2
7833	SLEB1	systemic lupus erythematosus susceptibility 1
7837	PXDN	peroxidasin homolog (Drosophila)
7839	LSL	Leptin, serum levels of
784	CACNB3	calcium channel, voltage-dependent, beta 3 subunit
7840	ALMS1	Alstrom syndrome 1
7842	IGKJ@	immunoglobulin kappa joining group
7845	PAFAH1B1P1	platelet-activating factor acetylhydrolase 1b, regulatory subunit 1 pseudogene 1
7849	PAX8	paired box 8
7850	IL1R2	interleukin 1 receptor, type II
7851	MALL	mal, T-cell differentiation protein-like
7852	CXCR4	chemokine (C-X-C motif) receptor 4
7855	FZD5	frizzled family receptor 5
7857	SCG2	secretogranin II
7866	IFRD2	interferon-related developmental regulator 2
7869	SEMA3B	sema domain, immunoglobulin domain (Ig), short basic domain, secreted, (semaphorin) 3B
7874	USP7	ubiquitin specific peptidase 7 (herpes virus-associated)
7879	RAB7A	RAB7A, member RAS oncogene family
7895	LAG5	Leukocyte antigen group 5
7896	TS13	Temperature sensitivity complementation, ts13
79031	PDCL3	phosducin-like 3

79037	PVRIG	poliovirus receptor related immunoglobulin domain containing
79066	METTL16	methyltransferase like 16
79068	FTO	fat mass and obesity associated
79072	FASTKD3	FAST kinase domains 3
7908	EOS	eosinophilia, familial
79092	CARD14	caspase recruitment domain family, member 14
79094	CHAC1	ChaC, cation transport regulator homolog 1 (E. coli)
790953	TSL	testis-expressed, seven-twelve, leukemia
79109	MAPKAP1	mitogen-activated protein kinase associated protein 1
79136	LY6G6E	lymphocyte antigen 6 complex, locus G6E
79139	DERL1	Der1-like domain family, member 1
79155	TNIP2	TNFAIP3 interacting protein 2
79156	PLEKHF1	pleckstrin homology domain containing, family F (with FYVE domain) member 1
79165	LENG1	leukocyte receptor cluster (LRC) member 1
79166	LILRP2	leukocyte immunoglobulin-like receptor pseudogene 2
79167	LILRP1	leukocyte immunoglobulin-like receptor pseudogene 1
79168	LILRA6	leukocyte immunoglobulin-like receptor, subfamily A (with TM domain), member 6
7917	BAG6	BCL2-associated athanogene 6
7921	D6S207E	Minor histocompatibility antigen HA-2
79363	RSG1	REM2 and RAB-like small GTPase 1
79368	FCRL2	Fc receptor-like 2
79370	BCL2L14	BCL2-like 14 (apoptosis facilitator)
7938	CP20	Lymphocyte cytosolic protein, molecular weight 20kD
7940	LST1	leukocyte specific transcript 1
79400	NOX5	NADPH oxidase, EF-hand calcium binding domain 5
7941	PLA2G7	phospholipase A2, group VII (platelet-activating factor acetylhydrolase, plasma)
79414	LRFN3	leucine rich repeat and fibronectin type III domain containing 3
79444	BIRC7	baculoviral IAP repeat containing 7
79465	ULBP3	UL16 binding protein 3
79469	DLEU2L	deleted in lymphocytic leukemia 2-like
7952	TNDM	diabetes mellitus, transient neonatal
7955	STL	six-twelve leukemia
7956	NTT	Noncoding transcript in T cells
79576	NKAP	NFKB activating protein
79589	RNF128	ring finger protein 128
79594	MUL1	mitochondrial E3 ubiquitin protein ligase 1
796	CALCA	calcitonin-related polypeptide alpha

79626	TNFAIP8L2	tumor necrosis factor, alpha-induced protein 8-like 2
7963	TS546	Temperature sensitivity complementation, cell cycle specific
79633	FAT4	FAT tumor suppressor homolog 4 (Drosophila)
7965	AIMP2	aminoacyl tRNA synthetase complex-interacting multifunctional protein 2
79652	TMEM204	transmembrane protein 204
79658	ARHGAP10	Rho GTPase activating protein 10
79671	NLRX1	NLR family member X1
79675	FASTKD1	FAST kinase domains 1
79679	VTCN1	V-set domain containing T cell activation inhibitor 1
79689	STEAP4	STEAP family member 4
79709	GLT25D1	glycosyltransferase 25 domain containing 1
79767	ELMO3	engulfment and cell motility 3
7979	SHFM1	split hand/foot malformation (ectrodactyly) type 1
79810	PTCD2	pentatricopeptide repeat domain 2
79811	SLTM	SAFB-like, transcription modulator
79834	PEAK1	NKF3 kinase family member
79840	NHEJ1	nonhomologous end-joining factor 1
79858	NEK11	NIMA (never in mitosis gene a)- related kinase 11
79865	TREML2	triggering receptor expressed on myeloid cells-like 2
79870	BAALC	brain and acute leukemia, cytoplasmic
79872	CBLL1	Cas-Br-M (murine) ecotropic retroviral transforming sequence-like 1
79874	RABEP2	rabaptin, RAB GTPase binding effector protein 2
79890	RIN3	Ras and Rab interactor 3
79902	NUP85	nucleoporin 85kDa
79931	TNIP3	TNFAIP3 interacting protein 3
79937	CNTNAP3	contactin associated protein-like 3
7994	MYST3	MYST histone acetyltransferase (monocytic leukemia) 3
79960	PHF17	PHD finger protein 17
79966	SCD5	stearoyl-CoA desaturase 5
79987	SVEP1	sushi, von Willebrand factor type A, EGF and pentraxin domain containing 1
80005	DOCK5	dedicator of cytokinesis 5
80013	FAM188A	family with sequence similarity 188, member A
80034	CSRNP3	cysteine-serine-rich nuclear protein 3
80070	ADAMTS20	ADAM metallopeptidase with thrombospondin type 1 motif, 20
8009	LALL	Lymphomatous acute lymphoblastic leukemia
8013	NR4A3	nuclear receptor subfamily 4, group A, member 3
80139	ZNF703	zinc finger protein 703

80142	PTGES2	prostaglandin E synthase 2
80149	ZC3H12A	zinc finger CCCH-type containing 12A
80162	ATHL1	ATH1, acid trehalase-like 1 (yeast)
80173	IFT74	intraflagellar transport 74 homolog (Chlamydomonas)
80196	RNF34	ring finger protein 34
80221	ACSF2	acyl-CoA synthetase family member 2
80227	PAAF1	proteasomal ATPase-associated factor 1
8023	H142T	Temperature sensitivity complementation, H142
80230	RUFY1	RUN and FYVE domain containing 1
8026	DGS2	DiGeorge syndrome chromosome region-2
8028	MLLT10	myeloid/lymphoid or mixed-lineage leukemia (trithorax homolog, Drosophila); translocated to, 10
8029	CUBN	cubilin (intrinsic factor-cobalamin receptor)
80310	PDGFD	platelet derived growth factor D
80326	WNT10A	wingless-type MMTV integration site family, member 10A
80328	ULBP2	UL16 binding protein 2
80329	ULBP1	UL16 binding protein 1
80331	DNAJC5	DnaJ (Hsp40) homolog, subfamily C, member 5
8038	ADAM12	ADAM metallopeptidase domain 12
80380	PDCD1LG2	programmed cell death 1 ligand 2
80381	CD276	CD276 molecule
8039	PROA	Proline(-) auxotroph, complementation of
8041	TP250	T-cell activation antigen p250
8061	FOSL1	FOS-like antigen 1
8065	CUL5	cullin 5
80714	PBX4	pre-B-cell leukemia homeobox 4
80725	SRCIN1	SRC kinase signaling inhibitor 1
80740	LY6G6C	lymphocyte antigen 6 complex, locus G6C
80741	LY6G5C	lymphocyte antigen 6 complex, locus G5C
80762	NDFIP1	Nedd4 family interacting protein 1
80781	COL18A1	collagen, type XVIII, alpha 1
8079	MLF2	myeloid leukemia factor 2
808	CALM3	calmodulin 3 (phosphorylase kinase, delta)
8082	SSPN	sarcospan (Kras oncogene-associated gene)
8085	MLL2	myeloid/lymphoid or mixed-lineage leukemia 2
80867	HCG2P7	HLA complex group 2 pseudogene 7
80868	HCG4B	HLA complex group 4B (non-protein coding)
8087	FXR1	fragile X mental retardation, autosomal homolog 1

80895	ILKAP	integrin-linked kinase-associated serine/threonine phosphatase
81	ACTN4	actinin, alpha 4
8100	IFT88	intraflagellar transport 88 homolog (Chlamydomonas)
8101	D13S25	Disrupted in B-cell neoplasia
81029	WNT5B	wingless-type MMTV integration site family, member 5B
81030	ZBP1	Z-DNA binding protein 1
81035	COLEC12	collectin sub-family member 12
81037	CLPTM1L	CLPTM1-like
8106	PABPN1	poly(A) binding protein, nuclear 1
811	CALR	calreticulin
8115	TCL1A	T-cell leukemia/lymphoma 1A
8120	AP3B2	adaptor-related protein complex 3, beta 2 subunit
813	CALU	calumenin
8140	SLC7A5	solute carrier family 7 (cationic amino acid transporter, y+ system), member 5
8144	FIMG1	myasthenia gravis, familial infantile, 1
81492	RSPH6A	radial spoke head 6 homolog A (Chlamydomonas)
81494	CFHR5	complement factor H-related 5
815	CAMK2A	calcium/calmodulin-dependent protein kinase II alpha
81502	HM13	histocompatibility (minor) 13
81533	ITFG1	integrin alpha FG-GAP repeat containing 1
81537	SGPP1	sphingosine-1-phosphate phosphatase 1
81542	TMX1	thioredoxin-related transmembrane protein 1
81566	CSRNP2	cysteine-serine-rich nuclear protein 2
81567	TXNDC5	thioredoxin domain containing 5 (endoplasmic reticulum)
81578	COL21A1	collagen, type XXI, alpha 1
816	CAMK2B	calcium/calmodulin-dependent protein kinase II beta
81607	PVRL4	poliovirus receptor-related 4
81618	ITM2C	integral membrane protein 2C
81623	DEFB126	defensin, beta 126
817	CAMK2D	calcium/calmodulin-dependent protein kinase II delta
81704	DOCK8	dedicator of cytokinesis 8
8174	MADCAM1	mucosal vascular addressin cell adhesion molecule 1
81788	NUAK2	NUAK family, SNF1-like kinase, 2
81793	TLR10	toll-like receptor 10
818	CAMK2G	calcium/calmodulin-dependent protein kinase II gamma
81844	TRIM56	tripartite motif containing 56
81858	SHARPIN	SHANK-associated RH domain interactor

81875	ISG20L2	interferon stimulated exonuclease gene 20kDa-like 2
8189	SYMPK	symplekin
819	CAMLG	calcium modulating ligand
8193	DPF1	D4, zinc and double PHD fingers family 1
8195	MKKS	McKusick-Kaufman syndrome
820	CAMP	cathelicidin antimicrobial peptide
8200	GDF5	growth differentiation factor 5
8201	MLRG	Myeloid leukemia-related gene (myeloid tumor suppressor)
8214	DGCR6	DiGeorge syndrome critical region gene 6
8217	IGLJ@	immunoglobulin lambda joining group
8218	CLTCL1	clathrin, heavy chain-like 1
8220	DGCR14	DiGeorge syndrome critical region gene 14
8227	AKAP17A	A kinase (PRKA) anchor protein 17A
8239	USP9X	ubiquitin specific peptidase 9, X-linked
8245	IDDMX	Diabetes mellitus, insulin-dependent, X-linked, susceptibility to
8277	TKTL1	transketolase-like 1
8287	USP9Y	ubiquitin specific peptidase 9, Y-linked
8288	EPX	eosinophil peroxidase
8301	PICALM	phosphatidylinositol binding clathrin assembly protein
8302	KLRC4	killer cell lectin-like receptor subfamily C, member 4
8312	AXIN1	axin 1
8320	EOMES	eomesodermin
8322	FZD4	frizzled family receptor 4
8324	FZD7	frizzled family receptor 7
8325	FZD8	frizzled family receptor 8
83259	PCDH11Y	protocadherin 11 Y-linked
8326	FZD9	frizzled family receptor 9
834	CASP1	caspase 1, apoptosis-related cysteine peptidase (interleukin 1, beta, convertase)
83416	FCRL5	Fc receptor-like 5
83417	FCRL4	Fc receptor-like 4
83439	TCF7L1	transcription factor 7-like 1 (T-cell specific, HMG-box)
83464	APH1B	anterior pharynx defective 1 homolog B (C. elegans)
835	CASP2	caspase 2, apoptosis-related cysteine peptidase
83543	AIF1L	allograft inflammatory factor 1-like
83591	THAP2	THAP domain containing, apoptosis associated protein 2
83593	RASSF5	Ras association (RalGDS/AF-6) domain family member 5
83595	SOX7	SRY (sex determining region Y)-box 7

83596	BCL2L12	BCL2-like 12 (proline rich)
836	CASP3	caspase 3, apoptosis-related cysteine peptidase
83605	CCM2	cerebral cavernous malformation 2
83660	TLN2	talin 2
83692	CD99L2	CD99 molecule-like 2
837	CASP4	caspase 4, apoptosis-related cysteine peptidase
83700	JAM3	junctional adhesion molecule 3
83706	FERMT3	fermitin family member 3
8373	IFIT1P1	interferon-induced protein with tetratricopeptide repeats 1 pseudogene 1
83737	ITCH	itchy E3 ubiquitin protein ligase homolog (mouse)
838	CASP5	caspase 5, apoptosis-related cysteine peptidase
8382	NME5	non-metastatic cells 5, protein expressed in (nucleoside-diphosphate kinase)
83861	RSPH3	radial spoke 3 homolog (Chlamydomonas)
83871	RAB34	RAB34, member RAS oncogene family
839	CASP6	caspase 6, apoptosis-related cysteine peptidase
83903	GSG2	germ cell associated 2 (haspin)
83941	TM2D1	TM2 domain containing 1
83953	FCAMR	Fc receptor, IgA, IgM, high affinity
83982	IFI27L2	interferon, alpha-inducible protein 27-like 2
83986	ITFG3	integrin alpha FG-GAP repeat containing 3
8399	PLA2G10	phospholipase A2, group X
840	CASP7	caspase 7, apoptosis-related cysteine peptidase
84002	B3GNT5	UDP-GlcNAc:betaGal beta-1,3-N-acetylglucosaminyltransferase 5
84033	OBSCN	obscurin, cytoskeletal calmodulin and titin-interacting RhoGEF
84034	EMILIN2	elastin microfibril interfacer 2
8404	SPARCL1	SPARC-like 1 (hevin)
84059	GPR98	G protein-coupled receptor 98
8406	SRPX	sushi-repeat containing protein, X-linked
84062	DTNBP1	dystrobrevin binding protein 1
84063	KIRREL2	kin of IRRE like 2 (Drosophila)
84086	IGLCOR22-2	immunoglobulin lambda constant/OR22-2 (pseudogene)
84087	IGLVIVOR22-2	immunoglobulin lambda variable (IV)/OR22-2 (pseudogene)
84088	IGLVIVOR22-1	immunoglobulin lambda variable (IV)/OR22-1 (pseudogene)
84089	IGLVVII-41-1	immunoglobulin lambda variable (VII)-41-1 (pseudogene)
84090	IGLVVI-25-1	immunoglobulin lambda variable (VI)-25-1 (pseudogene)
84091	IGLVVI-22-1	immunoglobulin lambda variable (VI)-22-1 (pseudogene)
84096	IGLCOR22-1	immunoglobulin lambda constant/OR22-1 (pseudogene)

84097	IGLVIV-66-1	immunoglobulin lambda variable (IV)-66-1 (pseudogene)
841	CASP8	caspase 8, apoptosis-related cysteine peptidase
84100	ARL6	ADP-ribosylation factor-like 6
84141	FAM176A	family with sequence similarity 176, member A
84148	MYST1	MYST histone acetyltransferase 1
84159	ARID5B	AT rich interactive domain 5B (MRF1-like)
8416	ANXA9	annexin A9
84166	NLRC5	NLR family, CARD domain containing 5
84168	ANTXR1	anthrax toxin receptor 1
84173	ELMOD3	ELMO/CED-12 domain containing 3
84174	SLA2	Src-like-adaptor 2
842	CASP9	caspase 9, apoptosis-related cysteine peptidase
84231	TRAF7	TNF receptor-associated factor 7
8425	LTBP4	latent transforming growth factor beta binding protein 4
84251	SGIP1	SH3-domain GRB2-like (endophilin) interacting protein 1
84260	TCHP	trichoplein, keratin filament binding
84262	PSMG3	proteasome (prosome, macropain) assembly chaperone 3
8428	STK24	serine/threonine kinase 24
84282	RNF135	ring finger protein 135
84287	ZDHHC16	zinc finger, DHHC-type containing 16
843	CASP10	caspase 10, apoptosis-related cysteine peptidase
84306	PDCD2L	programmed cell death 2-like
8435	SOAT2	sterol O-acyltransferase 2
8436	SDPR	serum deprivation response
844	CASQ1	calsequestrin 1 (fast-twitch, skeletal muscle)
8440	NCK2	NCK adaptor protein 2
84433	CARD11	caspase recruitment domain family, member 11
84440	RAB11FIP4	RAB11 family interacting protein 4 (class II)
84447	SYVN1	synovial apoptosis inhibitor 1, synoviolin
8445	DYRK2	dual-specificity tyrosine-(Y)-phosphorylation regulated kinase 2
84466	MEGF10	multiple EGF-like-domains 10
8447	DOC2B	double C2-like domains, beta
8448	DOC2A	double C2-like domains, alpha
8451	CUL4A	cullin 4A
8452	CUL3	cullin 3
84524	ZC3H8	zinc finger CCCH-type containing 8
8453	CUL2	cullin 2

8454	CUL1	cullin 1
8456	FOXN1	forkhead box N1
84565	LCS1	lymphedema-cholestasis syndrome 1
8462	KLF11	Kruppel-like factor 11
84632	AFAP1L2	actin filament associated protein 1-like 2
84639	IL1F10	interleukin 1 family, member 10 (theta)
84641	HIATL1	hippocampus abundant transcript-like 1
84674	CARD6	caspase recruitment domain family, member 6
84678	KDM2B	lysine (K)-specific demethylase 2B
8468	FKBP6	FK506 binding protein 6, 36kDa
84681	HINT2	histidine triad nucleotide binding protein 2
847	CAT	catalase
84707	BEX2	brain expressed X-linked 2
8473	OGT	O-linked N-acetylglucosamine (GlcNAc) transferase (UDP-N-acetylglucosamine:polypeptide-N-acetylglucosaminyl transferase)
8477	GPR65	G protein-coupled receptor 65
84807	NFKBID	nuclear factor of kappa light polypeptide gene enhancer in B-cells inhibitor, delta
84812	PLCD4	phospholipase C, delta 4
84817	TXNDC17	thioredoxin domain containing 17
84818	IL17RC	interleukin 17 receptor C
8482	SEMA7A	semaphorin 7A, GPI membrane anchor (John Milton Hagen blood group)
84824	FCRLA	Fc receptor-like A
84868	HAVCR2	hepatitis A virus cellular receptor 2
84876	ORAI1	ORAI calcium release-activated calcium modulator 1
84883	AIFM2	apoptosis-inducing factor, mitochondrion-associated, 2
84897	TBRG1	transforming growth factor beta regulator 1
84901	NFATC2IP	nuclear factor of activated T-cells, cytoplasmic, calcineurin-dependent 2 interacting protein
8491	MAP4K3	mitogen-activated protein kinase kinase kinase kinase 3
84941	HSH2D	hematopoietic SH2 domain containing
84944	MAEL	maelstrom homolog (Drosophila)
84951	TNS4	tensin 4
84957	RELT	RELT tumor necrosis factor receptor
84958	SYTL1	synaptotagmin-like 1
8496	PPFIBP1	PTPRF interacting protein, binding protein 1 (liprin beta 1)
84962	JUB	jub, ajuba homolog (Xenopus laevis)
84993	UBL7	ubiquitin-like 7 (bone marrow stromal cell-derived)
8502	PKP4	plakophilin 4

8503	PIK3R3	phosphoinositide-3-kinase, regulatory subunit 3 (gamma)
8506	CNTNAP1	contactin associated protein 1
8515	ITGA10	integrin, alpha 10
8516	ITGA8	integrin, alpha 8
8517	IKBKG	inhibitor of kappa light polypeptide gene enhancer in B-cells, kinase gamma
8518	IKBKAP	inhibitor of kappa light polypeptide gene enhancer in B-cells, kinase complex-associated protein
8519	IFITM1	interferon induced transmembrane protein 1 (9-27)
8525	DGKZ	diacylglycerol kinase, zeta
8526	DGKE	diacylglycerol kinase, epsilon 64kDa
8527	DGKD	diacylglycerol kinase, delta 130kDa
85301	COL27A1	collagen, type XXVII, alpha 1
85302	FBF1	Fas (TNFRSF6) binding factor 1
8531	CSDA	cold shock domain protein A
85313	PPIL4	peptidylprolyl isomerase (cyclophilin)-like 4
85329	LGALS12	lectin, galactoside-binding, soluble, 12
8535	CBX4	chromobox homolog 4
85359	DGCR6L	DiGeorge syndrome critical region gene 6-like
85366	MYLK2	myosin light chain kinase 2
8539	API5	apoptosis inhibitor 5
85409	NKD2	naked cuticle homolog 2 (Drosophila)
85439	STON2	stonin 2
85440	DOCK7	dedicator of cytokinesis 7
85445	CNTNAP4	contactin associated protein-like 4
8546	AP3B1	adaptor-related protein complex 3, beta 1 subunit
8547	FCN3	ficolin (collagen/fibrinogen domain containing) 3 (Hakata antigen)
85477	SCIN	scinderin
85480	TSLP	thymic stromal lymphopoietin
8554	PIAS1	protein inhibitor of activated STAT, 1
8563	THOC5	THO complex 5
8565	YARS	tyrosyl-tRNA synthetase
8567	MADD	MAP-kinase activating death domain
8569	MKNK1	MAP kinase interacting serine/threonine kinase 1
857	CAV1	caveolin 1, caveolae protein, 22kDa
8573	CASK	calcium/calmodulin-dependent serine protein kinase (MAGUK family)
8575	PRKRA	protein kinase, interferon-inducible double stranded RNA dependent activator
8578	SCARF1	scavenger receptor class F, member 1
8581	LY6D	lymphocyte antigen 6 complex, locus D

859	CAV3	caveolin 3
8600	TNFSF11	tumor necrosis factor (ligand) superfamily, member 11
861	RUNX1	runt-related transcription factor 1
8611	PPAP2A	phosphatidic acid phosphatase type 2A
8613	PPAP2B	phosphatidic acid phosphatase type 2B
8618	CADPS	Ca++-dependent secretion activator
8624	PSMG1	proteasome (prosome, macropain) assembly chaperone 1
8626	TP63	tumor protein p63
863	CBFA2T3	core-binding factor, runt domain, alpha subunit 2; translocated to, 3
8631	SKAP1	src kinase associated phosphoprotein 1
8633	UNC5C	unc-5 homolog C (C. elegans)
8638	OASL	2'-5'-oligoadenylate synthetase-like
8639	AOC3	amine oxidase, copper containing 3 (vascular adhesion protein 1)
864	RUNX3	runt-related transcription factor 3
8641	PCDHGB4	protocadherin gamma subfamily B, 4
8642	DCHS1	dachsous 1 (Drosophila)
8644	AKR1C3	aldo-keto reductase family 1, member C3 (3-alpha hydroxysteroid dehydrogenase, type II)
8646	CHRD	chordin
8651	SOCS1	suppressor of cytokine signaling 1
8654	PDE5A	phosphodiesterase 5A, cGMP-specific
8655	DYNLL1	dynein, light chain, LC8-type 1
8660	IRS2	insulin receptor substrate 2
867	CBL	Cas-Br-M (murine) ecotropic retroviral transforming sequence
8678	BECN1	beclin 1, autophagy related
868	CBLB	Cas-Br-M (murine) ecotropic retroviral transforming sequence b
8682	PEA15	phosphoprotein enriched in astrocytes 15
8685	MARCO	macrophage receptor with collagenous structure
8691	IDDM17	insulin-dependent diabetes mellitus 17
87	ACTN1	actinin, alpha 1
8706	B3GALNT1	beta-1,3-N-acetylgalactosaminyltransferase 1 (globoside blood group)
8712	PAGE1	P antigen family, member 1 (prostate associated)
8717	TRADD	TNFRSF1A-associated via death domain
8718	TNFRSF25	tumor necrosis factor receptor superfamily, member 25
8722	CTSF	cathepsin F
8723	SNX4	sorting nexin 4
8724	SNX3	sorting nexin 3
8727	CTNNAL1	catenin (cadherin-associated protein), alpha-like 1

873	CBR1	carbonyl reductase 1
8737	RIPK1	receptor (TNFRSF)-interacting serine-threonine kinase 1
8738	CRADD	CASP2 and RIPK1 domain containing adaptor with death domain
8739	HRK	harakiri, BCL2 interacting protein (contains only BH3 domain)
8740	TNFSF14	tumor necrosis factor (ligand) superfamily, member 14
8741	TNFSF13	tumor necrosis factor (ligand) superfamily, member 13
8742	TNFSF12	tumor necrosis factor (ligand) superfamily, member 12
8743	TNFSF10	tumor necrosis factor (ligand) superfamily, member 10
8744	TNFSF9	tumor necrosis factor (ligand) superfamily, member 9
8745	ADAM23	ADAM metallopeptidase domain 23
8751	ADAM15	ADAM metallopeptidase domain 15
8754	ADAM9	ADAM metallopeptidase domain 9
8763	CD164	CD164 molecule, sialomucin
8764	TNFRSF14	tumor necrosis factor receptor superfamily, member 14 (herpesvirus entry mediator)
8766	RAB11A	RAB11A, member RAS oncogene family
8767	RIPK2	receptor-interacting serine-threonine kinase 2
8771	TNFRSF6B	tumor necrosis factor receptor superfamily, member 6b, decoy
8772	FADD	Fas (TNFRSF6)-associated via death domain
8773	SNAP23	synaptosomal-associated protein, 23kDa
8778	SIGLEC5	sialic acid binding Ig-like lectin 5
8784	TNFRSF18	tumor necrosis factor receptor superfamily, member 18
8792	TNFRSF11A	tumor necrosis factor receptor superfamily, member 11a, NFKB activator
8793	TNFRSF10D	tumor necrosis factor receptor superfamily, member 10d, decoy with truncated death domain
8794	TNFRSF10C	tumor necrosis factor receptor superfamily, member 10c, decoy without an intracellular domain
8795	TNFRSF10B	tumor necrosis factor receptor superfamily, member 10b
8797	TNFRSF10A	tumor necrosis factor receptor superfamily, member 10a
88	ACTN2	actinin, alpha 2
8800	PEX11A	peroxisomal biogenesis factor 11 alpha
8805	TRIM24	tripartite motif containing 24
8807	IL18RAP	interleukin 18 receptor accessory protein
8808	IL1RL2	interleukin 1 receptor-like 2
8809	IL18R1	interleukin 18 receptor 1
882	CCAL1	chondrocalcinosis 1 (calcium pyrophosphate-deposition disease, early onset osteoarthritis)
8825	LIN7A	lin-7 homolog A (C. elegans)
8828	NRP2	neuropilin 2
8829	NRP1	neuropilin 1
8832	CD84	CD84 molecule

8835	SOCS2	suppressor of cytokine signaling 2
8837	CFLAR	CASP8 and FADD-like apoptosis regulator
8839	WISP2	WNT1 inducible signaling pathway protein 2
8840	WISP1	WNT1 inducible signaling pathway protein 1
8841	HDAC3	histone deacetylase 3
8847	DLEU2	deleted in lymphocytic leukemia 2 (non-protein coding)
885	CCK	cholecystokinin
8851	CDK5R1	cyclin-dependent kinase 5, regulatory subunit 1 (p35)
8852	AKAP4	A kinase (PRKA) anchor protein 4
8854	ALDH1A2	aldehyde dehydrogenase 1 family, member A2
8857	FCGBP	Fc fragment of IgG binding protein
886	CCKAR	cholecystokinin A receptor
8861	LDB1	LIM domain binding 1
8867	SYNJ1	synaptojanin 1
887	CCKBR	cholecystokinin B receptor
8870	IER3	immediate early response 3
8874	ARHGEF7	Rho guanine nucleotide exchange factor (GEF) 7
8876	VNN1	vanin 1
8877	SPHK1	sphingosine kinase 1
8878	SQSTM1	sequestosome 1
8879	SGPL1	sphingosine-1-phosphate lyase 1
8883	NAE1	NEDD8 activating enzyme E1 subunit 1
8887	TAX1BP1	Tax1 (human T-cell leukemia virus type I) binding protein 1
889	KRIT1	KRIT1, ankyrin repeat containing
89	ACTN3	actinin, alpha 3
8905	AP1S2	adaptor-related protein complex 1, sigma 2 subunit
8915	BCL10	B-cell CLL/lymphoma 10
8928	FOXH1	forkhead box H1
8930	MBD4	methyl-CpG binding domain protein 4
8935	SKAP2	src kinase associated phosphoprotein 2
8942	KYNU	kynureninase
8943	AP3D1	adaptor-related protein complex 3, delta 1 subunit
896	CCND3	cyclin D3
89765	RSPH1	radial spoke head 1 homolog (Chlamydomonas)
89770	IGHV3-30-5	immunoglobulin heavy variable 3-30-5
89780	WNT3A	wingless-type MMTV integration site family, member 3A
89781	HPS4	Hermansky-Pudlak syndrome 4

89782	LMLN	leishmanolysin-like (metallopeptidase M8 family)
89790	SIGLEC10	sialic acid binding Ig-like lectin 10
898	CCNE1	cyclin E1
89846	FGD3	FYVE, RhoGEF and PH domain containing 3
89848	FCHSD1	FCH and double SH3 domains 1
89857	KLHL6	kelch-like 6 (Drosophila)
89858	SIGLEC12	sialic acid binding Ig-like lectin 12
8986	RPS6KA4	ribosomal protein S6 kinase, 90kDa, polypeptide 4
89884	LHX4	LIM homeobox 4
89941	RHOT2	ras homolog gene family, member T2
8995	TNFSF18	tumor necrosis factor (ligand) superfamily, member 18
8996	NOL3	nucleolar protein 3 (apoptosis repressor with CARD domain)
8997	KALRN	kalirin, RhoGEF kinase
90	ACVR1	activin A receptor, type I
900	CCNG1	cyclin G1
90011	KIR3DX1	killer cell immunoglobulin-like receptor, three domains, X1
9002	F2RL3	coagulation factor II (thrombin) receptor-like 3
9020	MAP3K14	mitogen-activated protein kinase kinase kinase 14
9021	SOCS3	suppressor of cytokine signaling 3
90249	UNC5A	unc-5 homolog A (C. elegans)
9026	HIP1R	huntingtin interacting protein 1 related
90273	CEACAM21	carcinoembryonic antigen-related cell adhesion molecule 21
90293	KLHL13	kelch-like 13 (Drosophila)
90326	THAP3	THAP domain containing, apoptosis associated protein 3
9034	CCRL2	chemokine (C-C motif) receptor-like 2
9037	SEMA5A	sema domain, seven thrombospondin repeats (type 1 and type 1-like), transmembrane domain (TM) and short cytoplasmic domain, (semaphorin) 5A
90410	IFT20	intraflagellar transport 20 homolog (Chlamydomonas)
90427	BMF	Bcl2 modifying factor
9046	DOK2	docking protein 2, 56kDa
9051	PSTPIP1	proline-serine-threonine phosphatase interacting protein 1
90525	SHF	Src homology 2 domain containing F
9055	PRC1	protein regulator of cytokinesis 1
9056	SLC7A7	solute carrier family 7 (cationic amino acid transporter, y+ system), member 7
9057	SLC7A6	solute carrier family 7 (cationic amino acid transporter, y+ system), member 6
9058	SLC13A2	solute carrier family 13 (sodium-dependent dicarboxylate transporter), member 2
9066	SYT7	synaptotagmin VII

90665	TBL1Y	transducin (beta)-like 1, Y-linked
90678	LRSAM1	leucine rich repeat and sterile alpha motif containing 1
9068	ANGPTL1	angiopoietin-like 1
9069	CLDN12	claudin 12
9071	CLDN10	claudin 10
9073	CLDN8	claudin 8
9074	CLDN6	claudin 6
9075	CLDN2	claudin 2
9076	CLDN1	claudin 1
9080	CLDN9	claudin 9
9082	XKRY	XK, Kell blood group complex subunit-related, Y-linked
90865	IL33	interleukin 33
909	CD1A	CD1a molecule
9092	SART1	squamous cell carcinoma antigen recognized by T cells
9093	DNAJA3	DnaJ (Hsp40) homolog, subfamily A, member 3
90952	ESAM	endothelial cell adhesion molecule
9097	USP14	ubiquitin specific peptidase 14 (tRNA-guanine transglycosylase)
91	ACVR1B	activin A receptor, type IB
910	CD1B	CD1b molecule
9103	FCGR2C	Fc fragment of IgG, low affinity IIc, receptor for (CD32) (gene/pseudogene)
911	CD1C	CD1c molecule
91156	IGFN1	immunoglobulin-like and fibronectin type III domain containing 1
91179	SCARF2	scavenger receptor class F, member 2
912	CD1D	CD1d molecule
9123	SLC16A3	solute carrier family 16, member 3 (monocarboxylic acid transporter 4)
9125	RQCD1	RCD1 required for cell differentiation1 homolog (S. pombe)
913	CD1E	CD1e molecule
9131	AIFM1	apoptosis-inducing factor, mitochondrion-associated, 1
91316	LOC91316	glucuronidase, beta/immunoglobulin lambda-like polypeptide 1 pseudogene
9133	CCNB2	cyclin B2
9135	RABEP1	rabaptin, RAB GTPase binding effector protein 1
91353	IGLL3P	immunoglobulin lambda-like polypeptide 3, pseudogene
914	CD2	CD2 molecule
9140	ATG12	ATG12 autophagy related 12 homolog (S. cerevisiae)
9141	PDCD5	programmed cell death 5
915	CD3D	CD3d molecule, delta (CD3-TCR complex)
9156	EXO1	exonuclease 1

91584	PLXNA4	plexin A4
916	CD3E	CD3e molecule, epsilon (CD3-TCR complex)
91607	SLFN11	schlafen family member 11
9162	DGKI	diacylglycerol kinase, iota
9163	AMLCR2	acute myeloid leukemia chromosome region 2
91653	BOC	Boc homolog (mouse)
9166	EBAG9	estrogen receptor binding site associated, antigen, 9
91662	NLRP12	NLR family, pyrin domain containing 12
91663	MYADM	myeloid-associated differentiation marker
917	CD3G	CD3g molecule, gamma (CD3-TCR complex)
9173	IL1RL1	interleukin 1 receptor-like 1
9180	OSMR	oncostatin M receptor
91807	MYLK3	myosin light chain kinase 3
9181	ARHGEF2	Rho/Rac guanine nucleotide exchange factor (GEF) 2
919	CD247	CD247 molecule
9191	DEDD	death effector domain containing
91937	TIMD4	T-cell immunoglobulin and mucin domain containing 4
92	ACVR2A	activin A receptor, type IIA
920	CD4	CD4 molecule
921	CD5	CD5 molecule
9210	BMP15	bone morphogenetic protein 15
9212	AURKB	aurora kinase B
9214	FAIM3	Fas apoptotic inhibitory molecule 3
92140	MTDH	metadherin
92196	DAPL1	death associated protein-like 1
922	CD5L	CD5 molecule-like
9220	TIAF1	TGFB1-induced anti-apoptotic factor 1
92211	CDHR1	cadherin-related family member 1
9223	MAGI1	membrane associated guanylate kinase, WW and PDZ domain containing 1
92292	GLYATL1	glycine-N-acyltransferase-like 1
923	CD6	CD6 molecule
92304	SCGB3A1	secretoglobin, family 3A, member 1
9231	DLG5	discs, large homolog 5 (Drosophila)
92342	METTL18	methyltransferase like 18
9235	IL32	interleukin 32
9238	TBRG4	transforming growth factor beta regulator 4
924	CD7	CD7 molecule

9240	PNMA1	paraneoplastic antigen MA1
9241	NOG	noggin
9244	CRLF1	cytokine receptor-like factor 1
9246	UBE2L6	ubiquitin-conjugating enzyme E2L 6
9247	GCM2	glial cells missing homolog 2 (Drosophila)
925	CD8A	CD8a molecule
9252	RPS6KA5	ribosomal protein S6 kinase, 90kDa, polypeptide 5
9253	NUMBL	numb homolog (Drosophila)-like
9255	AIMP1	aminoacyl tRNA synthetase complex-interacting multifunctional protein 1
926	CD8B	CD8b molecule
9260	PDLIM7	PDZ and LIM domain 7 (enigma)
92609	TIMM50	translocase of inner mitochondrial membrane 50 homolog (S. cerevisiae)
9262	STK17B	serine/threonine kinase 17b
9263	STK17A	serine/threonine kinase 17a
9265	CYTH3	cytohesin 3
9266	CYTH2	cytohesin 2
9267	CYTH1	cytohesin 1
927	CD8BP	CD8b molecule pseudogene
9270	ITGB1BP1	integrin beta 1 binding protein 1
92737	DNER	delta/notch-like EGF repeat containing
9274	BCL7C	B-cell CLL/lymphoma 7C
9275	BCL7B	B-cell CLL/lymphoma 7B
928	CD9	CD9 molecule
9289	GPR56	G protein-coupled receptor 56
929	CD14	CD14 molecule
93	ACVR2B	activin A receptor, type IIB
930	CD19	CD19 molecule
9306	SOCS6	suppressor of cytokine signaling 6
9308	CD83	CD83 molecule
931	MS4A1	membrane-spanning 4-domains, subfamily A, member 1
9314	KLF4	Kruppel-like factor 4 (gut)
9315	C5orf13	chromosome 5 open reading frame 13
93185	IGSF8	immunoglobulin superfamily, member 8
9320	TRIP12	thyroid hormone receptor interactor 12
9322	TRIP10	thyroid hormone receptor interactor 10
933	CD22	CD22 molecule
9332	CD163	CD163 molecule

9341	VAMP3	vesicle-associated membrane protein 3 (cellubrevin)
9342	SNAP29	synaptosomal-associated protein, 29kDa
9344	TAOK2	TAO kinase 2
935	CD24L1	CD24 molecule-like 1
9350	CER1	cerberus 1, cysteine knot superfamily, homolog (Xenopus laevis)
9353	SLIT2	slit homolog 2 (Drosophila)
9358	ITGBL1	integrin, beta-like 1 (with EGF-like repeat domains)
936	CD24P2	CD24 molecule pseudogene 2
9360	PPIG	peptidylprolyl isomerase G (cyclophilin G)
93664	CADPS2	Ca++-dependent secretion activator 2
9368	SLC9A3R1	solute carrier family 9 (sodium/hydrogen exchanger), member 3 regulator 1
9369	NRXN3	neurexin 3
937	CD24P3	CD24 molecule pseudogene 3
9370	ADIPOQ	adiponectin, C1Q and collagen domain containing
9372	ZFYVE9	zinc finger, FYVE domain containing 9
9378	NRXN1	neurexin 1
9379	NRXN2	neurexin 2
938	CD24P4	CD24 molecule pseudogene 4
9381	OTOF	otoferlin
939	CD27	CD27 molecule
9392	TGFBRAP1	transforming growth factor, beta receptor associated protein 1
93978	CLEC6A	C-type lectin domain family 6, member A
9398	CD101	CD101 molecule
94	ACVRL1	activin A receptor type II-like 1
940	CD28	CD28 molecule
9402	GRAP2	GRB2-related adaptor protein 2
94025	MUC16	mucin 16, cell surface associated
9404	LPXN	leupaxin
94059	LENG9	leukocyte receptor cluster (LRC) member 9
941	CD80	CD80 molecule
94121	SYTL4	synaptotagmin-like 4
94122	SYTL5	synaptotagmin-like 5
9414	TJP2	tight junction protein 2 (zona occludens 2)
942	CD86	CD86 molecule
9421	HAND1	heart and neural crest derivatives expressed 1
9423	NTN1	netrin 1
94234	FOXQ1	forkhead box Q1

94241	TP53INP1	tumor protein p53 inducible nuclear protein 1
943	TNFRSF8	tumor necrosis factor receptor superfamily, member 8
9436	NCR2	natural cytotoxicity triggering receptor 2
9437	NCR1	natural cytotoxicity triggering receptor 1
944	TNFSF8	tumor necrosis factor (ligand) superfamily, member 8
9445	ITM2B	integral membrane protein 2B
9447	AIM2	absent in melanoma 2
9448	MAP4K4	mitogen-activated protein kinase kinase kinase kinase 4
945	CD33	CD33 molecule
9450	LY86	lymphocyte antigen 86
9451	EIF2AK3	eukaryotic translation initiation factor 2-alpha kinase 3
9459	ARHGEF6	Rac/Cdc42 guanine nucleotide exchange factor (GEF) 6
946	SIGLEC6	sialic acid binding Ig-like lectin 6
9463	PICK1	protein interacting with PRKCA 1
9464	HAND2	heart and neural crest derivatives expressed 2
9466	IL27RA	interleukin 27 receptor, alpha
9469	CHST3	carbohydrate (chondroitin 6) sulfotransferase 3
947	CD34	CD34 molecule
9473	C1orf38	chromosome 1 open reading frame 38
9474	ATG5	ATG5 autophagy related 5 homolog (S. cerevisiae)
9475	ROCK2	Rho-associated, coiled-coil containing protein kinase 2
9479	MAPK8IP1	mitogen-activated protein kinase 8 interacting protein 1
948	CD36	CD36 molecule (thrombospondin receptor)
9480	ONECUT2	one cut homeobox 2
9486	CHST10	carbohydrate sulfotransferase 10
949	SCARB1	scavenger receptor class B, member 1
9491	PSMF1	proteasome (prosome, macropain) inhibitor subunit 1 (PI31)
9493	KIF23	kinesin family member 23
950	SCARB2	scavenger receptor class B, member 2
9500	MAGED1	melanoma antigen family D, 1
9501	RPH3AL	rabphilin 3A-like (without C2 domains)
951	CD37	CD37 molecule
9510	ADAMTS1	ADAM metallopeptidase with thrombospondin type 1 motif, 1
9515	STXBP5L	syntaxin binding protein 5-like
9516	LITAF	lipopolysaccharide-induced TNF factor
9518	GDF15	growth differentiation factor 15
952	CD38	CD38 molecule

9521	EEF1E1	eukaryotic translation elongation factor 1 epsilon 1
9529	BAG5	BCL2-associated athanogene 5
953	ENTPD1	ectonucleoside triphosphate diphosphohydrolase 1
9530	BAG4	BCL2-associated athanogene 4
9531	BAG3	BCL2-associated athanogene 3
9532	BAG2	BCL2-associated athanogene 2
9536	PTGES	prostaglandin E synthase
9538	EI24	etoposide induced 2.4 mRNA
954	ENTPD2	ectonucleoside triphosphate diphosphohydrolase 2
9540	TP53I3	tumor protein p53 inducible protein 3
9543	IGDCC3	immunoglobulin superfamily, DCC subclass, member 3
9545	RAB3D	RAB3D, member RAS oncogene family
9547	CXCL14	chemokine (C-X-C motif) ligand 14
9560	CCL4L1	chemokine (C-C motif) ligand 4-like 1
9564	BCAR1	breast cancer anti-estrogen resistance 1
9572	NR1D1	nuclear receptor subfamily 1, group D, member 1
9573	GDF3	growth differentiation factor 3
9577	BRE	brain and reproductive organ-expressed (TNFRSF1A modulator)
958	CD40	CD40 molecule, TNF receptor superfamily member 5
959	CD40LG	CD40 ligand
9595	CYTIP	cytohesin 1 interacting protein
960	CD44	CD44 molecule (Indian blood group)
9603	NFE2L3	nuclear factor (erythroid-derived 2)-like 3
961	CD47	CD47 molecule
9610	RIN1	Ras and Rab interactor 1
9611	NCOR1	nuclear receptor corepressor 1
9616	RNF7	ring finger protein 7
9618	TRAF4	TNF receptor-associated factor 4
9619	ABCG1	ATP-binding cassette, sub-family G (WHITE), member 1
962	CD48	CD48 molecule
9620	CELSR1	cadherin, EGF LAG seven-pass G-type receptor 1 (flamingo homolog, Drosophila)
9623	TCL1B	T-cell leukemia/lymphoma 1B
9625	AATK	apoptosis-associated tyrosine kinase
963	CD53	CD53 molecule
9630	GNA14	guanine nucleotide binding protein (G protein), alpha 14
9635	CLCA2	chloride channel accessory 2
9636	ISG15	ISG15 ubiquitin-like modifier

9637	FEZ2	fasciculation and elongation protein zeta 2 (zygin II)
9638	FEZ1	fasciculation and elongation protein zeta 1 (zygin I)
9641	IKBKE	inhibitor of kappa light polypeptide gene enhancer in B-cells, kinase epsilon
9647	PPM1F	protein phosphatase, Mg2+/Mn2+ dependent, 1F
965	CD58	CD58 molecule
9655	SOCS5	suppressor of cytokine signaling 5
9656	MDC1	mediator of DNA-damage checkpoint 1
966	CD59	CD59 molecule, complement regulatory protein
96626	LIMS3	LIM and senescent cell antigen-like domains 3
967	CD63	CD63 molecule
9671	WSCD2	WSC domain containing 2
9672	SDC3	syndecan 3
968	CD68	CD68 molecule
9685	CLINT1	clathrin interactor 1
969	CD69	CD69 molecule
9690	UBE3C	ubiquitin protein ligase E3C
9699	RIMS2	regulating synaptic membrane exocytosis 2
970	CD70	CD70 molecule
9700	ESPL1	extra spindle pole bodies homolog 1 (S. cerevisiae)
9708	PCDHGA8	protocadherin gamma subfamily A, 8
9709	HERPUD1	homocysteine-inducible, endoplasmic reticulum stress-inducible, ubiquitin-like domain member 1
971	CD72	CD72 molecule
9711	KIAA0226	KIAA0226
9719	ADAMTSL2	ADAMTS-like 2
972	CD74	CD74 molecule, major histocompatibility complex, class II invariant chain
9722	NOS1AP	nitric oxide synthase 1 (neuronal) adaptor protein
9723	SEMA3E	sema domain, immunoglobulin domain (Ig), short basic domain, secreted, (semaphorin) 3E
9727	RAB11FIP3	RAB11 family interacting protein 3 (class II)
973	CD79A	CD79a molecule, immunoglobulin-associated alpha
9730	VPRBP	Vpr (HIV-1) binding protein
9732	DOCK4	dedicator of cytokinesis 4
9733	SART3	squamous cell carcinoma antigen recognized by T cells 3
9734	HDAC9	histone deacetylase 9
9738	CCP110	centriolar coiled coil protein 110kDa
974	CD79B	CD79b molecule, immunoglobulin-associated beta
9742	IFT140	intraflagellar transport 140 homolog (Chlamydomonas)
9746	CLSTN3	calsyntenin 3

9748	SLK	STE20-like kinase
975	CD81	CD81 molecule
9751	SNPH	syntaphilin
9752	PCDHA9	protocadherin alpha 9
9757	MLL4	myeloid/lymphoid or mixed-lineage leukemia 4
9759	HDAC4	histone deacetylase 4
976	CD97	CD97 molecule
9760	TOX	thymocyte selection-associated high mobility group box
9765	ZFYVE16	zinc finger, FYVE domain containing 16
977	CD151	CD151 molecule (Raph blood group)
9774	BCLAF1	BCL2-associated transcription factor 1
9783	RIMS3	regulating synaptic membrane exocytosis 3
9784	SNX17	sorting nexin 17
9788	MTSS1	metastasis suppressor 1
9805	SCRN1	secernin 1
9812	KIAA0141	KIAA0141
9821	RB1CC1	RB1-inducible coiled-coil 1
9826	ARHGEF11	Rho guanine nucleotide exchange factor (GEF) 11
9828	ARHGEF17	Rho guanine nucleotide exchange factor (GEF) 17
983	CDK1	cyclin-dependent kinase 1
9832	JAKMIP2	janus kinase and microtubule interacting protein 2
9839	ZEB2	zinc finger E-box binding homeobox 2
984	CDK11B	cyclin-dependent kinase 11B
9844	ELMO1	engulfment and cell motility 1
9846	GAB2	GRB2-associated binding protein 2
9854	C2CD2L	C2CD2-like
9860	LRIG2	leucine-rich repeats and immunoglobulin-like domains 2
9861	PSMD6	proteasome (prosome, macropain) 26S subunit, non-ATPase, 6
9865	TRIL	TLR4 interactor with leucine-rich repeats
9873	FCHSD2	FCH and double SH3 domains 2
989	SEPT7	septin 7
990	CDC6	cell division cycle 6 homolog (S. cerevisiae)
9902	MRC2	mannose receptor, C type 2
9903	KLHL21	kelch-like 21 (Drosophila)
9915	ARNT2	aryl-hydrocarbon receptor nuclear translocator 2
9936	CD302	CD302 molecule
9943	OXSR1	oxidative-stress responsive 1

9948	WDR1	WD repeat domain 1
9965	FGF19	fibroblast growth factor 19
9966	TNFSF15	tumor necrosis factor (ligand) superfamily, member 15
998	CDC42	cell division cycle 42 (GTP binding protein, 25kDa)
9984	THOC1	THO complex 1
999	CDH1	cadherin 1, type 1, E-cadherin (epithelial)
9993	DGCR2	DiGeorge syndrome critical region gene 2
9994	CASP8AP2	caspase 8 associated protein 2
